# Application of Cyclic Diaryliodonium Salts in the Synthesis of Axially Chiral Natural Product Analogues

**DOI:** 10.1021/acs.orglett.4c01308

**Published:** 2024-06-17

**Authors:** Moritz K. T. Klischan, Céline David, Daniel Grudzinski, Wolfgang Frey, Björn Stork, Jörg Pietruszka

**Affiliations:** † Heinrich-Heine-Universität Düsseldorf im Forschungszentrum Jülich, Mathematisch-Naturwissenschaftliche Fakultät, Institut für Bioorganische Chemie, 52428 Jülich, Germany; ‡ Institute of Molecular Medicine I, Medical Faculty and University Hospital Düsseldorf, Heinrich Heine University Düsseldorf, Universitätsstr. 1, 40225 Düsseldorf, Germany; § Institute of Organic Chemistry, University of Stuttgart, 70569 Stuttgart, Germany; ∥ Institut für Bio- und Geowissenschaften 1 (IBG-1: Biotechnologie), Forschungszentrum Jülich, 52428 Jülich, Germany

## Abstract

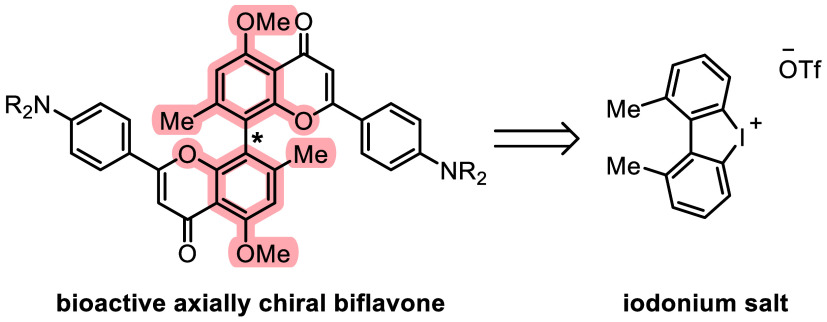

The application of cyclic diaryliodonium salts in the synthesis of bioactive natural product analogues was demonstrated. Axially chiral biaryls were obtained via the enantioselective ring opening of cyclic diaryliodonium salts. Regioselective borylation was key in accessing both enantiomers of a biphenol key intermediate in eight steps overall. 8,8″-Amino biflavones were synthesized, their bioactivity profiled, and the eutomer identified. The structure–activity relationship was probed.

The 8,8″-biflavones are a class of bioactive natural products. Though the naturally occurring cupressuflavone (CUF)^1,2^ has been evaluated biologically to a limited extent,^3−9^ non-natural analogues have so far hardly been investigated regarding their biological activity.^10,11^ The comparably simple yet biologically relevant flavone scaffold^12^ of 8,8″-biflavones in combination with the lack of a thorough biological profiling make this compound class of great interest for further investigations. Axially chiral biaryls and their application in natural product synthesis have been reviewed a number of times.^13,14^ Enantioselective total syntheses of CUF methyl ethers were established in the late 1990s by Zhang et al.,^15^ Lin et al.,^16^ and Li et al.^17^ Chiral auxiliaries were used to obtain the enantiopure products, which limited the scalability of their protocols.

The enantioselective construction of C–C bonds in biaryls poses a synthetic challenge.^18−20^ Our group approached this issue by the synthesis of biphenol **1** as a common C_2_-symmetrical building block in natural product synthesis (Scheme 1A).^21^ The usefulness of this building block has so far been shown in the total syntheses of di-*epi*-gonytolid A by deracemization (Scheme 1B)^21^ and isokotanine A by enzymatic kinetic resolution (Scheme 1C).^22^ In our previous study, we observed significant bioactivity for a racemic C_2_-symmetrical amino 8,8″-biflavone against both malignant human cell lines, as well as antimicrobial activity (Scheme 1D).^11^ Motivated by this earlier finding of a bioactive lead structure, we sought to explore a route that would provide us with both enantiomers of biphenol **1** in eight steps overall to synthesize the desired biflavones in a target-oriented library approach (Scheme 1E). In addition to cutting down on steps, a more modular approach enables access to further biarylic structures. Key to this strategy would be the use of cyclic diaryliodonium salts. The utility of these stereodynamic intermediates was first harnessed by the Gu group^23^ and has since been explored extensively in recent literature and used as well-established platforms that can be ring-opened enantioselectively using an abundant variety of nucleophiles compiled in a recent review.^24^ Electron-rich arenes are generally not well-accepted by standard conditions to provide iodonium salts,^25,26^ which makes these useful building blocks somewhat underexplored in the use of natural product synthesis. This gap will be bridged by leveraging the use of *meta*-selective iridium-catalyzed borylations to provide us with the 1,3,5-substitution pattern common in polyketide- and terpenoid-based natural products.^13,18,21^ Overall, using the borylations as handles for further diversification in addition to the plethora of ring-opening protocols available, this synthetic strategy enables access to a variety of possible target biaryls.

**Scheme 1:**
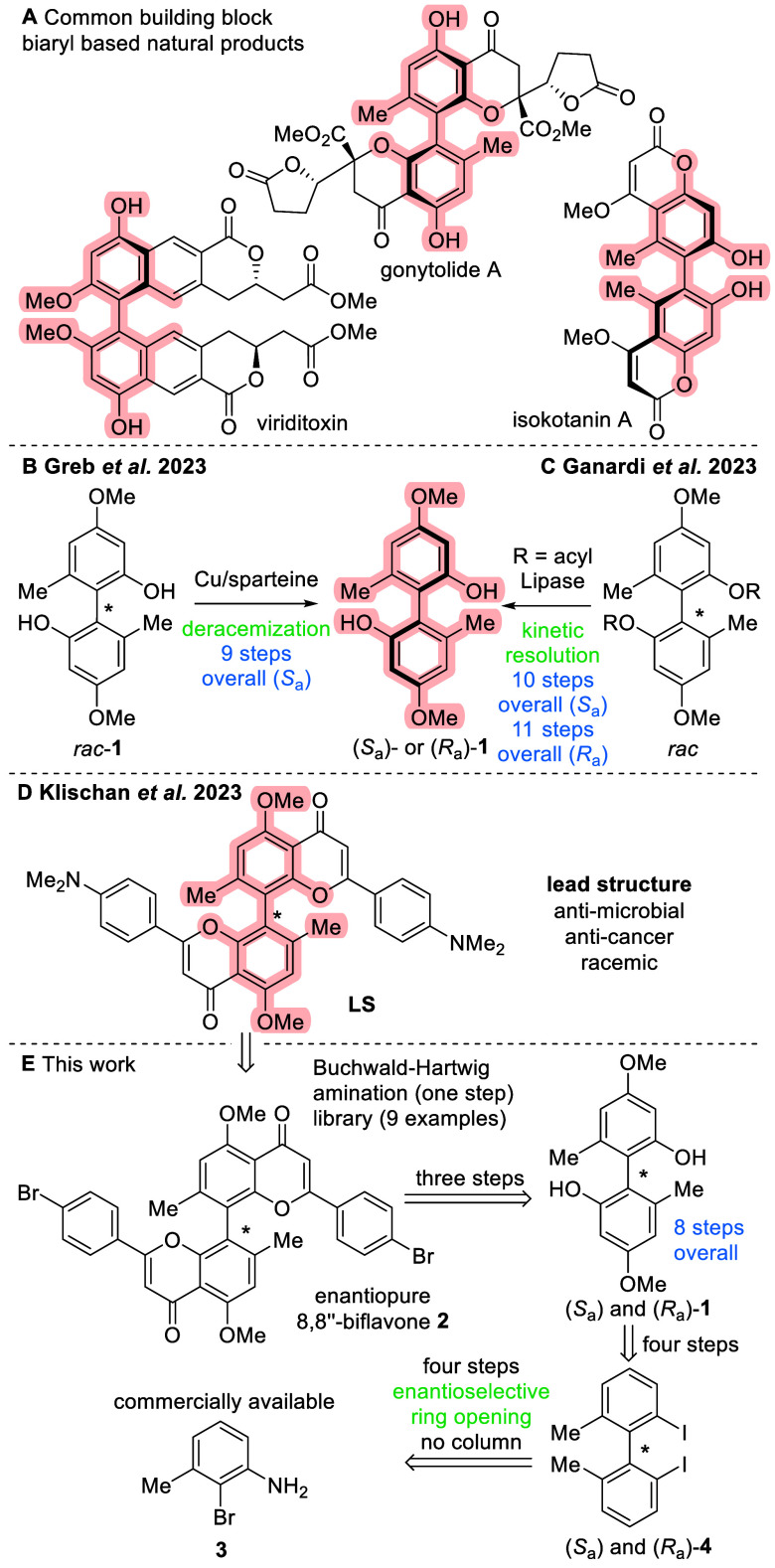
(A) Biphenol **1** as a Common Building Block in Natural Product Synthesis, (B) Synthesis of Biphenol (*S*_a_)-**1** by Deracemization, (C) Enzymatic Kinetic Resolution of Biphenol **1**, (D) Previously Identified Lead Structure (**LS**), and (E) Enantioselective Synthesis of Biflavones Involving Biphenol Building Block **1**

In our previous study,^11^ even though yields of the amino-substituted lead compound biflavone **LS** were low, brominated biflavone **2** could be obtained in high yields. Thus, starting from the readily available biflavone **2**,^11^ Buchwald–Hartwig amination using secondary and primary amines would give us access of racemic amino-biflavones in a single step to broaden the scope considerable (Scheme 1E). Moreover, both enantiomers of the most bioactive compound were to be synthesized. Starting from commercially available brominated arene **3**, enantiopure 2,2′-diiodobiaryl **4** could be accessed on multigram scale following an enantioselective ring-opening step. A sequence of four steps toward *meta*-selective methoxylation would yield biphenol **1**. Finally, the synthesis of biflavone **2** over three steps with subsequent Buchwald–Hartwig amination would ultimately provide both enantiomers of the desired amino biflavones.

Initial conditions of the sequence toward 2,2′-iodobiaryl **4** were based on protocols by Zhu et al. and Ke et al.^27,28^ The Suzuki coupling proceeded smoothly to provide us with the 2-amino biaryl **5** (not shown) as the hydrochloride salt **6** by avoiding column purification in a yield of 91% (Scheme 2). Following Suzuki coupling, desired 2-iodobiaryl **7** was obtained via a Sandmeyer reaction in a yield of 82% by filtration over silica. Cyclic diaryliodonium salt **8** was then isolated following an oxidative cyclization in a yield of 78%, again without column chromatographic isolation. Two methods exist for the enantioselective ring opening of iodonium salts using halides.^27,28^ By following a modified protocol of Zhu et al. making use of CH_2_Cl_2_ in combination with NaI (Table S1), both enantiomers of 2,2′-iodobiaryl **4** were obtained in yields of 82–94% with 91–94% ee, again avoiding the use of column chromatography.

**Scheme 2:**
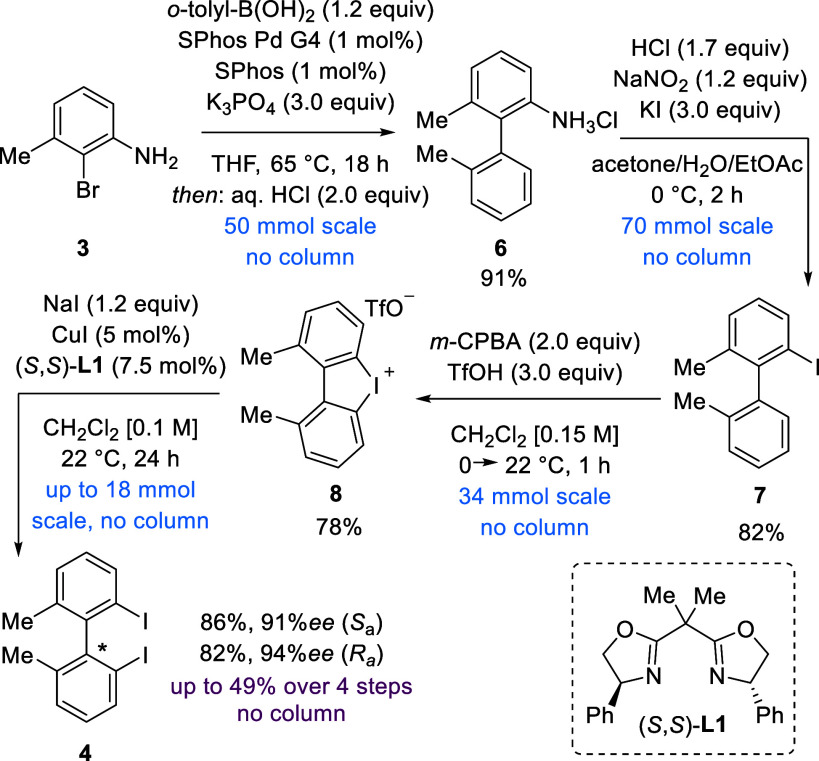
Synthesis Route toward Both Enantiomers of 2,2′-Diiodo Biaryl **4**

With both enantiomers of 2,2′-iodobiaryl **4** in hand, we continued with the *meta*-selective borylation. Sterically controlled Ir-catalyzed C–H activation established in the early 2000s by the Hartwig and Miyaura groups^29,30^ generates otherwise hard-to-access 1,3,5-substitution patterns.^31^ We sought to harness the power of this transformation by a double borylation and subsequent oxidation of the forming boronic acid ester **9** (Scheme 3). After a short screening (Table S2), we found that 3 mol % [Ir(COD)OMe]_2_ gave excellent yields of 98% on 10 mmol scale by filtration over silica. With a protocol for the borylation in hand, we investigated the oxidation of both boronic acid esters to generate biphenol **10** (Scheme 3). Oxone as the oxidant gave the desired product in yields of 82–85%. Thus, overoxidation of the aryl iodide was negligible in our case (Table S3). Afterward, 2,2′-diiodobiaryl **11** could be isolated in yields of 89–95% by double methyl protection of biphenol **10**. The high enantiomeric excess was not lowered by any of the steps in this sequence. To access biphenol **1**, we next investigated the twofold transformation of the aryl iodide into a phenol. After extensive screening (Table S4), the literature-known protocol by Ke et al.^27^ using metal–halogen exchange followed by the addition of freshly distilled nitrobenzene proved the most suitable method in providing biphenol **1**. Recrystallization enriched the enantiomeric excess for both (*R*_a_)- and (*S*_a_)-enantiomer (>99% ee) with overall yields of 47%, respectively.

**Scheme 3:**
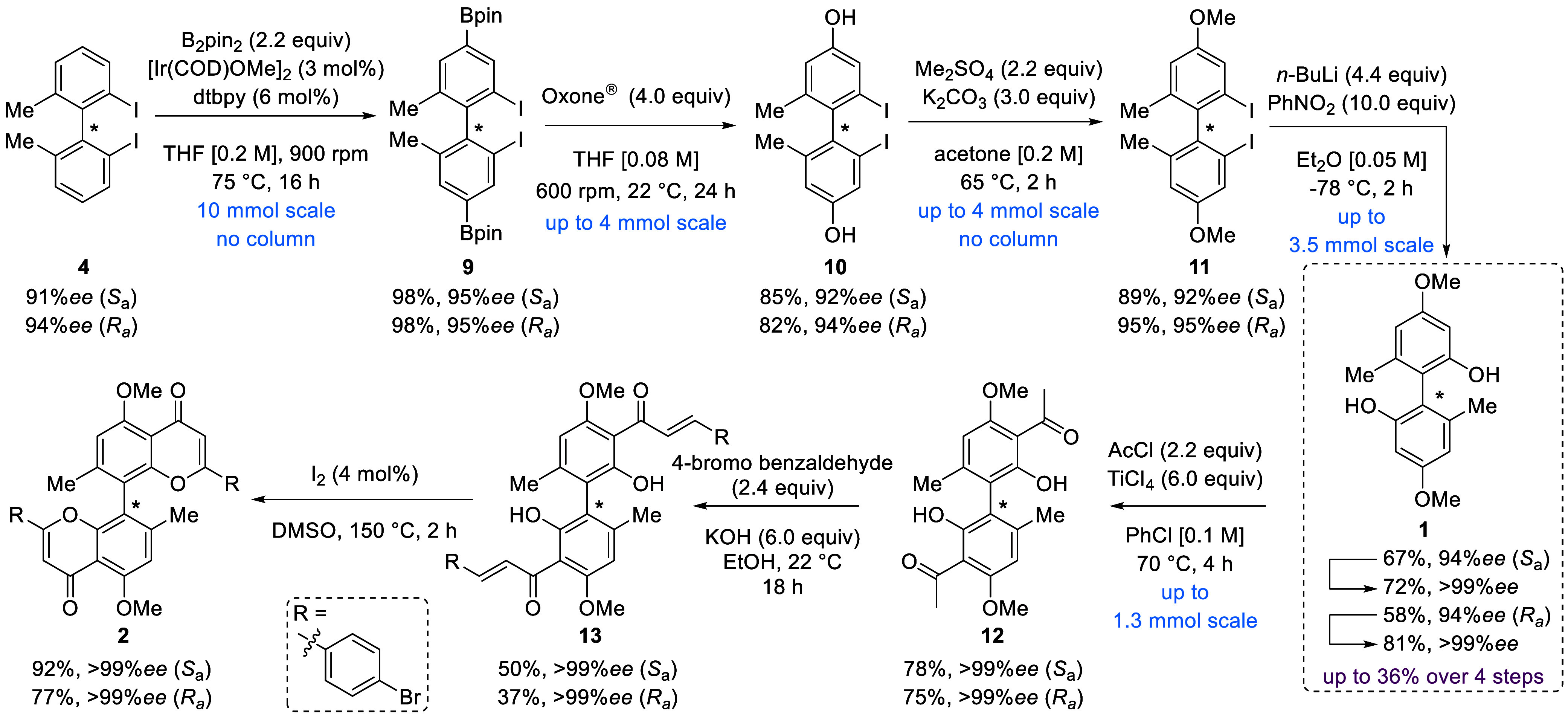
Synthesis of Biphenol **1** and Its Application in the Synthesis of Both Enantiomers of 8,8″-Biflavone **2**

With a robust method for the synthesis of biphenol **1** established, we focused our efforts on the synthesis of biflavone **2**. Following our previously reported protocol,^21^ the Friedel–Crafts acetylation was conducted, and both enantiomers were isolated in yields of 75–78% (Scheme 3). Both enantiomers of enantiopure bichalcone **13** (37–50%) were subsequently isolated. The low yield of this step can be attributed to column chromatographic isolation caused by the additional formation of flavanone side products. Finally, biflavone **2** was isolated in yields of 77–92%. The high enantiomeric excess >99% ee was retained.

With brominated biflavone **2** in hand, we investigated Buchwald–Hartwig amination. After a short round of screening (Table S6), we established a protocol especially suitable for secondary amines using RuPhos.^32^ Amines chosen included commercially available cyclic amines, as well as noncyclic alkylamines. Additionally, the hydrochloride of dimethylamine was accepted. Yields of 75–94% were obtained overall for all six examples of secondary amines **14**–**19** (Scheme 4A). Primary bulky benzylamine could also be coupled but gave the corresponding product **20** in a yield of 34%. We attribute the low yield to the potential double amination of the forming secondary amine and, thus, to oligomerization. When using the hydrochloride of methylamine, we opted for the use of BrettPhos as the ligand of choice. Still, conversion of starting material was incomplete, and the desired product **21** could only be isolated in a yield of 8%. Additionally non-C_2_-symmetrical biflavone **22** was obtained as a side product of biflavone **21** in a yield of 7% that turned out to contain a methoxy/methylamine substitution pattern. Overall, we were able to synthesize a library of nine 8,8″-biflavones with conditions especially suited for secondary amines. Hydrochlorides were accepted, as well. The first non-C_2_-symmetrical 8,8″-biflavone **22** could be isolated.

**Scheme 4:**
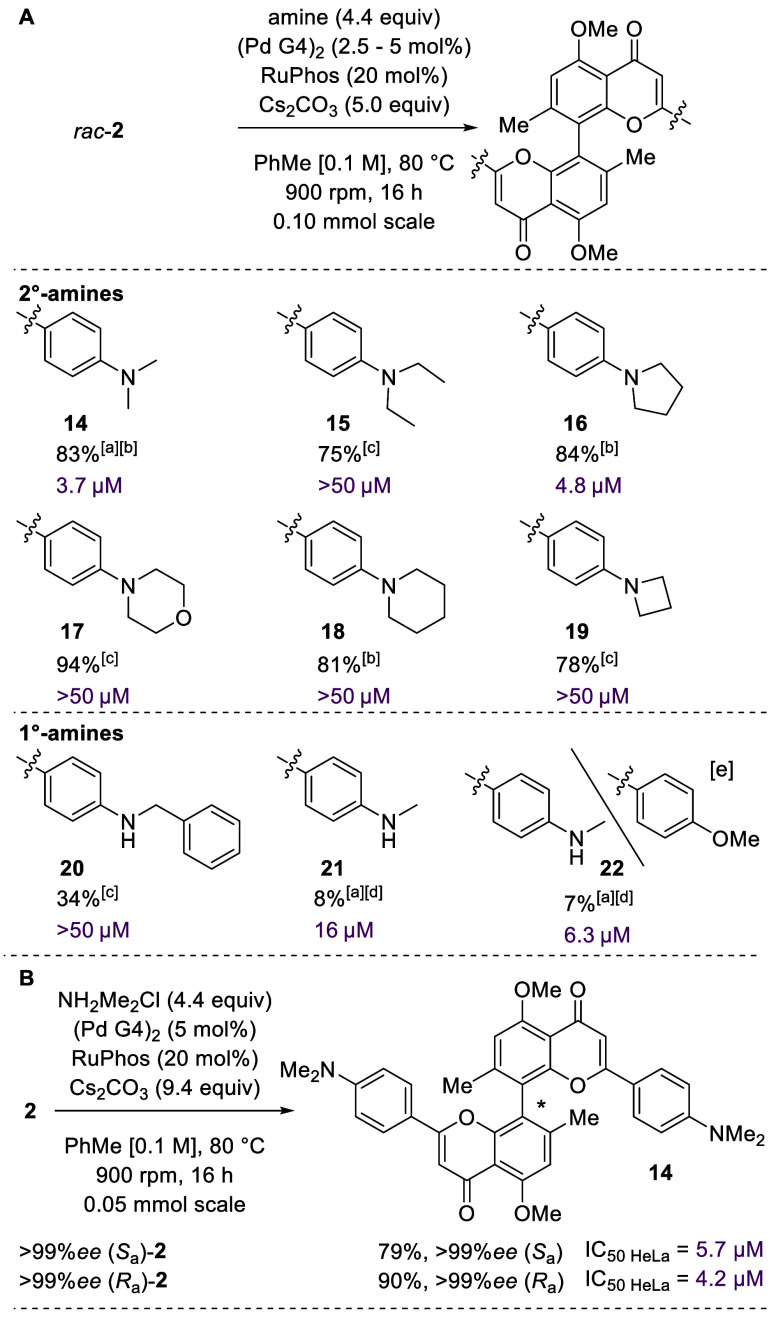
Synthesis of a Library of 8,8″-Amino Biflavones by Palladium-Catalyzed Buchwald–Hartwig Amination Hydrochloride salt of amide used, 9.4 equiv of Cs_2_CO_3_. Reacted with 2.5 mol % (Pd G4)_2_. Reacted with 5.0 mol % (Pd G4)_2_. Reacted with 5.0 mol % (Pd G4)_2_ and BrettPhos (20 mol %). Non-C_2_-symmetrical methylamine/methoxy biflavone, side product using NH_3_MeCl. Yields of isolated products. IC_50_ values against HeLa cells (purple).

Next, the cytotoxicity of the obtained biflavones was tested against malignant human cell lines (HeLa cells). An Alamar Blue assay was chosen as the results gave tight confidence intervals. A comparison of MTT assay and Alamar Blue assay revealed that the IC_50 HeLa_ values were similar to our previously reported values.^11^ After ruling out palladium as a bioactive contaminant^33^ and assessing the bioactivity of the dedicated library, biflavone **14** (3.7 μM) was identified as the most bioactive compound (Scheme 4A). Additionally, minor autophagy modulating properties could be observed for some of the most active compounds (Figure S36). With these results in hand, we continued with the synthesis of both enantiomers of biflavone **14** (Scheme 4B). The Buchwald–Hartwig amination again proceeded smoothly to provide us with both enantiomers in yields of 79% (*S*_a_) and 90% (*R*_a_). It was revealed that the IC_50_ of (*R*_a_)-**14** was similar albeit somewhat higher than that of the racemic mixture [5.7 μM (*S*_a_), 4.2 μM (*R*_a_), and 3.7 μM (*rac*)].

In summary, we were able to show the first application of a cyclic diaryliodonium salt in a natural-product-inspired synthesis. Both enantiomers of biphenol **1** were successfully synthesized in high enantiomeric excess. We showed that the 1,3,5-substitution pattern common among natural products can easily be accessed by our protocols. Additionally, biaryl **9** can serve as a synthetically useful intermediate. We applied this established synthesis route toward the identification of the active enantiomer against malignant human cell lines. Using our previous best hit as a lead structure, we used palladium-catalyzed Buchwald–Hartwig transformations to synthesize a library of 8,8″-biflavones. We evaluated the bioactivity of these racemic biflavones by probing the structure–activity relationship. We then synthesized both enantiomers of the most active compound using our established method and assessed the activity against HeLa cells.

## Data Availability

The data underlying this study are available in the published article and its Supporting Information.

## Supporting Information Available

Additional synthesis procedures; computational details, including all coordinates; coordinates of X-ray structures of compounds **9** and (*R*_a_)-**13**; bioactivity data; biological activity graphs; copies of all ^1^H and ^13^C NMR spectra; and HPLC chromatograms (PDF)

## Dedication

This work is dedicated to Prof. Dr. Thomas J. J. Müller on the occasion of his 60th birthday.
