# Supplementary material for: Application of Cyclic Diaryliodonium Salts in the Synthesis of Axially Chiral Natural Product Analogues
Source: Org Lett. 2024 Jun 17;26(25):5258–62. doi: 10.1021/acs.orglett.4c01308 (PMC11217949; doi:10.1021/acs.orglett.4c01308)
Supplement: Supplementary file 1 — ol4c01308_si_001.pdf [file ol4c01308_si_001.pdf]

# Application of cyclic diaryliodonium salts in the synthesis of axially chiral natural product analogues

Moritz K. T. Klischan[a], Céline David[b], Daniel Grudzinski[a], Wolfgang Frey[c], Björn Stork[b], Jörg Pietruszka[a,d]*

[a]Institute of Bioorganic Chemistry, Heinrich Heine University Düsseldorf, Forschungszentrum Jülich, Stetternicher Forst, Geb.15.8, 52426 Jülich (Germany), E-Mail: j.pietruszka@fz-juelich.de

[b]Institute of Molecular Medicine I, Medical Faculty and University Hospital Düsseldorf, Heinrich Heine University Düsseldorf, Universitätsstr. 1, 40225 Düsseldorf (Germany), E-Mail: bjoern-stork@uni-duesseldorf.de

[c]Institute of Organic Chemistry, University of Stuttgart, 70569 Stuttgart (Germany)

[d]Institut für Bio- und Geowissenschaften (IBG-1: Bioorganische Chemie) Forschungszentrum, 52428 Jülich (Germany) j.pietruszka@fz-juelich.de

## Table of contents


1 Experimental synthesis procedures ..... S3

1.1 General information ..... S3

1.2 Screening reactions general considerations ..... S5

1.2.1 Iodonium Ring Opening ..... S7

1.2.2 Borylation ..... S8

1.2.3 Oxidation Boronic acid ester ..... S9

1.2.4 Dehalo-Oxygenation ..... S10

1.2.5 Deuteration Experiments ..... S11

1.2.6 Flavone Buchwald Hartwig Amination ..... S11

1.2.7 Biflavone Buchwald Hartwig Amination ..... S12

1.2.8 Biphenol Synthesis ..... S14

1.2.9 Miscellaneous Syntheses ..... S30

General Procedure 1 Buchwald Hartwig Amination (GP1) ..... S37

1.3 Pd-content ..... S47

1.4 Chiral HPLC Chromatograms ..... S49

1.5 Reversed Phase HPLC ..... S65

1.5.1 HPLC Chromatograms ..... S67

2 Biological Data ..... S77

2.1 Tables ..... S78

2.2 Western Blots ..... S80

2.3 Materials and Methods ..... S80

3 NMR Spectra ..... S83

3.1.1 Biphenol ..... S83

3.1.2 Biflavones ..... S122

Miscellaneous ..... S149

4 X-Ray ..... S165

5 References ..... S169

# 1 Experimental synthesis procedures

## 1.1 General information

All chemicals not synthesized or present in the group were purchased from *Sigma-Aldrich Co.*, *Alfa Aesar GmbH & Co. KG*, *Merck KGaA* or *Fluorochem Ltd.* All reactants were used without any further purification unless stated otherwise. Methanol was dried using activated molecular sieve (3 Å). DMSO was degassed *via* freeze-pump-thaw. Anhyd. diethylether and dichloromethane were taken from the solvent purifier *MB SPS-800* by *MBraun*. Silica gel 60 (0.040 – 0.063 mm, 230 – 400 mesh) by *Machery Nagel* used for synthesis was dried in an oven at 110 °C over night. Anhyd. solid reagents were stored in a desiccator under an atmosphere of $N_2$.

**Working under inert conditions**

All glassware and stirring bars used for reactions under anhyd. or inert conditions were put in an oven at 110 °C for at least 12 h. When removing glassware from the oven it was sealed airtight using septa and stopcocks. It was then attached to a Schlenk-line and left to cool under nitrogen-gas, which was itself dried over *SICAPENT®*, for several minutes. Glassware was then dried using Schlenk-technique by heating the glassware under vacuum for several minutes and then letting it cool to room temperature under $N_2$-flow. This process was repeated three times. Septa were only opened briefly during the addition of reactants under $N_2$-countercurrent. Liquid reactants, solvents and solutions of reactants were transferred using syringes flushed three times with $N_2$.

**Laboratory devices**

Solvents were removed using rotary evaporators at a bath temperature of 40 °C under reduced pressure. Analytic balance AE 163 by Mettler Toledo was used to determine and weigh yields and reactants. Sonication of reactions was conducted using ultrasonic cleaning bath T310 by Elma Schmidbauer GmbH. Distillations of liquid aldehydes were conducted using Kugelrohrofen *Glass Oven B-580* and *Glass Oven B-585* by *Büchi* under reduced pressure.

**Chromatography**

For thin layer chromatography (TLC) silica gel plates (*Polygram® SIL G/UV 254*) by *Machery-Nagel* with fluorescent indicator. Spots were made visible under UV light,

using aqueous potassium permanganate solution, CAM, Molydip, anisaldehyde. Column chromatographic purification was conducted using the appropriate solvent mixture and silica gel 60 (0.040 – 0.063 mm, 230 – 400 mesh) by *Machery Nagel* in cylindric glass columns by applying pressure with compressed air.

**Analytical devices**

$^{1}$H-, $^{13}$C-, DEPT-135-, COSY-, HSQC-, HMBC-NMR spectra were measured on the spectrometer *Bruker Avance/DRX 600* at a frequency of 600 MHz ($^{1}$H) and 151 MHz ($^{13}$C). $^{19}$F-, $^{11}$B- and select $^{1}$H- and $^{13}$C-NMR spectra were measured on the spectrometer *Bruker Avance/DRX 300* at a frequency of 282 MHz ($^{19}$F), 96 MHz ($^{11}$B), 300 MHz ($^{1}$H) and 75 MHz ($^{13}$C). Deuterated chloroform with or without 0.03 vol% TMS ($CDCl_3$) or deuterated d$^{6}$-DMSO were used as solvent. The $^{1}$H- and $^{13}$C-spectra were referenced to the solvent peak ($CDCl_3$ δ = 7.26 ppm ($^{1}$H), δ = 77.16 ppm ($^{13}$C)). Data were evaluated using the software *MNova* (*MestReNova*) version 14.1.2 by *Mestrelab Research*. Coupling constants *J* were given in Hz and chemical shifts δ in ppm (parts per million). Multiplicities are abbreviated as the following: singlet (s), broad singlet (brs), doublet (d), triplet (t), quartet (q), multiplet (m). 2D-NMR methods including, COSY-, HSQC-, HMBC- and ROESY NMR were used to assign detected $^{1}$H- and $^{13}$C-NMR peaks to the corresponding atoms in the characterized molecules.

High resolution mass spectrometry (HRMS) was measured at the Heinrich Heine University Düsseldorf (Applied Biosystems/ MDS SCIEXQ Model Trap 4000) with electron spray ionization (ESI). Low resolution mass spectrometry was conducted using the express**ion** CMS system by Advion, Inc. in combination with an atmospheric pressure chemical ionization (APCI) or electron spray ionization (ESI). Gas chromatographic mass spectrometry (GC-MS) was conducted using the Thermo Scientific TRACE 1310 gas chromatograph (ISQ QD Single Quadrupole Mass Spectrometer, Helium). Elemental analyses were measured at the Heinrich Heine University Düsseldorf (Elementar, Vario Micro Cube). Furthermore, melting points (Stuart Scientific, Melting Point Apparatus SMP3), HPLC chromatograms (Thermo Scientific, Dionex UltiMate 3000 Column Comportment) and optical rotation (A.Krüss, P8000-TF) were measured with the appropriate devices. IR spectra were recorded using a *SpectrumTwo FT-IR* by *PerkinElmer* with attenuated total reflection (ATR). The absorption bands were given in units of wave numbers ($cm^{-1}$).

**X-ray**

X-ray crystallographic data were measured by Wolfgang Frey at the Department for Single Crystal Diffractometry of the Institute of Organic Chemistry at the University of Stuttgart. Data was collected using a Bruker Kappa APEXII Duo diffractometer. The indicated deposition numbers (CCDC #) contain the supplementary crystallographic data for this paper. These data are provided free of charge by the joint Cambridge Crystallographic Data Centre and Fachinformationszentrum Karlsruhe Access Structures service.

**Chemicals**

*n*-BuLi in hexanes was titrated using diphenylacetic acid in triplicates. Commercial nitrobenzene was washed with aq. NaOH solution (2 M), dist. water, HCl (1 M), dist. water and sat. aq. NaCl solution and dried over $MgSO_4$. Then vacuum distillation was performed at 90 °C, $8{\cdot}10^{-1}$mbar (head temperature 67 °C). After stable temperature the liquid was collected into a round bottom flask with $CaH_2$. The nitrobenzene distilled this way was stored under argon and sealed airtight. DMSO was degassed by freeze pump thaw method. Morpholine was distilled prior to use at 50 °C and $1.9{\cdot}10^{-1}$ mbar. Benzylamine was distilled prior to use at 170 °C and $2.3{\cdot}10^{-1}$ mbar.

## 1.2 Screening reactions general considerations

**Working with transition metal catalyst**

Stir bars used in transition metal catalyzed reactions were cleaned of trace metal impurities by stirring in a bath of conc. aqua regia. Reactions were performed in 2 dram (2 dr.) vials (ThermoScientific National B7999-3) equipped with a PTFE lined cap (ThermoScientific National B7995-15) and a stir bar (Fisher cat no. 14-513-57, 12 x 4.5 mm). Hot vials equipped with stir bars (110 °C for 24 h) were taken out of the oven and immediately capped with a rubber stopper (Saint-Gobain Natural Rubber Folding Skirt Stoppers cat. no. 407010-50) and equipped with Argon filled balloons with an additional needle as an outlet. An oil bath equipped with a stir bar was used as the heating source for such reactions. After adding all non-volatile reagents, the outlet needle was removed. After adding all the reagents, the screw thread of the vial was lined with PTFE tape (in the same direction as the threading). The reactions were then sealed by removing the septum still equipped with the balloon swiftly and screwing on the PTFE lined cap, screwing on the lid tightly. The vial was the sealed with PTFE tape.

Reagents were weighed without gloves to avoid electrostatic discharging while using the analytical balances.

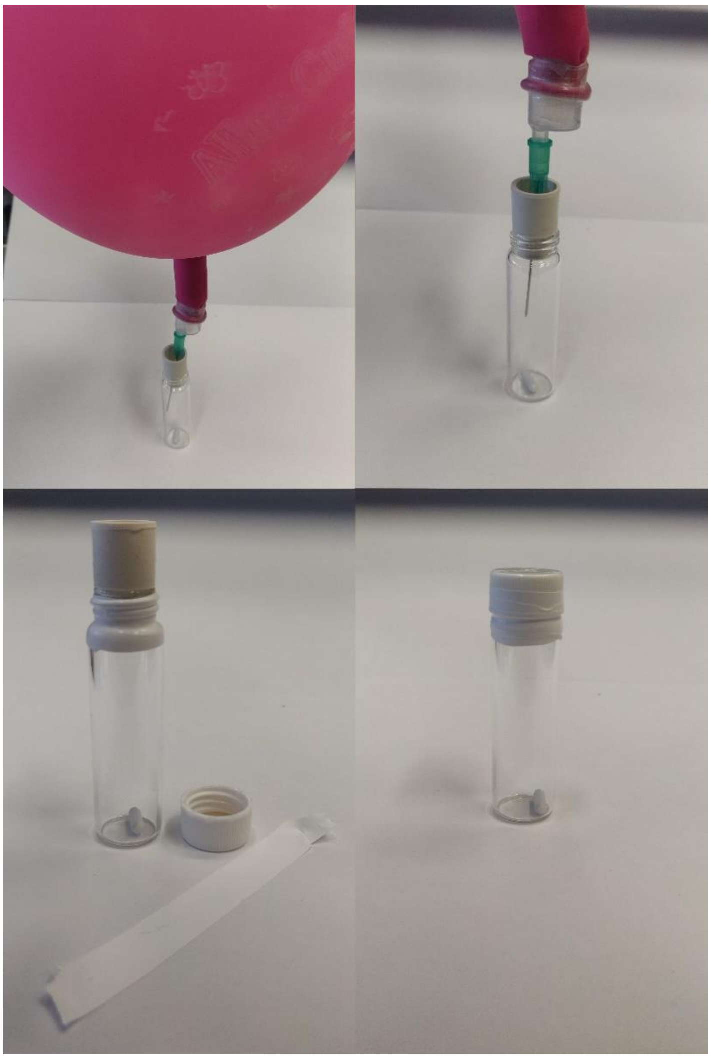

Figure S1: Setup using 2 dram vials. Top left: vial capped with argon balloon. Top right: Zoom in to show stirring bar. Bottom left: threading lined with PTFE-tape (clockwise). Bottom right: septum removed and replaced with PTFE-lined rubber cap, sealed with PTFE-tape (clockwise).

Screening reaction

### 1.2.1 Iodonium Ring Opening

The enantioselective ring opening of stereodynamic iodonium salts has been established by Zhu *et al.*[1] and Ke *et al.*[2] We investigated the viability of their approaches for our scale up and ultimately decided on the protocol by Zhu *et al.* to avoid the use of HFIP due to concerns regarding polyfluorinated compounds (Table S1).[3] We investigated the background ring opening rate in the absence of both ligand (entry 1) and Cu and ligand (entry 2).

Table S1: Enantioselective DYKAT of cyclic biaryl iodonium salt **8**.

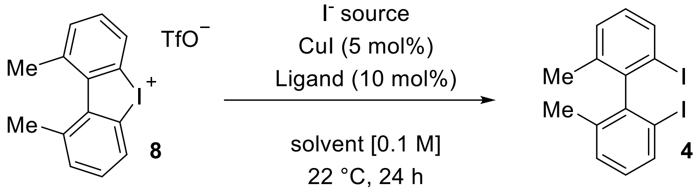


| Entry | Cu [mol%] | Scale [mmol] | Method | Ligand [mol%] | Product [%][a] | ee [%][c] |
|---|---|---|---|---|---|---|
| 1 | 5 | 0.10 | **A** | (*S*,*S*)-**L1** | (100) | 92 |
| 2 | 5 | 0.10 | **B** | (*S*,*S*)-**L1** | (73) | 88 |
| 3 | 5 | 1.50 | **A** | (*S*,*S*)-**L1** | 96 | 94 |
| 4[b] | 5 | 1.50 | **B** | (*S*,*S*)-**L1** | 82 | 84 |
| 5[b] | 5 | 12.0 | **B** | (*S*,*S*)-**L1** | 94 | 91 |
| 6 | 5 | 18.0 | **B** | (*S*,*S*)-**L1** | 86 | 91 |
| 7 | 5 | 18.0 | **B** | (*R*,*R*)-**L1** | 82 | −94 |

Method A: (*S,S*)-**L1** 10 mol%, TBAI (1.0 equiv), HFIP. Method B: (*S,S*)-**L1** 7.5 mol%, NaI (1.2 equiv), $CH_2Cl_2$. [a] Isolated yield; conversion to product according to $^1$H-NMR in parentheses using 1,3,5-trimethoxybenzene as an internal standard. [b] (*S,S*)-**L1** 10 mol%. [c] enantiomers not base line separated by HPLC, thus *ee* reported may vary.

### 1.2.2 Borylation

*Meta*-selective borylations were first established by Ishiyama *et al.* and their protocol used as the basis for our investigations.[4] It is worth mentioning, that Deng *et al.* independently investigated the double borylation on biarylic systems.[5] Separation of the mono-borylated side product proved not possible thus full conversion was of major importance for full characterization of biaryl **9**. The use of slight excess of $B_2pin_2$ in addition to the use of 3 mol% Iridium catalyst was crucial for full conversion and a high yield.

Table S2: Double *meta*-selective borylation.

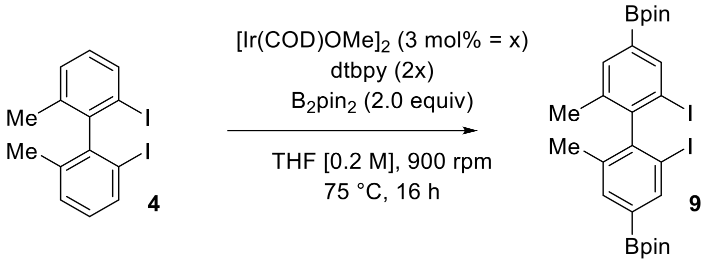


| Entry | Variations | Product [%][a] |
|---|---|---|
| 1 | None | (89±3) |
| 2 | $[Ir(COD)OMe]_2$ (0.38 mol%) | (1±1) |
| 3 | $[Ir(COD)OMe]_2$ (0.75 mol%) | (0±0) |
| 4 | $[Ir(COD)OMe]_2$ (1.5 mol%) | (7) |
| 5 | 1 h reaction time | (78±0) (28±5)[b] |
| 6 | Anhydr $B_2pin_2$ | (90) |
| 7 | $B_2pin_2$ (2.2 equiv) | (99) |
| 8 | 10.0 mmol scale, $B_2pin_2$ (2.2 equiv) | 98±0[c] |

Reactions were performed on 0.10 mmol scale. Entries with standard deviation performed as duplicates. [a] Conversion to product according to $^1$H-NMR in parentheses using 1,3,5-trimethoxybenzene as an internal standard. [b] mono borylated product. [c] Isolated yield.

### 1.2.3 Oxidation Boronic acid ester

We based our investigations on a protocol established by Maleczka *et al.*[6]

Table S3: Double oxidation of aryl boronic acid esters.

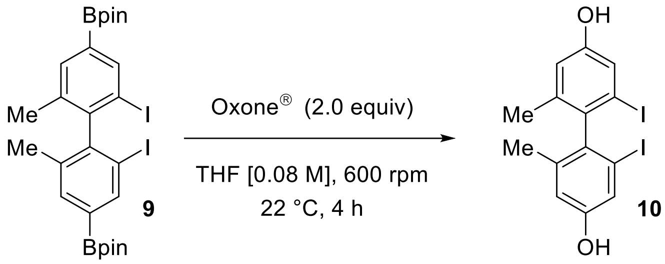


| Entry | Variations | $(ArOH)_2$ [%][a] | ArBpin-ArOH [%][a] | $(ArBpin)_2$ [%][a] |
|---|---|---|---|---|
| 1 | None | 68 | 24 | 3 |
| 2 | Acetone | 22 | 0 | 73 |
| 3 | 24 h | 75 | 15 | 4 |
| 4 | 24 h, Oxone® (4.0 equiv) | 77 | 5 | 0 |
| 5 | 24 h, Oxone® (4.0 equiv), 0.40 mmol scale | **85**[b] | - | - |
| 6 | 24 h, Oxone® (4.0 equiv), 4.00 mmol scale | **83±3**[b] | - | - |

Reactions performed on a 0.05 mmol scale. [a] Conversion to product according to $^1$H-NMR using 1,3,5-trimethoxybenzene as an internal standard. [b] isolated yield.

### 1.2.4 Dehalo-Oxygenation

We explored various methods of transforming the 2,2′-diiodo biaryl into the desired 2,2′-biphenol (Table S4). The use of alternative oxidants and transition metal catalysis proved unsuccessful so we ultimately settled on the established protocol by Ke *et al.*[2]

Table S4: Dehalo-oxygenation via metal halogen exchange.

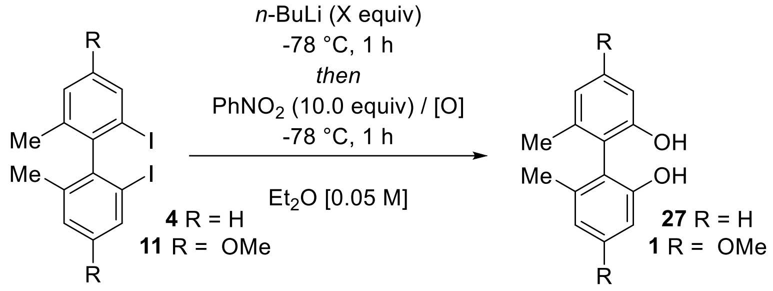


| Entry | Variations | Products[a] | | | |
|---|---|---|---|---|---|
| R = H | *n*-BuLi (2.2 equiv) | $(ArOH)_2$ [%] | $(ArH)_2$ [%] | ArH-ArOH [%] | $(ArI)_2$ [%] |
| 1 | None | 44 | 29 | 24 | 0 |
| 2 | [O] = Oxygen dried over acetone/dry ice trap | | | | |
| 3 | [O] = Oxygen balloon | 11 | 27 | 24 | 7 |
| 4 | Conditions: CuI (20 mol%), dibenzoylmethane (1.0 equiv), KOH (6.0 equiv), DMSO:$H_2O$ (1:1) [0.25 M][7] | 2 | 0 | 0 | 95 |
| 5 | [O] = Oxygen balloon dried over Sicapent® | 20 | 33 | 27 | 0 |
| 6 | [O] = Oxygen balloon dried over Sicapent® Equip balloon, stir for 16 h at −78 °C | 17 | 23 | 31 | 0 |
| 7 | [O] = Oxygen balloon dried over Sicapent® Equip balloon, stir for 1 h at 22 °C | 17 | 22 | 30 | 0 |
| 8 | [O] = Oxygen balloon dried over Sicapent® Equip balloon, stir for 16 h at 22 °C | 15 | 23 | 30 | 0 |
| 9 | [O] = Oxygen balloon dried over $CaCl_2$ | 22 | 15 | 33 | 0 |
| 10 | [O] = Oxygen balloon dried over Sicapent® Quench with $D_2O$ after 1 h $O_2$ at 22 °C | 16 | 30[c] | 28 | 0 |
| 11 | *n*-BuLi (4.4 equiv), [O] = Oxygen balloon dried over Sicapent® | 28 | 15 | 35 | 0 |
| 12 | *t*-BuLi (4.4 equiv), [O] = Oxygen balloon dried over Sicapent® | 16 | 14 | 23 | 17 |
| 13 | *n*-BuLi (4.4 equiv) | 54 (**44**)[b] | 27 | 11 | 0 |
| 14 | *t*-BuLi (4.4 equiv) | 26 | 45 | 0 | 0 |
| R = OMe | *n*-BuLi (4.4 equiv) | | | | |
| 15 | *n*-BuLi (2.2 equiv) | 21 | 16 | 26 | 27 |
| 16 | None | 56±1 (**54**)[b] | 16±1 | 16±1 | 0 |
| 17 | $PhNO_2$ solution in $Et_2O$ at -78 °C | 52 | 14 | 23 | 0 |

| | | | | | |
|---|---|---|---|---|---|
| 18 | $PhNO_2$ solution in $Et_2O$ at 22 °C | 22 | 16 | 26 | 0 |
| 19 | Conditions: $Pd(dba)_2$ (5 mol%), *t*-BuBrettPhos (20 mol%), KOH (6.0 equiv), $H_2O$ (40 equiv), dioxane [0.25 M][8[d]] | 0 | 0 | 6 | 81 |
| 20 | −95 °C[e] | 59 | 8 | 18 | 0 |
| 21 | 0.40 mmol scale | 52 | 7 | 15 | 0 |
| 22 | 1.97 mmol scale | (**55**)[b] | - | - | - |
| 23 | 3.49 mmol scale | (**62**)[b] | - | - | - |

Reactions were performed on 0.10 mmol scale. Entries with standard deviation performed as duplicates. [a] Conversion to product according to $^1$H-NMR using 1,3,5-trimethoxybenzene as an internal standard. [b] isolated yield. [c] 25% deuterated according to $^1$H-NMR. [d] positive control using 4-iodo anisol gave conversion to product [e] methanol:liquid $N_2$ bath, temperature according to thermometer

### 1.2.5 Deuteration Experiments

To show the efficacy of the metal-halogen exchange step we conducted deuteration experiments (Table S5).

Table S5: Deuteration experiments to show the efficacy of the metal-halogen exchange.

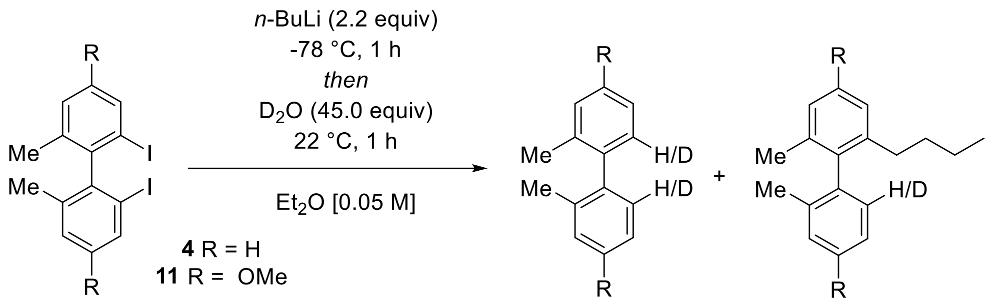


| Entry | Variations | Products[a] | | | |
|---|---|---|---|---|---|
| | R = H | $(ArH/D)_2$ [%] | Deuteration [%] | ArH/D-ArBu [%] | Deuteration [%] |
| 1 | None | 95 | 94 | 0 | - |
| 2 | THF | 5 | - | 90 | 96 |
| 3 | THF, stir at 22 °C for 1 h before $D_2O$ addition | 4 | - | 59[b] | 0 |
| | R = OMe | | | | |
| 4 | *n*-BuLi (4.4 equiv) | 88 | 89 | - | - |

Reactions were performed on a 0.10 mmol scale. [a] Conversion to product according to $^1$H-NMR using 1,3,5-trimethoxybenzene as an internal standard. [b] suspected 41% conversion to $(ArBu)_2$ product.

### 1.2.6 Flavone Buchwald Hartwig Amination

We investigated the Buchwald Hartwig amination starting with readily available monomer flavone **23**.[9]

Key in the established amination protocol was, to make sure not to purge the amine with an argon balloon (Table S6, entry 4). High volatility of the amine resulted in substantial evaporation of the amine and thus incomplete conversion of starting material.

Table S6: Screening of Pd-catalyzed Buchwald Hartwig amination.

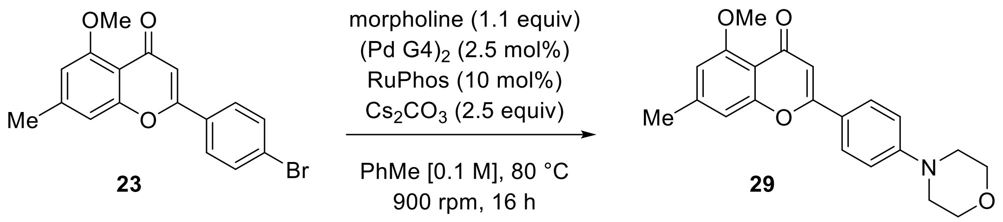


| Entry | Variation | Conversion of 23 [%] | Product [%][a] |
|---|---|---|---|
| 1 | None | 0 | 0 |
| 2 | BINAP (10 mol%) | 5 | 0 |
| 3 | Morpholine (2.2 equiv) | 42±2 | 6±1 |
| 4 | Don't purge Morpholine, Morpholine (2.2 equiv) | 72 | 59 |
| 5 | Don't purge Morpholine, Morpholine (2.2 equiv), RuPhos (20 mol%) | 100 | 89±5 |
| 6 | Don't purge Morpholine, Morpholine (2.2 equiv), RuPhos (40 mol%) | 100 | 94±1 (89)[b] |

Reactions were performed on a 0.10 mmol scale. Entries with standard deviation performed as duplicates. [a] Conversion to product according to $^1$H-NMR in parentheses using 1,3,5-trimethoxybenzene as an internal standard. [b] Isolated yield.

Additionally, the use of benzylamine as a primary amine was investigated. The use of 2.2 equiv of benzylamine was key in full conversion of starting material.

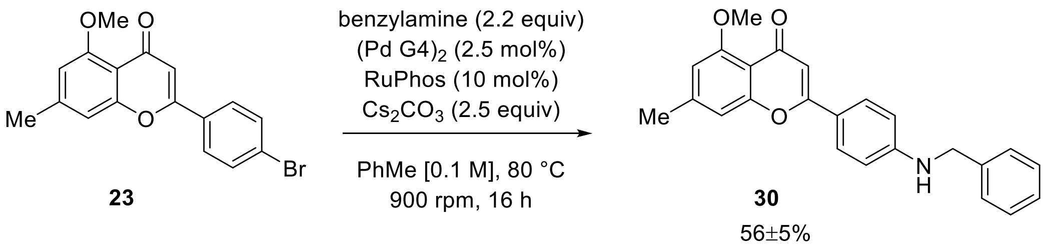


Scheme S1: Synthesis of amino flavone **30**. Reaction performed as duplicate. Full conversion of **23**.

### 1.2.7 Biflavone Buchwald Hartwig Amination

Morpholine and benzylamine were distilled prior to use. Pd-loading of 2.5 mol% led to incomplete conversions in some cases, increasing the Palladium loading to 5 mol% corresponding to the Pd-loading for the flavone monomers, full conversion to product was observed. Using hydrochlorides of amines required additional equivalents of base.

Table S7: Buchwald Hartwig amination to obtain amino 8,8′-biflavones.

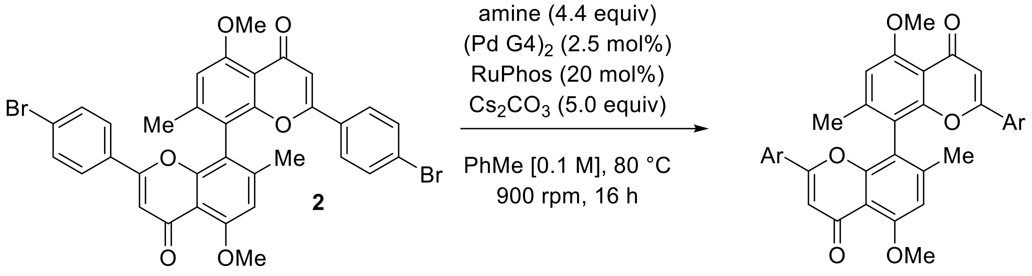


| Entry | Variation | amine | Product | Product [%][a] |
|---|---|---|---|---|
| 1 | $Cs_2CO_3$ (9.4 equiv) | $NHMe_2\cdot HCl$ | **14** | 83 (92) |
| 2 | $(Pd\ G4)_2$ (5.0 mol%) | $NHEt_2$ | **15** | 75 (81) |
| 3 | None | Pyrrolidine | **16** | 84 (91) |
| 4 | None | Morpholine | **17** | 94 (97) |
| 5 | $(Pd\ G4)_2$ (5.0 mol%) | Piperidine | **18** | 81 (89) |
| 6 | $(Pd\ G4)_2$ (5.0 mol%) | Azetidine | **19** | 78 (81) |
| 7 | $(Pd\ G4)_2$ (5.0 mol%) | Benzylamine | **20** | 34 (59) |
| 8 | $(Pd\ G4)_2$ (5.0 mol%), BrettPhos (20 mol%), $Cs_2CO_3$ (9.4 equiv) | $NH_2Me\cdot HCl$ | **21** | 8[b] |

Reactions were performed on a 0.10 mmol scale. [a] Isolated yield, conversion to product according to $^1$H-NMR in parentheses using 1,3,5-trimethoxybenzene as an internal standard. [b] Incomplete conversion thus impossible to determine conversion to product according to $^1$H-NMR.

## 1.2.8 Biphenol Synthesis

### 1.2.8.1 2',6-Dimethyl-[1,1'-biphenyl]-2-amine (5)

A 100 mL dry Schlenk round-bottom flask equipped with stir bar was charged with 2-bromo-3-methylaniline (625 µL, 1.00 equiv, 5.00 mmol) and 2-methylphenylboronic acid pinacol ester (1.80 g, 1.20 equiv, 7.50 mmol), SPhos Pd G4 (99.3 mg, 125 µmol, 2.5 mol%). and SPhos (51.3 mg, 50.0 µmol, 2.5 mol%) and capped with a rubber septum and anhydr. THF (25.0 mL, 0.21 M, degassed by sparging for 20 min) was added. In a separate flask $K_3PO_4$ (3.19 g, 3.00 equiv, 15.0 mmol) was dried at 80 °C overnight in vacuo and then dissolved in degassed water (2.5 M, degassed by sparging for 20 min) and then added to the reaction vessel at once. The reaction was then heated to 65 °C and stirred for 16 h. After completion, the reaction mixture was filtered over a pad of Celite® washing with EtOAc and the solvents removed in vacuo. The product was isolated by column chromatography (petroleum ether:EtOAc 95:5 v/v) as an amber oil in a yield of 933 mg (4.73 mmol, 95%). The analytical data were in accordance with literature. [10]

**$^{1}$H NMR** (600 MHz, $CDCl_3$): δ 1.92 (s, 3H, Me), 2.08 (s, 3H, Me′), 3.34 (s, 2H, $NH_2$), 6.63 (d, $^{3}J_{3\text{-}4}$ = 7.9 Hz, 1H, 3-H), 6.70 (d, $^{3}J_{5\text{-}4}$ = 7.5 Hz, 1H, 5-H), 7.07 (dd, $^{3}J_{4\text{-}3,4\text{-}5}$ = 7.7 Hz, 1H, 4-H), 7.10 – 7.15 (m, 1H, 6′-H), 7.25 – 7.31 (m, 2H, 4′-H + 5′-H), 7.30 – 7.35 (m, 1H, 3′-H).

**$^{13}$C NMR** (151 MHz, $CDCl_3$): δ 19.40 (Me′), 20.32 (Me), 112.67 (C-3), 119.97 (C-5), 126.73 (C-5′), 127.21 (C-1), 127.80 (C-4′), 127.99 (C-4), 130.01 (C-6′), 130.58 (C-3′), 136.97 (C-6), 137.23 (C-2′), 137.61 (C-1′), 143.87 (C-2).

**IR (ATR film) [cm$^{-1}$]:** 3468, 3378, 3060, 2921, 1668, 1610, 1464, 1303, 1004, 765.

**TLC** (petroleum ether:EtOAc 95:5 v/v): $R_f$ = 0.23; (penH:EtOAc 8:2 v/v) $R_f$ = 0.37

**HR-MS (ESI):** m/z calculated for $[C_{14}H_{16}N]^+$ ([M + H$^+$]): 198.1277, found: 198.1280.

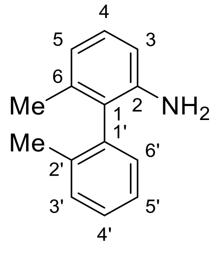


**5**

### 1.2.8.2 2',6-dimethyl-[1,1'-biphenyl]-2-amine hydrochloride (6)

A 500 mL dry Schlenk round-bottom flask equipped with stir bar was charged with 2-bromo-3-methylaniline (6.16 mL, 1.00 equiv, 50.0 mmol), a o-tolylboronic acid (8.16 g, 1.20 equiv, 60.0 mmol), SPhos Pd G4 (397 mg, 1 mol%, 0.500 mmol) and SPhos (205 mg, 1 mol%, 0.500 mmol) and capped with a rubber septum and anhydr. THF (250 mL, 0.21 M, degassed by sparging for 20 min) was added. In a separate flask $K_3PO_4$ (31.9 g, 3.00 equiv, 150 mmol) was dried at 80 °C overnight in vacuo and then dissolved in degassed water (2.5 M, degassed by sparging for 20 min) and then added to the reaction vessel at once. The reaction was then heated to 65 °C and stirred for 16 h. After completion, the reaction mixture was filtered over a pad of Celite® washing with EtOAc and the solvents removed in vacuo. The crude product was dissolved in pentane. Aq. HCl-solution (1 M), was added and the two phase mixture stirred vigorously forming off-white solids immediately. The solids were filtered off, washed with copious *n*-pentane, and dried in a desiccator over night. The product was obtained as off-white solids without further purification in a yield of 10.2 g (43.8 mmol, 91%).

**$^1$H NMR** (600 MHz, $CDCl_3$): δ 9.60 (s, 3H, $NH_3^+$), 7.43 (dd, *J* = 5.5, 3.7 Hz, 1H, Ar-H), 7.30 (d, *J* = 1.6 Hz, 1H, Ar-H), 7.26 (dd, *J* = 7.6, 1.9 Hz, 1H, Ar-H), 7.08 – 7.15 (m, 3H, Ar-H), 2.02 (s, 3H, $CH_3$), 1.99 (s, 3H, $CH_3$).

**$^{13}$C NMR** (151 MHz, $CDCl_3$): δ 138.4, 137.1, 136.1, 133.9, 130.9, 130.3, 130.1, 128.8, 128.8, 128.3, 126.6, 122.0, 20.0 ($CH_3$), 19.9 ($CH_3$).

**IR (ATR film) [$cm^{-1}$]:** 2850, 2596, 1584, 1528, 1463, 778, 763, 743, 730.

**HR-MS (ESI):** m/z calculated for $[C_{14}H_{16}N]^+$ ([M - $Cl^-$]): 198.1277, found: 198.1280.

**Melting point:** 173 – 180 °C

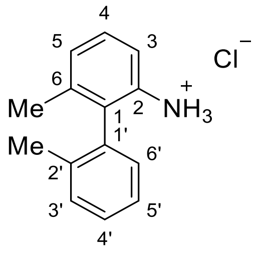


**6**

#### 1.2.8.3 2-iodo-2',6-dimethyl-1,1'-biphenyl (7)

A 500 mL round bottom flask equipped with stir bar was charged with 2-amino biaryl **6** (16.4 g, 1.0 equiv, 70.0 mmol) and dissolved in acetone (1.00 M). A solution of aq. HCl (1.7 M, 70.0 mL, 1.7 equiv, 119 mmol) was added and the reaction mixture stirred at 0 °C for 5 min. Aq. $NaNO_2$ (0.86 M, 124 mL, 1.5 equiv, 105 mmol) solution was added. The reaction was stirred at 0 °C for 30 min. EtOAc (1.00 M regarding the amine, 70 mL) was added and a solution of aq. KI (3.00 M, 70 mL, 3.0 equiv, 210 mmol) was added dropwise over 1 h at 0 °C. The reaction was stirred at 22 °C for an additional 1 h. Then aq. sat. $Na_2SO_3$ was added and the mixture extracted with EtOAc (3x), washed with aq. sat. NaCl-solution and dried over $MgSO_4$. Solvents were removed in vacuo and the crude product then suspended in pentane and filtered over a plug of silica washing with pentane (check with TLC for any remaining product stuck to silica). Solvents were removed in vacuo and the product isolated as a colorless oil in a yield of 17.7 g (57.5 mmol, 82%).

**$^1$H NMR** (600 MHz, $CDCl_3$): δ 7.79 (d, $^3J$ = 8.0 Hz, 1H, H-3), 7.26 (m, 3H, H-3', H-4', H-5'), 7.25 (d, $^3J$ = 7.6 Hz, 1H, H-5), 6.99 (dd, $^3J$ = 7.4, 1.4 Hz, 1H, H-6'), 6.96 (t, $^3J$ = 7.8 Hz, 1H, H-4), 2.03 (s, 3H, Me), 2.01 (s, 3H, Me')

**$^{13}$C NMR** (151 MHz, $CDCl_3$): δ 145.9 (C-1), 144.1 (C-1'), 137.8 (C-6), 136.6 (C-3), 135.5 (C-2'), 130.2 (C-3'), 129.8 (C-5), 129.0 (C-6'), 128.9 (C-4'), 128.0 (C-4), 126.2 (C-5'), 101.2 (C-2), 21.9 (Me), 19.6 (Me')

**IR (ATR film) [cm$^{-1}$]:** 3050, 3016, 2971, 2947, 2919, 2858, 1555, 1454, 1439, 1173, 1115, 1008, 828, 758, 741, 727, 638, 461.

**TLC** (petroleum ether:EtOAc 95:5 v/v): $R_f$ = 0.63

**APCI-MS:** m/z: ([M$^+$]): found: 308.1. ([M - I$^-$]): found: 181.2

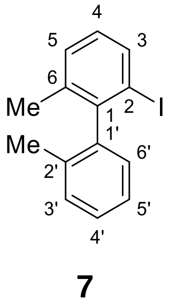


**7**

### 1.2.8.4 1,9-dimethyldibenzo[b,d]iodol-5-ium (8)

A 1000 mL round bottom flask was charged with biaryl **7** (10.5 g, 1.0 equiv, 33.9 mmol), $CH_2Cl_2$ (226 mL, 0.15 M). To the stirred solution was added *m*-CPBA (15.6 g, 2.0 equiv, 67.8 mmol). After full dissolution of the *m*-CPBA, the solution was cooled to 0 °C and TfOH (9.00 mL, 3.0 equiv, 102 mmol) added dropwise via a dropping funnel. The resulting suspension was stirred at room temperature for 1 h. The solvent was removed in vacuo and the resulting crude product suspended in $Et_2O$. The resulting mixture was stirred for 20 min and the solids collected by filtration and washed with additional $Et_2O$. The product was obtained as off-white solids (12.0 g, 26.4 mmol, 78%).

The analytical data were in accordance with literature.[2]

**$^1$H NMR** (600 MHz, $CDCl_3$): δ 8.25 (d, $^3J$ = 8.0 Hz, 2H, H-4), 7.58 (d, $^3J$ = 7.6 Hz, 2H, H-2), 7.53 (t, $^{3J}$ = 7.8 Hz, 2H, H-3), 2.57 (s, 6H, Me).

**$^{13}$C NMR** (151 MHz, $CDCl_3$): δ 141.9 (C-6), 140.5 (C-1), 134.0 (C-2), 130.3 (C-3), 128.9 (C-4), 120.1 (C-5), 24.1 (Me)

**$^{19}$F NMR** (282 MHz, $CDCl_3$): δ -78.02.

**IR (ATR film) [cm$^{-1}$]:** 3485 (broad), 3103, 3081, 1442, 1287, 1239, 1224, 1163, 1025, 777, 696, 636, 574, 516.

**TLC** (petroleum ether:EtOAc 2:8 v/v): $R_f$ = 0.21

**HR-MS (ESI):** m/z calculated for $[C_{14}H_{12}I]^+$ ([M - OTf$^-$]): 306.9979, found: 306.9978.

**Melting point:** 170 – 171 °C

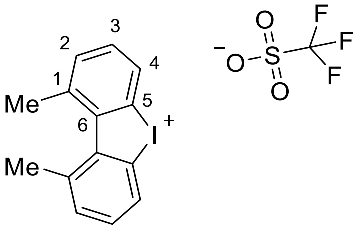


**8**

### 1.2.8.5 2,2'-diiodo-6,6'-dimethyl-1,1'-biphenyl (8)

A dry 500 mL round bottom flask was charged with CuI (171 mg, 5 mol%, 0.900 mmol) and bisoxazoline ligand (*S,S*)-**L1** (452 mg, 7.5 mol%, 1.35 mmol). Anhydr. $CH_2Cl_2$ (180 mL, 0.1 M) was added and the mixture stirred for 10 min. Iodonium salt **8** (8.21 g,

1.0 equiv, 18.0 mmol) was added and once fully dissolved flame dried NaI (3.24 g, 1.2 equiv, 21.6 mmol) was added at once. A septum and Argon balloon were equipped, and the reaction stirred for 24 h at room temperature. The reaction mixture was washed with saturated aqueous $Na_2SO_3$ solution, washed with sat. aq. NaCl solution and dried over $MgSO_4$. Solvent was removed in vacuo and the crude product suspended in *n*-pentane. The crude product was then filtered over a plug of silica washing with copious amounts of *n*-pentane until no more product elution could be detected by TLC. The product was obtained as white crystalline solids (6.70 g, 15.4 mmol, 86%, 91%*ee* $S_a$).

A further experiment on the same scale with ligand (*R*,*R*)-**L1** gave the enantiocomplementary product as white crystalline solids (6.30 g, 18.0 mmol, 82%, 94%*ee* $R_a$).

A further experiment with CuI (14.3 mg, 5 mol%, 7.50 µmol), anhydr. $CH_2Cl_2$ (15 mL, 0.1 M), iodonium salt **8** (684 mg, 1.0 equiv, 1.50 mmol) and TBAI (554 mg, 1.0 equiv, 1.50 mmol) without ligand gave the racemic product as white solids (608 mg, 1.40 mmol, 93%, *rac*).

The analytical data were in accordance with literature.[2]

**$^1$H NMR** (600 MHz, $CDCl_3$): δ 7.81 (d, $^3J$ = 7.9 Hz, 1H, H-3), 7.28 (d, $^3J$ = 7.6 Hz, 1H, H-5), 7.00 (t, $^3J$ = 7.8 Hz, 1H, H-4), 2.01 (s, 3H, Me).

**$^{13}$C NMR** (151 MHz, $CDCl_3$): δ 147.6 (C-1), 137.7 (C-6), 136.9 (C-3), 130.2 (C-5), 129.6 (C-4), 100.8 (C-2), 21.6 (Me).

**IR (ATR film) [cm$^{-1}$]:** 2978, 2934, 2835, 1600, 1575, 1490, 1457, 1432, 1387, 1353, 1315, 1272, 1247, 1145, 1125, 1073, 1046, 1026, 963, 862, 829, 760, 659.

**TLC** (petroleum ether:EtOAc 9:1 v/v): $R_f$ = 0.68

**APCI-MS:** m/z: ([M + H$^+$]): found: 433.8.

**Melting point:** 94 – 96 °C (*rac*)

92 – 93 °C ($S_a$, 92%*ee* HPLC) (93 – 95 °C)[11]

**HPLC:** Chiralpak® IC (Daciel) 250 ° 4.6 mm, 25 °C, 0.5 mL min$^{-1}$, 225 nm, *n*-heptane:*i*-PrOH 99.9:0.1 (v/v) $t_R$($S_a$) = 10.0 min, $t_R$($R_a$) = 11.1 min

**Optical rotation:** $[\alpha]^{25}_{D}$ = +35.0 (±0.1, duplicate) (c = 0.970, $CHCl_3$, *S*a, 92%ee by chiral HPLC)

$[\alpha]^{27.5}_{D}$ = +26.8 (c = 0.970, $CHCl_3$, Sa, 92%ee by chiral HPLC) −21.0° (*R*a)[2]

$[\alpha]^{20}_{D}$ = +28.2 (c = 0.970, $CHCl_3$, Sa, 92%ee by chiral HPLC)

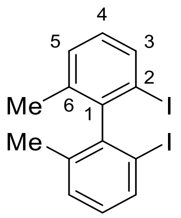


**4**

### 1.2.8.6 2,2'-(2,2'-diiodo-6,6'-dimethyl-[1,1'-biphenyl]-4,4'-diyl)bis(4,4,5,5-tetramethyl-1,3,2-dioxaborolane) (9)

A dry 250 mL Schlenk-vial equipped with a stir bar was charged with biaryl **4** (4.34 g, 1.00 equiv, 10.0 mmol, 91%*ee* *S*a) and $B_2pin_2$ (5.33 g, 2.10 equiv, 21.0 mmol). A separate dry Schlenk-flask equipped with a stir bar was charged with $(Ir(COD)OMe)_2$ (199 mg, 3 mol%, 0.30 mmol), dtbpy (161 mg, 6 mol%, 0.60 mmol), and THF (50 mL). The catalyst mixture was stirred for 5 min and then added to the reaction mixture. The flask was sealed with PTFE band and the reaction mixture stirred at 700 rpm at 75 °C for 16 h. MeOH (20 mL) was added to quench HBpin. The crude mixture was filtered over a plug of silica washing with petroleum ether:EtOAc (9:1, 5000 mL) until no more product was detectable by TLC (product elutes poorly on silica). Solvent was removed *in vacuo* until 500 mL remained. The organic phase was then washed with sat. aq. $NaHCO_3$ (3x 100 mL) to remove pinacol, the org. phase dried over $MgSO_4$ and solvent removed in vacuo. The isolated product was obtained as white solids (6.74 g, 9.83 mmol, 98%, 95%*ee* *S*a).

A further experiment using biaryl **4** (4.34 g, 1.00 equiv, 10.0 mmol, 94%*ee* *R*a) gave the enantiocomplementary product as white solids (6.75 g, 9.84 mmol, 98%, 95%*ee* *R*a).

**$^1$H NMR** (600 MHz, $CDCl_3$): δ 8.23 (s, 1H, H-3), 7.68 (s, 1H, H-5), 1.98 (s, 3H, Me), 1.36 (s, 12H, B(OC<u>*Me*</u>$_2$)$_2$).

$^{13}$**C NMR** (151 MHz, $CDCl_3$): δ 150.2 (C-1), 143.0 (C-3), 136.9 (C-6), 136.3 (C-5), 130.9 (brs, C-4), 100.4 (C-2), 84.3 (B(O<u>C</u>Me$_2$)$_2$), 25.1 (B(OC*<u>Me</u>*$_2$)$_2$), 25.1 (B(OC*<u>Me</u>*$_2$)$_2$), 21.2 (Me).

$^{11}$**B NMR** (96 MHz, $CDCl_3$): δ 31.52.

**IR (ATR film) [cm$^{-1}$]:** 2978, 2928, 1595, 1527, 1423, 1371, 1339, 1314, 1270, 1237, 1213, 1141, 1124, 1006, 966, 909, 888, 852, 801, 732, 697, 684, 648, 579.

**TLC** (petroleum ether:EtOAc 95:5 v/v): $R_f$ = 0.35

**HR-MS (ESI):** m/z calculated for $[C_{26}H_{38}B_2NI_2O_4]^+$ ($[M + NH_4^+]$): 704.1071, found: 704.1078.

**Melting point:** 262 – 268 °C (*rac*)

267 – 270 °C (*S*$_a$, 98%*ee* HPLC)

**HPLC:** Lux® Amylose-1 (Phenomenex) 250 ° 4.6 mm, 10 °C, 0.5 mL min$^{-1}$, 222 nm, *n*-heptane:*i*-PrOH 99:1 (v/v) $t_R$(*S*$_a$) = 6.5 min, $t_R$(*R*$_a$) = 6.8 min

**Optical rotation**: $[\alpha]^{25}_D$ = +20.3 (±0.4, duplicate) (c = 1.00, $CHCl_3$, *S*$_a$, 98%*ee* by chiral HPLC)

**X-ray: 9** was dissolved in a glass vial in little $Et_2O$, layered with *n*-pentane and solvent left to mix at 22 °C over time sealed with a plastic cap. CCDC 2342278

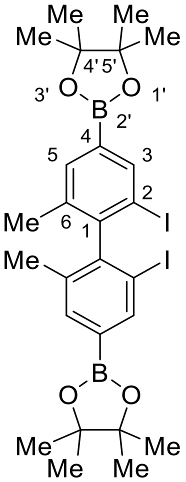


**9**

#### 1.2.8.7 2,2'-diiodo-6,6'-dimethyl-[1,1'-biphenyl]-4,4'-diol (10)

A 250 mL round bottom flask equipped with stir bar was charged with biaryl **9** (2.74 g, 1.0 equiv, 4.00 mmol, 95%*ee* $S_a$) and THF (50 mL, 0.08 M). A solution of Oxone® (2.45 g, 4.0 equiv, 8.00 mmol) in water (48.0 mL) was added and the suspension stirred at 600 rpm at room temperature for 24 h. Aqueous 10% $Na_2SO_3$ solution (100 mL) was added, and the suspension stirred for 5 min. The aq. phase was extracted using $CH_2Cl_2$ (3x 150 mL) and the combined organic phases washed with sat. aq. NaCl solution and dried over $MgSO_4$. The solvent was removed in vacuo. The product was isolated by column chromatography (petroleum ether:EtOAc 7:3 v/v). and obtained as white solids (1.58 g, 3.39 mmol, 85%, 92%*ee* $S_a$).

A further experiment using biaryl **9** (2.74 g, 1.00 equiv, 4.00 mmol, 95%*ee* $R_a$) gave the enantiocomplementary product as white solids (1.52 g, 3.26 mmol, 82%, 94%*ee* $R_a$).

**$^1$H NMR** (600 MHz, $CDCl_3$): δ 7.30 (d, *J* = 2.5 Hz, 2H, H-3), 6.78 (d, *J* = 2.5 Hz, 2H, H-5), 4.80 (s, 2H, OH), 1.96 (s, 6H, Me).

**$^{13}$C NMR** (151 MHz, $CDCl_3$): δ 155.3 (C-4), 140.1 (C-1), 139.1 (C-6), 123.5 (C-3), 117.4 (C-5), 101.9 (C-1), 21.9 (Me).

**IR (ATR film) [cm$^{-1}$]:** 3342 (broad), 1595, 1566, 1441, 1415, 1328, 1270, 1198, 1131, 1112, 1007, 972, 909, 854, 795, 733, 614.

**TLC** (petroleum ether:EtOAc 6:4 v/v): $R_f$ = 0.52

**APCI-MS:** m/z: ([M + H$^+$]): found: 467.0

**Elemental analysis** (calcd., found for $C_{14}H_{12}I_2O_2$): C (36.08, 36.37), H (2.60, 2.87)

**Melting point:** 171 – 173 °C (*rac*)

162 – 167 °C ($S_a$, 97%*ee* HPLC)

**HPLC:** Lux® Amylose-1 (Phenomenex) 250 ° 4.6 mm, 25 °C, 0.5 mL min$^{-1}$, 205 nm, *n*-heptane:*i*-PrOH 80:20 (v/v) $t_R(S_a)$ = 12.4 min, $t_R(R_a)$ = 11.3 min

**Optical rotation**: $[\alpha]^{25}_D$ = +26.2 (±0.2, duplicate) (c = 0.30, $CHCl_3$, $S_a$, 97%*ee* by chiral HPLC)

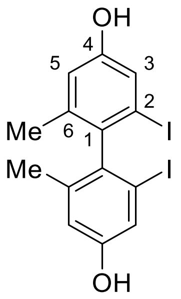


**10**

2,2'-diiodo-6,6'-dimethyl-[1,1'-biphenyl]-4-ol (**24**) was obtained as a sideproduct in analytical quantities.

**$^1$H NMR** (600 MHz, $CDCl_3$): δ 7.79 (d, *J* = 7.9 Hz, 1H, H-3′), 7.32 (d, *J* = 2.5 Hz, 1H, H-3), 7.26 (d, *J* = 7.5 Hz, 1H, H-5′), 6.98 (t, *J* = 7.7 Hz, 1H, H-4′), 6.79 (d, *J* = 2.5 Hz, 1H, H-5), 5.00 (s, 1H, OH), 2.02 (s, 3H, Me′), 1.95 (s, 3H, Me).

**$^{13}$C NMR** (151 MHz, $CDCl_3$): δ 155.3 (C-4), 147.2 (C-1′), 140.6 (C-1), 138.5, 138.4, 136.8 (C-3′), 130.1 (C-5′), 129.5 (C-4′), 123.6 (C-3), 117.5 (C-5), 102.1 (C-2′), 100.6 (C-2), 21.7 (Me), 21.6 (Me)

**IR (ATR film) [$cm^{-1}$]:** 3389 (broad), 3038, 2924, 2847, 1603, 1565, 1448, 1442, 1420, 1275, 1206, 1122, 1000, 847, 804, 763, 732.

**TLC** (petroleum ether:EtOAc 6:4 v/v): $R_f$ = 0.69

**APCI-MS:** m/z: ([M + $H^+$]): found: 451.0

**Elemental analysis** (calcd., found for $C_{14}H_{12}I_2O$): C (37.36, 37.13), H (2.69, 2.85)

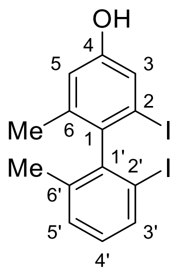


**24**

### 1.2.8.8 2,2'-diiodo-4,4'-dimethoxy-6,6'-dimethyl-1,1'-biphenyl (11)

A 100 mL round bottom flask equipped with stir bar was charged with biaryl **10** (1.86 g, 1.0 equiv, 4.00 mmol, 92%*ee* $S_a$) and acetone (20 mL, 0.2 M). $K_2CO_3$ (1.66 g, 3.0 equiv, 12.0 mmol) was added and stirred at 700 rpm. The reaction was cooled to 0 °C and $Me_2SO_4$ (0.84 mL, 2.2 equiv, 8.80 mmol) was added dropwise. A reflux condenser was equipped, and the reaction mixture stirred at 65 °C for 2 h. Saturated aqueous $Na_2SO_3$ (10 mL) solution was added and the reaction stirred at 40 °C for 20 min to quench excess $Me_2SO_4$. The aq. phase was then extracted using EtOAc (3x 50 mL), the combined organic phases were washed with sat. aq. NaCl solution, dried over $MgSO_4$ and solvents removed in vacuo. The product was obtained as a white solids (1.76 g, 3.56 mmol, 89%, 92%*ee* $S_a$).

A further experiment using biaryl **10** (1.32 g, 1.00 equiv, 2.83 mmol, 94%*ee* $R_a$) gave the product as a white solid (1.32 g, 2.68 mmol, 95%, 95%*ee* $R_a$).

**$^1$H NMR** (600 MHz, $CDCl_3$): δ 7.33 (d, *J* = 2.5 Hz, 2H, H-3), 6.83 (d, *J* = 2.6 Hz, 2H, H-5), 3.82 (s, 6H, OMe), 1.98 (s, 6H, Me).

**$^{13}$C NMR** (151 MHz, $CDCl_3$): δ 159.2 (C-4), 140.2 (C-6), 138.8 (C-1), 121.6 (C-3), 116.4 (C-5), 102.0 (C-2), 55.5 (OMe), 22.0 (Me).

**IR (ATR film) [cm$^{-1}$]:** 2925, 2834, 1593, 1540, 1464, 1426, 1283, 1252, 1214, 1131, 1056, 987, 851, 775, 730, 586, 495.

**TLC** (petroleum ether:EtOAc 7:3 v/v): $R_f$ = 0.65

**HR-MS (ESI):** m/z calculated for $[C_{16}H_{17}I_2O_2]^+$ ([M + H$^+$]): 494.9313, found: 494.9310.

**Melting point:** 96 – 98 °C (*rac*)

109 – 113 °C ($S_a$, 98%*ee* HPLC)

**HPLC:** Chiralpak® IC (Daciel) 250 ° 4.6 mm, 25 °C, 0.5 mL min$^{-1}$, 208 nm, *n*-heptane:*i*-PrOH 99.9:0.1 (v/v) $t_R$($S_a$) = 8.9 min, $t_R$($R_a$) = 8.5 min

**Optical rotation**: $[\alpha]^{25}_D$ = +31.9 (±0.1, duplicate) (c = 0.99, $CHCl_3$, $S_a$, 98%*ee* by chiral HPLC)

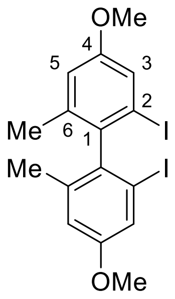


**11**

**1.2.8.9 4,4'-dimethoxy-6,6'-dimethyl-[1,1'-biphenyl]-2,2'-diol (1)**

A dry 250 mL Schlenk-vial equipped with stir bar was charged with biaryl **11** (1.72 g, 1.0 equiv, 3.49 mmol, 94%*ee* $S_a$) and anhydrous $Et_2O$ (70 mL, 0.05 M). To the stirred (700 rpm) solution at -78 °C was added a solution of *n*-BuLi in hexanes (2.5 M, 6.15 mL, 4.4 equiv, 15.6 mmol). The mixture was stirred at -78 °C for 1 h. Then freshly distilled nitrobenzene (3.58 mL, 10.0 equiv, 34.9 mmol) was added at once at -78 °C. The mixture was then stirred at -78 °C for 1 h. The reaction was left to warm to room temperature and stirred for 30 min. Then MeOH (20 mL) was added and the reaction was stirred for 10 min. $K_2HPO_4$/$KH_2PO_4$-buffer (KPi-buffer, 1 M, pH 7, 40 mL) was added and the aq. phase extracted with EtOAc (3x 100 mL). The combined organic phases were washed with sat. aq. NaCl solution, dried over $MgSO_4$ and solvents removed in vacuo. The product was isolated by column chromatography (petroleum ether:EtOAc 75:25 v/v) and obtained as off-white solids (645 mg, 2.35 mmol, 67%, 94%*ee* $S_a$). The product was recrystallized (petroleum ether:EtOAc 4:6 v/v, 5.2 mL) to provide the product as off-white solids (463 mg, 1.69 mmol, 48%, >99%*ee* $S_a$).

A further experiment using biaryl **11** (974 mg, 1.0 equiv, 1.97 mmol, 94%*ee* $R_a$) gave the product as white solids (311 mg, 1.14 mmol, 58%, 94%*ee* $R_a$). After recrystallization the product was obtained as off-white solids (254.4 mg, 0.93 mmol, 47%, >99%*ee* $R_a$).

The analytical data were in accordance with literature.[12]

**$^1$H NMR** (600 MHz, $CDCl_3$): δ 6.49 (d, *J* = 2.5 Hz, 2H, H-5), 6.47 (d, *J* = 2.5 Hz, 2H, H-3), 4.84 (s, 2H, OH), 3.81 (s, 6H, OMe), 1.97 (s, 6H, Me).

**$^{13}$C NMR** (151 MHz, $CDCl_3$): δ 161.1 (C-4), 155.4 (C-2), 140.5 (C-6), 111.3 (C-1), 109.0 (C-5), 98.4 (C-3), 55.3 (OMe), 19.8 (Me).

**IR (ATR film) [$cm^{-1}$]:** 3446 (broad), 2946, 2839, 1615, 1576, 1480, 1441, 1330, 1300, 1195, 1148, 1070, 1040, 836.

**TLC** (petroleum ether:EtOAc, 7:3 v/v): $R_f$ = 0.26

**APCI-MS:** m/z: ([M + $H^+$]): found: 275.

**Melting point:** 148 – 150 °C (*rac*) (148 – 150 °C)[12]

170 – 172 °C ($S_a$, >99%*ee* HPLC) (172 – 174°C)[12]

169 – 171 °C ($R_a$, >99%*ee* HPLC)

**HPLC:** Lux® Amylose-1 (Phenomenex) 250 ° 4.6 mm, 25 °C, 0.5 mL $min^{-1}$, 205 nm, *n*-heptane:*i*-PrOH 50:50 (v/v) $t_R(S_a)$ = 14.5 min, $t_R(R_a)$ = 53.1 min

**Optical rotation**: $[\alpha]^{25}_D$ = -31.1 (±0.7, duplicate) (c = 0.98, $CHCl_3$, $S_a$, >99%*ee* by chiral HPLC) -31.9[12]

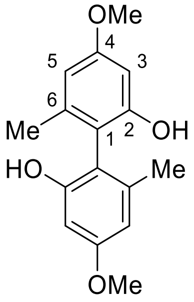


**1**

**1.2.8.10 Synthesis of *rac*-1,1'-(2,2'-dihydroxy-4,4'-dimethoxy-6,6'-dimethyl-[1,1'-biphenyl]-3,3'-diyl)bis(ethan-1-one) (12)**

Following our literature known procedure[12] a 50 mL Schlenk-vial equipped with stir bar was charged with biaryl **1** (343 mg, 1.0 equiv, 1.25 mmol, >99%*ee* $S_a$) and anhydr. chlorobenzene (12.5 mL, 0.1 M). The solution was stirred at 0 °C (600 rpm), acetyl chloride (200 µL, 2.2 equiv, 2.75 mmol) added dropwise and then the mixture stirred at room temperature for 30 minutes. Then $TiCl_4$ (820 µL, 6.0 equiv, 7.50 mmol) was added dropwise and the reaction mixture stirred at 70 °C for 4 h. Then the dark brown mixture was slowly transferred into a 250 mL Erlenmeyer beaker equipped with stir bar with $K_2HPO_4/KH_2PO_4$-buffer (KPi-buffer, 1 M, 100 mL, pH 7) at 0 °C. The pH was then

adjusted to pH 4 by the addition of 1 M HCl-solution. The resulting white suspension was then stirred for 15 min, sonicated for 15 min, and then stirred for 30 min. The mixture was filtered over a pad of celite using a wide Buchner-type funnel. The filter cake was washed with $CH_2Cl_2$ (100 mL), $H_2O$ (100 mL), then again with $CH_2Cl_2$ (800 mL). The filter cake was then transferred into an Erlenmeyer flask and stirred with $CH_2Cl_2$ (250 mL) overnight. The suspension was then again filtered over celite. The filtrates were combined, and the aqueous phase extracted with $CH_2Cl_2$ (4x 250 mL). The combined aqueous phases were washed with sat. aq. NaCl solution, dried over $MgSO_4$ and solvents removed in vacuo. The product was isolated by washing the crude product with acetone (10 mL). The remaining product in the wash fraction was isolated by column chromatography ($CH_2Cl_2$:PhMe 9:1 v/v to $CH_2Cl_2$:MeOH 99:1 v/v). The thus combined isolated product was obtained as yellow solids (332 mg [washed] + 20 mg [column], 0.98 mmol, 78%, >99%*ee* $S_a$).

A further experiment using biaryl **1** (233 mg, 1.0 equiv, 0.85 mmol, >99%*ee* $R_a$) gave the product as yellow solids (181 mg [washed] + 48 mg [column], 0.64 mmol, 75%, >99%*ee* $R_a$).

The analytical data were in accordance with literature.[12]

**$^1$H NMR** (600 MHz, $CDCl_3$): δ 13.68 (s, 1H, OH), 6.38 (s, 1H, H 5), 3.93 (s, 3H, OMe), 2.67 (s, 3H, COMe), 2.06 (s, 3H, Me)

**$^{13}$C NMR** (151 MHz, $CDCl_3$): δ 204.8 (<u>CO</u>Me), 162.4 (C-2), 160.8 (C-4), 147.5 (C-6), 117.4 (C-1), 109.4 (C-3), 103.0 (C-5), 55.6 (OMe), 33.7 (CO<u>*Me*</u>), 21.0 (Me).

**IR (ATR film) [cm$^{-1}$]**: 1599, 1360, 1283, 1202, 1119, 9693, 865, 834, 655, 573, 534.

**TLC** (petroleum ether:EtOAc, 7:3 v/v): $R_f$ = 0.32

**TLC** ($CH_2Cl_2$:PhMe, 9:1 v/v): $R_f$ = 0.25

**HR-MS (ESI):** m/z calculated for $[C_{20}H_{23}O_6]^+$ ([M + H$^+$]): 359.1489, found: 359.1492.

**Melting point:** 233 – 235 °C (*rac*) (233 – 236 °C)[12]

231 – 235 °C ($S_a$, >99%*ee* HPLC) (209 – 210)[12]

231 – 234 °C ($R_a$, >99%*ee* HPLC)

**HPLC:** Lux® Amylose-1 (Phenomenex) 250 ° 4.6 mm, 25 °C, 0.5 mL min$^{-1}$, 274 nm, *n*-heptane:*i*-PrOH 50:50 (v/v) $t_R(S_a)$ = 7.5 min, $t_R(R_a)$ = 10.4 min

**Optical rotation**: $[\alpha]^{25}_{D}$ = +49.0 (±0.4, triplicate) (c = 0.96, $CHCl_3$, $S_a$, >99%*ee* by chiral HPLC) +43.1[12]

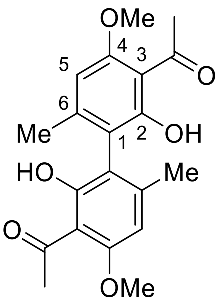


**12**

### 1.2.8.11 (2E,2'E)-1,1'-(2,2'-dihydroxy-4,4'-dimethoxy-6,6'-dimethyl-[1,1'-biphenyl]-3,3'-diyl)bis(3-(4-bromophenyl)prop-2-en-1-one) (13)

A vial equipped with a stirring bar was charged with acetophenone **12** (285 mg, 1.0 equiv, 0.80 mmol) and ethanol (3.2 mL, 0.25 M). An aqueous solution of KOH (3 M, 3.2 mL, 12.0 equiv, 9.60 mmol) and 4-bromo benzaldehyde (355 mg, 1.92 mmol, 2.4 equiv) were added consecutively. The mixture was stirred for 16 h after which aq. HCl-solution (1 M, 20 mL) was added. The aq. phase was extracted using $CH_2Cl_2$ (3x 30 mL), washed with sat. aq. NaCl solution, dried over $MgSO_4$ and solvents removed in vacuo. The product was isolated via column chromatography (petroleum ether:$CH_2Cl_2$ 4:6 v/v then petroleum ether:EtOAc 7:3 v/v). The isolated product was suspended in EtOAc and sonicated to remove traces of $CH_2Cl_2$, the process was repeated with MeOH to remove traces of $CH_2Cl_2$. The solvent was then removed *in vacuo*. The product was isolated as orange crystalline solids (276.5 mg, 0.40 mmol, 50%, >99%*ee* $S_a$).

A further experiment using biaryl **12** (143 mg, 1.0 equiv, 0.4 mmol, >99%*ee* $R_a$) gave the product as orange crystalline solids (101 mg, 0.15 mmol, 37%, >99%*ee* $R_a$).

A further experiment using biaryl **12** (1.43 g, 1.0 equiv, 4.00 mmol, *rac*) gave the product as orange crystalline solids (2.67 g, 3.88 mmol, 97%, *rac*). After the addition of HCl-solution, the solids were filtered off, washed with water and then MeOH (10 mL). The solids were then dissolved using $CH_2Cl_2$, washed with sat. aq. NaCl solution and dried over $MgSO_4$. MeOH was added (10 mL) and the solvents carefully removed in vacuo.

The analytical data were in accordance with literature.[9]

**$^1$H NMR** (600 MHz, $CDCl_3$): δ 13.65 (s, 2H, OH), 7.90 (d, *J* = 15.6 Hz, 2H, H-6′), 7.71 (d, *J* = 15.6 Hz, 2H, H-5′), 7.54 (d, *J* = 8.4 Hz, 4H, H-3′), 7.47 (d, *J* = 8.5 Hz, 4H, H-2′), 6.43 (s, 2H, H-5), 3.99 (s, 6H, OMe), 2.11 (s, 6H, Me).

**$^{13}$C NMR** (151 MHz, $CDCl_3$): δ 194.0 (C=O), 162.9 (C-2), 160.3 (C-4), 147.8 (C-6), 141.1 (C-5′), 134.7 (C-1′), 132.3 (C-3′), 129.9 (C-2′), 128.7 (C-6′), 124.4 (C-4′), 117.8 (C-1), 110.0 (C-3), 103.5 (C-5), 56.0 (OMe), 21.1 (Me).

**IR (ATR film) [$cm^{-1}$]**: 2970, 2941, 2848, 2251, 1627, 1605, 1559, 1485, 1389, 1359, 1323, 1213, 1178, 1141, 1114, 1072, 1035, 1009, 979, 946, 908, 875, 819, 801, 786, 731, 648, 632, 604, 571, 535, 507, 491.

**TLC** (petroleum ether:$CH_2Cl_2$, 5:5 v/v): $R_f$ = 0.11, (petroleum ether:EtOAc, 7:3 v/v): $R_f$ = 0.54

**HR-MS (ESI):** m/z calculated for $[C_{34}H_{29}O_6Br_2]^+$ ([M + $H^+$]): 691.0325, found: 691.0324.

**Melting point:** 230 – 236 °C (*rac*)

233 – 237 °C (decomposition) ($S_a$, >99%*ee* HPLC)

227 – 230 °C (decomposition) ($R_a$, >99%*ee* HPLC)

**HPLC:** CHIRALPAK® IA (Daciel) 250 ° 4.6 mm, 25 °C, 0.5 mL $min^{-1}$, 335 nm, *n*-heptane:*i*-PrOH 50:50 (v/v) $t_R(S_a)$ = 25.5 min, $t_R(R_a)$ = 40.9 min

**Optical rotation**: $[\alpha]^{25}_D$ = +69.4° (±0.6, duplicate) (c = 1.01, $CHCl_3$, $S_a$, >99%*ee* by chiral HPLC)

**X-ray:** (*R*)-**13** was dissolved in $CDCl_3$, filtered over a syringe filter into a vial and the solvent left to evaporate over time. CCDC 2342250

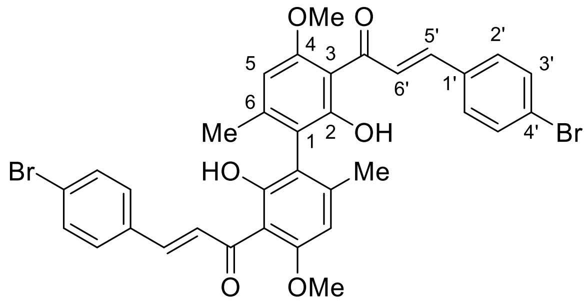

## 13

**1.2.8.12 2,2'-bis(4-bromophenyl)-5,5'-dimethoxy-7,7'-dimethyl-4*H*,4'*H*-[8,8'-bichromene]-4,4'-dione (2)**

A 2-dr. vial equipped with stir bar was charged with bichalcone **13** (96.9 mg, 1.0 equiv, 140 µmol, >99% *ee* $S_a$) and a solution of iodine in degassed DMSO (0.08 M, 175 µL, 0.1 equiv, 14.0 µmol). The reaction was stirred at 150 °C for 2 h. Sat. aq. $Na_2SO_3$ (1 mL) solution was added and the aq. phase extracted with $CH_2Cl_2$ (3x 15 mL). The combined org. phases were washed with sat. aq. NaCl solution, water (3x) sat. aq. NaCl solution again, dried over $MgSO_4$ and solvents removed in vacuo. The product was obtained as off-white solids (89.0 mg, 129 µmol, 92%, >99%*ee* $S_a$).

A further experiment using bichalcone **13** (83.1 mg, 1.0 equiv, 120 µmol, >99% *ee* $R_a$) gave the product as off-white solids (63.2 mg, 91.8 µmol, 77%, >99%*ee* $R_a$).

A further experiment using bichalcone **13** (1.29 g, 1.0 equiv, 1.86 mmol, *rac*) gave the product as off-white solids (1.22 g, 1.77 mmol, 95%, *rac*).

The analytical data were in accordance with literature.[9]

**$^1$H NMR** (600 MHz, $CDCl_3$): δ 7.42 (d, *J* = 8.7 Hz, 4H, H-3′), 7.14 (d, *J* = 8.7 Hz, 4H, H-2′), 6.89 (s, 2H, H-6), 6.69 (s, 2H, H-3), 4.10 (s, 6H, OMe), 2.18 (s, 6H, Me)

**$^{13}$C NMR** (151 MHz, $CDCl_3$): δ 178.3 (C=O), 159.7 (C-2), 159.4 (C-5), 155.6 (C-8a), 145.1 (C-7), 132.5 (C-2′), 130.1 (C-1′), 126.9 (C-3′), 126.2 (C-4′), 116.2 (C-8), 112.9 (C-4a), 108.8 (C-3), 108.5 (C-6), 56.7 (OMe), 20.8 (Me).

**IR (ATR film) [cm$^{-1}$]:** 3005, 2931, 2851, 2240, 1773, 1638, 1597, 1562, 1479, 1464, 1403, 1368, 1329, 1303, 1275, 1260, 1207, 1187, 1167, 1122, 1061, 1030, 1008, 977, 955, 907, 830, 794, 681, 645, 626, 573, 557, 532, 499, 478.

**TLC** ($CH_2Cl_2$:MeOH, 98:2 v/v): $R_f$ = 0.15

**HR-MS (ESI):** m/z calculated for $[C_{34}H_{25}O_6Br_2]^+$ ($[M + H^+]$): 687.0012, found: 687.0017.

**Melting point:** 245 °C (decomposition) (*rac*)

270 °C (decomposition) ($S_a$, >99%*ee* HPLC)

270 °C (decomposition) ($R_a$, >99%*ee* HPLC)

**HPLC:** Lux® Amylose-1 (Phenomenex) 250 ° 4.6 mm, 25 °C, 0.5 mL min$^{-1}$, 331 nm, *n*-heptane:*i*-PrOH 50:50 (v/v) $t_R(S_a)$ = 18.0 min, $t_R(R_a)$ = 24.3 min

**Optical rotation**: $[\alpha]^{25}_D$ = +25.7 (±0.1, duplicate) (c = 1.08, $CHCl_3$, $S_a$, >99%*ee* by chiral HPLC)

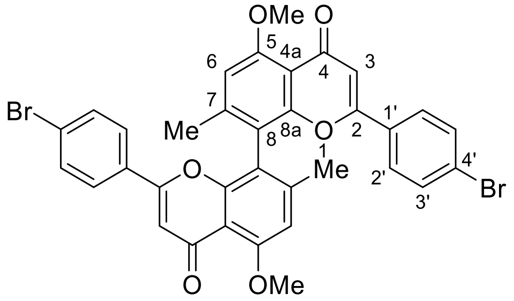


**2**

### 1.2.9 Miscellaneous Syntheses

#### 1.2.9.1 N-methyl-[1,1'-biphenyl]-2-amine (25)

A dry 250 mL Schlenk round bottom flask equipped with stir bar was charged with 2-amino biphenyl (2.00 g, 1.00 equiv, 11.8 mmol) and anhydrous THF (40 mL, 0.30 M). The mixture was cooled to 0 °C, *n*-BuLi in hexanes(2.3 M, 5.40 mL, 1.05 equiv, 12.4 mmol) was added dropwise and the reaction mixture stirred at 0 °C for 1 h. Iodomethane (740 µL, 1.01 equiv, 11.9 mmol) was added dropwise and the reaction stirred for a further 30 min at 22 °C. Sat. aq. $NaHCO_3$ solution (25 mL) and $H_2O$ (25 mL)were added and the aq. phase extracted with $Et_2O$ (3x), the combined org. phases dried over $MgSO_4$ and solvents removed in vacuo. The product was obtained as a 95:5 mixture of mono and dimethylated products according to $^1$H NMR and used in the next step without further isolation in a yield of 2.15 g.

**$^1$H NMR** (600 MHz, $CDCl_3$): δ 7.40 – 7.48 (m, 4H), 7.35 (tt, *J* = 6.6, 1.8 Hz, 1H), 7.28 (dd, *J* = 7.8, 1.7 Hz, 1H), 7.09 (dd, *J* = 7.4, 1.7 Hz, 1H), 6.78 (td, *J* = 7.4, 1.1 Hz, 1H), 6.68 (m, 1H), 3.96 (s, 1H, *NH*Me), 2.76 – 2.83 (m, 3H, NH*Me*)

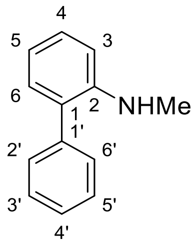


**25**

### 1.2.9.2 Bis-((2'-(methylamino)-[1,1'-biphenyl]-2-yl)((methylsulfonyl)oxy)palladium(II)) (Pd G4)$_2$ (26)

A 100 mL round bottom flask equipped with stir bar was charged with 2-amino biaryl **25** (2.15 g, 1.0 equiv, 11.7 mmol) and THF (30 mL, 0.39 M). Methanesulfonic acid (720 µL, 0.95 equiv, 11.1 mmol) was added dropwise and the reaction mixture stirred at 22 °C for 15 min. Pd(OAc)$_2$ (2.48 g, 0.95 equiv, 11.1 mmol) was added in one portion, the walls of the vessel rinsed off using THF (15 mL). The flask was capped with a rubber septum and the mixture stirred at 50 °C for 1 h. Solvents were removed in vacuo, Et$_2$O (40 mL) added and the mixture sonicated for 1 h. The precipitating tan solids were filtered off washing with additional Et$_2$O and dried in vacuo. The product was obtained without any additional isolation step as tan solids in a yield of 4.23 g (5.52 mmol, 94%).

**$^1$H NMR** (600 MHz, CDCl$_3$): δ 7.53 (d, *J* = 7.7 Hz, 2H), 7.34 (d, *J* = 7.6 Hz, 2H), 7.22 (t, *J* = 7.5 Hz, 3H), 7.11 – 7.18 (m, 3H), 7.08 (t, *J* = 7.3 Hz, 2H), 6.98 (t, *J* = 7.5 Hz, 2H), 2.80 (s, 6H), 2.61 (s, 6H).

**$^{13}$C NMR** (151 MHz, CDCl$_3$): δ 140.8, 139.0 (d, *J* = 53.6 Hz), 134.4 (d, *J* = 74.1 Hz), 128.8, 128.0, 127.4, 126.6, 125.9, 124.8, 120.7, 44.6, 39.50.

**ESI-MS:** m/z: ([M - MeSO$_3^-$ + 2 MeCN]): found: 370.1 (100), 372.1 (84), 369.1 (78) (MeCN:H$_2$O 8:2 v/v + 0.1% HCO$_2$H)

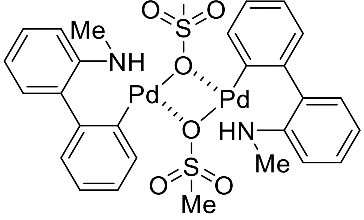


**26**

#### 1.2.9.3 6,6'-dimethyl-[1,1'-biphenyl]-2,2'-diol (27)

A microwave vial equipped with stir bar was charged with 2,2′-diiodo biaryl **4** (43.3 mg, 1.0 equiv, 0.100 mmol) and anhydr. $Et_2O$ (2.0 mL, 0.05 M). To the stirred (700 rpm) solution at -78 °C was added a solution of *n*-BuLi in pentanes (2.5 M, 88.0 µL, 2.2 equiv, 0.220 mmol). The mixture was stirred at -78 °C for 1 h. Then, freshly distilled nitrobenzene (103 µL, 10.0 equiv, 1.00 mmol) was added at once at -78 °C. The mixture was then stirred at -78 °C for 1 h. The reaction was left to warm to room temperature and stirred for 30 min. Then MeOH (2.0 mL) was added, and the reaction was stirred for 10 min $H_2O$ (10 mL) was added, the aq. phase extracted with $CH_2Cl_2$ (3x) and the combined org. phases discarded. Then HCl-solution (1 M, 2.0 mL) was added and the aq. phase extracted with $CH_2Cl_2$ (3x 20 mL). The combined organic phases were washed with sat. aq. NaCl solution, dried over $MgSO_4$ and solvents removed in vacuo. The product was obtained as off-white solids in a yield of 11.2 mg (52.3 µmol, 52%).

The analytical data were in accordance with literature.[2,13]

**$^1$H NMR** (600 MHz, $CDCl_3$): δ 7.17 – 7.35 (m, 2H, H-4), 6.93 (d, *J* = 7.5 Hz, 2H, H-5), 6.90 (d, *J* = 8.2 Hz, 2H, H-3), 4.72 (s, 2H, OH), 2.01 (s, 6H, Me).

**$^{13}$C NMR** (151 MHz, $CDCl_3$): δ 154.0 (C-2), 139.1 (C-6), 130.2 (C-4), 122.7 (C-5), 119.7 (C-1), 113.3 (C-3), 19.6 (Me).

**IR (ATR film) [cm$^{-1}$]:** 3484 (broad signal), 2930, 1580, 1466, 1338, 1277, 1262, 1178, 776, 746.

**Melting point**: °C (163 – 164 °C) (*rac*) (159 – 160 °C)[14]

**APCI-MS:** m/z: ([M + H$^+$]): found: 215.2

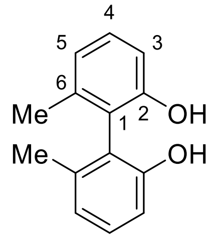


**27**

### 1.2.9.4 2-butyl-2',6-dimethyl-1,1'-biphenyl-6'-*d* (28)

A microwave vial equipped with stir bar was charged with 2,2′-diiodo biaryl **4** (43.4 mg, 1.0 equiv, 0.100 mmol) and anhydr. $Et_2O$ (2.0 mL, 0.05 M). To the stirred (700 rpm) solution at -78 °C was added a solution of *n*-BuLi in hexanes (2.5 M, 88.0 µL, 4.4 equiv, 0.220 mmol). The mixture was stirred at -78 °C for 1 h. Then $D_2O$ (100 µL, 45.0 equiv, 4.50 mmol) was added at once at -78 °C. The mixture was left to warm to room temperature and stirred for 30 min. Then MeOH (2.0 mL) was added and the reaction was stirred for 10 min. $K_2HPO_4$/$KH_2PO_4$-buffer (KPi-buffer, 1 M, pH 7, 5.0 mL) was added and the aq. phase extracted with EtOAc (3x 20 mL). The combined organic phases were washed with sat. aq. NaCl solution, dried over $MgSO_4$ and solvents removed in vacuo. 1,3,5-trimethoxybenzene (12.5 mg) was added to determine the conversion to product according to $^1$H NMR (90%). The product was isolated by filtration over silica washing with *n*-pentane and solvents removed in vacuo. The title compound was isolated as a colorless oil in analytical quantaties.

**$^1$H NMR** (600 MHz, $CDCl_3$): δ 7.24 – 7.31 (m, 3H, ArH), 7.22 (dd, *J* = 7.1, 2.3 Hz, 1H, ArH), 7.20 (t, *J* = 7.6 Hz, 1H, ArH), 7.13 (d, *J* = 7.6 Hz, 1H, H-5'), 7.10 (d, *J* = 7.5 Hz, 1H, H-3'), 7.03 (d, *J* = 7.3 Hz, 0H, D-6'), 2.24 – 2.32 (m, 1H, H-1b''), 2.17 (ddd, *J* = 13.7, 8.8, 7.0 Hz, 1H, H-1a''), 1.96 (s, 3H, Me'), 1.93 (s, 3H, Me), 1.38 (dqd, *J* = 8.8, 7.4, 1.7 Hz, 2H, H-2''), 1.16 (qd, *J* = 7.2, 3.4 Hz, 2H, H-3''), 0.75 (t, *J* = 7.4 Hz, 3H, H-4'').

**$^{13}$C NMR** (151 MHz, $CDCl_3$): δ 140.8, 140.7, 140.2, 136.9, 136.1, 136.0, 130.0, 127.3 (C-3'), 127.1, 127.1, 126.5 (C-3), 125.7, 33.2 (C-1''), 33.1 (C-2''), 22.7 (C-3''), 20.6 (Me), 19.7 (Me'), 13.9 (C-4'').

**IR (ATR film) [cm$^{-1}$]:** 2953, 2923, 2862, 1740, 1459, 1383, 1011, 799, 761, 655.

**TLC** (100% petroleum ether): $R_f$ = 0.56

**GC-MS(EI-MS):** m/z: ([M$^+$]): found: 239.3

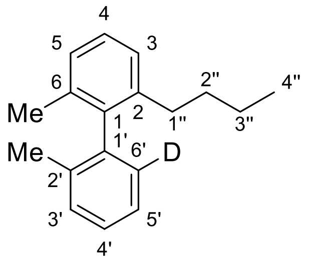

## 28

### 1.2.9.5 5-methoxy-7-methyl-2-(4-morpholinophenyl)-4H-chromen-4-one (29)

A 2 dr. vial was charged with flavone[9] **23** (34.5 mg, 1.00 equiv, 0.100 mmol), RuPhos (18.6 mg, 40 mol%, 40.0 μmol), $(Pd\ G4)_2$ (**26**) (1.9 mg, 2.5 mol%, 2.5 μmol). A separate dry vial was charged with morpholine (38.4 mg, 4.40 equiv, 0.440 mmol) (making sure not to purge the vial with an argon balloon so the volatile amine wouldn't evaporate) and PhMe (2 mL). Then that solution of amine in PhMe (1 mL, 2.20 equiv, 0.220 mmol) was added, the reaction vessel capped and sealed using PTFE tape and then stirred at 80 °C for 16 h. After letting the reaction cool, the reaction was filtered over a pad of silica into a vial containing a defined amount of 1,3,5-trimethoxybenzene (0.4 – 0.8 equiv), washing with $CH_2Cl_2$:MeOH (7:3 v/v). The solvent was removed and conversion to product determined by $^1$H-NMR. The product was isolated by column chromatography ($CH_2Cl_2$:MeOH 97:3 v/v to $CH_2Cl_2$:EtOAc 7:3 v/v) as brown solids in a yield of 31.4 mg (89.4 μmol, 89%)

**$^1$H NMR** (600 MHz, $CDCl_3$): δ 7.78 (d, *J* = 8.4 Hz, 2H, H-2'), 6.95 (d, *J* = 8.4 Hz, 2H, H-3'), 6.93 (s, 1H, H-8), 6.60 (s, 2H, H-3 + H-6), 3.97 (s, 3H, OMe), 3.87 (t, *J* = 4.8 Hz, 4H, H-2''), 3.28 (t, *J* = 4.8 Hz, 4H, H-1''), 2.44 (s, 3H, Me).

**$^{13}$C NMR** (151 MHz, $CDCl_3$): δ 178.4 (C-4), 161.3 (C-2), 159.6 (C-5), 158.4 (C-8a), 153.2 (C-4'), 144.9 (C-7), 127.5 (C-2'), 121.8 (C-1'), 114.6 (C-3'), 112.5 (C-4a), 110.3 (C-8), 107.7 (C-6), 107.1 (C-3), 66.7 (C-2''), 56.5 (OMe), 48.0 (C-1''), 22.4 (Me).

**IR (ATR film) [cm$^{-1}$]**: 3466, 2920, 2847, 1633, 1603, 1517, 1481, 1377, 1335, 1233 1203, 1118, 1049, 926, 826

**TLC** ($CH_2Cl_2$:MeOH, 95:5 v/v): $R_f$ = 0.46

**HR-MS (ESI):** m/z calculated for $[C_{21}H_{22}NO_4]^+$ ($[M + H^+]$): 352.1543, found: 352.1549 (-1.5 ppm).

**Melting point:** 218 – 221 °C (brown discoloration)

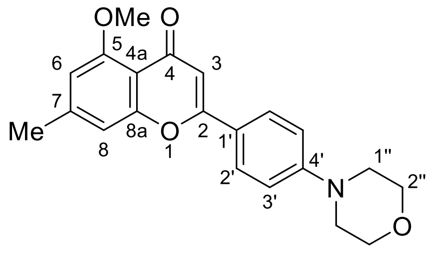


**29**

#### 1.2.9.6 2-(4-(benzylamino)phenyl)-5-methoxy-7-methyl-4H-chromen-4-one (30)

A 2 dr. vial was charged with flavone[9] **23** (34.5 mg, 1.00 equiv, 0.100 mmol), RuPhos (4.7 mg, 10 mol%, 10 µmol), $(Pd\ G4)_2$ (**26**) (1.9 mg, 2.5 mol%, 2.5 µmol). A separate dry vial was charged with benzylamine (47.1 mg, 4.40 equiv, 0.440 mmol) (making sure not to purge the vial with an argon balloon so the volatile amine wouldn't evaporate) and PhMe (2 mL). Then that solution of amine in PhMe (1 mL, 2.20 equiv, 0.220 mmol) was added, the reaction vessel capped and sealed using PTFE tape and then stirred at 80 °C for 16 h. After letting the reaction cool, the reaction was filtered over a pad of silica into a vial containing a defined amount of 1,3,5-trimethoxybenzene (0.4 – 0.8 equiv), washing with $CH_2Cl_2$:MeOH (7:3 v/v). The solvent was removed and conversion to product determined by $^1$H-NMR. The product was isolated by column chromatography ($CH_2Cl_2$:MeOH 97:3 v/v to $CH_2Cl_2$:EtOAc 7:3 v/v) as orange solids in analytical quantities. (54% conversion to product according to $^1$H-NMR).

**$^1$H NMR** (600 MHz, $CDCl_3$): δ 7.66 – 7.75 (m, 2H, H-2'), 7.36 (m, 4H, H-2''+3''), 7.30 (h, *J* = 4.1 Hz, 1H, H-4''), 6.91 (s, 1H, H-8), 6.64 (m, 2H, H-3'), 6.60 (s, 1H, H-6), 6.57 (s, 1H, H-3), 4.41 (s, 2H, $CH_2$), 3.97 (s, 3H, OMe), 2.44 (s, 3H, Me).

**$^{13}$C NMR** (151 MHz, $CDCl_3$): δ 178.4 (C-4), 161.7 (C-2), 159.6 (C-5), 158.4 (C-8a), 150.7 (C-4'), 144.7 (C-7), 138.5 (C-1''), 129.0 (C-2''/3''), 127.8 (C-2'), 127.7 (C-4''), 127.5 (C-2''/3''), 120.1 (C-1'), 112.7 (C-3'), 112.5 (C-4a), 110.3 (C-8), 107.7 (C-6), 106.4 (C-3), 56.5 (OMe), 47.9 (CH2), 22.4 (Me).

**IR (ATR film) [cm$^{-1}$]**: 3307, 2921, 2848, 1638, 1599, 1526, 1479, 1348, 1253, 1192, 1122, 1050, 837, 817, 698

**TLC** ($CH_2Cl_2$:MeOH, 96:4 v/v): $R_f$ = 0.35

**HR-MS (ESI):** m/z calculated for $[C_{21}H_{22}NO_4]^+$ ($[M + H^+]$): 372.1595, found: 352.1594 (-0.2 ppm).

**Melting point:** 250 – 251 °C

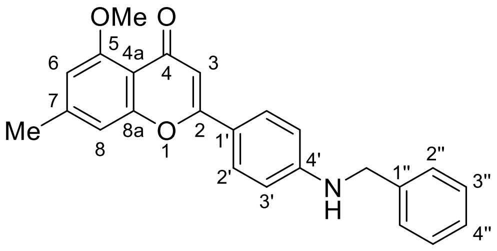


**30**

## General Procedure 1 Buchwald Hartwig Amination (GP1)

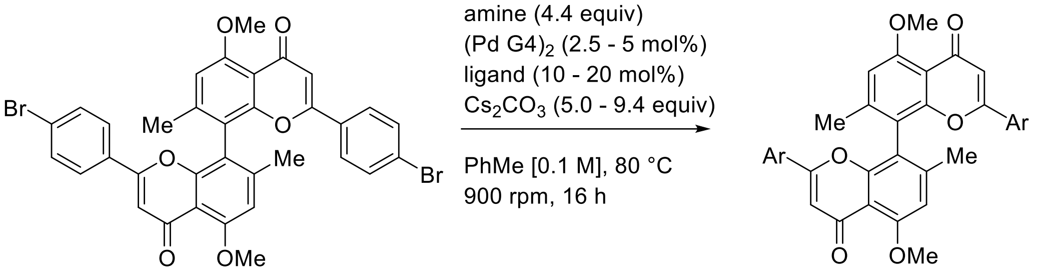


Hydrochloride amine (**GP**1**a**)

A 2-dr vial equipped with stir bar which were dried at 110 °C over night was left to cool under Argon-atmosphere. The vial was then charged with $Cs_2CO_3$ (306 mg, 9.40 equiv, 0.940 mmol), biflavone **2** (68.8 mg, 1.00 equiv, 0.100 mmol), ligand (10 – 20mol%), (Pd G4)$_2$ (**26**) (3.8 mg, 5 mol%, 10.0 µmol) and hydrochloride amine (4.4 equiv) in that order. Then PhMe (1 mL, 0.1 M) was added, the reaction vessel capped and sealed using PTFE tape and then stirred at 80 °C for 16 h (900 rpm). After letting the reaction cool, the reaction was filtered over a pad of silica into a vial containing a defined amount of 1,3,5-trimethoxybenzene (0.4 – 0.8 equiv), washing with $CH_2Cl_2$:MeOH (9:1 v/v). The solvent was removed and conversion to product determined by $^1$H-NMR. The product was isolated by column chromatography ($CH_2Cl_2$:EtOAc 1:9 v/v to $CH_2Cl_2$:MeOH 96:4 v/v).

Amine (**GP**1**b**)

A 2-dr vial equipped with stir bar which were dried at 110 °C over night was left to cool under Argon-atmosphere. The vial was then charged with $Cs_2CO_3$ (306 mg, 9.40 equiv, 0.940 mmol), biflavone **2** (68.8 mg, 1.00 equiv, 0.100 mmol), ligand (10 – 20mol%), (Pd G4)$_2$ (**26**) (3.8 mg, 5 mol%, 10.0 µmol). Then a solution of amine in PhMe (1 mL, 4.4 equiv, 0.440 mmol) prepared in a separate dry vial was added (making sure not to purge the vial with an argon balloon so volatile amines wouldn't evaporate), the reaction vessel capped and sealed using PTFE tape and then stirred at 80 °C for 16 h. After letting the reaction cool, the reaction was filtered over a pad of silica into a vial containing a defined amount of 1,3,5-trimethoxybenzene (0.4 – 0.8 equiv), washing with $CH_2Cl_2$:MeOH (9:1 v/v). The solvent was removed and conversion to product determined by $^1$H-NMR. The product was isolated by column chromatography ($CH_2Cl_2$:EtOAc 1:9 v/v to $CH_2Cl_2$:MeOH 96:4 v/v).

### 1.2.9.7 2,2'-bis(4-(dimethylamino)phenyl)-5,5'-dimethoxy-7,7'-dimethyl-4*H*,4'*H*-[8,8'-bichromene]-4,4'-dione (14)

The title compound was synthesized according to **GP**1**a** using biflavone **2** (68.8 mg, 1.0 equiv, 100 µmol, *rac*), RuPhos (9.3 mg, 20 mol%, 20 µmol), (Pd G4)$_2$ (**26**) (1.9 mg, 2.5 mol%,2.5 µmol) and dimethylamine hydrochloride (35.9 mg, 4.40 equiv, 0.440 mmol). The product was isolated as yellow solids in a yield of 51.3 mg (83.5 µmol, 83%, *rac*).

**Enantiopure**

A further experiment following **GP**1**a** using biflavone **2** (34.4 mg, 1.0 equiv, 50.0 µmol, >99%*ee* $S_a$), RuPhos (4.7 mg, 20 mol%, 10 µmol), (Pd G4)$_2$ (**26**) (1.9 mg, 5 mol%,2.5 µmol) and dimethylamine hydrochloride (17.9 mg, 2.20 equiv, 0.220 mmol) gave the product as pale-yellow solids (24.2 mg, 39.4 µmol, 79%, >99%*ee* $S_a$). (81% conversion to product according to $^1$H-NMR) (isolation by column chromatography $CH_2Cl_2$:EtOAc 9:1 v/v to $CH_2Cl_2$:MeOH 97:3)

A further experiment following **GP**1**a** using biflavone **2** (34.4 mg, 1.0 equiv, 50.0 µmol, >99%*ee* $R_a$), RuPhos (4.7 mg, 20 mol%, 10 µmol), (Pd G4)$_2$ (**26**) (1.9 mg, 5 mol%,2.5 µmol) and dimethylamine hydrochloride (17.9 mg, 2.20 equiv, 0.220 mmol) gave the product as pale-yellow solids (27.8 mg, 45.2 µmol, 90%, >99%*ee* $R_a$). (89% conversion to product according to $^1$H-NMR) (isolation by column chromatography $CH_2Cl_2$:EtOAc 9:1 v/v to $CH_2Cl_2$:MeOH 97:3)

The analytical data were in accordance with literature.[9]

**$^1$H NMR** (600 MHz, $CDCl_3$): δ 7.16 (d, *J* = 9.0 Hz, 4H, H-2'), 6.86 (s, 2H, H-6), 6.58 (s, 2H, H-3), 6.51 (d, *J* = 9.1 Hz, 4H, H-3'), 4.10 (s, 6H, OMe), 2.97 (s, 12H, $NMe_2$), 2.17 (s, 6H, Me).

**$^{13}$C NMR** (151 MHz, $CDCl_3$): δ 178.8 (C-4), 161.9 (C-2), 159.0 (C-5), 155.7 (C-8a), 152.3 (C-4'), 144.2 (C-7), 127.0 (C-2'), 117.7 (C-1'), 116.7 (C-8), 112.8 (C-4a), 111.8 (C-3'), 108.0 (C-6), 105.4 (C-3), 56.6 (OMe), 40.2 ($NMe_2$), 20.7 (Me).

**IR (ATR film) [cm$^{-1}$]**: 2923, 2237, 1731, 1604, 1591, 1524, 1495, 1364, 1335, 1302, 1284, 1251, 1197, 1171, 1120, 1063, 908, 819, 761, 728, 662, 642, 582, 570, 544, 531, 512.

**TLC** (EtOAc:$CH_2Cl_2$:MeOH, 7:2.5:0.5 v/v): $R_f$ = 0.16

**TLC** (EtOAc:$CH_2Cl_2$:MeOH, 96:4 v/v): $R_f$ = 0.29

**HR-MS (ESI):** m/z calculated for $[C_{38}H_{37}N_2O_6]^+$ ([M + $H^+$]): 617.2646, found: 617.2653.

**Melting point:** 196 - 199 °C (*rac*)

**HPLC:** Lux® Amylose-1 (Phenomenex) 250 ° 4.6 mm, 25 °C, 0.5 mL $min^{-1}$, 331 nm, *n*-heptane:*i*-PrOH 50:50 (v/v) $t_R$($S_a$) = 9.53 min, $t_R$($R_a$) = 12.8 min.

**Optical rotation:** $[\alpha]^{25}_D$ = +44.0 (±1.1, duplicate) (c = 0.250, $CH_2Cl_2$, $S_a$, >99%ee by chiral HPLC)

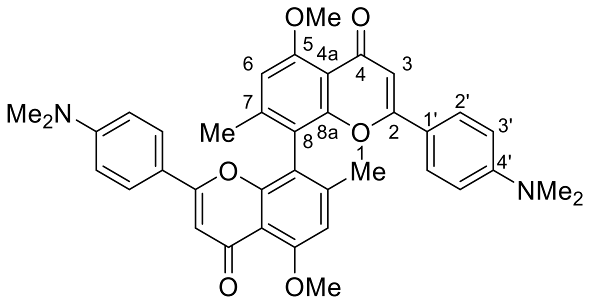


**14**

### 1.2.9.8 *rac*-2,2'-bis(4-(diethylamino)phenyl)-5,5'-dimethoxy-7,7'-dimethyl-4H,4'H-[8,8'-bichromene]-4,4'-dione (15)

The title compound was synthesized according to **GP1b** using RuPhos (9.3 mg, 20 mol%, 20 µmol), $(Pd\ G4)_2$ (**26**) (3.8 mg, 5 mol%, 5.0 µmol) and diethylamine (32.2 mg, 4.40 equiv, 0.440 mmol). The product was isolated as yellow solids in a yield of 50.0 mg (74.5 µmol, 75%).

**$^1$H NMR** (600 MHz, $CDCl_3$): δ 7.10 – 7.15 (m, 4H, H-2'), 6.83 (s, 2H, H-6), 6.55 (d, *J* = 2.0 Hz, 2H, H-3), 6.46 (d, *J* = 8.6 Hz, 4H, H-3'), 4.08 (s, 6H, OMe), 3.31 (q, *J* = 7.2 Hz, 8H, $\underline{CH_2}CH_3$), 2.13 (d, *J* = 1.7 Hz, 6H, Me), 1.07 – 1.16 (m, 12H, $CH_2\underline{CH_3}$)

**$^{13}$C NMR** (151 MHz, $CDCl_3$): δ 178.8 (C-4), 162.0 (C-2), 158.9 (C-5), 155.6 (C-8a), 149.9 (C-4'), 144.1 (C-7), 127.3 (C-2'), 116.7 (C-1'), 116.6 (C-8), 112.8 (C-4a), 111.2 (C-3'), 108.0 (C-6), 104.9 (C-3), 56.5 (OMe), 44.4 ($\underline{CH_2}CH_3$), 20.6 (Me), 12.6 ($CH_2\underline{CH_3}$).

**IR (ATR film) [$cm^{-1}$]**: 3474, 2969, 2924, 2851, 1635, 1602, 1590, 1374, 1333, 1200, 1119, 1061, 817

**TLC** ($CH_2Cl_2$:MeOH, 95:5 v/v): $R_f$ = 0.57

**HR-MS (ESI):** m/z calculated for $[C_{42}H_{45}N_2O_6]^+$ ([M + H$^+$]): 673.3272, found: 673.3274 (-0.3 ppm).

**Melting point:** 173 – 176 °C

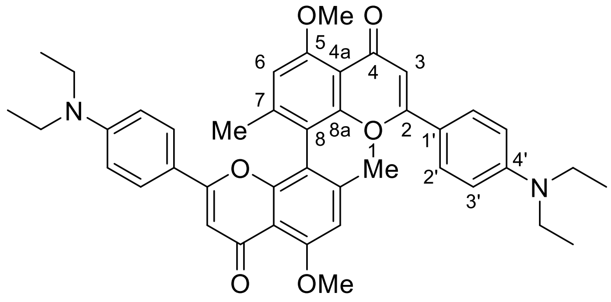


**15**

### 1.2.9.9 *rac*-5,5'-dimethoxy-7,7'-dimethyl-2,2'-bis(4-(pyrrolidin-1-yl)phenyl)-4H,4'H-[8,8'-bichromene]-4,4'-dione (16)

The title compound was synthesized according to **GP1b** using RuPhos (9.3 mg, 20 mol%, 20 µmol), $(Pd\ G4)_2$ (**26**) (1.9 mg, 2.5 mol%,2.5 µmol) and pyrrolidine (31.3 mg, 4.40 equiv, 0.440 mmol). The product was isolated as yellow solids in a yield of 56.2 mg (84.3 µmol, 84%).

**$^1$H NMR** (600 MHz, $CDCl_3$): δ 7.15 (d, *J* = 8.5 Hz, 4H, H-2’), 6.84 (s, 2H, H-6), 6.56 (s, 2H, H-3), 6.36 (d, *J* = 8.6 Hz, 4H, H-3’), 4.09 (s, 6H, OMe), 3.27 (d, *J* = 6.5 Hz, 8H, H-1’’), 2.16 (s, 6H, Me), 1.95 – 2.01 (m, 8H, H-2’’).

**$^{13}$C NMR** (151 MHz, $CDCl_3$): δ 178.8 (C-4), 162.2 (C-2), 159.0 (C-5), 155.7 (C-8a), 149.9 (C-4’), 144.1 (C-7), 127.2 (C-3’), 117.0 (C-1’), 116.7 (C-8), 112.8 (C-4a), 111.8 (C-3’), 108.0 (C-6), 105.0 (C-3), 56.6 (OMe), 47.6 (C-1’’), 25.6 (C-2’’), 20.7 (Me).

**IR (ATR film) [cm$^{-1}$]**: 3507, 2968, 2847, 1632, 1603, 1589, 15221, 1371, 1336, 1194, 1118, 1062, 815.

**TLC** ($CH_2Cl_2$:MeOH, 95:5 v/v): $R_f$ = 0.65

**HR-MS (ESI):** m/z calculated for $[C_{42}H_{41}N_2O_6]^+$ ([M + H$^+$]): 669.2959, found: 669.2956 (0.5 ppm).

**Melting point:** 281 – 285 °C (brown discoloration)

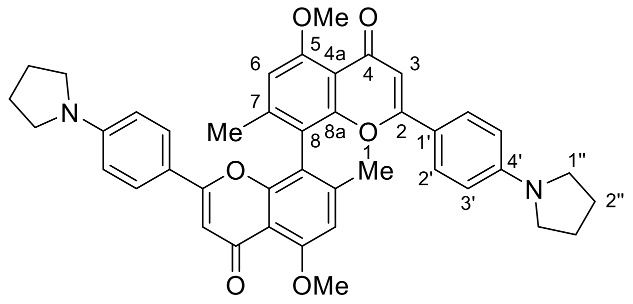


**16**

**1.2.9.10 *rac*-5,5'-dimethoxy-7,7'-dimethyl-2,2'-bis(4-morpholinophenyl)-4H,4'H-[8,8'-bichromene]-4,4'-dione (17)**

The title compound was synthesized according to **GP1b** using RuPhos (9.3 mg, 20 mol%, 20 µmol), $(Pd\ G4)_2$ (**26**) (1.9 mg, 2.5 mol%, 2.5 µmol) and morpholine (39.3 mg, 4.40 equiv, 0.440 mmol). The product was isolated as yellow solids in a yield of 65.6 mg (93.9 µmol, 94%)

**$^1$H NMR** (600 MHz, $CDCl_3$): δ 7.17 (d, *J* = 8.7 Hz, 4H, H-2'), 6.85 (s, 2H, H-6), 6.66 – 6.74 (m, 4H, H-3'), 6.60 (s, 2H, H-3), 4.09 (s, 6H, OMe), 3.81 (t, *J* = 4.9 Hz, 8H, H-2''), 3.19 (t, *J* = 4.8 Hz, 8H, H-1''), 2.17 (s, 6H, Me).

**$^{13}$C NMR** (151 MHz, $CDCl_3$): δ 178.7 (C-4), 161.3 (C-2), 159.1 (C-5), 155.7 (C-8a), 153.1 (C-4'), 144.5 (C-7), 127.0 (C-2'), 121.0 (C-1'), 116.6 (C-8), 114.5 (C-3'), 112.8 (C-4a), 108.2 (C-6), 106.3 (C-3), 66.7 (C-2''), 56.6 (OMe), 47.8 (C-1'), 20.7 (Me).

**IR (ATR film) [cm$^{-1}$]**: 3492, 2957, 2924, 2852, 1633, 1602, 1517, 1378, 1332, 1232, 1202, 1119, 1064, 928, 825.

**TLC** ($CH_2Cl_2$:MeOH, 95:5 v/v): $R_f$ = 0.62

**HR-MS (ESI):** m/z calculated for $[C_{42}H_{41}N_2O_8]^+$ ([M + $H^+$]): 701.2857, found: 701.2869 (-1.6 ppm).

**Melting point:** 233 – 258 °C (brown discoloration)

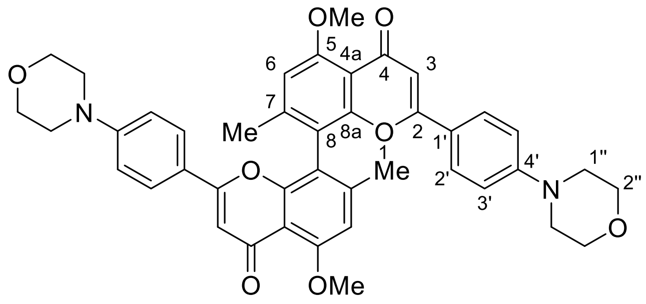


**17**

**1.2.9.11 *rac*-5,5'-dimethoxy-7,7'-dimethyl-2,2'-bis(4-(piperidin-1-yl)phenyl)-4H,4'H-[8,8'-bichromene]-4,4'-dione (18)**

The title compound was synthesized according to **GP1b** using BrettPhos (9.3 mg, 20 mol%, 20 µmol), (Pd G4)$_2$ (**26**) (3.8 mg, 5 mol%, 5.0 µmol) and piperidine (37.5 mg, 4.40 equiv, 0.440 mmol). The product was isolated as yellow solids in a yield of 56.5 mg (81.3 µmol, 81%).

**$^1$H NMR** (600 MHz, $CDCl_3$): δ 7.14 (d, *J* = 8.6 Hz, 4H, H-2'), 6.84 (s, 2H, H-6), 6.70 (d, *J* = 8.7 Hz, 4H, H-3'), 6.58 (s, 2H, H-3), 4.09 (s, 6H, OMe), 3.25 (t, *J* = 5.0 Hz, 8H, H-1''), 2.17 (s, 6H, Me), 1.61 (d, *J* = 10.1 Hz, 12H, H-2''+3'').

**$^{13}$C NMR** (151 MHz, $CDCl_3$): δ 178.8 (C-4), 161.6 (C-2), 159.1 (C-5), 155.7 (C-8a), 153.2 (C-4'), 144.3 (C-7), 127.1 (C-2'), 119.3 (C-1'), 116.6 (C-8), 114.6 (C-3'), 112.8 (C-4a), 108.1 (C-6), 105.9 (C-3), 56.6 (OMe), 48.9 (C-1''), 25.4 (C-2''), 24.4 (C-3''), 20.7 (Me).

**IR (ATR film) [cm$^{-1}$]**: 3477, 2930, 2847, 1633, 1603, 1516, 1383, 1336, 1234, 1199, 1061, 1024, 828.

**TLC** ($CH_2Cl_2$:MeOH, 95:5 v/v): $R_f$ = 0.61

**HR-MS (ESI):** m/z calculated for $[C_{44}H_{45}N_2O_6]^+$ ([M + H$^+$]): 697.3272, found: 697.3281 (-1.3 ppm).

**Melting point:** 195 – 208 °C (brown discoloration)

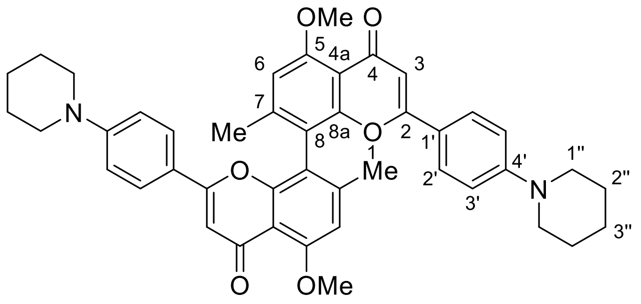

**18**

#### 1.2.9.12 *rac*-2,2'-bis(4-(azetidin-1-yl)phenyl)-5,5'-dimethoxy-7,7'-dimethyl-4H,4'H-[8,8'-bichromene]-4,4'-dione (19)

The title compound was synthesized according to **GP1b** using RuPhos (9.3 mg, 20 mol%, 20 µmol), (Pd G4)$_2$ (**26**) (3.8 mg, 5 mol%, 10.0 µmol) and azetidine (25.1 mg, 4.40 equiv, 0.440 mmol). The product was isolated as yellow solids in a yield of 50.0 mg (78.2 µmol, 78%).

**$^1$H NMR** (600 MHz, $CDCl_3$): δ 7.06 – 7.15 (m, 4H, H-2'), 6.84 (s, 2H, H-6), 6.56 (s, 2H, H-3), 6.18 – 6.25 (m, 4H, H-3'), 4.09 (s, 6H, OMe), 3.87 – 3.93 (m, 8H, H-1''), 2.36 (p, *J* = 7.3 Hz, 4H, H-2''), 2.16 (s, 6H, Me).

**$^{13}$C NMR** (151 MHz, $CDCl_3$): δ 178.8 (C-4), 161.9 (C-2), 159.0 (C-5), 155.7 (C-8a), 153.5 (C-4'), 144.3 (C-7), 126.9 (C-2'), 118.5 (C-1'), 116.6 (C-8), 112.8 (C-4a), 110.8 (C-3'), 108.0 (C-6), 105.5 (C-3), 56.6 (OMe), 51.9 (C-1''), 20.7 (Me), 16.7 (C-2'').

**IR (ATR film) [cm$^{-1}$]**: 3469, 2930, 2855, 2233, 1634, 1604, 1518, 1477, 1365, 1335, 1298, 1246, 1183, 1119, 1060, 907, 825, 724, 646.

**TLC** ($CH_2Cl_2$:MeOH, 96:4 v/v): $R_f$ = 0.38

**HR-MS (ESI):** m/z calculated for $[C_{40}H_{37}N_2O_6]^+$ ([M + H$^+$]): 641.2646, found: 641.2652 (-1.0 ppm).

**Melting point:** 275 °C (decomposition) (*rac*)

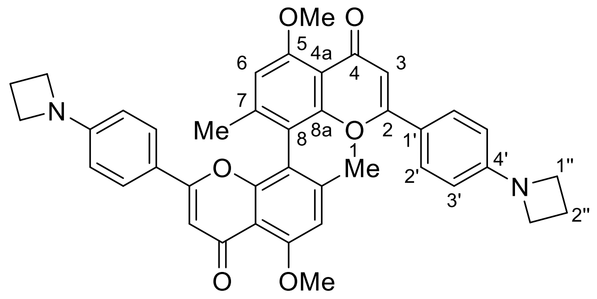


**19**

### 1.2.9.13 *rac*-2,2'-bis(4-(benzylamino)phenyl)-5,5'-dimethoxy-7,7'-dimethyl-4H,4'H-[8,8'-bichromene]-4,4'-dione (20)

The title compound was synthesized according to **GP1b** using RuPhos (9.3 mg, 20 mol%, 20 µmol), (Pd G4)$_2$ (**26**) (3.8 mg, 5 mol%, 5.0 µmol) and morpholine (47.1 mg, 4.40 equiv, 0.440 mmol). The product was isolated as yellow solids in a yield of 24.9 mg (33.7 µmol, 34%).

**$^1$H NMR** (600 MHz, $CDCl_3$): δ 7.27 – 7.37 (m, 10H, Ph), 7.11 (d, *J* = 8.6 Hz, 4H. H-2'), 6.83 (s, 2H, H-6), 6.56 (s, 2H, H-3), 6.47 (d, *J* = 8.5 Hz, 4H, H-3'), 4.33 (s, 4H, CH2), 4.07 (s, 6H, OMe), 2.15 (s, 6H, Me).

**$^{13}$C NMR** (151 MHz, $CDCl_3$): δ 178.8 (C-4), 161.7 (C-2), 159.1 (C-5), 155.7 (C-8a), 150.7 (C-4'), 144.4 (C-7), 138.4, 128.9, 127.7 (C-4''), 127.5, 127.4 (C-2'), 119.5 (C-1'), 116.6 (C-8), 112.8 (C-4a), 112.7 (C-3'), 108.1 (C-6), 105.7 (C-3), 56.7 (OMe), 47.8 ($CH_2$), 20.7 (Me).

**IR (ATR film) [cm$^{-1}$]**: 3321, 2929, 2847, 1632, 1603, 1591, 1525, 1370, 1332, 1251, 1183, 1120, 1060, 826, 733, 699.

**TLC** ($CH_2Cl_2$:MeOH, 95:5 v/v): $R_f$ = 0.64

**HR-MS (ESI):** m/z calculated for $[C_{48}H_{41}N_2O_6]^+$ ([M + H$^+$]): 741.2959, found: 741.2965 (-0.8 ppm).

**Melting point:** 286 – 290 °C (brown discoloration)

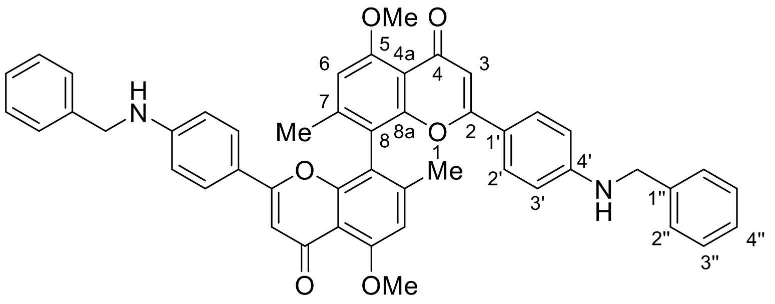


**20**

**1.2.9.14 *rac*-5,5'-dimethoxy-7,7'-dimethyl-2,2'-bis(4-(methylamino)phenyl)-4H,4'H-[8,8'-bichromene]-4,4'-dione (21)**

The title compound was synthesized according to **GP**1**a** using BrettPhos (10.7 mg, 20 mol%, 20.0 µmol), (Pd G4)$_2$ (**26**) (3.8 mg, 5 mol%, 5.0 µmol) and methylamine hydrochloride (25.1 mg, 4.40 equiv, 0.440 mmol). The product was isolated as yellow solids in a yield of 4.6 mg (7.81 µmol, 8%).

**$^1$H NMR** (600 MHz, $CDCl_3$): δ 7.05 – 7.18 (m, 4H, H-2'), 6.84 (s, 2H, H-6), 6.56 (s, 2H, H-3), 6.38 – 6.48 (m, 4H, H-3'), 4.09 (s, 6H, OMe), 2.82 (s, 6H, NH<u>*Me*</u>), 2.17 (s, 6H, Me).

**$^{13}$C NMR** (151 MHz, $CDCl_3$): δ 178.8 (C-4), 161.9 (C-2), 159.0 (C-5), 155.7 (C-8a), 151.8 (C-4'), 144.3 (C-7), 127.3 (C-2'), 118.9 (C-1'), 116.6 (C-8), 112.8 (C-4a), 112.2 (C-3'), 108.0 (C-6), 105.5 (C-3), 56.6 (OMe), 30.3 (NHMe), 20.7 (Me).

**IR (ATR film) [cm$^{-1}$]:** 3340, 2930, 2233, 1633, 1588, 1535, 1330, 1246, 1193, 1125, 1064, 829, 731.

**TLC** ($CH_2Cl_2$:MeOH, 96:4 v/v): $R_f$ = 0.19

**HR-MS (ESI):** m/z calculated for $[C_{36}H_{33}N_2O_6]^+$ ($[M + H^+]$): 589.2333, found: 589.2339 (-1.0 ppm).

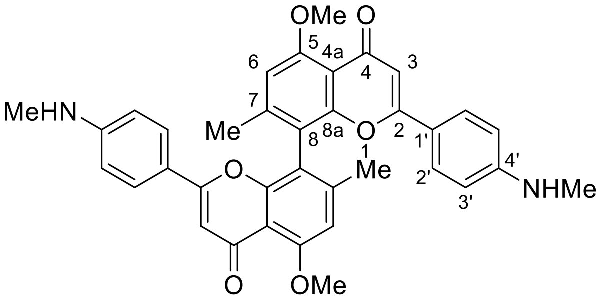


**21**

1.2.9.14.1 *rac*-2-(4-bromophenyl)-5,5'-dimethoxy-7,7'-dimethyl-2'-(4-(methylamino)phenyl)-4H,4'H-[8,8'-bichromene]-4,4'-dione (**22**)

The title compound was isolated as a side product during the synthesis of **21** as orange solids in a yield of 4.4 mg (7.46 µmol, 7%)

**$^1$H NMR** (600 MHz, $CDCl_3$): δ 7.23 (d, *J* = 8.9 Hz, 1H, H-2''), 7.13 (d, *J* = 8.8 Hz, 2H, H-2'''), 6.87 (s, 1H, H-6), 6.86 (s, 1H, H-6'), 6.78 (d, *J* = 8.9 Hz, 2H, H-3''), 6.63 (s, 1H, H-3), 6.58 (s, 1H, H-3'), 6.46 (d, *J* = 8.5 Hz, 2H, H-3'''), 4.10 (s, 3H, OMe/OMe'), 4.09 (s, 3H, OMe/OMe'), 3.78 (s, 3H, OMe''), 2.83 (s, 3H, NHMe), 2.18 (s, 3H, Me/Me'), 2.17 (s, 3H, Me/Me')

**$^{13}$C NMR** (151 MHz, $CDCl_3$): δ 178.8, 178.8 (C-4), 162.3, 161.8, 161.0, 159.1, 159.1, 155.7, 151.7, 151.6, 144.8, 144.3, 127.3, 127.3, 123.4, 116.7, 116.4, 114.6, 112.8, 112.4, 108.3, 108.1, 107.1, 105.5, 56.7, 56.7, 55.6 (OMe''), 30.4 (NHMe), 20.8, 20.7.

**IR (ATR film) [cm$^{-1}$]:** 3340, 2938, 2249, 1740, 1633, 1595, 1368, 1330, 1254, 1186, 1118, 1057, 829, 731.

**TLC** ($CH_2Cl_2$:MeOH, 96:4 v/v): $R_f$ = 0.25

**HR-MS (ESI):** m/z calculated for $[C_{36}H_{32}NO_7]^+$ ([M + H$^+$]): 590.2173, found: 590.2169 (0.7 ppm).

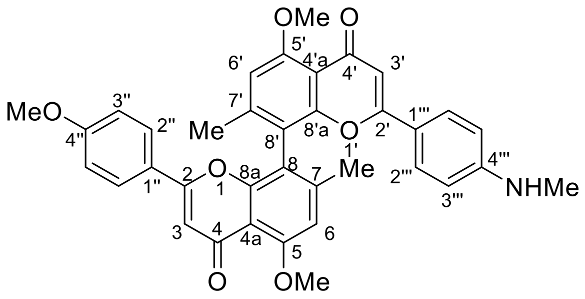


**22**

## 1.3 Pd-content

The palladium content of pharmaceutically relevant compounds is of great importance.[15-17] With the final step in our sequence being the Pd-catalyzed Buchwald Hartwig amination we sought to assess the Pd-content by an established protocol making use of a Tsuji-Trost type deallylation reaction.[18] This protocol was established in the late 2000s by the Koide group.[19,20] The compounds were suspended in aqua regia. Allyl Pittsburgh Green ether (APE) in combination with tris (2-furyl)phosphine were added as the reagent cocktail for the assessment. A calibration curve using a palladium standard solution 1 mg/mL Pd in 10% to 20% HCl (Thermo Scientific Lot #A0426720) gave good linearity for 0 – 3 ppm Pd-content ($R^2$ = 0.99). Measurements were performed in triplicates using Tecan reader infinite M1000 PRO.

Table S8:Palladium-content of biflavones subjected to biological testing with standard deviation.

| Compound | Pd-content [ppm] | SD [ppm] |
|---|---|---|
| **14** | <0.1 | 0.0 |
| **15** | 0.2 | 0.2 |
| **16** | <0.1 | 0.0 |
| **17** | <0.1 | 0.0 |
| **18** | <0.1 | 0.0 |
| **19** | 4.0 | 0.5 |
| **20** | 0.2 | 0.2 |
| (*R*)-**14** | 0.6 | 0.1 |
| (*S*)-**14** | <0.1 | 0.1 |
| **29** | <0.1 | 0.0 |
| **30** | <0.1 | 0.0 |

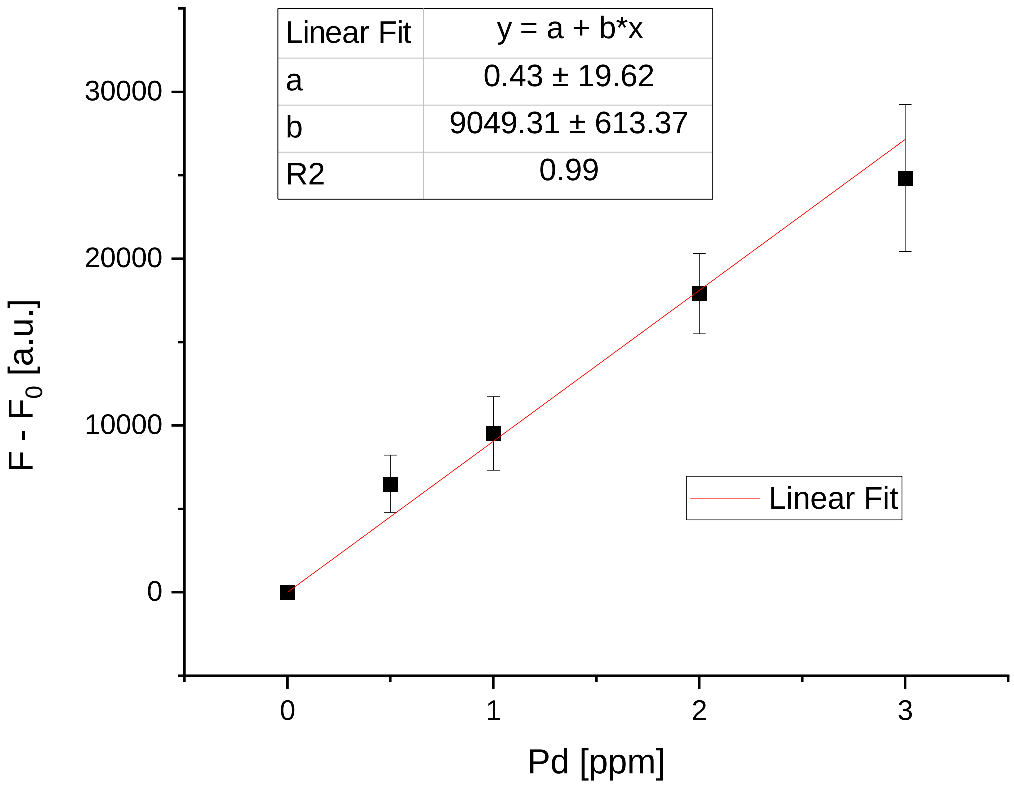


Figure S2: Calibration curve to determine Pd-content in the measured samples.

Overall, the palladium content of the content of the measured compounds was lower than 1 ppm except for biflavone **19**. Still, the obtained biological data did appear to be influenced by the Pd-content in our investigations **19** ($IC_{50\ HeLa}$ >50 µM).

## 1.4 Chiral HPLC Chromatograms

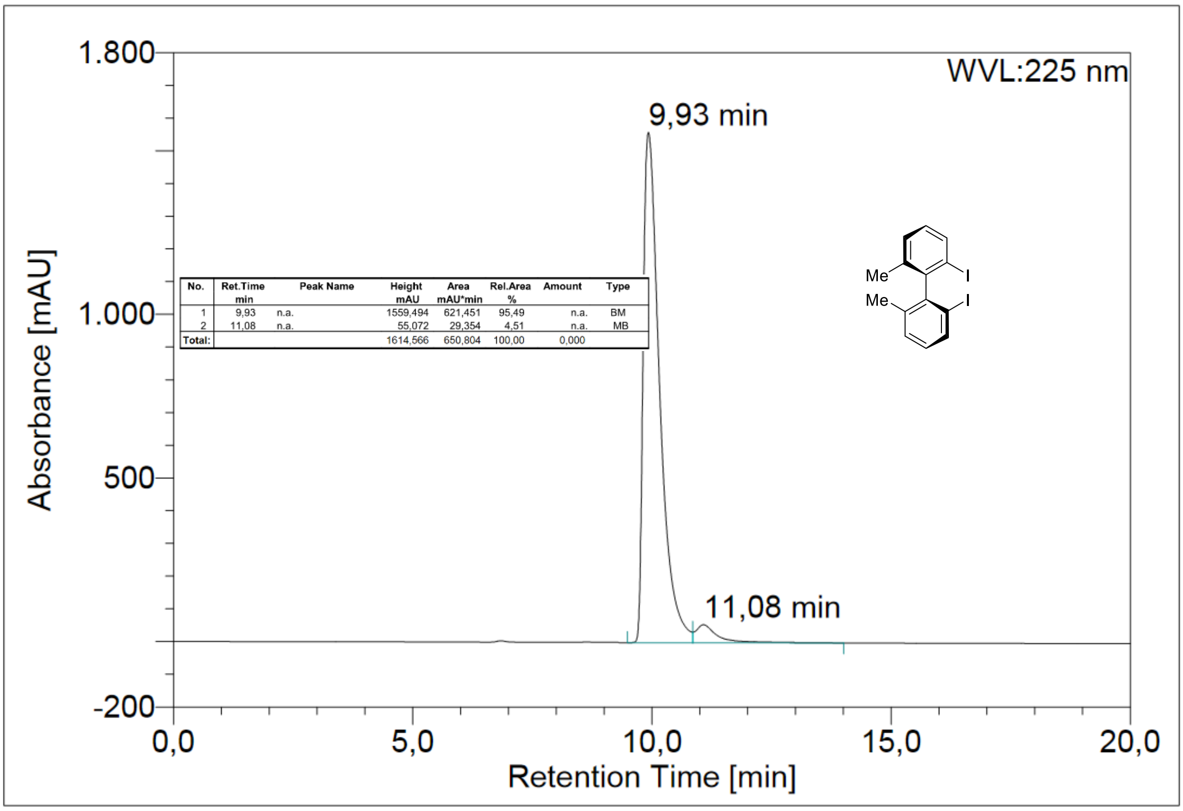


| No. | Ret.Time min | Peak Name | Height mAU | Area mAU*min | Rel.Area % | Amount | Type |
|---|---|---|---|---|---|---|---|
| 1 | 9,93 | n.a. | 1559,494 | 621,451 | 95,49 | n.a. | BM |
| 2 | 11,08 | n.a. | 55,072 | 29,354 | 4,51 | n.a. | MB |
| Total: | | | 1614,566 | 650,804 | 100,00 | 0,000 | |

Figure S3: HPLC Chromatogram of biaryl **10** 91%*ee* ($S_a$). Chiralpak® IC (Daciel) 250 ° 4.6 mm, 25 °C, 0.5 mL min$^{-1}$, 225 nm, *n*-heptane:*i*-PrOH 99.9:0.1 (v/v) $t_R$($S_a$) = 10.0 min, $t_R$($R_a$) = 11.1 min.

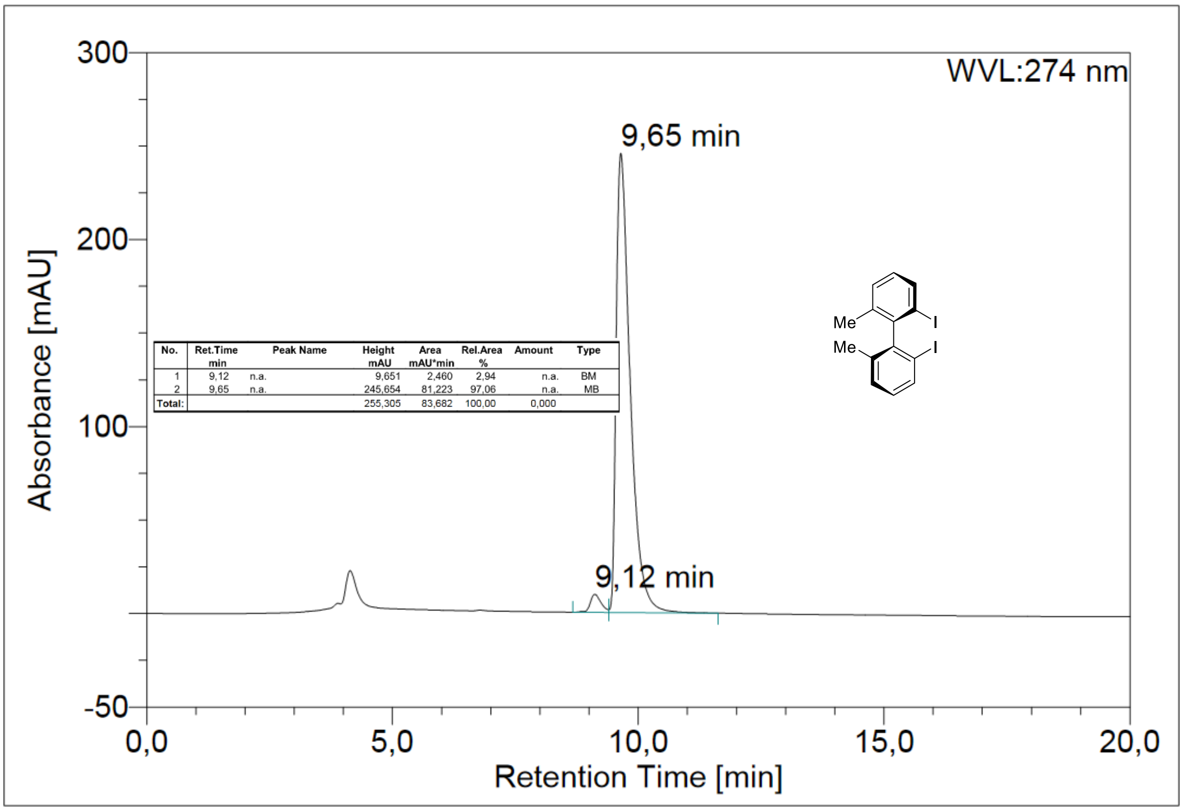


| No. | Ret.Time min | Peak Name | Height mAU | Area mAU*min | Rel.Area % | Amount | Type |
|---|---|---|---|---|---|---|---|
| 1 | 9,12 | n.a. | 9,651 | 2,460 | 2,94 | n.a. | BM |
| 2 | 9,65 | n.a. | 245,654 | 81,223 | 97,06 | n.a. | MB |
| Total: | | | 255,305 | 83,682 | 100,00 | 0,000 | |

Figure S4: HPLC Chromatogram of biaryl **10** 94%*ee* ($R_a$). Chiralpak® IC (Daciel) 250 ° 4.6 mm, 25 °C, 0.5 mL min$^{-1}$, 225 nm, *n*-heptane:*i*-PrOH 99.9:0.1 (v/v) $t_R$($S_a$) = 10.0 min, $t_R$($R_a$) = 11.1 min.

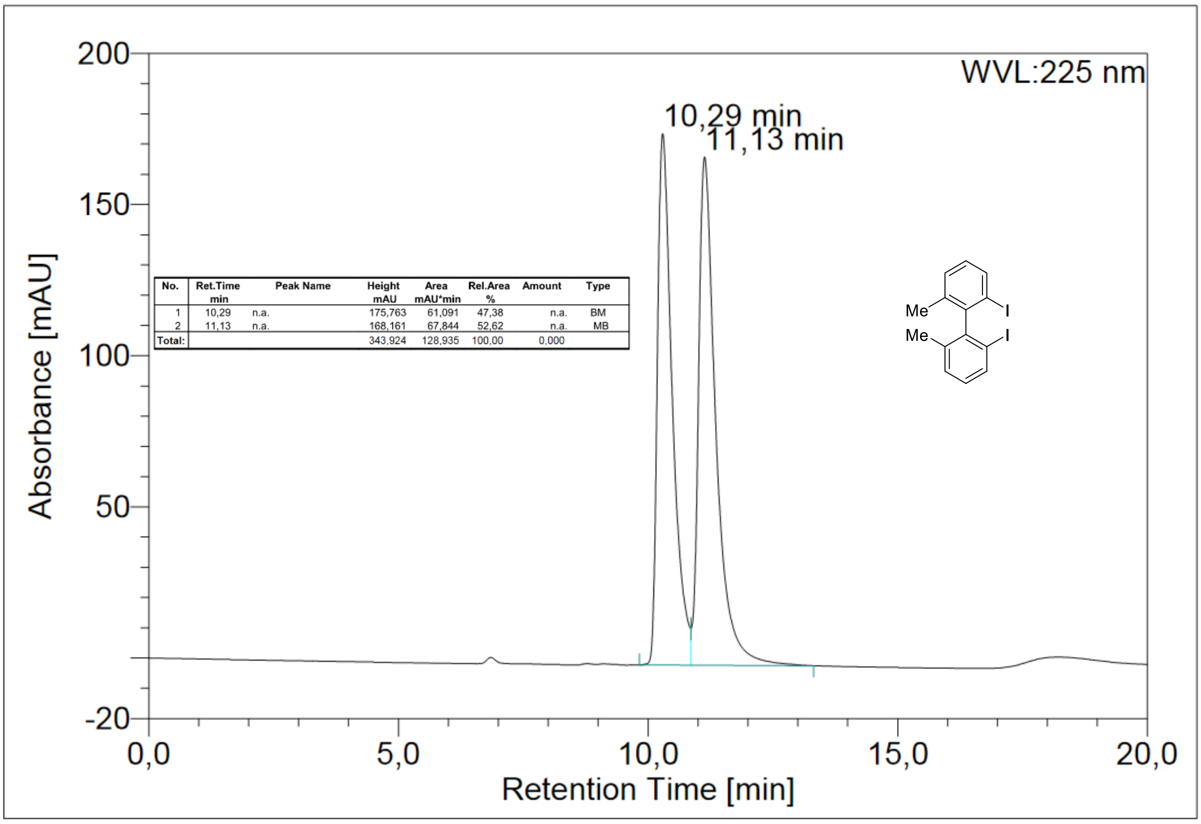


| No. | Ret.Time min | Peak Name | Height mAU | Area mAU*min | Rel.Area % | Amount | Type |
|---|---|---|---|---|---|---|---|
| 1 | 10,29 | n.a. | 175,763 | 61,091 | 47,38 | n.a. | BM |
| 2 | 11,13 | n.a. | 168,161 | 67,844 | 52,62 | n.a. | MB |
| Total: | | | 343,924 | 128,935 | 100,00 | 0,000 | |

Figure S5: HPLC Chromatogram of biaryl *rac*-**4**. Chiralpak® IC (Daciel) 250 ° 4.6 mm, 25 °C, 0.5 mL $min^{-1}$, 225 nm, *n*-heptane:*i*-PrOH 99.9:0.1 (v/v) $t_R$($S_a$) = 10.0 min, $t_R$($R_a$) = 11.1 min.

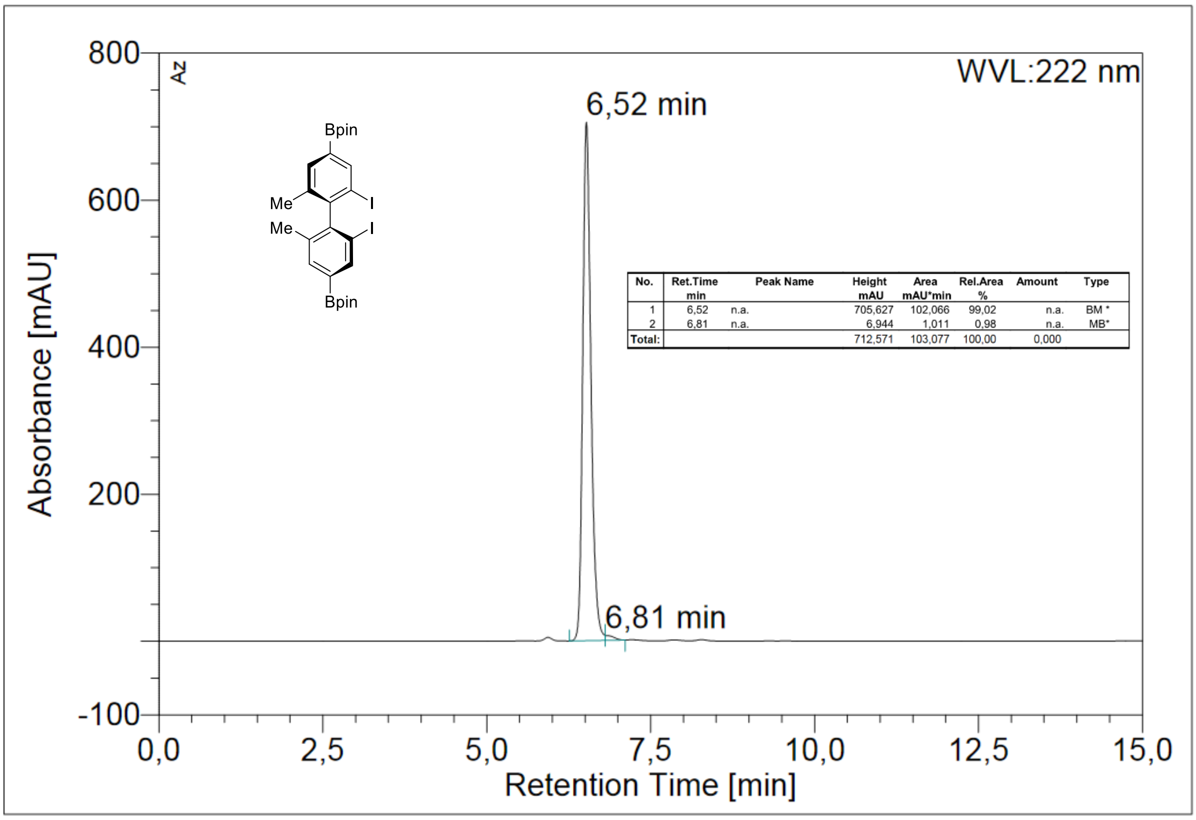


| No. | Ret.Time min | Peak Name | Height mAU | Area mAU*min | Rel.Area % | Amount | Type |
|---|---|---|---|---|---|---|---|
| 1 | 6,52 | n.a. | 705,627 | 102,066 | 99,02 | n.a. | BM * |
| 2 | 6,81 | n.a. | 6,944 | 1,011 | 0,98 | n.a. | MB* |
| Total: | | | 712,571 | 103,077 | 100,00 | 0,000 | |

Figure S6: HPLC Chromatogram of biaryl **9** 98%*ee* ($S_a$). Lux® Amylose-1 (Phenomenex) 250 ° 4.6 mm, 10 °C, 0.5 mL $min^{-1}$, 222 nm, *n*-heptane:*i*-PrOH 99:1 (v/v) $t_R$($S_a$) = 6.5 min, $t_R$($R_a$) = 6.8 min.

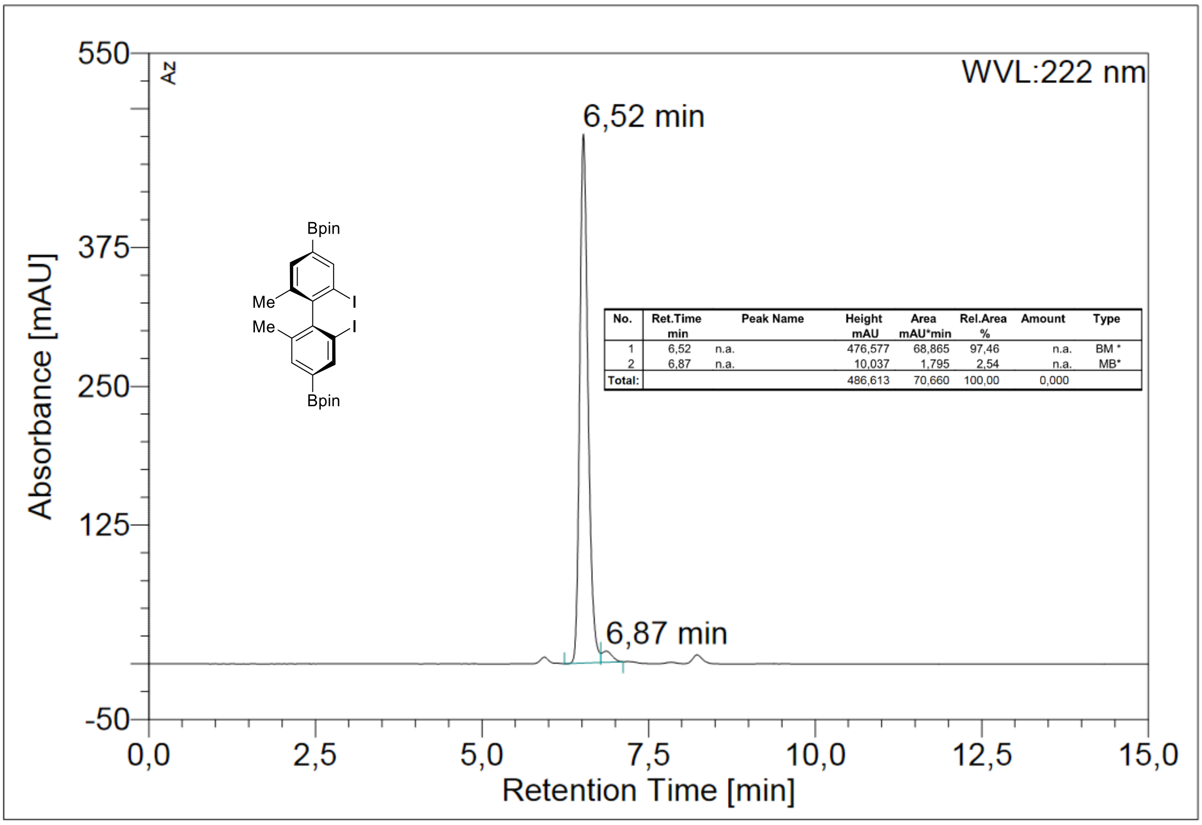


| No. | Ret.Time min | Peak Name | Height mAU | Area mAU*min | Rel.Area % | Amount | Type |
|---|---|---|---|---|---|---|---|
| 1 | 6,52 | n.a. | 476,577 | 68,865 | 97,46 | n.a. | BM * |
| 2 | 6,87 | n.a. | 10,037 | 1,795 | 2,54 | n.a. | MB* |
| Total: | | | 486,613 | 70,660 | 100,00 | 0,000 | |

Figure S7: HPLC Chromatogram of biaryl **9** 95%*ee* ($S_a$). Lux® Amylose-1 (Phenomenex) 250 ° 4.6 mm, 10 °C, 0.5 mL min$^{-1}$, 222 nm, *n*-heptane:*i*-PrOH 99:1 (v/v) $t_R(S_a)$ = 6.5 min, $t_R(R_a)$ = 6.8 min.

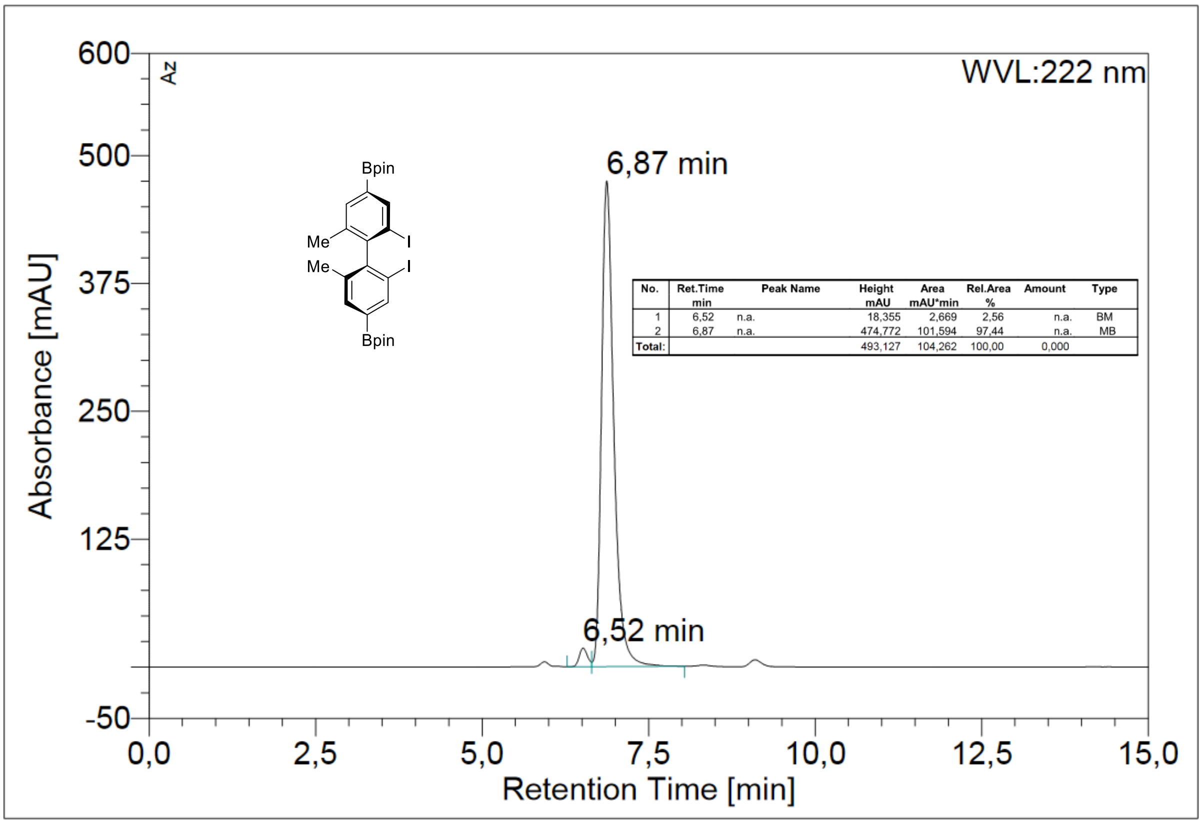


| No. | Ret.Time min | Peak Name | Height mAU | Area mAU*min | Rel.Area % | Amount | Type |
|---|---|---|---|---|---|---|---|
| 1 | 6,52 | n.a. | 18,355 | 2,669 | 2,56 | n.a. | BM |
| 2 | 6,87 | n.a. | 474,772 | 101,594 | 97,44 | n.a. | MB |
| Total: | | | 493,127 | 104,262 | 100,00 | 0,000 | |

Figure S8: HPLC Chromatogram of biaryl **9** 95%*ee* ($R_a$). Lux® Amylose-1 (Phenomenex) 250 ° 4.6 mm, 10 °C, 0.5 mL min$^{-1}$, 222 nm, *n*-heptane:*i*-PrOH 99:1 (v/v) $t_R(S_a)$ = 6.5 min, $t_R(R_a)$ = 6.8 min.

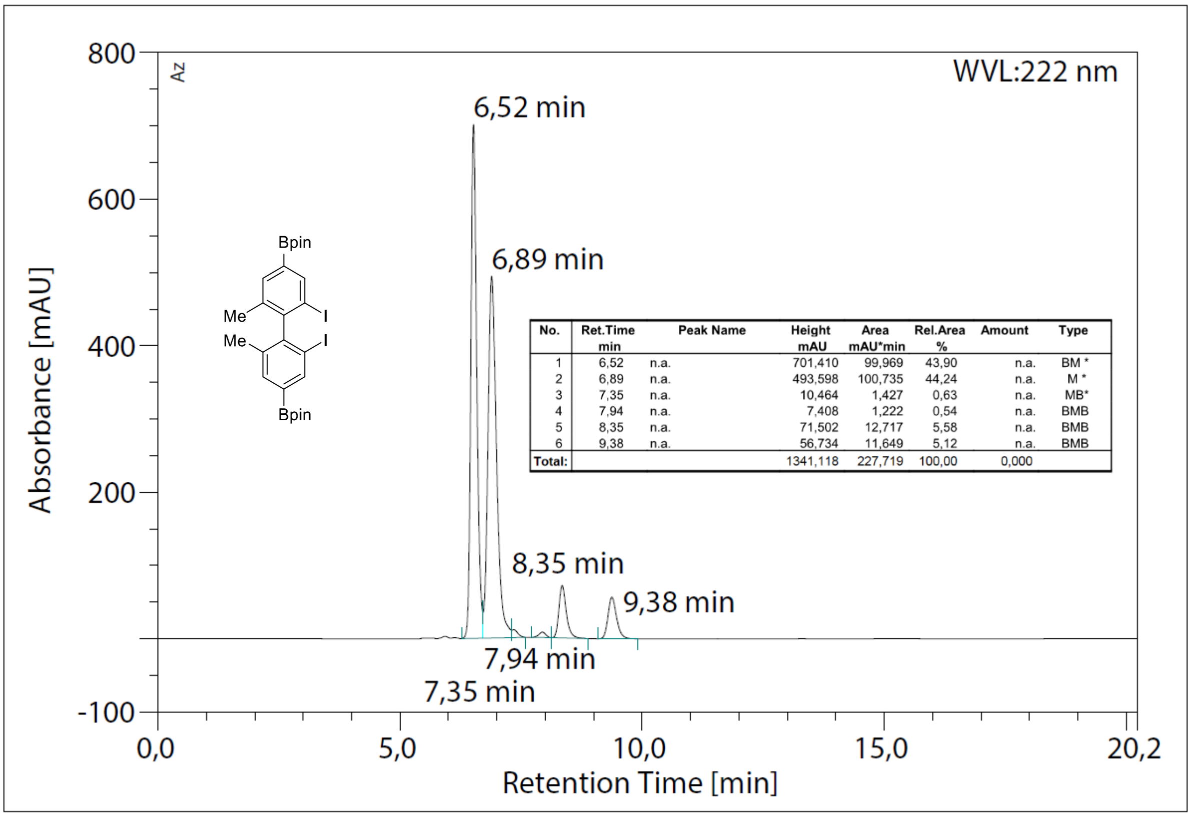


| No. | Ret.Time min | Peak Name | Height mAU | Area mAU*min | Rel.Area % | Amount | Type |
|---|---|---|---|---|---|---|---|
| 1 | 6,52 | n.a. | 701,410 | 99,969 | 43,90 | n.a. | BM * |
| 2 | 6,89 | n.a. | 493,598 | 100,735 | 44,24 | n.a. | M * |
| 3 | 7,35 | n.a. | 10,464 | 1,427 | 0,63 | n.a. | MB* |
| 4 | 7,94 | n.a. | 7,408 | 1,222 | 0,54 | n.a. | BMB |
| 5 | 8,35 | n.a. | 71,502 | 12,717 | 5,58 | n.a. | BMB |
| 6 | 9,38 | n.a. | 56,734 | 11,649 | 5,12 | n.a. | BMB |
| Total: | | | 1341,118 | 227,719 | 100,00 | 0,000 | |

Figure S9: HPLC Chromatogram of biaryl *rac*-**9**. Lux® Amylose-1 (Phenomenex) 250 ° 4.6 mm, 10 °C, 0.5 mL min$^{-1}$, 222 nm, *n*-heptane:*i*-PrOH 99:1 (v/v) $t_R$($S_a$) = 6.5 min, $t_R$($R_a$) = 6.8 min.

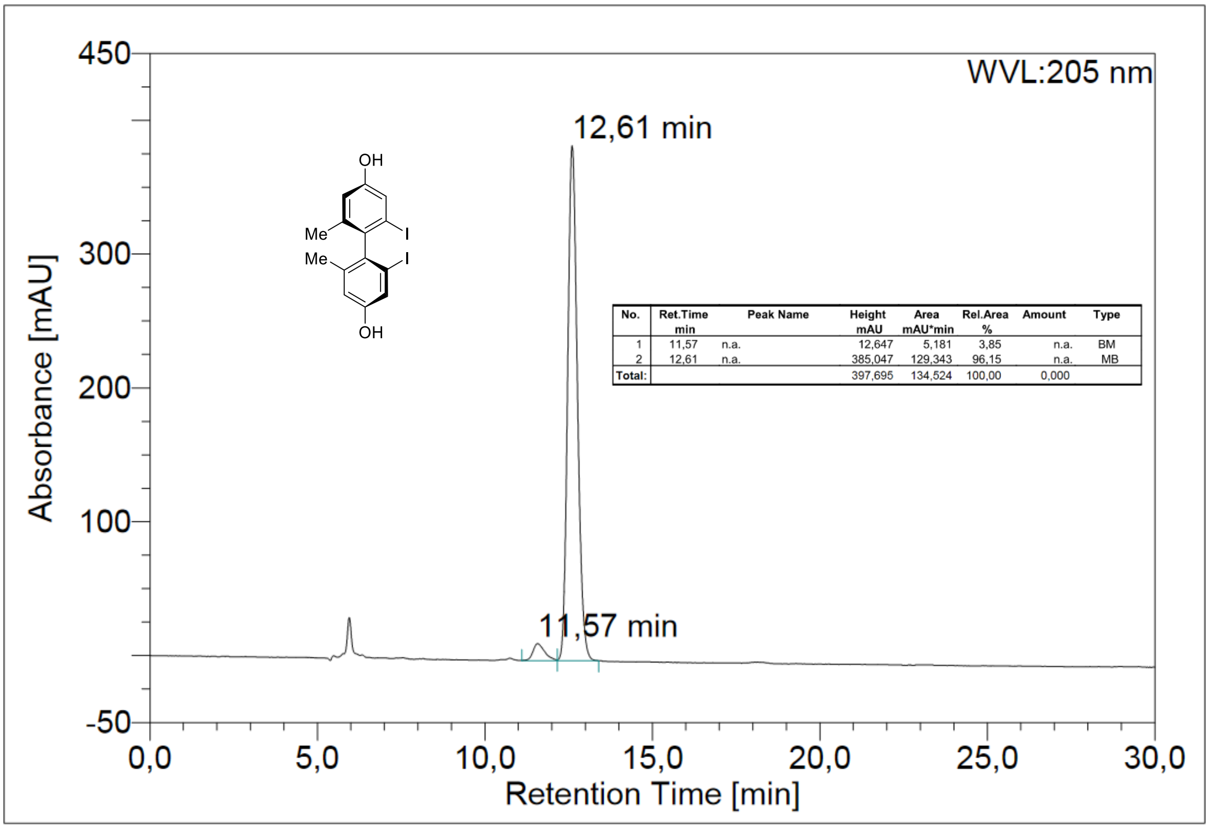


| No. | Ret.Time min | Peak Name | Height mAU | Area mAU*min | Rel.Area % | Amount | Type |
|---|---|---|---|---|---|---|---|
| 1 | 11,57 | n.a. | 12,647 | 5,181 | 3,85 | n.a. | BM |
| 2 | 12,61 | n.a. | 385,047 | 129,343 | 96,15 | n.a. | MB |
| Total: | | | 397,695 | 134,524 | 100,00 | 0,000 | |

Figure S10: HPLC Chromatogram of biaryl **10** 92%*ee* ($S_a$). Lux® Amylose-1 (Phenomenex) 250 ° 4.6 mm, 25 °C, 0.5 mL min$^{-1}$, 205 nm, *n*-heptane:*i*-PrOH 80:20 (v/v) $t_R$($S_a$) = 12.4 min, $t_R$($R_a$) = 11.3 min.

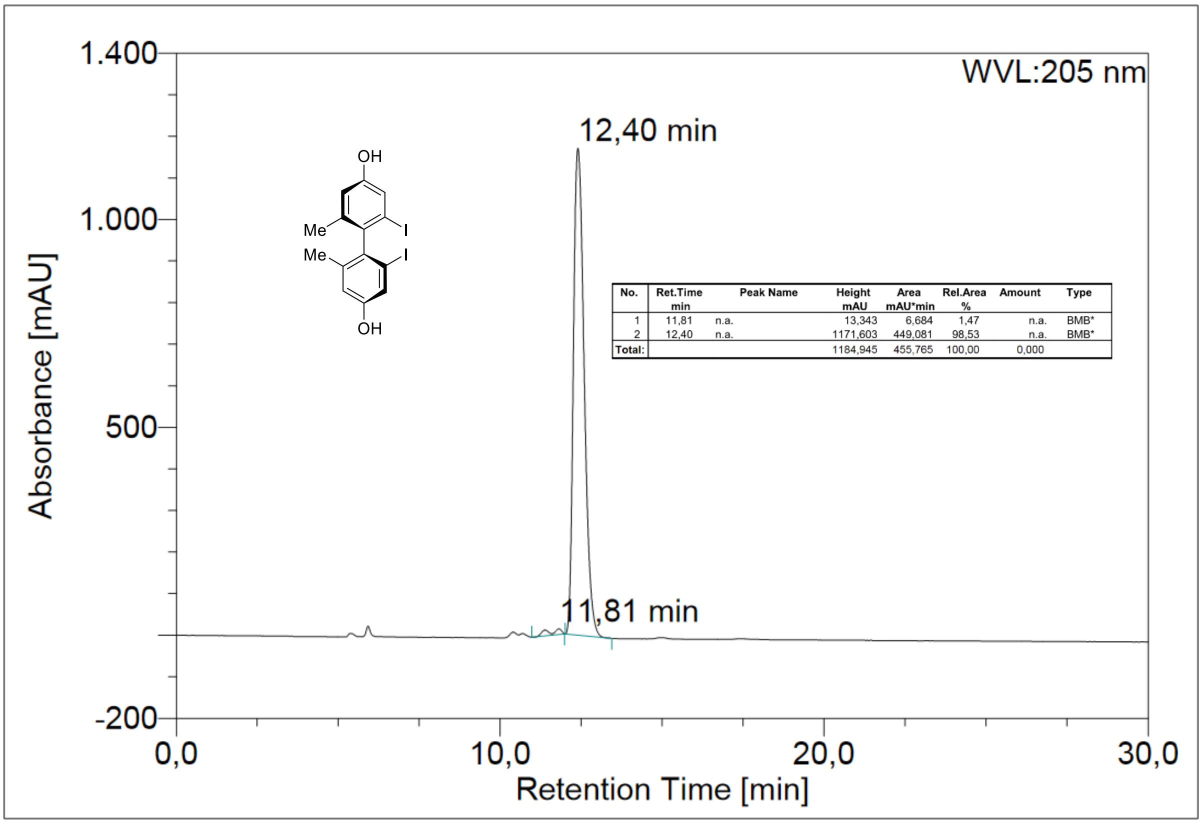


| No. | Ret.Time min | Peak Name | Height mAU | Area mAU*min | Rel.Area % | Amount | Type |
|---|---|---|---|---|---|---|---|
| 1 | 11,81 | n.a. | 13,343 | 6,684 | 1,47 | n.a. | BMB* |
| 2 | 12,40 | n.a. | 1171,603 | 449,081 | 98,53 | n.a. | BMB* |
| Total: | | | 1184,945 | 455,765 | 100,00 | 0,000 | |

Figure S11: HPLC Chromatogram of biaryl **10** 97%*ee* ($S_a$). Lux® Amylose-1 (Phenomenex) 250 ° 4.6 mm, 25 °C, 0.5 mL min$^{-1}$, 205 nm, *n*-heptane:*i*-PrOH 80:20 (v/v) $t_R(S_a)$ = 12.4 min, $t_R(R_a)$ = 11.3 min.

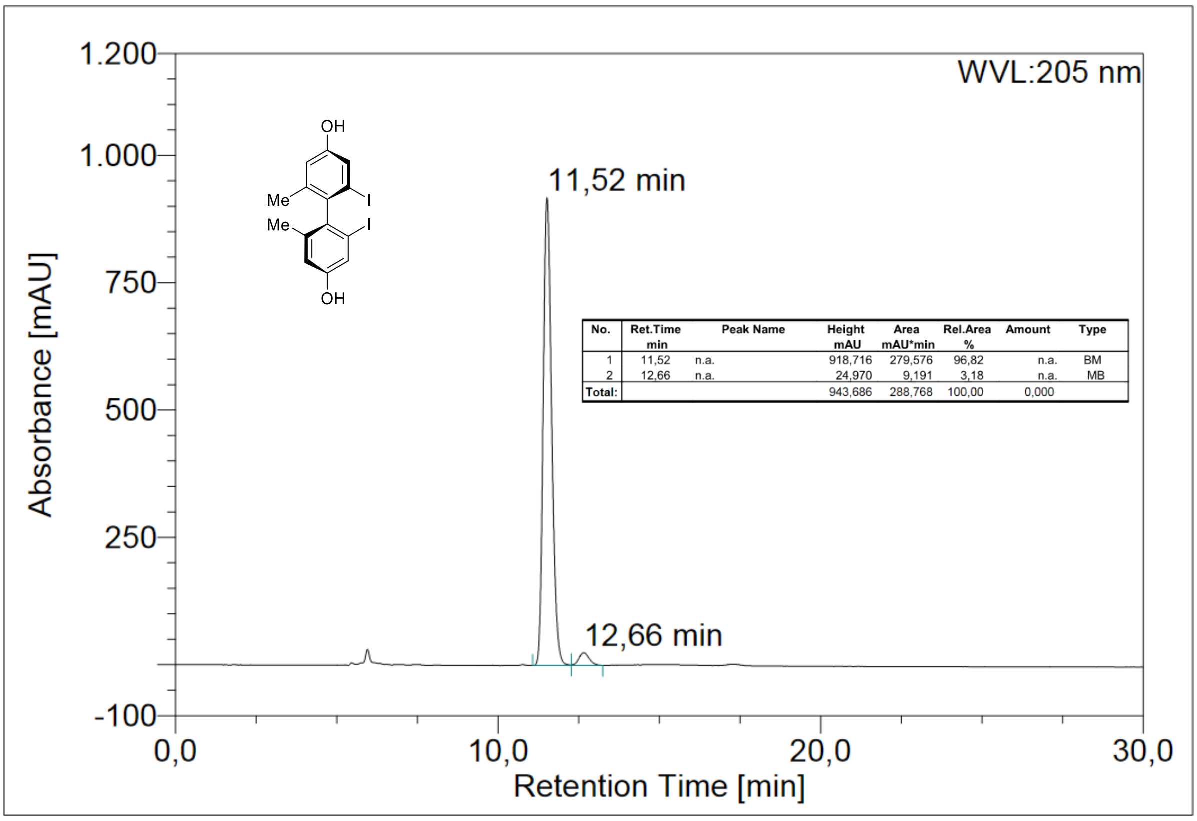


| No. | Ret.Time min | Peak Name | Height mAU | Area mAU*min | Rel.Area % | Amount | Type |
|---|---|---|---|---|---|---|---|
| 1 | 11,52 | n.a. | 918,716 | 279,576 | 96,82 | n.a. | BM |
| 2 | 12,66 | n.a. | 24,970 | 9,191 | 3,18 | n.a. | MB |
| Total: | | | 943,686 | 288,768 | 100,00 | 0,000 | |

Figure S12: HPLC Chromatogram of biaryl **10** 94%*ee* ($R_a$). Lux® Amylose-1 (Phenomenex) 250 ° 4.6 mm, 25 °C, 0.5 mL min$^{-1}$, 205 nm, *n*-heptane:*i*-PrOH 80:20 (v/v) $t_R(S_a)$ = 12.4 min, $t_R(R_a)$ = 11.3 min.

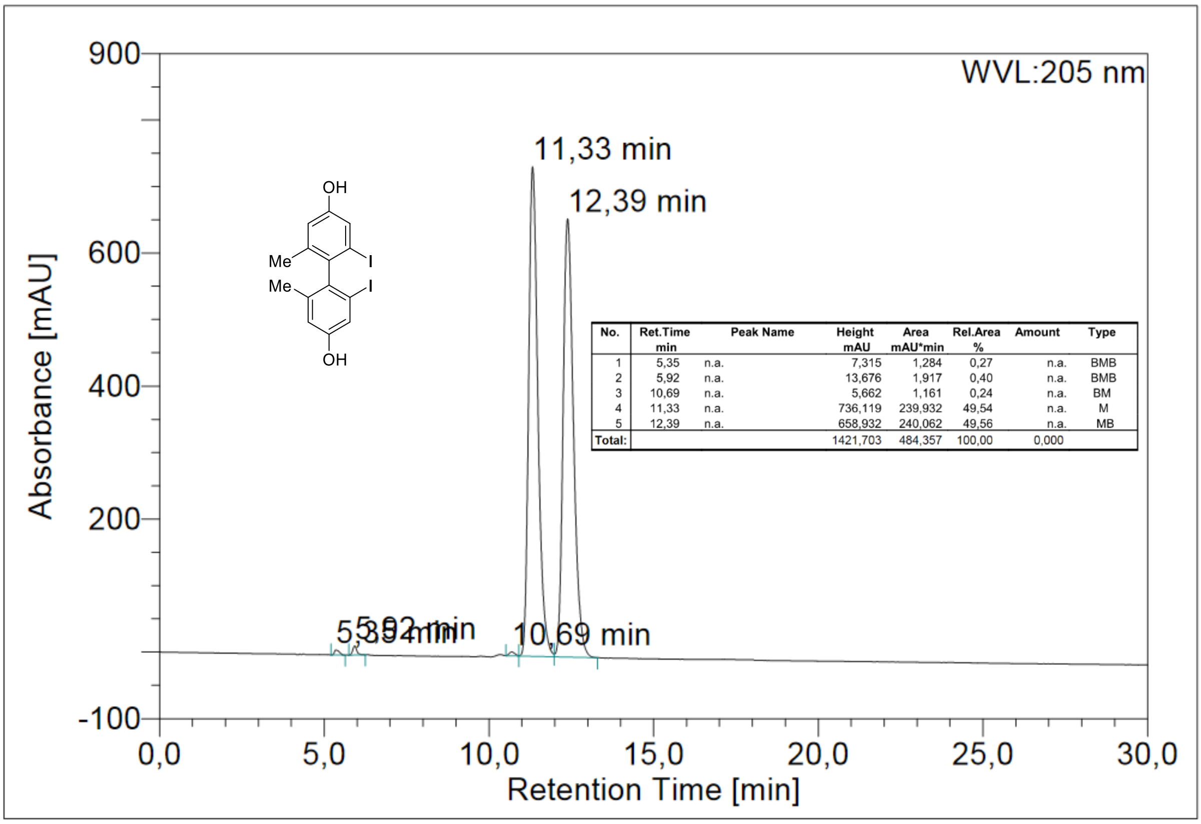


| No. | Ret.Time min | Peak Name | Height mAU | Area mAU*min | Rel.Area % | Amount | Type |
|---|---|---|---|---|---|---|---|
| 1 | 5,35 | n.a. | 7,315 | 1,284 | 0,27 | n.a. | BMB |
| 2 | 5,92 | n.a. | 13,676 | 1,917 | 0,40 | n.a. | BMB |
| 3 | 10,69 | n.a. | 5,662 | 1,161 | 0,24 | n.a. | BM |
| 4 | 11,33 | n.a. | 736,119 | 239,932 | 49,54 | n.a. | M |
| 5 | 12,39 | n.a. | 658,932 | 240,062 | 49,56 | n.a. | MB |
| Total: | | | 1421,703 | 484,357 | 100,00 | 0,000 | |

Figure S13: HPLC Chromatogram of biaryl *rac*-**10**. Lux® Amylose-1 (Phenomenex) 250 ° 4.6 mm, 25 °C, 0.5 mL min$^{-1}$, 205 nm, *n*-heptane:*i*-PrOH 80:20 (v/v) $t_R$(*S*a) = 12.4 min, $t_R$(*R*a) = 11.3 min.

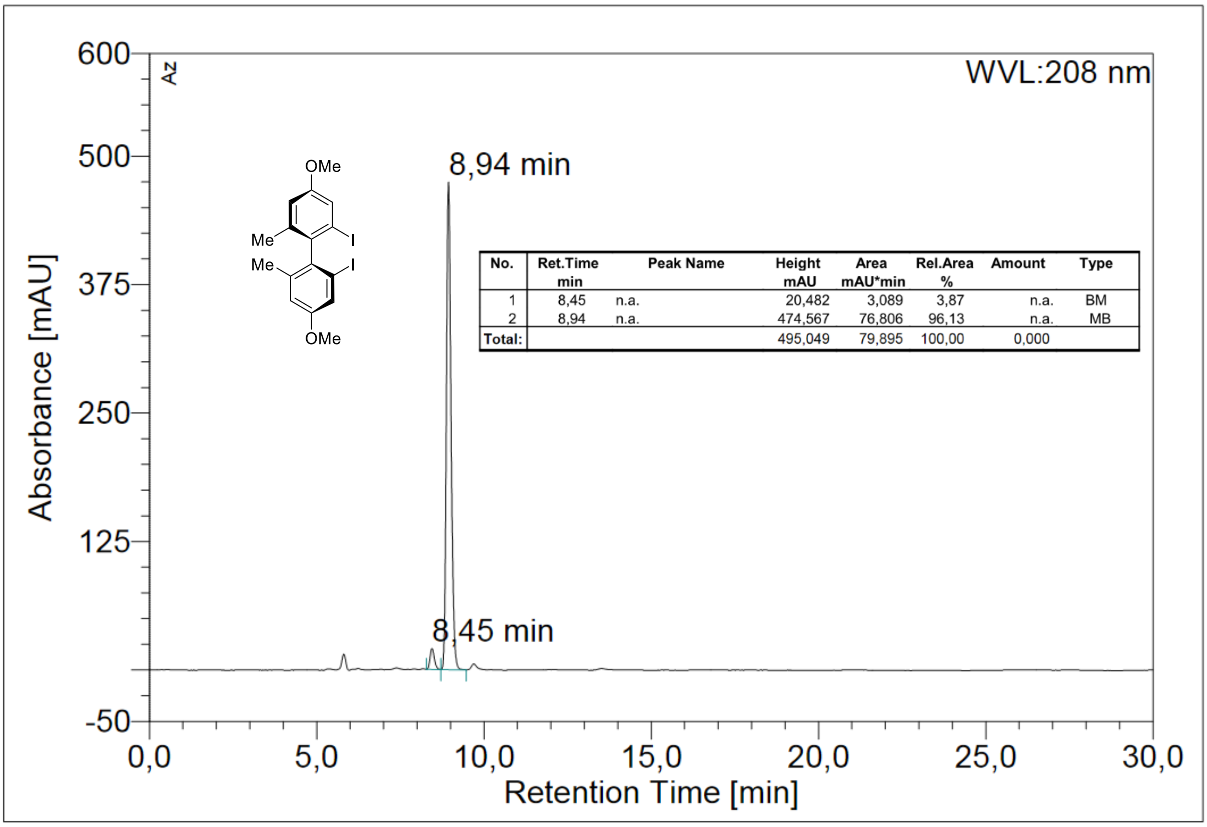


| No. | Ret.Time min | Peak Name | Height mAU | Area mAU*min | Rel.Area % | Amount | Type |
|---|---|---|---|---|---|---|---|
| 1 | 8,45 | n.a. | 20,482 | 3,089 | 3,87 | n.a. | BM |
| 2 | 8,94 | n.a. | 474,567 | 76,806 | 96,13 | n.a. | MB |
| Total: | | | 495,049 | 79,895 | 100,00 | 0,000 | |

Figure S14: HPLC Chromatogram of biaryl **11** 92%*ee* (*S*a). Chiralpak® IC (Daciel) 250 ° 4.6 mm, 25 °C, 0.5 mL min$^{-1}$, 208 nm, *n*-heptane:*i*-PrOH 99.9:0.1 (v/v) $t_R$(*S*a) = 8.9 min, $t_R$(*R*a) = 8.5 min.

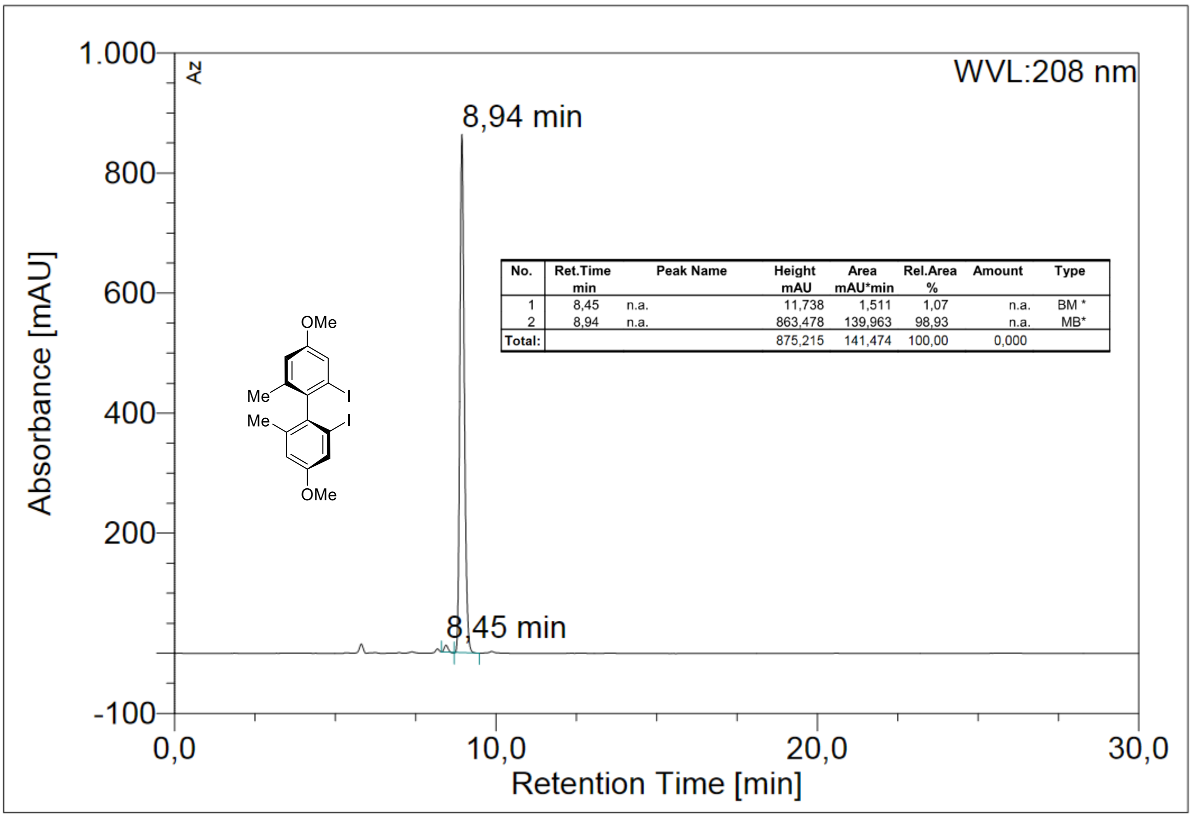


Figure S15: HPLC Chromatogram of biaryl **11** 98%*ee* ($S_a$). Chiralpak® IC (Daciel) 250 ° 4.6 mm, 25 °C, 0.5 mL min$^{-1}$, 208 nm, *n*-heptane:*i*-PrOH 99.9:0.1 (v/v) $t_R(S_a)$ = 8.9 min, $t_R(R_a)$ = 8.5 min.

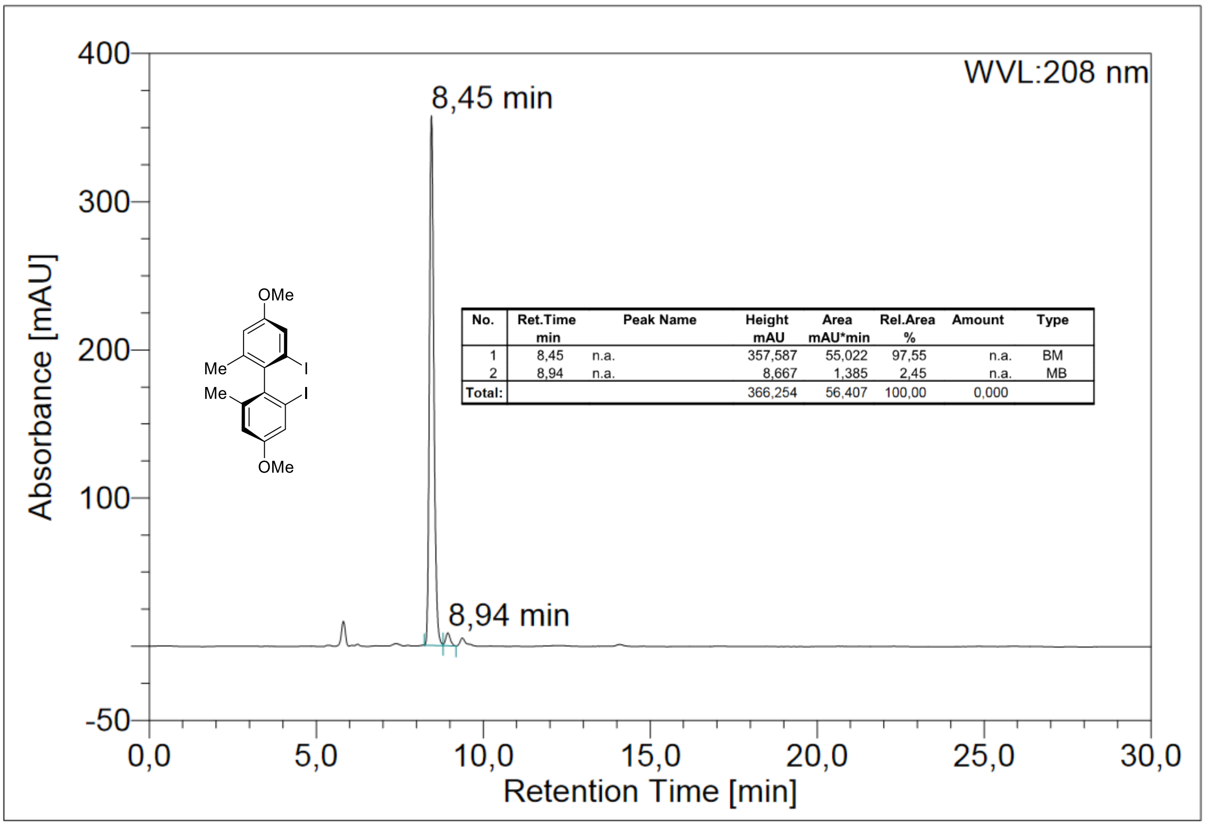


Figure S16: HPLC Chromatogram of biaryl **11** 95%*ee* ($R_a$). Chiralpak® IC (Daciel) 250 ° 4.6 mm, 25 °C, 0.5 mL min$^{-1}$, 208 nm, *n*-heptane:*i*-PrOH 99.9:0.1 (v/v) $t_R(S_a)$ = 8.9 min, $t_R(R_a)$ = 8.5 min.

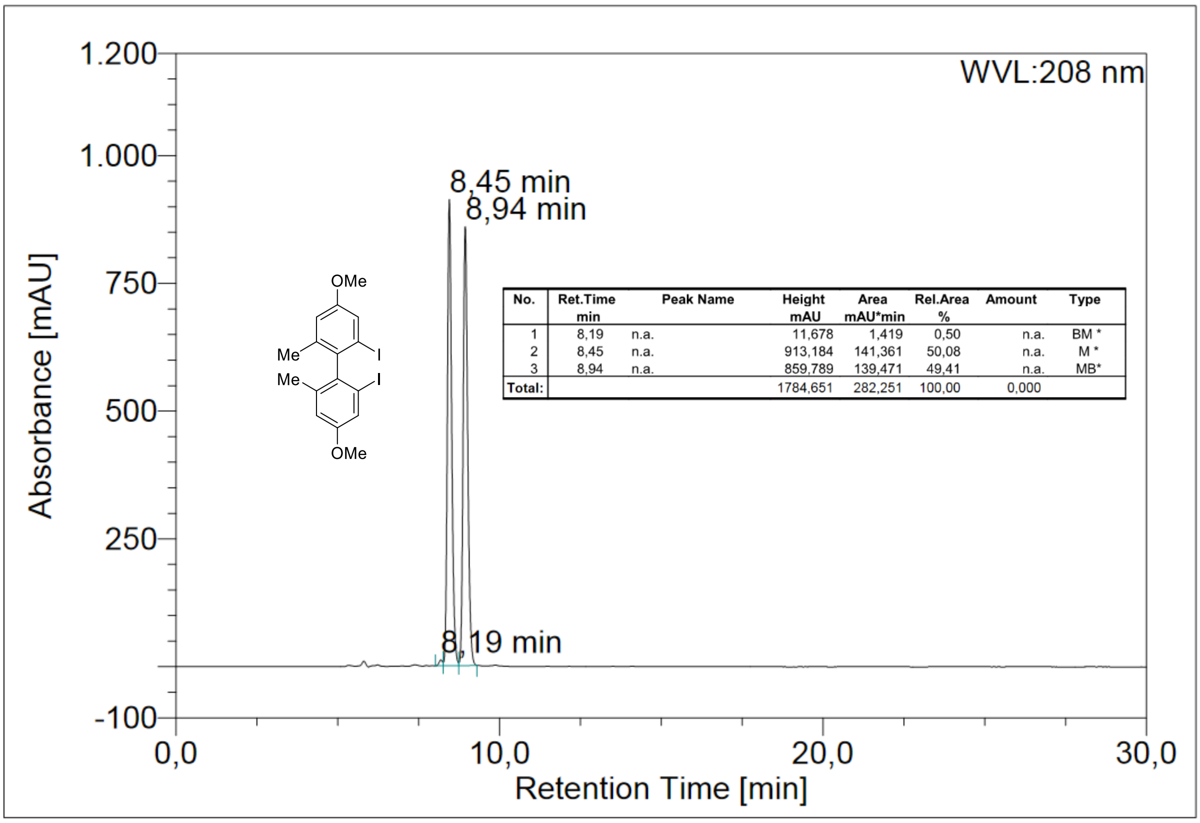


Figure S17: HPLC Chromatogram of biaryl *rac*-**11**. Chiralpak® IC (Daciel) 250 ° 4.6 mm, 25 °C, 0.5 mL min$^{-1}$, 208 nm, *n*-heptane:*i*-PrOH 99.9:0.1 (v/v) $t_R$($S_a$) = 8.9 min, $t_R$($R_a$) = 8.5 min.

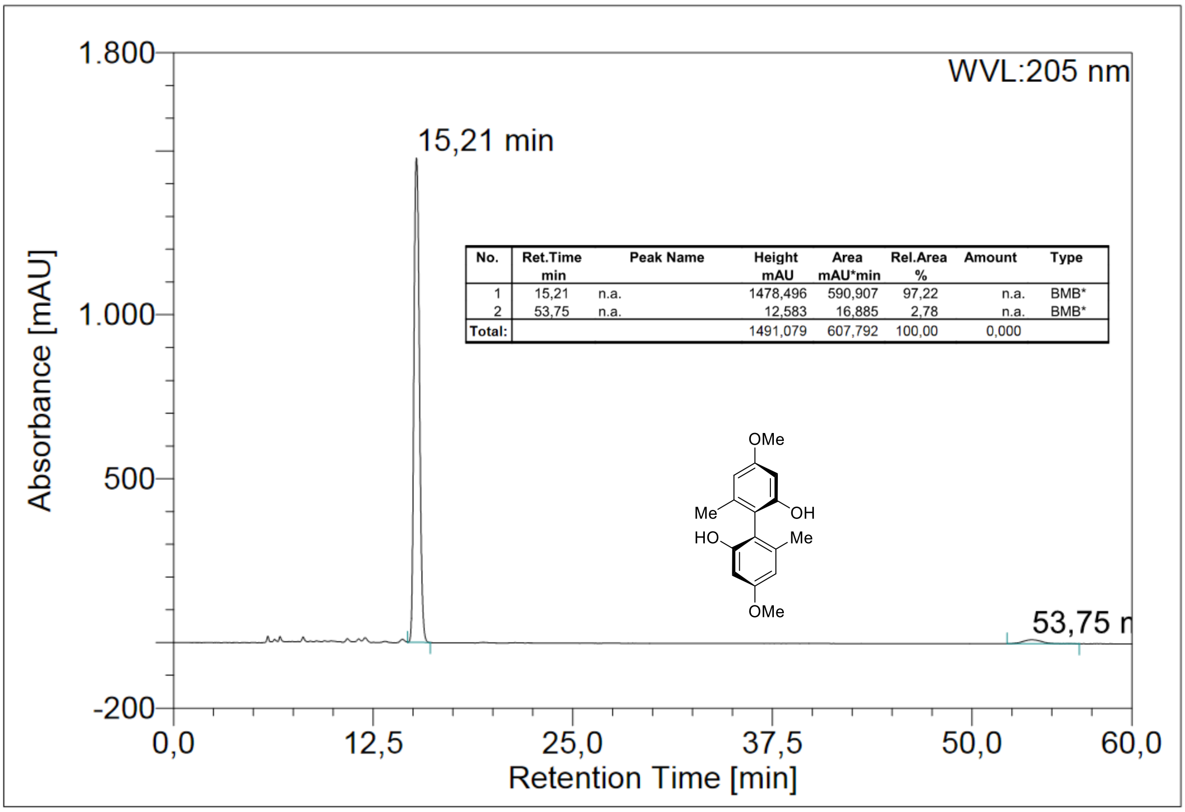


Figure S18: HPLC Chromatogram of biaryl **1** 94%*ee* ($S_a$). Lux® Amylose-1 (Phenomenex) 250 ° 4.6 mm, 25 °C, 0.5 mL min$^{-1}$, 205 nm, *n*-heptane:*i*-PrOH 50:50 (v/v) $t_R$($S_a$) = 14.5 min, $t_R$($R_a$) = 53.1 min.

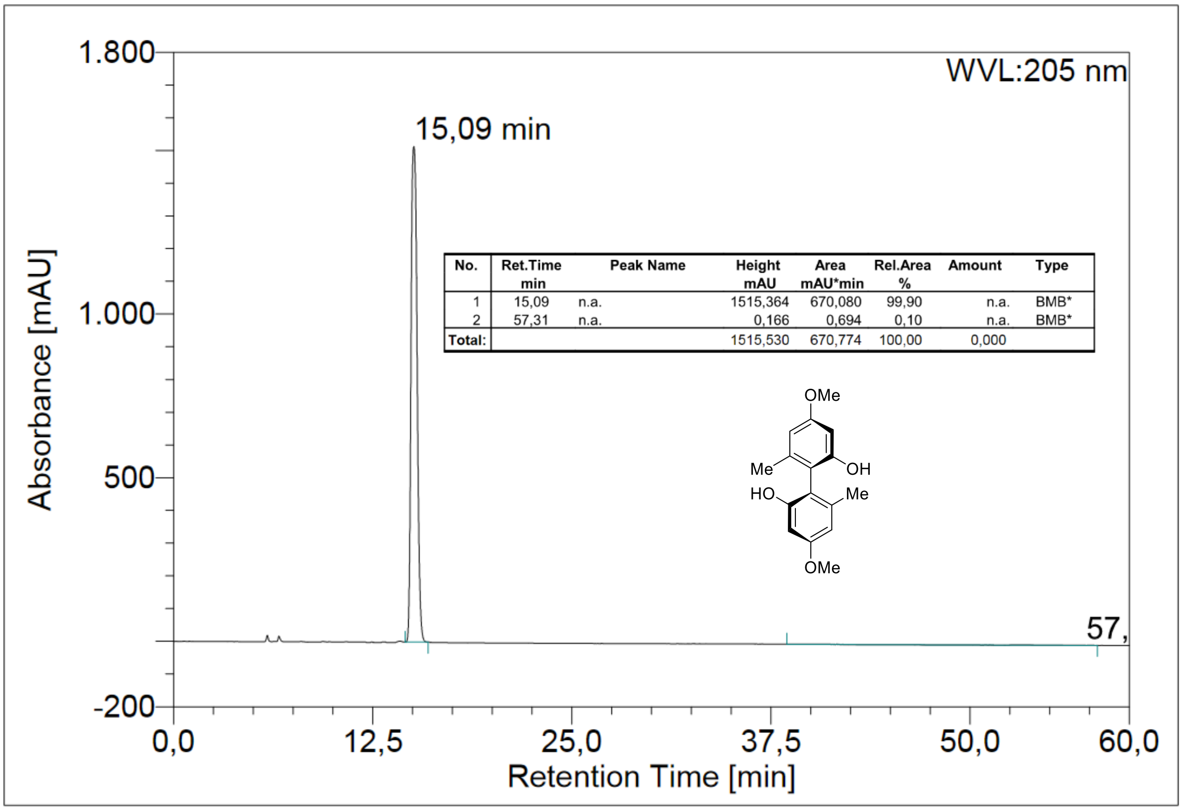


| No. | Ret.Time min | Peak Name | Height mAU | Area mAU*min | Rel.Area % | Amount | Type |
|---|---|---|---|---|---|---|---|
| 1 | 15,09 | n.a. | 1515,364 | 670,080 | 99,90 | n.a. | BMB* |
| 2 | 57,31 | n.a. | 0,166 | 0,694 | 0,10 | n.a. | BMB* |
| Total: | | | 1515,530 | 670,774 | 100,00 | 0,000 | |

Figure S19: HPLC Chromatogram of biaryl **1** >99%*ee* ($S_a$). Lux® Amylose-1 (Phenomenex) 250 ° 4.6 mm, 25 °C, 0.5 mL $min^{-1}$, 205 nm, *n*-heptane:*i*-PrOH 50:50 (v/v) $t_R(S_a)$ = 14.5 min, $t_R(R_a)$ = 53.1 min.

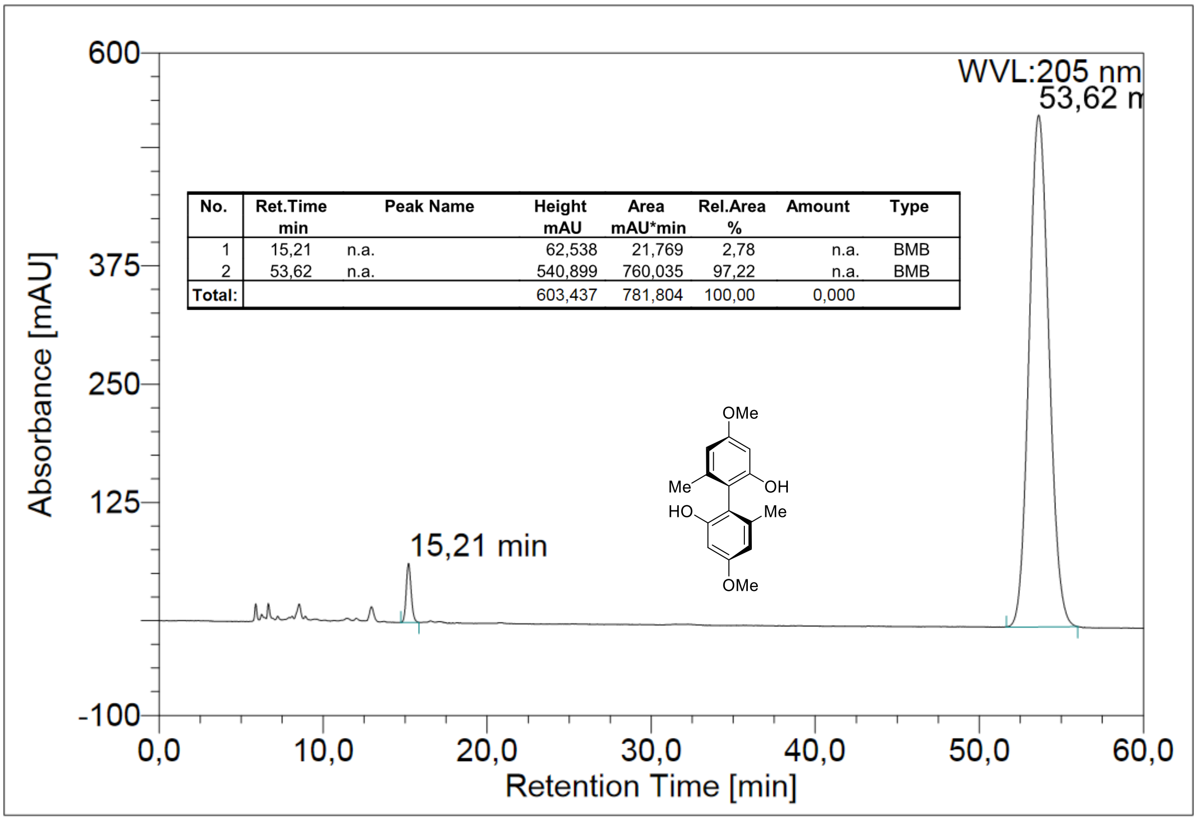


| No. | Ret.Time min | Peak Name | Height mAU | Area mAU*min | Rel.Area % | Amount | Type |
|---|---|---|---|---|---|---|---|
| 1 | 15,21 | n.a. | 62,538 | 21,769 | 2,78 | n.a. | BMB |
| 2 | 53,62 | n.a. | 540,899 | 760,035 | 97,22 | n.a. | BMB |
| Total: | | | 603,437 | 781,804 | 100,00 | 0,000 | |

Figure S20: HPLC Chromatogram of biaryl **1** 94%*ee* ($R_a$). Lux® Amylose-1 (Phenomenex) 250 ° 4.6 mm, 25 °C, 0.5 mL $min^{-1}$, 205 nm, *n*-heptane:*i*-PrOH 50:50 (v/v) $t_R(S_a)$ = 14.5 min, $t_R(R_a)$ = 53.1 min.

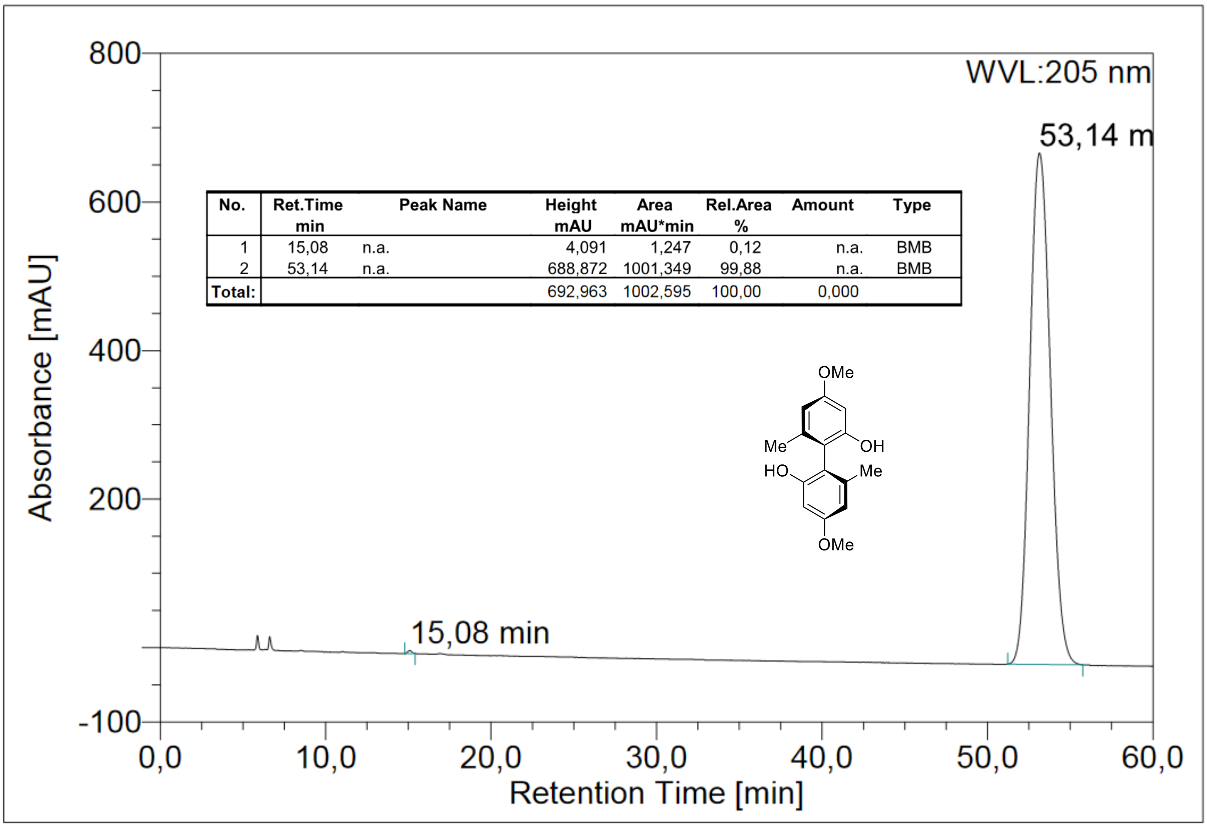


| No. | Ret.Time min | Peak Name | Height mAU | Area mAU*min | Rel.Area % | Amount | Type |
|---|---|---|---|---|---|---|---|
| 1 | 15,08 | n.a. | 4,091 | 1,247 | 0,12 | n.a. | BMB |
| 2 | 53,14 | n.a. | 688,872 | 1001,349 | 99,88 | n.a. | BMB |
| Total: | | | 692,963 | 1002,595 | 100,00 | 0,000 | |

Figure S21: HPLC Chromatogram of biaryl **1** >99%*ee* (*R*a). Lux® Amylose-1 (Phenomenex) 250 ° 4.6 mm, 25 °C, 0.5 mL min$^{-1}$, 205 nm, *n*-heptane:*i*-PrOH 50:50 (v/v) $t_R(S_a)$ = 14.5 min, $t_R(R_a)$ = 53.1 min.

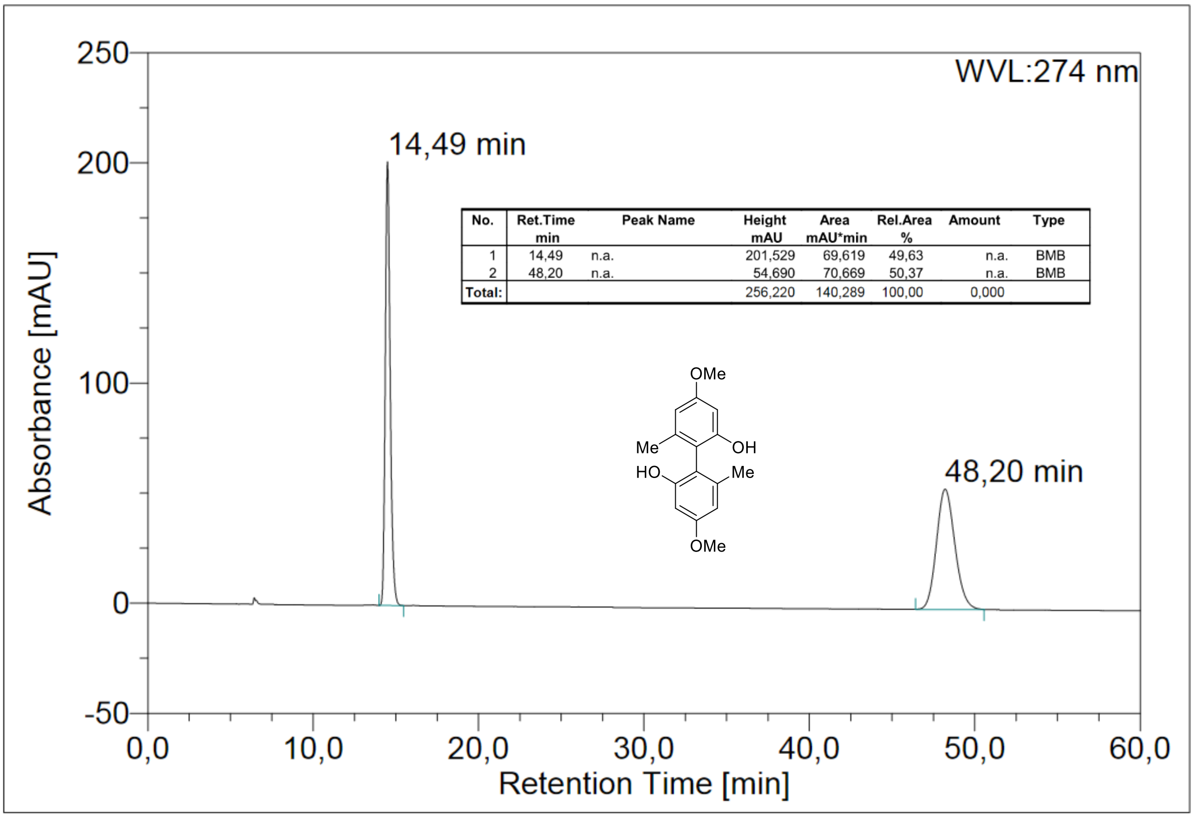


| No. | Ret.Time min | Peak Name | Height mAU | Area mAU*min | Rel.Area % | Amount | Type |
|---|---|---|---|---|---|---|---|
| 1 | 14,49 | n.a. | 201,529 | 69,619 | 49,63 | n.a. | BMB |
| 2 | 48,20 | n.a. | 54,690 | 70,669 | 50,37 | n.a. | BMB |
| Total: | | | 256,220 | 140,289 | 100,00 | 0,000 | |

Figure S22: HPLC Chromatogram of biaryl *rac*-**1**. Lux® Amylose-1 (Phenomenex) 250 ° 4.6 mm, 25 °C, 0.5 mL min$^{-1}$, 205 nm, *n*-heptane:*i*-PrOH 50:50 (v/v) $t_R(S_a)$ = 14.5 min, $t_R(R_a)$ = 53.1 min.

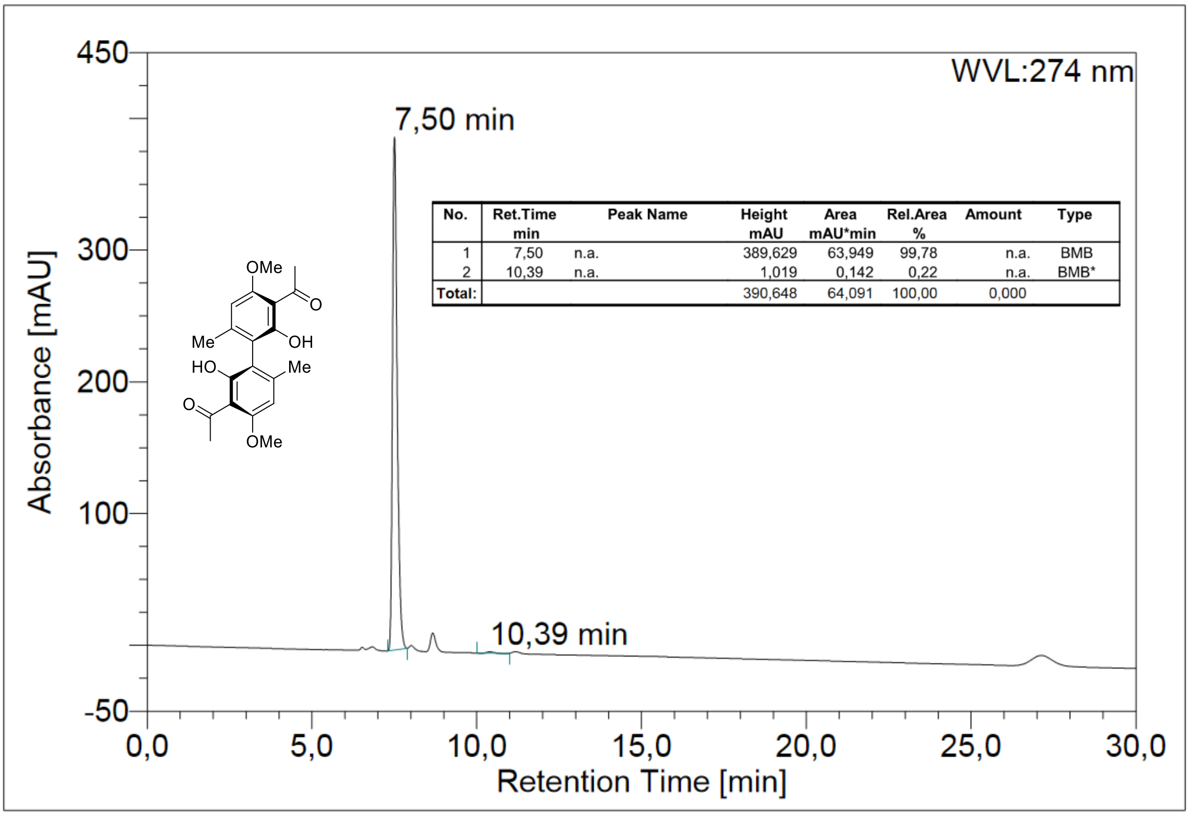


| No. | Ret.Time min | Peak Name | Height mAU | Area mAU*min | Rel.Area % | Amount | Type |
|---|---|---|---|---|---|---|---|
| 1 | 7,50 | n.a. | 389,629 | 63,949 | 99,78 | n.a. | BMB |
| 2 | 10,39 | n.a. | 1,019 | 0,142 | 0,22 | n.a. | BMB* |
| Total: | | | 390,648 | 64,091 | 100,00 | 0,000 | |

Figure S23: HPLC Chromatogram of biaryl **12** >99%*ee* ($S_a$). Lux® Amylose-1 (Phenomenex) 250 ° 4.6 mm, 25 °C, 0.5 mL $min^{-1}$, 274 nm, *n*-heptane:*i*-PrOH 50:50 (v/v) $t_R(S_a)$ = 7.5 min, $t_R(R_a)$ = 10.4 min.

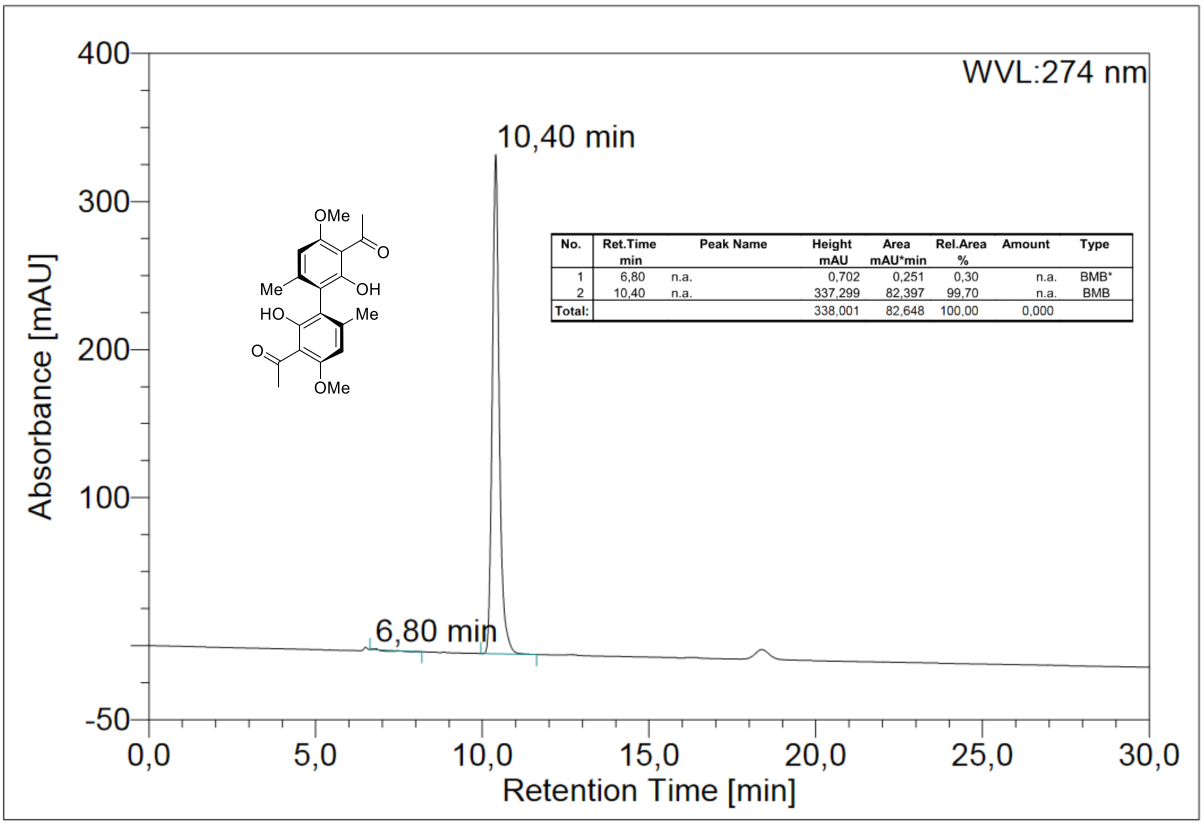


| No. | Ret.Time min | Peak Name | Height mAU | Area mAU*min | Rel.Area % | Amount | Type |
|---|---|---|---|---|---|---|---|
| 1 | 6,80 | n.a. | 0,702 | 0,251 | 0,30 | n.a. | BMB* |
| 2 | 10,40 | n.a. | 337,299 | 82,397 | 99,70 | n.a. | BMB |
| Total: | | | 338,001 | 82,648 | 100,00 | 0,000 | |

Figure S24: HPLC Chromatogram of biaryl **12** >99%*ee* ($R_a$). Lux® Amylose-1 (Phenomenex) 250 ° 4.6 mm, 25 °C, 0.5 mL $min^{-1}$, 274 nm, *n*-heptane:*i*-PrOH 50:50 (v/v) $t_R(S_a)$ = 7.5 min, $t_R(R_a)$ = 10.4 min

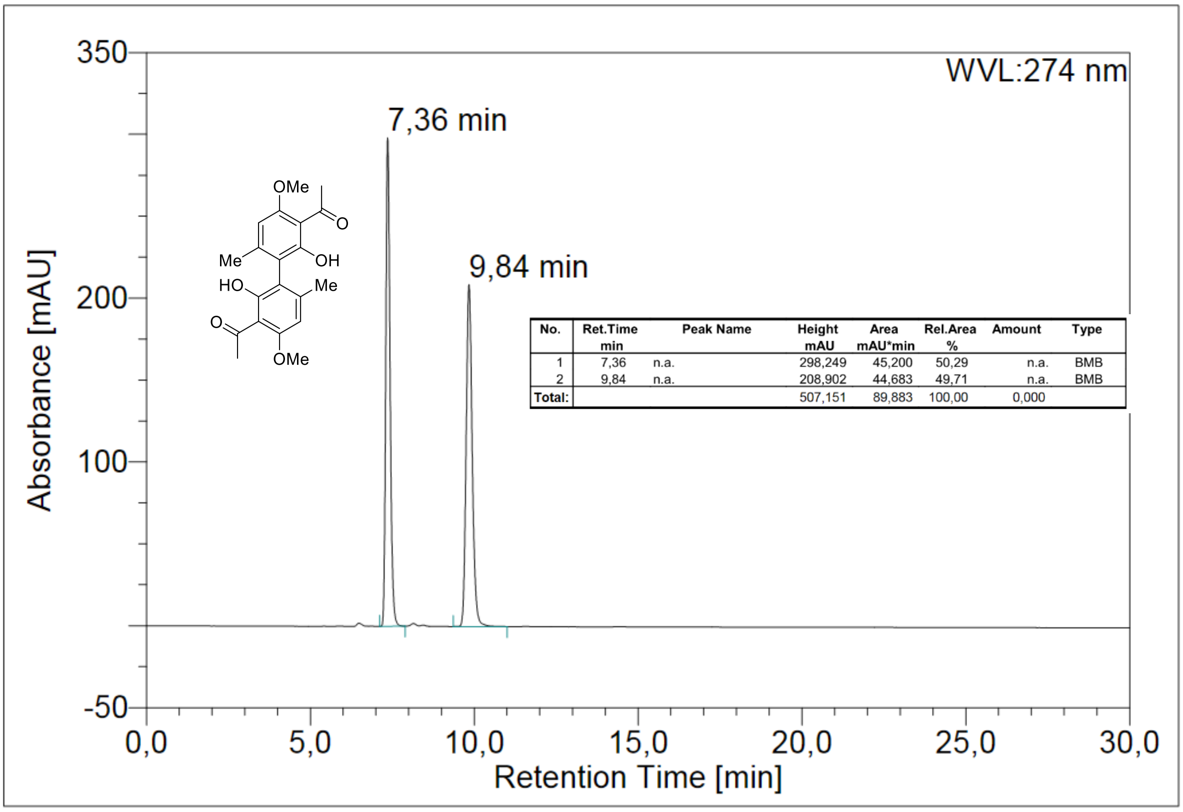

| No. | Ret.Time min | Peak Name | Height mAU | Area mAU*min | Rel.Area % | Amount | Type |
|---|---|---|---|---|---|---|---|
| 1 | 7,36 | n.a. | 298,249 | 45,200 | 50,29 | n.a. | BMB |
| 2 | 9,84 | n.a. | 208,902 | 44,683 | 49,71 | n.a. | BMB |
| Total: | | | 507,151 | 89,883 | 100,00 | 0,000 | |



.

Figure S25: HPLC Chromatogram of biaryl *rac*-**12** Lux® Amylose-1 (Phenomenex) 250 ° 4.6 mm, 25 °C, 0.5 mL min$^{-1}$, 274 nm, *n*-heptane:*i*-PrOH 50:50 (v/v) $t_R(S_a)$ = 7.5 min, $t_R(R_a)$ = 10.4 min

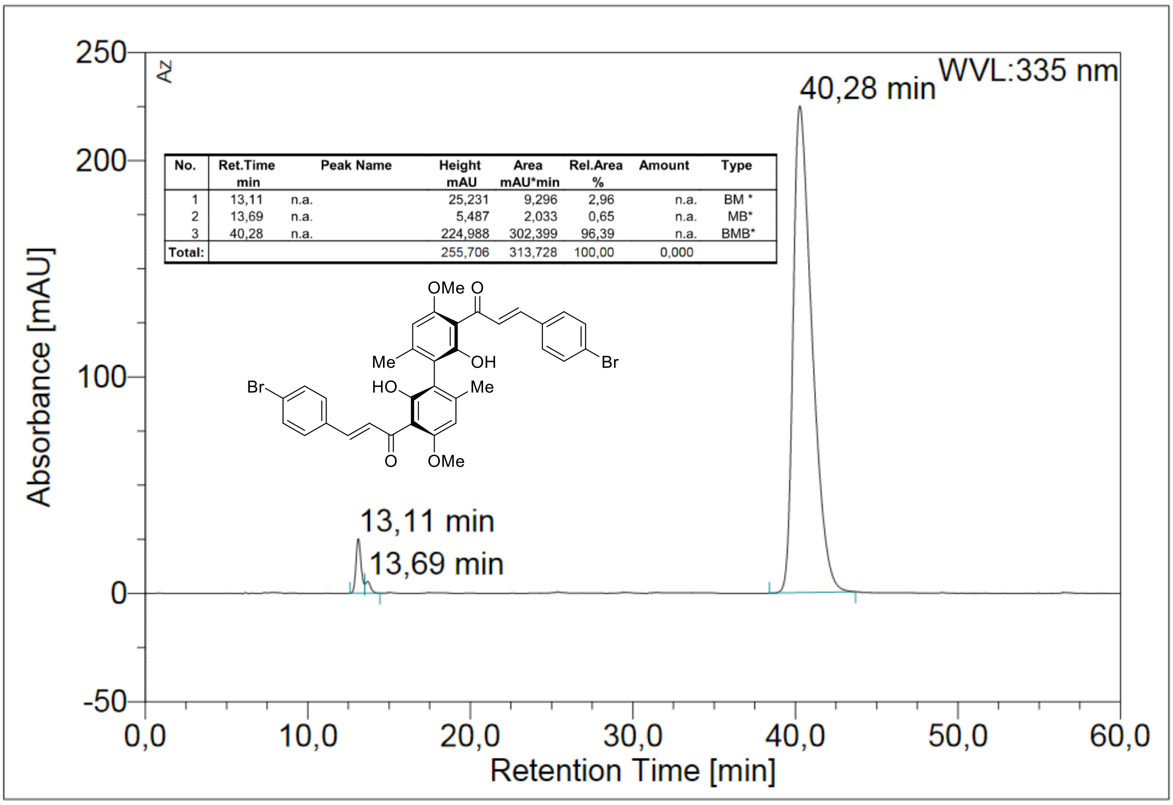

| No. | Ret.Time min | Peak Name | Height mAU | Area mAU*min | Rel.Area % | Amount | Type |
|---|---|---|---|---|---|---|---|
| 1 | 13,11 | n.a. | 25,231 | 9,296 | 2,96 | n.a. | BM * |
| 2 | 13,69 | n.a. | 5,487 | 2,033 | 0,65 | n.a. | MB* |
| 3 | 40,28 | n.a. | 224,988 | 302,399 | 96,39 | n.a. | BMB* |
| Total: | | | 255,706 | 313,728 | 100,00 | 0,000 | |



Figure S26: HPLC Chromatogram of biaryl **13** >99%*ee* ($S_a$). CHIRALPAK® IA (Daciel) 250 ° 4.6 mm, 25 °C, 0.5 mL min$^{-1}$, 335 nm, *n*-heptane:*i*-PrOH 50:50 (v/v) $t_R(S_a)$ = 25.5 min, $t_R(R_a)$ = 40.9 min.

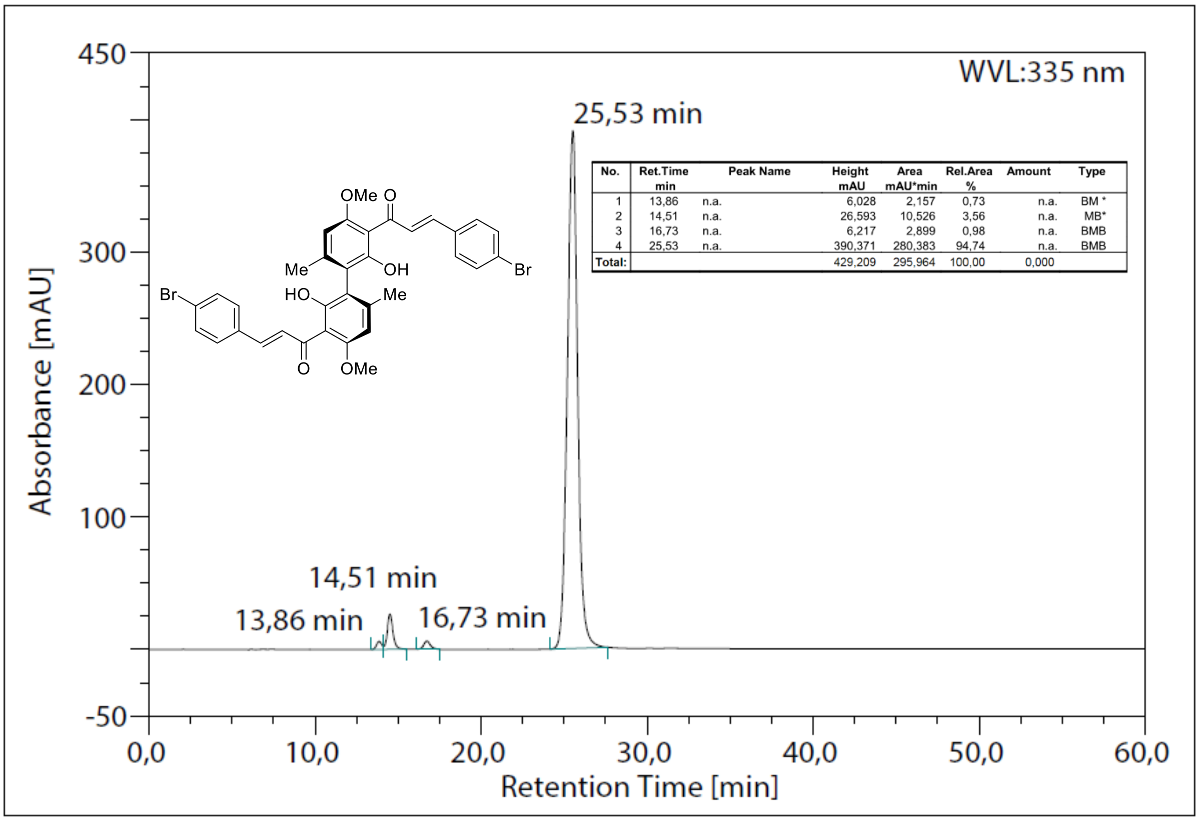


| No. | Ret.Time min | Peak Name | Height mAU | Area mAU*min | Rel.Area % | Amount | Type |
|---|---|---|---|---|---|---|---|
| 1 | 13,86 | n.a. | 6,028 | 2,157 | 0,73 | n.a. | BM * |
| 2 | 14,51 | n.a. | 26,593 | 10,526 | 3,56 | n.a. | MB* |
| 3 | 16,73 | n.a. | 6,217 | 2,899 | 0,98 | n.a. | BMB |
| 4 | 25,53 | n.a. | 390,371 | 280,383 | 94,74 | n.a. | BMB |
| Total: | | | 429,209 | 295,964 | 100,00 | 0,000 | |

Figure S27: HPLC Chromatogram of biaryl **13** >99%*ee* (*R*a). CHIRALPAK® IA (Daciel) 250 ° 4.6 mm, 25 °C, 0.5 mL min$^{-1}$, 335 nm, *n*-heptane:*i*-PrOH 50:50 (v/v) $t_R$(*S*a) = 25.5 min, $t_R$(*R*a) = 40.9 min.

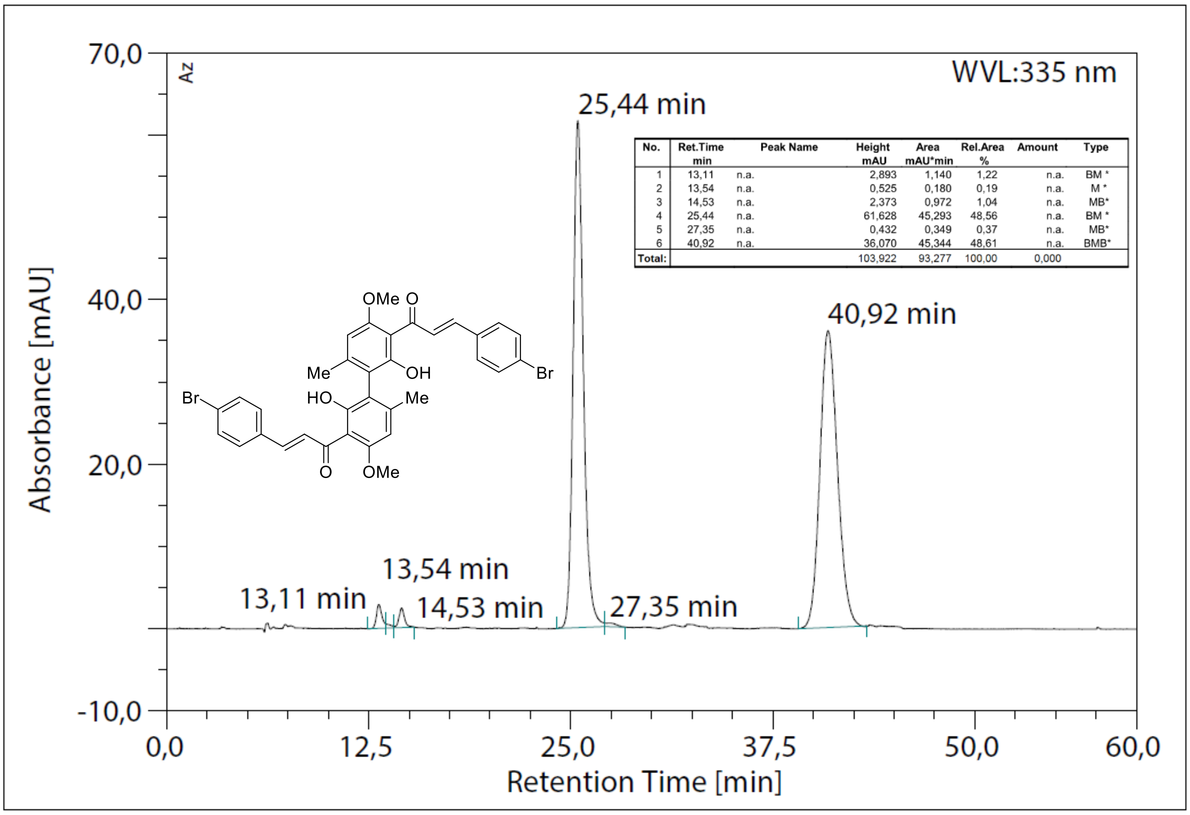


| No. | Ret.Time min | Peak Name | Height mAU | Area mAU*min | Rel.Area % | Amount | Type |
|---|---|---|---|---|---|---|---|
| 1 | 13,11 | n.a. | 2,893 | 1,140 | 1,22 | n.a. | BM * |
| 2 | 13,54 | n.a. | 0,525 | 0,180 | 0,19 | n.a. | M * |
| 3 | 14,53 | n.a. | 2,373 | 0,972 | 1,04 | n.a. | MB* |
| 4 | 25,44 | n.a. | 61,628 | 45,293 | 48,56 | n.a. | BM * |
| 5 | 27,35 | n.a. | 0,432 | 0,349 | 0,37 | n.a. | MB* |
| 6 | 40,92 | n.a. | 36,070 | 45,344 | 48,61 | n.a. | BMB* |
| Total: | | | 103,922 | 93,277 | 100,00 | 0,000 | |

Figure S28: HPLC Chromatogram of biaryl *rac*-**13**. CHIRALPAK® IA (Daciel) 250 ° 4.6 mm, 25 °C, 0.5 mL min$^{-1}$, 335 nm, *n*-heptane:*i*-PrOH 50:50 (v/v) $t_R$(*S*a) = 25.5 min, $t_R$(*R*a) = 40.9 min.

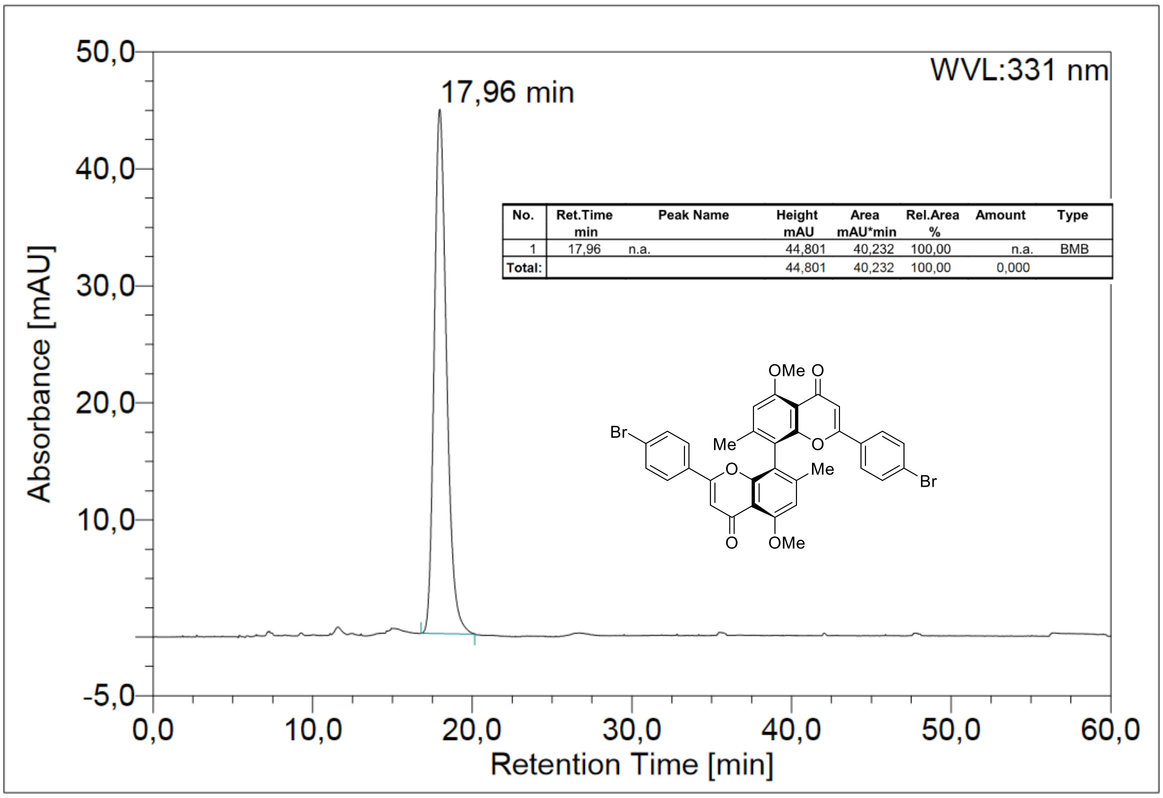


| No. | Ret.Time min | Peak Name | Height mAU | Area mAU*min | Rel.Area % | Amount | Type |
|---|---|---|---|---|---|---|---|
| 1 | 17,96 | n.a. | 44,801 | 40,232 | 100,00 | n.a. | BMB |
| Total: | | | 44,801 | 40,232 | 100,00 | 0,000 | |

Figure S29: HPLC Chromatogram of biaryl **2** >99%*ee* ($S_a$). Lux® Amylose-1 (Phenomenex) 250 ° 4.6 mm, 25 °C, 0.5 mL min$^{-1}$, 331 nm, *n*-heptane:*i*-PrOH 50:50 (v/v) $t_R$($S_a$) = 18.0 min, $t_R$($R_a$) = 24.3 min.

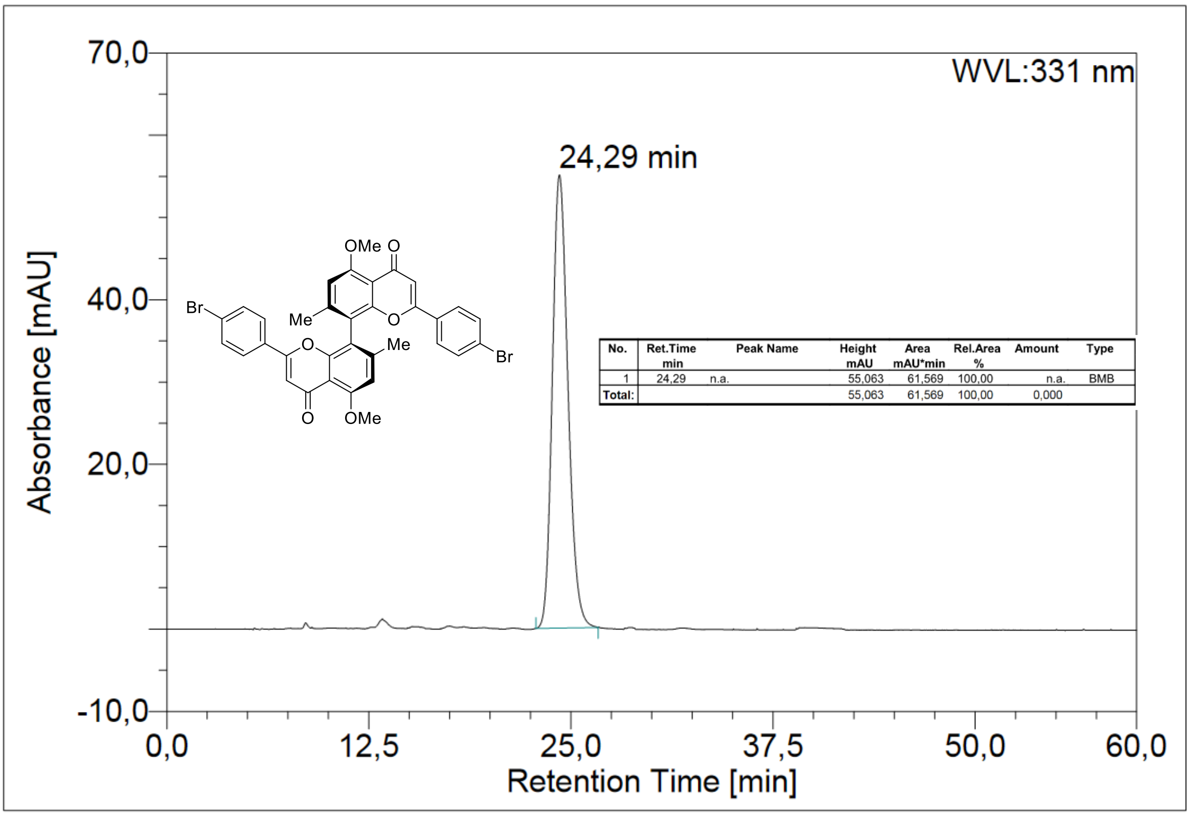


| No. | Ret.Time min | Peak Name | Height mAU | Area mAU*min | Rel.Area % | Amount | Type |
|---|---|---|---|---|---|---|---|
| 1 | 24,29 | n.a. | 55,063 | 61,569 | 100,00 | n.a. | BMB |
| Total: | | | 55,063 | 61,569 | 100,00 | 0,000 | |

Figure S30: HPLC Chromatogram of biaryl **2** >99%*ee* ($R_a$). Lux® Amylose-1 (Phenomenex) 250 ° 4.6 mm, 25 °C, 0.5 mL min$^{-1}$, 331 nm, *n*-heptane:*i*-PrOH 50:50 (v/v) $t_R$($S_a$) = 18.0 min, $t_R$($R_a$) = 24.3 min.

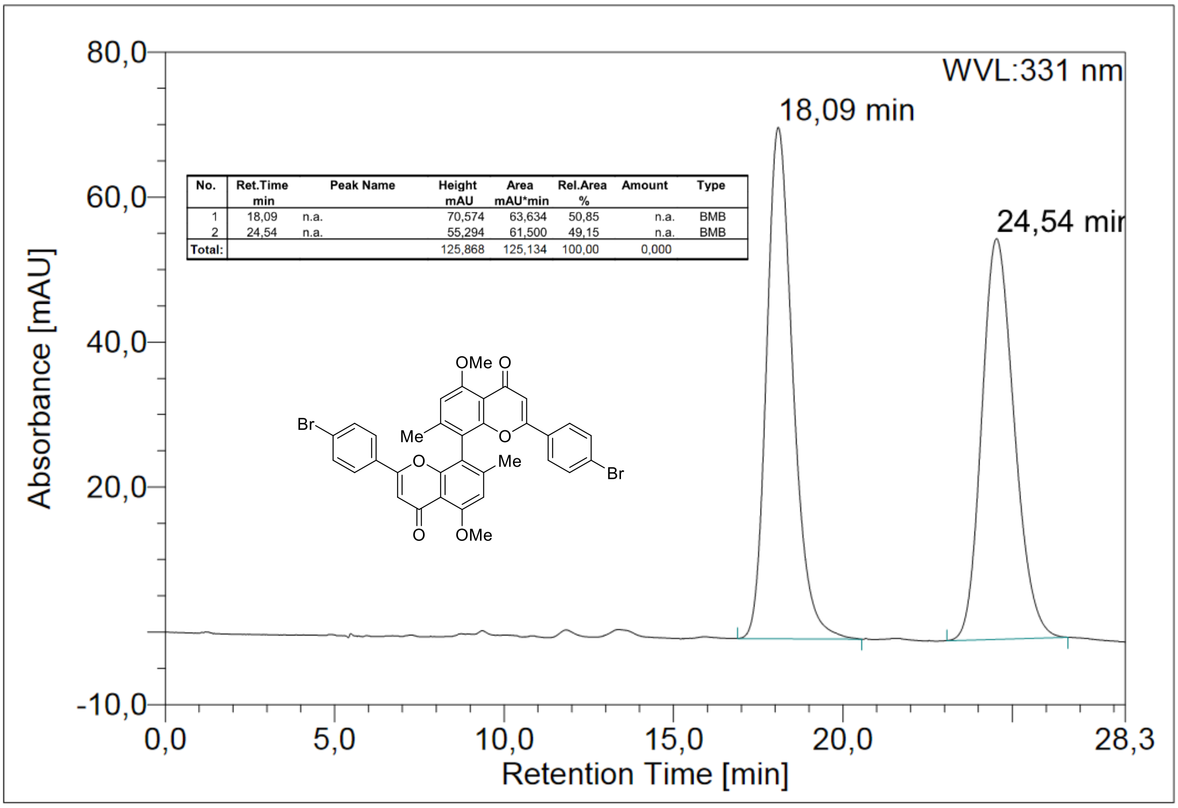


Figure S31: HPLC Chromatogram of biaryl *rac*-**2**. Lux® Amylose-1 (Phenomenex) 250 ° 4.6 mm, 25 °C, 0.5 mL min$^{-1}$, 331 nm, *n*-heptane:*i*-PrOH 50:50 (v/v) $t_R$($S_a$) = 18.0 min, $t_R$($R_a$) = 24.3 min.

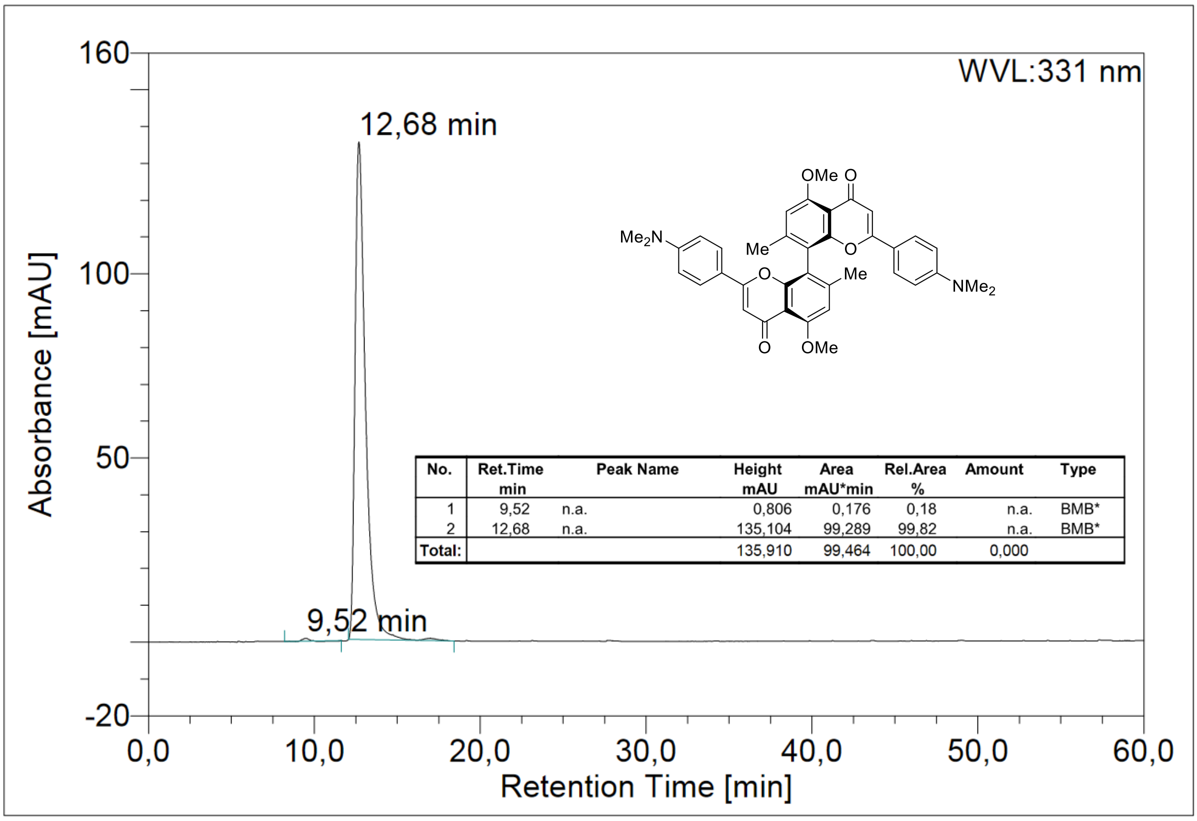


Figure S32: HPLC Chromatogram of biaryl **14** >99%*ee* ($S_a$). Lux® Amylose-1 (Phenomenex) 250 ° 4.6 mm, 25 °C, 0.5 mL min$^{-1}$, 331 nm, *n*-heptane:*i*-PrOH 50:50 (v/v) $t_R$($S_a$) = 9.53 min, $t_R$($R_a$) = 12.8 min.

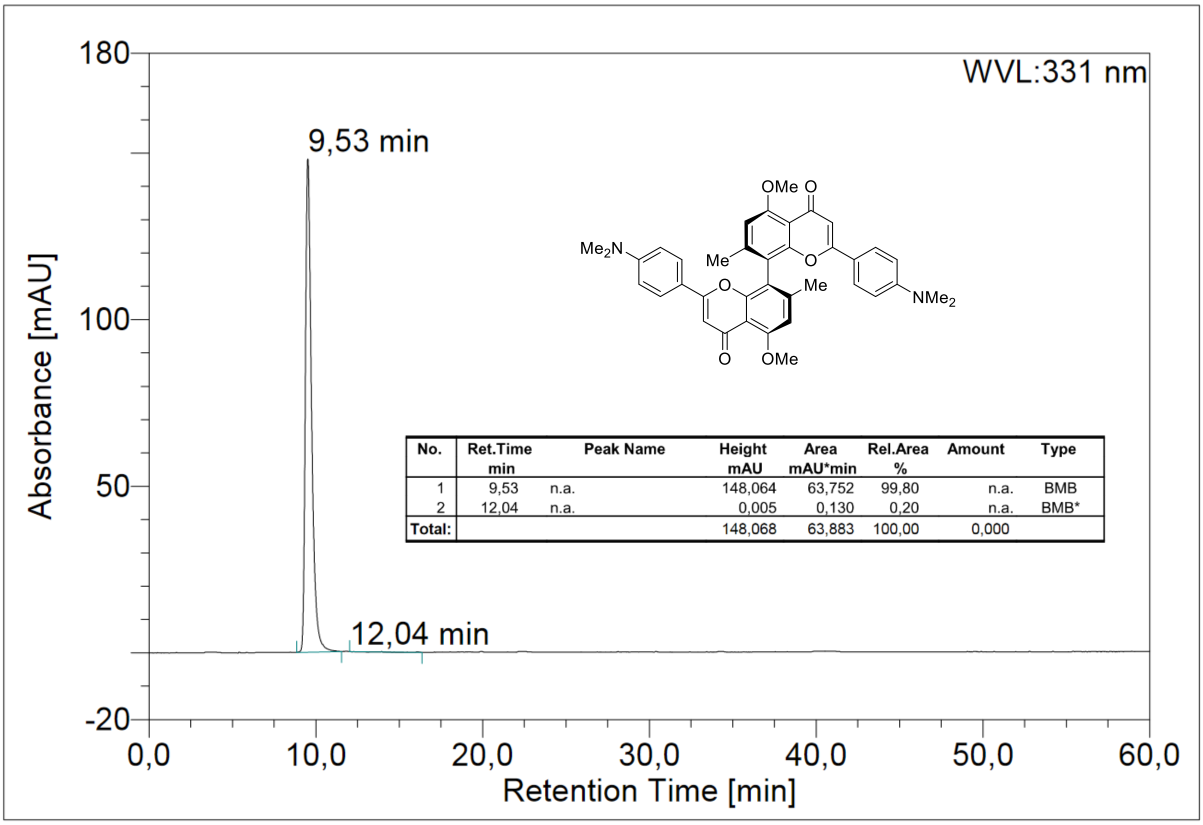


| No. | Ret.Time min | Peak Name | Height mAU | Area mAU*min | Rel.Area % | Amount | Type |
|---|---|---|---|---|---|---|---|
| 1 | 9,53 | n.a. | 148,064 | 63,752 | 99,80 | n.a. | BMB |
| 2 | 12,04 | n.a. | 0,005 | 0,130 | 0,20 | n.a. | BMB* |
| Total: | | | 148,068 | 63,883 | 100,00 | 0,000 | |

Figure S33: HPLC Chromatogram of biaryl **14** >99%*ee* ($R_a$). Lux® Amylose-1 (Phenomenex) 250 ° 4.6 mm, 25 °C, 0.5 mL min$^{-1}$, 331 nm, *n*-heptane:*i*-PrOH 50:50 (v/v) $t_R$($S_a$) = 9.53 min, $t_R$($R_a$) = 12.8 min.

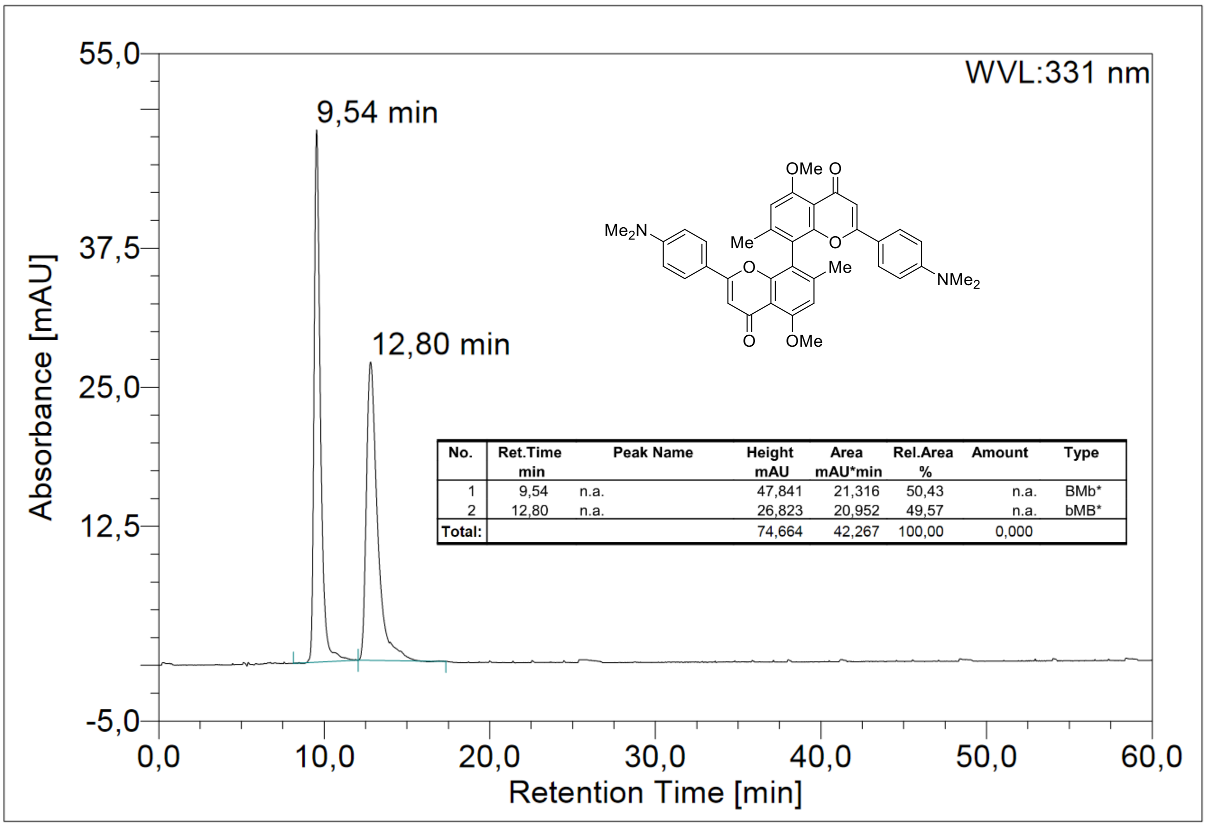


| No. | Ret.Time min | Peak Name | Height mAU | Area mAU*min | Rel.Area % | Amount | Type |
|---|---|---|---|---|---|---|---|
| 1 | 9,54 | n.a. | 47,841 | 21,316 | 50,43 | n.a. | BMb* |
| 2 | 12,80 | n.a. | 26,823 | 20,952 | 49,57 | n.a. | bMB* |
| Total: | | | 74,664 | 42,267 | 100,00 | 0,000 | |

Figure S34: HPLC Chromatogram of biaryl *rac*-**14**. Lux® Amylose-1 (Phenomenex) 250 ° 4.6 mm, 25 °C, 0.5 mL min$^{-1}$, 331 nm, *n*-heptane:*i*-PrOH 50:50 (v/v) $t_R$($S_a$) = 9.53 min, $t_R$($R_a$) = 12.8 min.

## 1.5 Reversed Phase HPLC

Method:

Hyperclone 5 µ ODS (C18), 125*4 mm, 120 Å, Fa. Phenomenex, 1 mL/min, A: $H_2O$; B: Methanol

| | | |
|---|---|---|
| Gradient: | 0-2 min | 10% B/90% A |
| | 2-15 min | Gradient 10% B/90% A to 100% B/0% A |
| | 15-20 min | 100% B/0% A |
| | 20-21 min | Gradient 100% B/0% A to 10% B/90% A |
| | 21-30 min | 10% B/90% A |

Table S9: Purity of biflavones subjected to biological testing.

| **Compound** | ***t*R [min]** | **Purity according to reversed phase HPLC [%]** |
|---|---|---|
| **14** | 15.67 | 76.8 |
| **15** | 16.86 | 94.9 |
| **16** | 16.86 | 94.3 |
| **17** | 14.75 | 92.5 |
| **18** | 17.88 | 92.5 |
| **19** | 16.11 | 97.1 |
| **20** | 15.95 | 95.1 |
| **21** | 14.40 | 95.4 |
| **22** | 14.71 | 86.7 |
| **29** | 14.71 | 99.7 |
| **30** | 15.61 | 96.9 |
| ($S_a$)-**14** | 15.43 | 98.5 |
| ($R_a$)-**14** | 15.49 | 98.9 |

Samples injected using DMSO stock solution.

## 1.5.1 HPLC Chromatograms

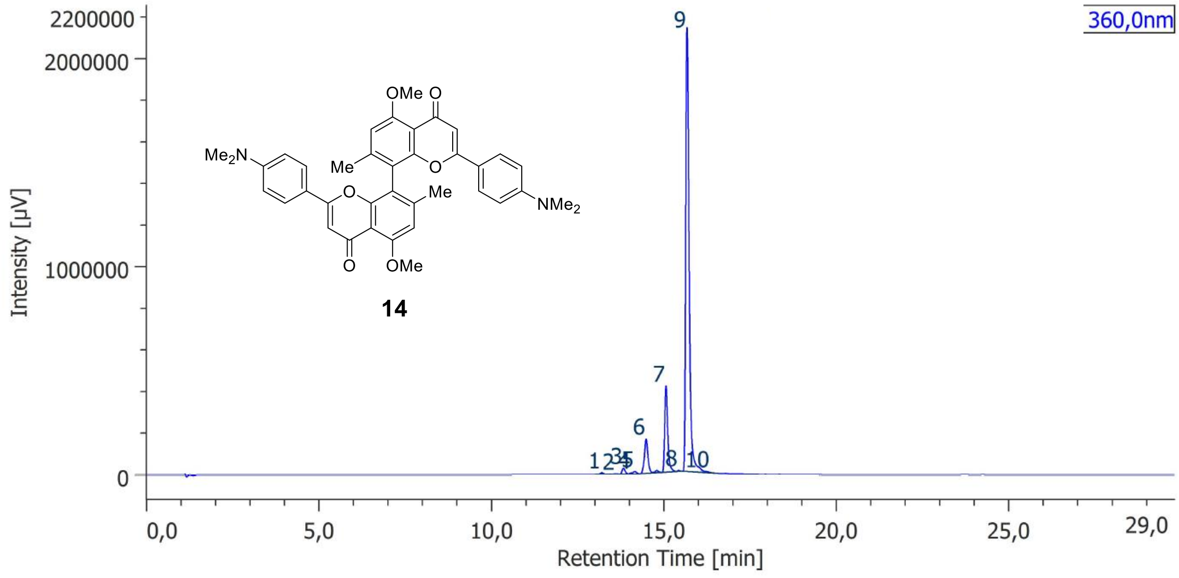


| # | Peak Name | CH | tR [min] | Area [µV·sec] | Height [µV] | Area% | Height% | Symmetry Factor | Warning |
|---|---|---|---|---|---|---|---|---|---|
| 1 | Unknown | 10 | 13,188 | 46366 | 7766 | 0,246 | 0,280 | 1,022 | |
| 2 | Unknown | 10 | 13,593 | 11045 | 1753 | 0,058 | 0,063 | 0,929 | |
| 3 | Unknown | 10 | 13,823 | 170714 | 28570 | 0,904 | 1,029 | 1,160 | |
| 4 | Unknown | 10 | 14,043 | 23762 | 5129 | 0,126 | 0,185 | N/A | |
| 5 | Unknown | 10 | 14,150 | 75056 | 11299 | 0,398 | 0,407 | N/A | |
| 6 | Unknown | 10 | 14,478 | 1177775 | 166129 | 6,238 | 5,983 | 1,067 | |
| 7 | Unknown | 10 | 15,055 | 2579169 | 414449 | 13,660 | 14,926 | 1,442 | |
| 8 | Unknown | 10 | 15,413 | 24924 | 4687 | 0,132 | 0,169 | N/A | |
| 9 | Unknown | 10 | 15,665 | 14499915 | 2131114 | 76,797 | 76,751 | 1,614 | |
| 10 | Unknown | 10 | 16,162 | 272155 | 5780 | 1,441 | 0,208 | N/A | |

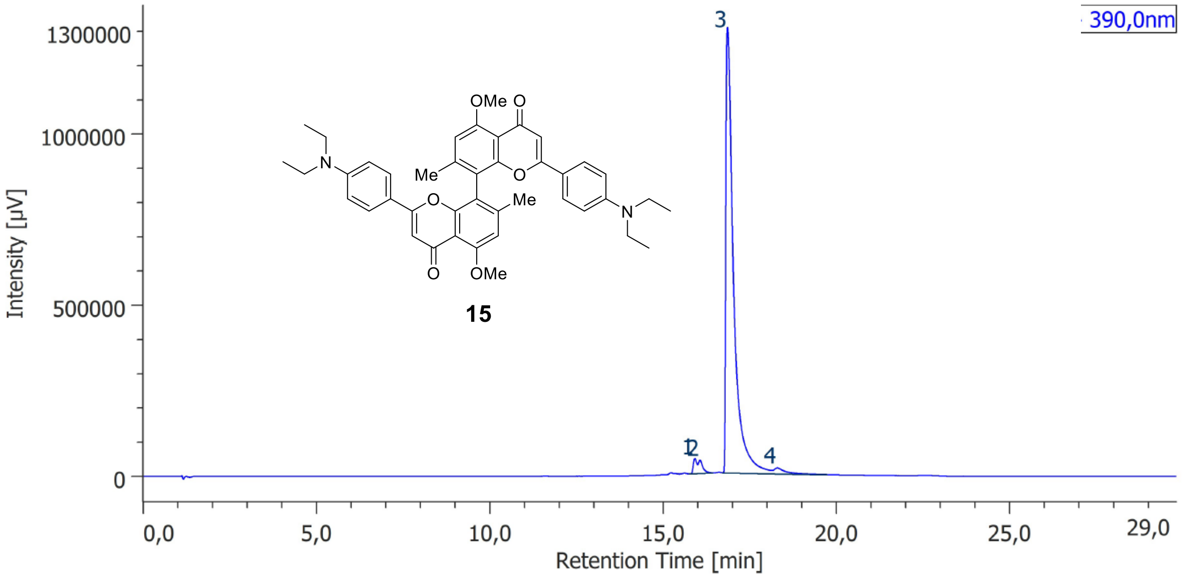


| # | Peak Name | CH | tR [min] | Area [µV·sec] | Height [µV] | Area% | Height% | Symmetry Factor | Warning |
|---|---|---|---|---|---|---|---|---|---|
| 1 | Unknown | 10 | 15,910 | 305826 | 44638 | 1,437 | 3,185 | N/A | |
| 2 | Unknown | 10 | 16,057 | 359739 | 38967 | 1,691 | 2,780 | N/A | |
| 3 | Unknown | 10 | 16,855 | 20189443 | 1300691 | 94,881 | 92,797 | 3,708 | |
| 4 | Unknown | 10 | 18,285 | 423770 | 17363 | 1,992 | 1,239 | N/A | |

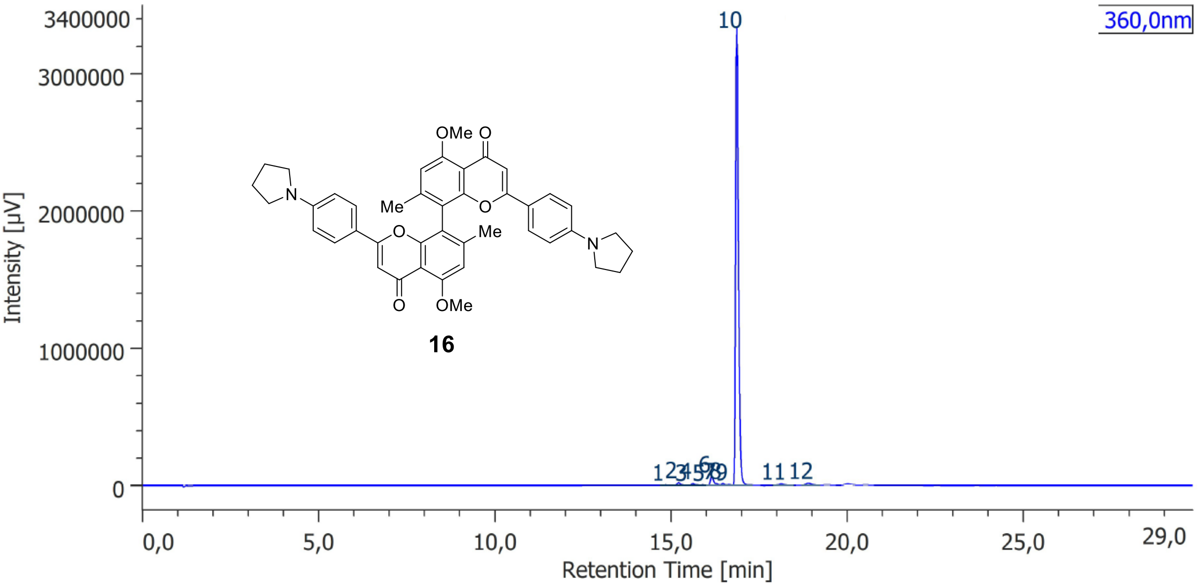


| # | Peak Name | CH | tR [min] | Area [µV·sec] | Height [µV] | Area% | Height% | Symmetry Factor | Warning |
|---|---|---|---|---|---|---|---|---|---|
| 1 | Unknown | 10 | 14,833 | 22893 | 3355 | 0,115 | 0,096 | 0,886 | |
| 2 | Unknown | 10 | 15,205 | 136247 | 19156 | 0,687 | 0,550 | N/A | |
| 3 | Unknown | 10 | 15,488 | 13457 | 513 | 0,068 | 0,015 | N/A | |
| 4 | Unknown | 10 | 15,608 | 75079 | 12401 | 0,379 | 0,356 | N/A | |
| 5 | Unknown | 10 | 15,978 | 0 | 2 | 0,000 | 0,000 | N/A | |
| 6 | Unknown | 10 | 16,152 | 386828 | 63084 | 1,951 | 1,812 | N/A | |
| 7 | Unknown | 10 | 16,293 | 60405 | 11251 | 0,305 | 0,323 | N/A | |
| 8 | Unknown | 10 | 16,463 | 72960 | 13639 | 0,368 | 0,392 | N/A | |
| 9 | Unknown | 10 | 16,633 | 31164 | 5075 | 0,157 | 0,146 | N/A | |
| 10 | Unknown | 10 | 16,863 | 18705196 | 3326418 | 94,348 | 95,546 | 1,353 | |
| 11 | Unknown | 10 | 18,110 | 129634 | 10841 | 0,654 | 0,311 | 1,165 | |
| 12 | Unknown | 10 | 18,885 | 191862 | 15755 | 0,968 | 0,453 | 1,320 | |

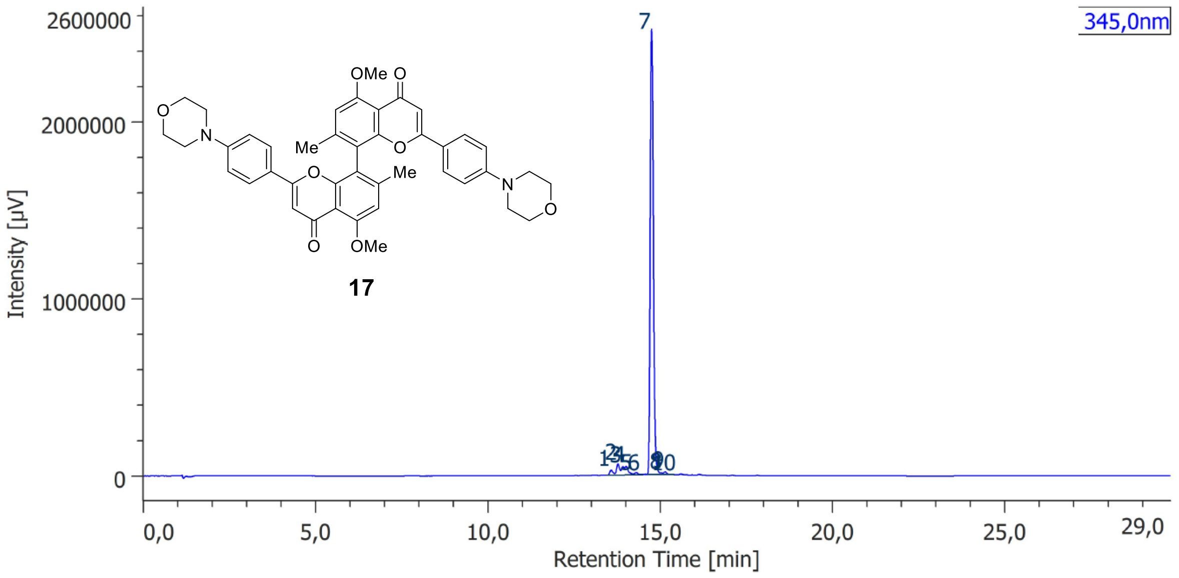


| # | Peak Name | CH | tR [min] | Area [μV·sec] | Height [μV] | Area% | Height% | Symmetry Factor | Warning |
|---|---|---|---|---|---|---|---|---|---|
| 1 | Unknown | 10 | 13,568 | 191606 | 28341 | 1,081 | 1,037 | N/A | |
| 2 | Unknown | 10 | 13,770 | 376222 | 62843 | 2,123 | 2,300 | N/A | |
| 3 | Unknown | 10 | 13,910 | 266858 | 47023 | 1,506 | 1,721 | N/A | |
| 4 | Unknown | 10 | 14,005 | 284662 | 48530 | 1,607 | 1,776 | N/A | |
| 5 | Unknown | 10 | 14,205 | 67933 | 3755 | 0,383 | 0,137 | N/A | |
| 6 | Unknown | 10 | 14,430 | 670 | 256 | 0,004 | 0,009 | 1,489 | |
| 7 | Unknown | 10 | 14,745 | 16386035 | 2515909 | 92,483 | 92,070 | 1,254 | |
| 8 | Unknown | 10 | 15,067 | 40973 | 8428 | 0,231 | 0,308 | N/A | |
| 9 | Unknown | 10 | 15,133 | 95926 | 16165 | 0,541 | 0,592 | N/A | |
| 10 | Unknown | 10 | 15,300 | 7027 | 1355 | 0,040 | 0,050 | N/A | |

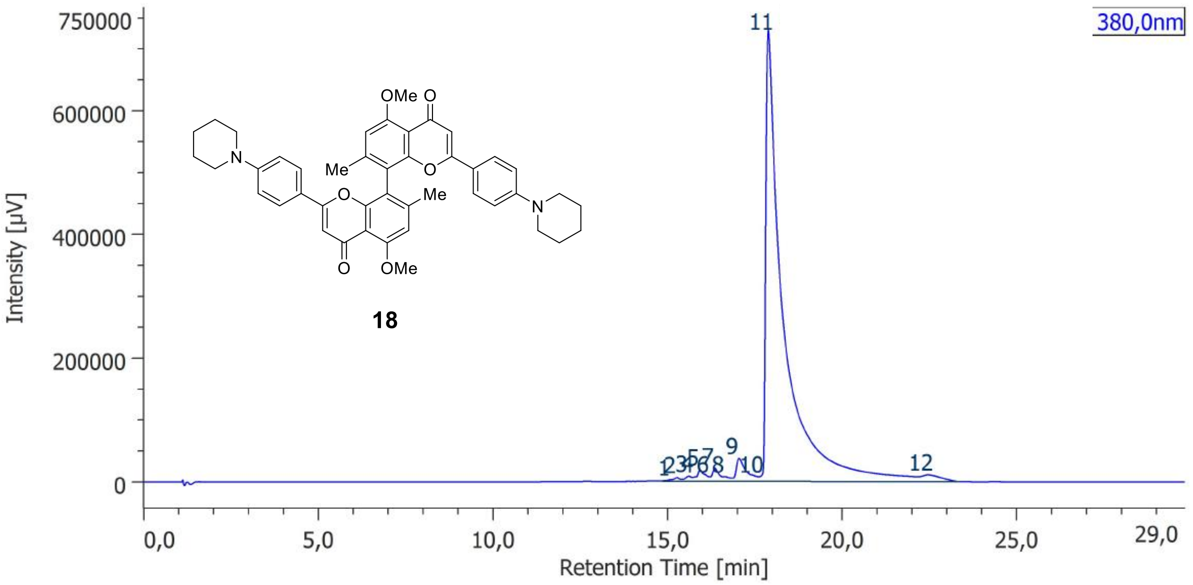


| # | Peak Name | CH | tR [min] | Area [µV·sec] | Height [µV] | Area% | Height% | Symmetry Factor | Warning |
|---|---|---|---|---|---|---|---|---|---|
| 1 | Unknown | 10 | 15,102 | 13847 | 1911 | 0,050 | 0,223 | N/A | |
| 2 | Unknown | 10 | 15,265 | 65982 | 5499 | 0,238 | 0,640 | N/A | |
| 3 | Unknown | 10 | 15,597 | 98427 | 7660 | 0,355 | 0,892 | N/A | |
| 4 | Unknown | 10 | 15,768 | 43010 | 6677 | 0,155 | 0,777 | N/A | |
| 5 | Unknown | 10 | 15,923 | 165702 | 18370 | 0,598 | 2,139 | N/A | |
| 6 | Unknown | 10 | 16,205 | 100133 | 6940 | 0,361 | 0,808 | N/A | |
| 7 | Unknown | 10 | 16,350 | 276588 | 20685 | 0,998 | 2,408 | N/A | |
| 8 | Unknown | 10 | 16,648 | 97515 | 7015 | 0,352 | 0,817 | N/A | |
| 9 | Unknown | 10 | 17,040 | 630369 | 36886 | 2,274 | 4,295 | N/A | |
| 10 | Unknown | 10 | 17,593 | 124041 | 7108 | 0,448 | 0,828 | N/A | |
| 11 | Unknown | 10 | 17,882 | 25649352 | 729257 | 92,547 | 84,910 | 6,978 | |
| 12 | Unknown | 10 | 22,450 | 450118 | 10849 | 1,624 | 1,263 | N/A | |

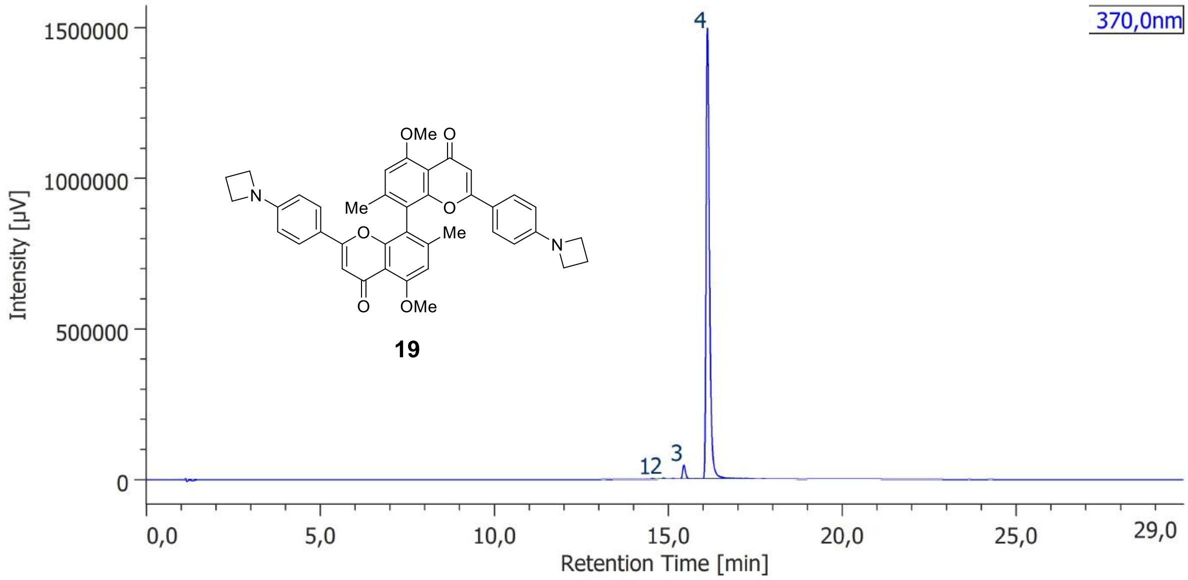


| # | Peak Name | CH | tR [min] | Area [µV·sec] | Height [µV] | Area% | Height% | Symmetry Factor | Warning |
|---|---|---|---|---|---|---|---|---|---|
| 1 | Unknown | 10 | 14,528 | 14236 | 2643 | 0,136 | 0,171 | 1,206 | |
| 2 | Unknown | 10 | 14,853 | 16611 | 3258 | 0,159 | 0,211 | 1,045 | |
| 3 | Unknown | 10 | 15,443 | 267111 | 46294 | 2,558 | 2,993 | 1,212 | |
| 4 | Unknown | 10 | 16,118 | 10145483 | 1494603 | 97,147 | 96,626 | 1,531 | |

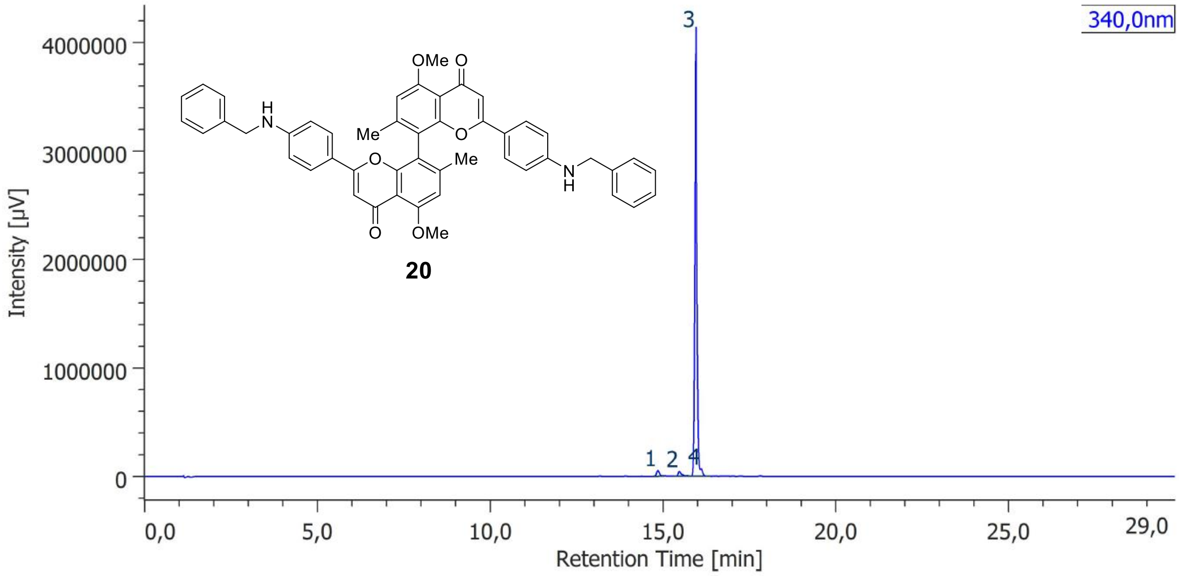


| # | Peak Name | CH | tR [min] | Area [µV·sec] | Height [µV] | Area% | Height% | Symmetry Factor | Warning |
|---|---|---|---|---|---|---|---|---|---|
| 1 | Unknown | 10 | 14,843 | 278296 | 52036 | 1,634 | 1,209 | 1,160 | |
| 2 | Unknown | 10 | 15,468 | 222473 | 44530 | 1,306 | 1,035 | N/A | |
| 3 | Unknown | 10 | 15,953 | 16193703 | 4137018 | 95,091 | 96,154 | 0,984 | |
| 4 | Unknown | 10 | 16,097 | 335245 | 68906 | 1,969 | 1,602 | N/A | |

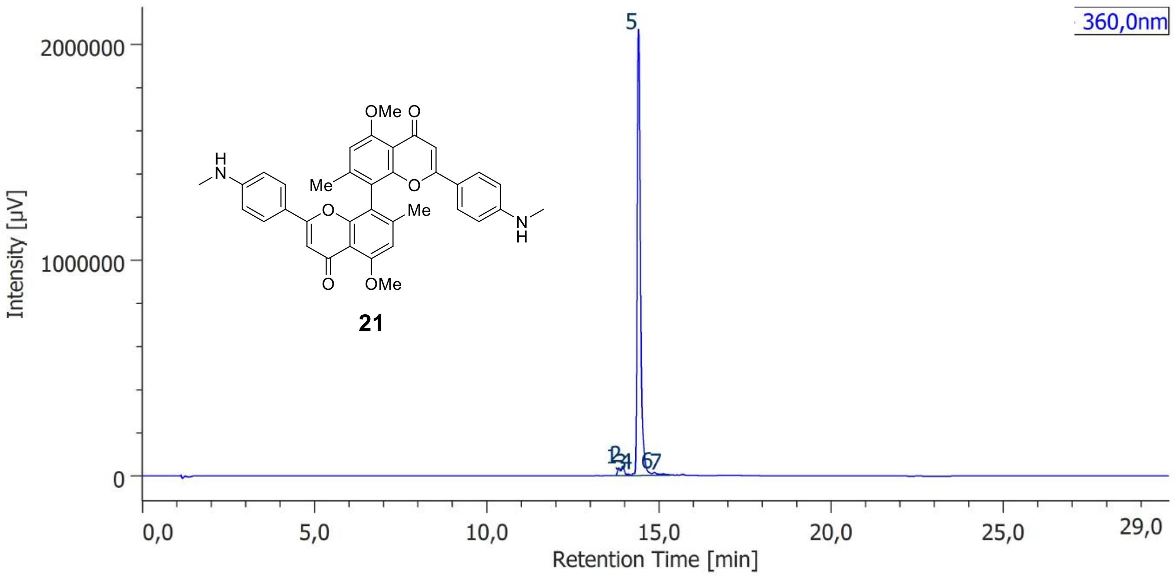


| # | Peak Name | CH | tR [min] | Area [μV·sec] | Height [μV] | Area% | Height% | Symmetry Factor | Warning |
|---|---|---|---|---|---|---|---|---|---|
| 1 | Unknown | 10 | 13,827 | 196154 | 36741 | 1,342 | 1,685 | N/A | |
| 2 | Unknown | 10 | 13,943 | 257465 | 44195 | 1,762 | 2,026 | N/A | |
| 3 | Unknown | 10 | 14,095 | 32926 | 6149 | 0,225 | 0,282 | N/A | |
| 4 | Unknown | 10 | 14,252 | 41885 | 9878 | 0,287 | 0,453 | N/A | |
| 5 | Unknown | 10 | 14,398 | 13935183 | 2066232 | 95,368 | 94,740 | 1,616 | |
| 6 | Unknown | 10 | 14,853 | 106945 | 12322 | 0,732 | 0,565 | N/A | |
| 7 | Unknown | 10 | 15,110 | 41418 | 5431 | 0,283 | 0,249 | N/A | |

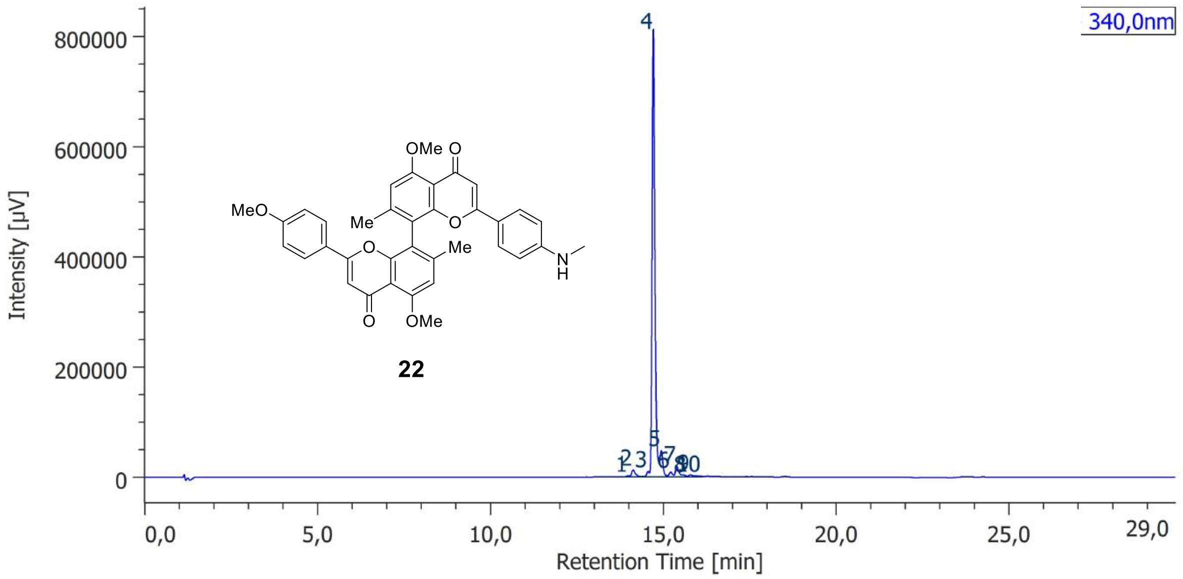


| # | Peak Name | CH | tR [min] | Area [µV·sec] | Height [µV] | Area% | Height% | Symmetry Factor | Warning |
|---|---|---|---|---|---|---|---|---|---|
| 1 | Unknown | 10 | 13,985 | 9294 | 1734 | 0,178 | 0,189 | N/A | |
| 2 | Unknown | 10 | 14,118 | 94488 | 12628 | 1,811 | 1,380 | N/A | |
| 3 | Unknown | 10 | 14,550 | 49233 | 9811 | 0,944 | 1,072 | N/A | |
| 4 | Unknown | 10 | 14,707 | 4523975 | 811670 | 86,729 | 88,692 | 1,288 | |
| 5 | Unknown | 10 | 14,935 | 299913 | 47571 | 5,750 | 5,198 | N/A | |
| 6 | Unknown | 10 | 15,202 | 57800 | 8439 | 1,108 | 0,922 | N/A | |
| 7 | Unknown | 10 | 15,385 | 126340 | 18637 | 2,422 | 2,036 | N/A | |
| 8 | Unknown | 10 | 15,678 | 23029 | 564 | 0,441 | 0,062 | N/A | |
| 9 | Unknown | 10 | 15,770 | 22378 | 3101 | 0,429 | 0,339 | N/A | |
| 10 | Unknown | 10 | 15,927 | 9795 | 998 | 0,188 | 0,109 | N/A | |

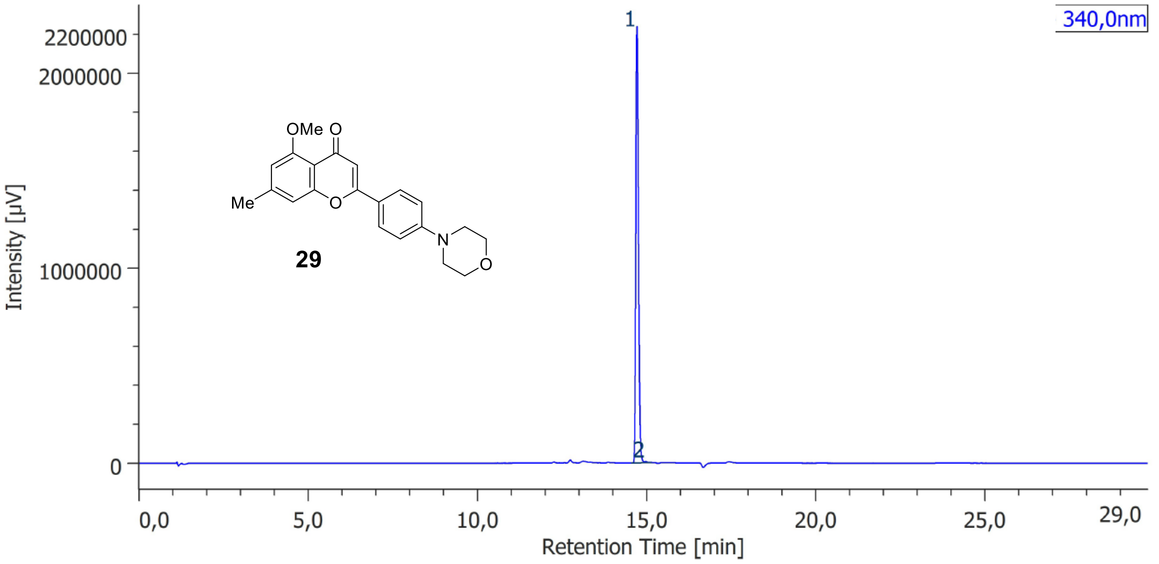


| # | Peak Name | CH | tR [min] | Area [µV·sec] | Height [µV] | Area% | Height% | Symmetry Factor | Warning |
|---|---|---|---|---|---|---|---|---|---|
| 1 | Unknown | 10 | 14,713 | 12319437 | 2235794 | 99,728 | 99,746 | 1,220 | |
| 2 | Unknown | 10 | 14,965 | 33611 | 5689 | 0,272 | 0,254 | N/A | |

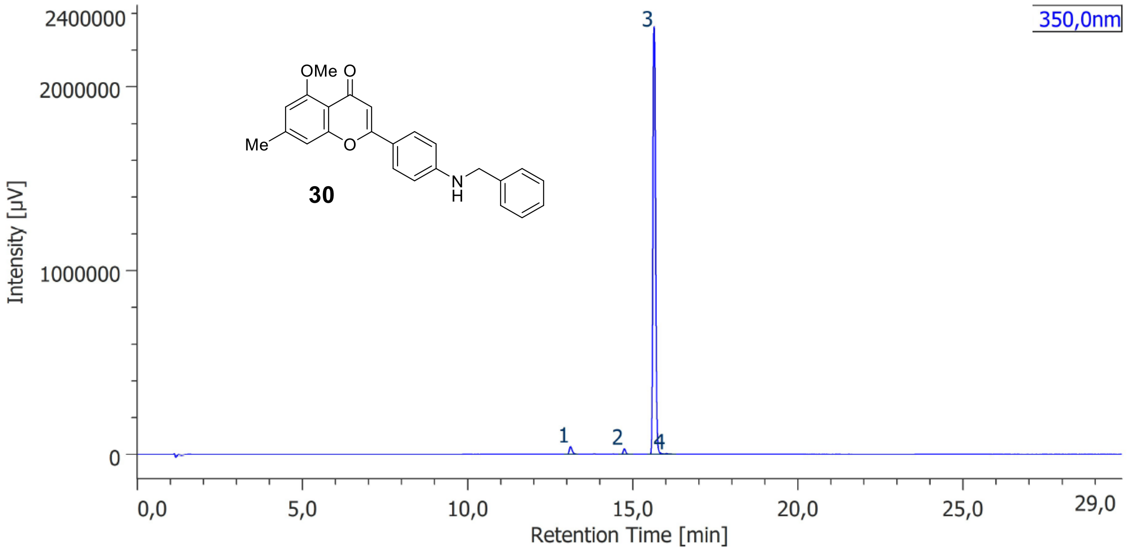


| # | Peak Name | CH | tR [min] | Area [µV·sec] | Height [µV] | Area% | Height% | Symmetry Factor | Warning |
|---|---|---|---|---|---|---|---|---|---|
| 1 | Unknown | 10 | 13,110 | 250526 | 41394 | 1,837 | 1,725 | 1,344 | |
| 2 | Unknown | 10 | 14,740 | 144343 | 29366 | 1,059 | 1,224 | 1,167 | |
| 3 | Unknown | 10 | 15,642 | 13208839 | 2324795 | 96,875 | 96,879 | 1,187 | |
| 4 | Unknown | 10 | 16,007 | 31191 | 4122 | 0,229 | 0,172 | N/A | |

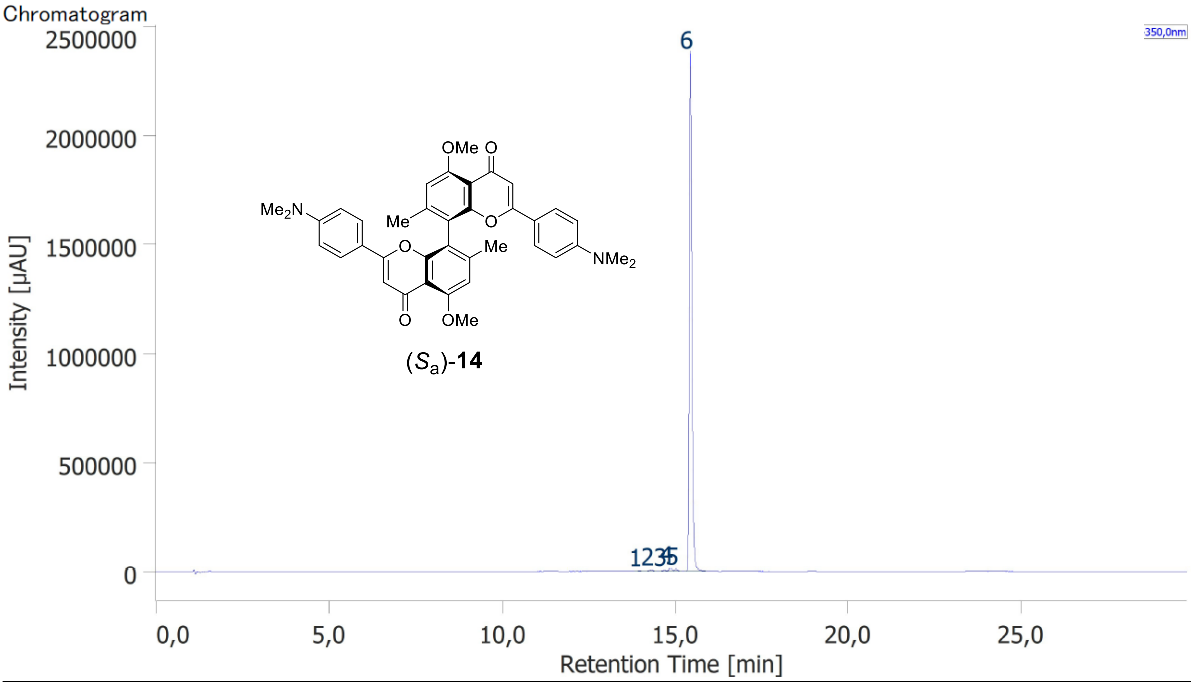


| # | Peak Name | CH | tR [min] | Area [µV·sec] | Height [µV] | Area% | Height% | Symmetry Factor | Warning |
|---|---|---|---|---|---|---|---|---|---|
| 1 | Unknown | 10 | 13,957 | 14574 | 2695 | 0,115 | 0,111 | 0,921 | |
| 2 | Unknown | 10 | 14,303 | 35988 | 4468 | 0,284 | 0,185 | 0,943 | |
| 3 | Unknown | 10 | 14,675 | 17805 | 3450 | 0,140 | 0,143 | N/A | |
| 4 | Unknown | 10 | 14,852 | 77669 | 13302 | 0,613 | 0,550 | N/A | |
| 5 | Unknown | 10 | 15,013 | 44378 | 7300 | 0,350 | 0,302 | N/A | |
| 6 | Unknown | 10 | 15,432 | 12483137 | 2386415 | 98,498 | 98,709 | 1,369 | |
| Total | | | | 12673551 | 2417630 | | | | |

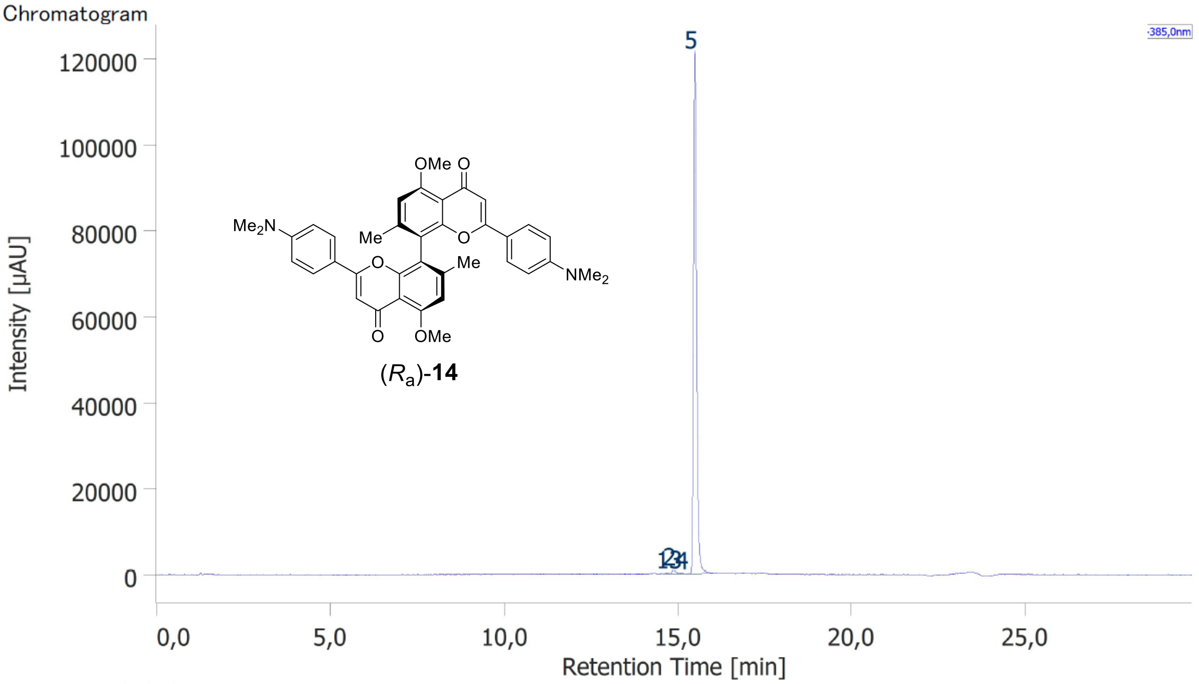


| # | Peak Name | CH | tR [min] | Area [μV·sec] | Height [μV] | Area% | Height% | Symmetry Factor | Warning |
|---|---|---|---|---|---|---|---|---|---|
| 1 | Unknown | 10 | 14,672 | 690 | 183 | 0,092 | 0,149 | 1,105 | |
| 2 | Unknown | 10 | 14,868 | 5928 | 860 | 0,787 | 0,699 | N/A | |
| 3 | Unknown | 10 | 15,048 | 1411 | 248 | 0,187 | 0,202 | N/A | |
| 4 | Unknown | 10 | 15,223 | 9 | 17 | 0,001 | 0,014 | N/A | |
| 5 | Unknown | 10 | 15,485 | 745062 | 121655 | 98,933 | 98,936 | 1,306 | |
| Total | | | | 753100 | 122963 | | | | |

# 2 Biological Data

We set out to investigate, if the means of synthesis, namely the presence of trace transition metal contaminations in the final products would affect the $IC_{50}$ values (Table S8).[15-17] To our delight, the $IC_{50}$ value of the previously synthesized biflavone **14** ($IC_{50\ HeLa}$ = 3.6 µM) as a control matched the $IC_{50}$ value of our newly synthesized amino biflavone via Buchwald-Hartwig coupling ($IC_{50\ HeLa}$ = 3.7 µM) (Table S10). With these assessments in place, the library was evaluated. Most synthesized 8,8″-amino biflavones showed no discernible bioactivity. The size of the amino substituent appeared to correlate with the $IC_{50}$ (low $IC_{50}$ meaning high bioactivity). Previously mentioned dimethylamine-biflavone **14**, pyrrolidine-biflavone **16**, methyl amine biflavone **21** and non-$C_2$-symmetrical methylamine/methoxy biflavone **22** exhibited biological activity ($IC_{50}$ <50 µM). To identify the mode of action of the selective cytotoxicity against HeLa cells, the autophagy modulating properties were assessed and minor effects observed for biflavones **14**, **16** and **18** (Figures S36).

## 2.1 Tables

Table S10: $IC_{50}$-values and confidence intervals of Cytotoxicity against HeLa cells with 95% confidence interval. A lower value corresponds to a higher activity. Compounds synthesized via palladium-mediated Buchwald-Hartwig amination. [a] Pd-free synthesis route.[9]

| **Compound** | **-NR$_2$** | **$IC_{50\ (HeLa)}$ [µM]** | **$IC_{50}$ 95% CI [µM]** |
|---|---|---|---|
| Biflavones | | | |
| **14** | 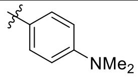 | 3.713 | N/A |
| **14**[a] | 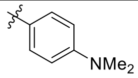 | 3.603 | N/A |
| **15** | 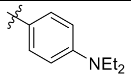 | >50 | N/A |
| **16** | 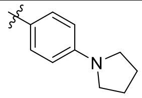 | 4.795 | 4.368 – 5.254 |
| **17** | 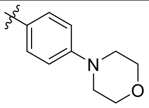 | >50 | N/A |
| **18** | 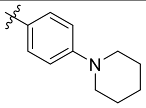 | >50 | N/A |
| **19** | 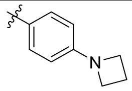 | >50 | N/A |
| **20** | 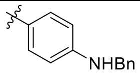 | >50 | N/A |
| **21** | 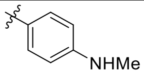 | 15.98 | 14.55 – 17.60 |
| **22** | 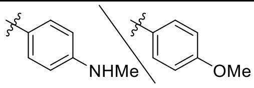 | 6.337 | 6.091 – 6.580 |
| (*R*a)-**14** | 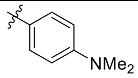 | 4.211 | N/A |
| (*S*a)-**14** | 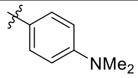 | 5.710 | 5.098 – 6.322 |
| Flavones | | | |
| **29** | 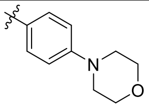 | >50 | N/A |
| **30** | 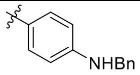 | >50 | N/A |

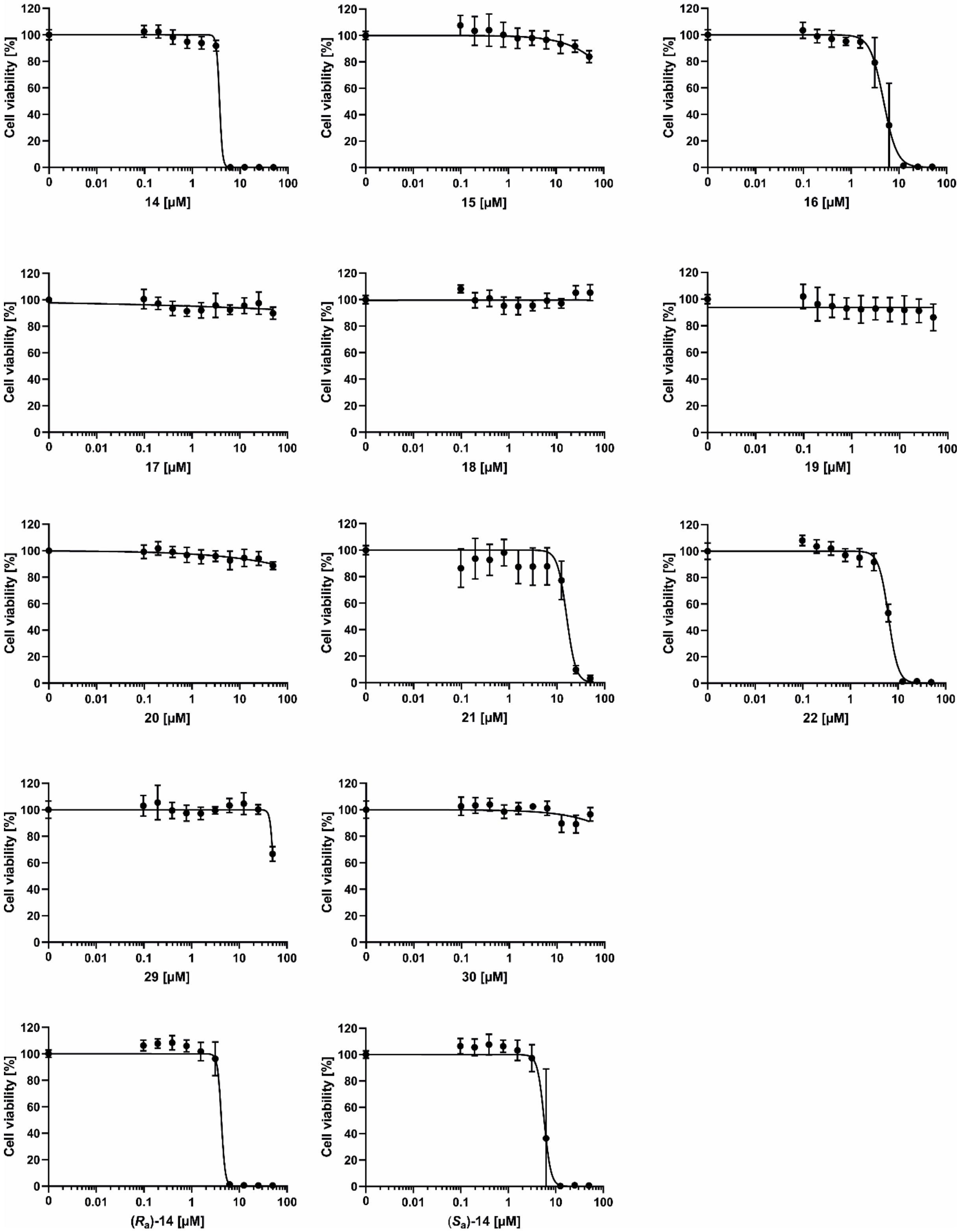


Figure S35: Analysis of the cytotoxicity of the compounds against human cancer cells. HeLa wt cells were treated with different concentrations of the respective compound for 48 h. After treatment, cell viability was measured using a resazurin reduction assay (Alamar Blue®). Results are shown as the mean ± SD of three independent experiments performed in triplicates for each treatment.

## 2.2 Western Blots

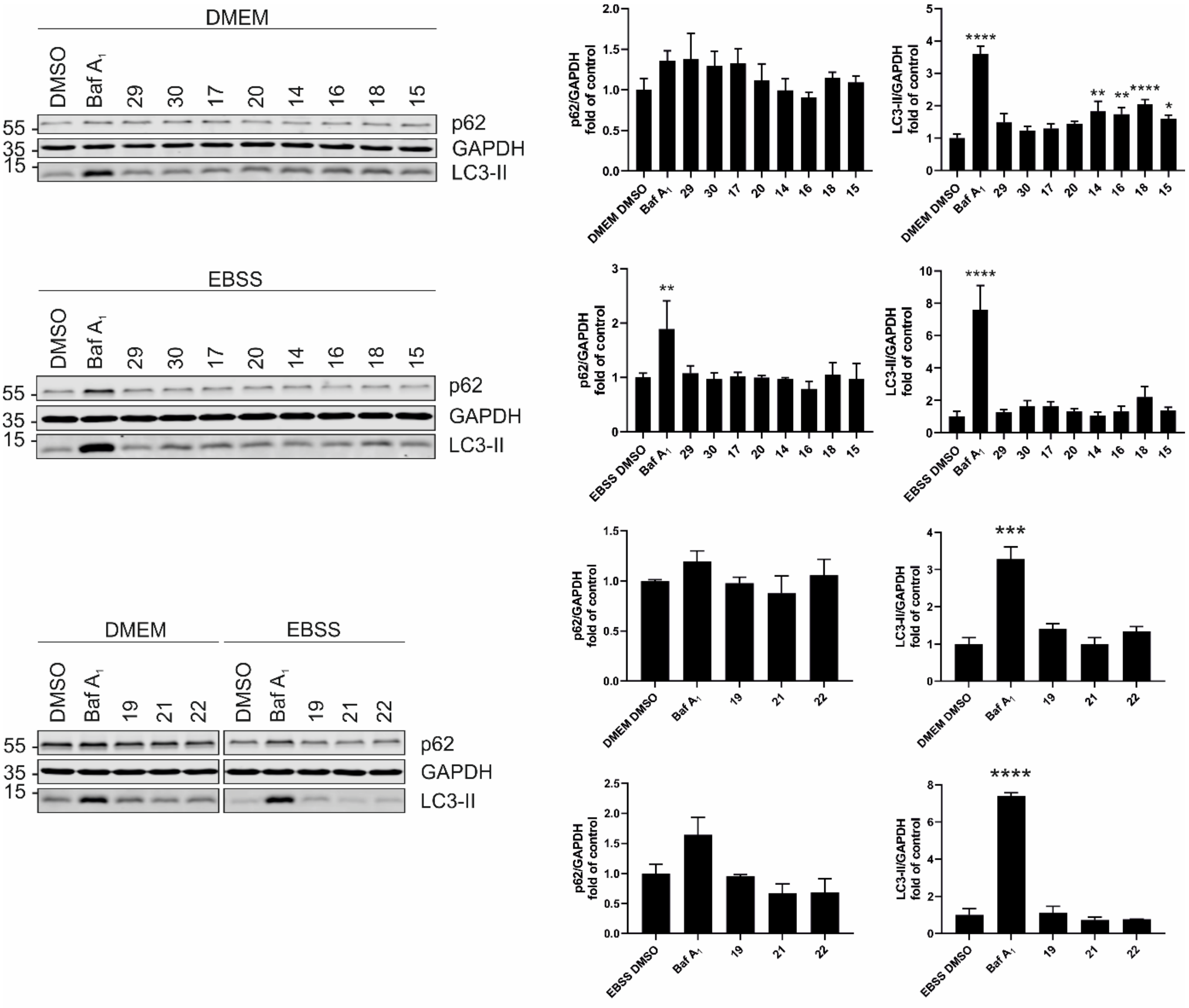


Figure S36: Analysis of possible autophagy-modulating properties of the compounds in human cancer cells. HeLa wt cells were treated with 10 µM of the respective compound or 40 nM Bafilomycin A1 as a positive control in full growth medium (DMEM) or starvation medium (EBSS) for 6 h. After cell lysis, lysates were analyzed by immunoblotting for p62, LC3 and GAPDH. Fold changes were calculated by dividing each normalized ratio (normalized protein to normalized GAPDH) by the average of the ratios of the control lane (solvent control DMSO). Results are shown as mean+SD. Statistical analysis was performed using ordinary one-way ANOVA (corrected by Tukey's multiple comparisons test). *P < 0.05, **P < 0.01, ***P < 0.001, ****P < 0.0001.

## 2.3 Materials and Methods

### Cell culture

HeLa wt cells were cultured in Dulbecco´s Modified Eagle Medium (DMEM, Gibco® by Life Technologies #41965-039) containing 10% Fetal Bovine Serum (FBS, Sigma-Aldrich #F9665, LOT 0001655429), 4.5 g/L D-glucose, 100 units/mL Penicillin and 100 µg/mL Streptomycin (Gibco® by Life Technologies #15140-122). The cells were cultivated and treated at 37 °C and 5% CO2 in a humidified atmosphere.

**Antibodies**

Antibodies against p62 (PROGEN #GP62-C), GAPDH (Abcam #ab8345) and LC3 (Cell Signaling Technology #2775) were used. IRDye 800- or IRDye680- conjugated secondary antibodies were purchased from LI-COR Biosciences (#926-32213; #926-68072; #926-68077).

**Cell viability:**

Viability of HeLa wt cells was measured using the resazurin reduction assay (also known as Alamar Blue® assay). HeLa wt cells were seeded in 96-well plates with a density of $3 \times 10^3$ cells/well. One day after seeding, cells were treated with different concentrations (between 0.0977 µM and 50 µM) of the test compounds, 2.5 µM Staurosporine (Biozol #LCL-S-9300) as a positive control or DMSO (PanReac AppliChem, Darmstadt, Germany, #A3672) as a solvent control. After 46 h incubation time, wells were washed once with PBS, filled with growth medium again and resazurin (Sigma #R7017) was added to the cells to a final concentration of 0.02 mg/ml. 96 well-plates were then incubated at 37 °C and 5% $CO_2$ in a humidified atmosphere for 2 h. Absorbance was measured at 560/590 nm with a microplate reader (BioTek, Synergy Mx). After subtraction of the reference signal, each value was normalized to the mean of the absorbance of the solvent control and multiplied by 100. All $IC_{50}$ values were calculated using GraphPad Prism (version 8.0.2).

**Western Blot:**

HeLa wt cells were seeded in 60 mm dishes with a density of $7 \times 10^5$ cells/dish. One day after seeding, cells were treated with 10 µM of the respective test compound, 40 nM Bafilomycin $A_1$ (Sigma-Aldrich #B1793) as a positive control or DMSO as a solvent control in full medium or starvation medium (EBSS, Gibco® by Life Technologies #24010-043) for 6 h. After, cells were harvested by scraping, washed once with ice-cold phosphate-buffered saline (PBS) and lysed in standard ice-cold lysis buffer (20 mM Tris-HCl (pH 7.5), 150 mM NaCl, 0.5 mM EDTA, 1 % (v/v) Triton X-100, protease inhibitor cocktail [Roche, #58698000] and PhosSTOP [Roche, #04906837001]) for 30

min on ice. Lysates were clarified by centrifugation at 13,300 rpm for 15 min at 4 °C. Equal amounts of protein were determined by Bradford method. After adding Lämmli buffer, samples were boiled at 95 °C. Samples were then subjected to SDS-PAGE. Proteins were then transferred to PVDF membranes (Millipore, #IPFL00010) and analyzed using the indicated primary antibodies and appropriate IRDye-conjugated secondary antibodies. Protein signals were detected using an Odyssey Infrared Imaging system (LI-COR Biosciences) and quantified using Image Studio Lite 5.2 (LI-COR Biosciences). The signal intensity of each protein band was divided by the average of the intensity of all bands of this protein. The ratios were normalized to the loading control, and fold changes were calculated by dividing each normalized ratio by the average of the ratios of the control line (solvent control DMSO). The results are shown as mean + standard deviation (SD). Statistical analysis was performed using ordinary one-way ANOVA (corrected by Tukey's multiple comparisons test). Compared treatments are indicated in the corresponding bar diagrams. *P < 0.05, **P < 0.01, ***P < 0.001, ****P < 0.0001. All statistical data were calculated with GraphPad Prism (version 8.0.2).

# 3 NMR Spectra

## 3.1.1 Biphenol

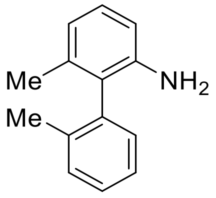


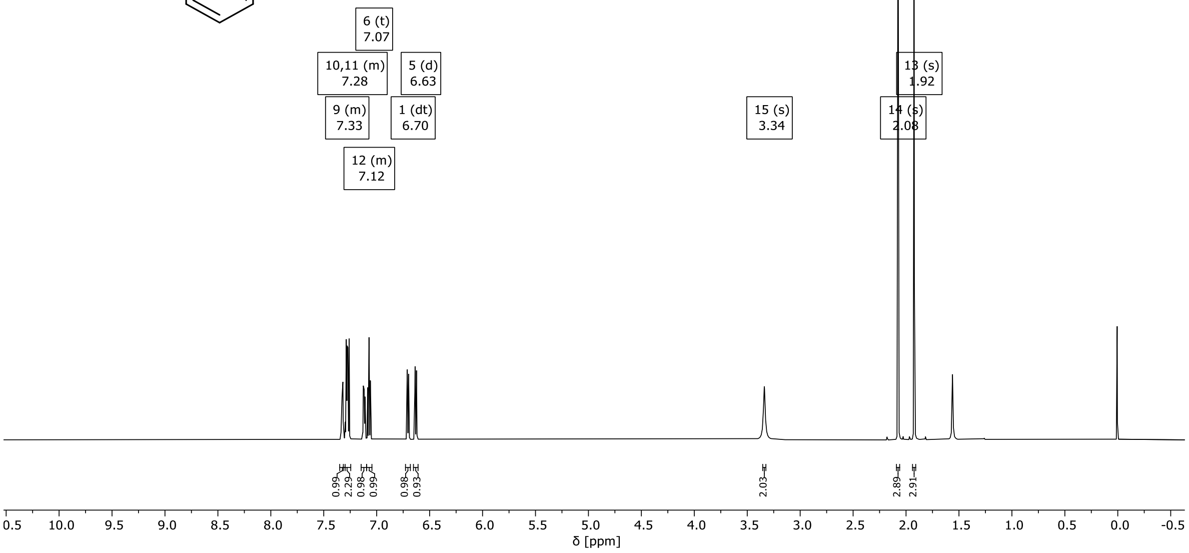


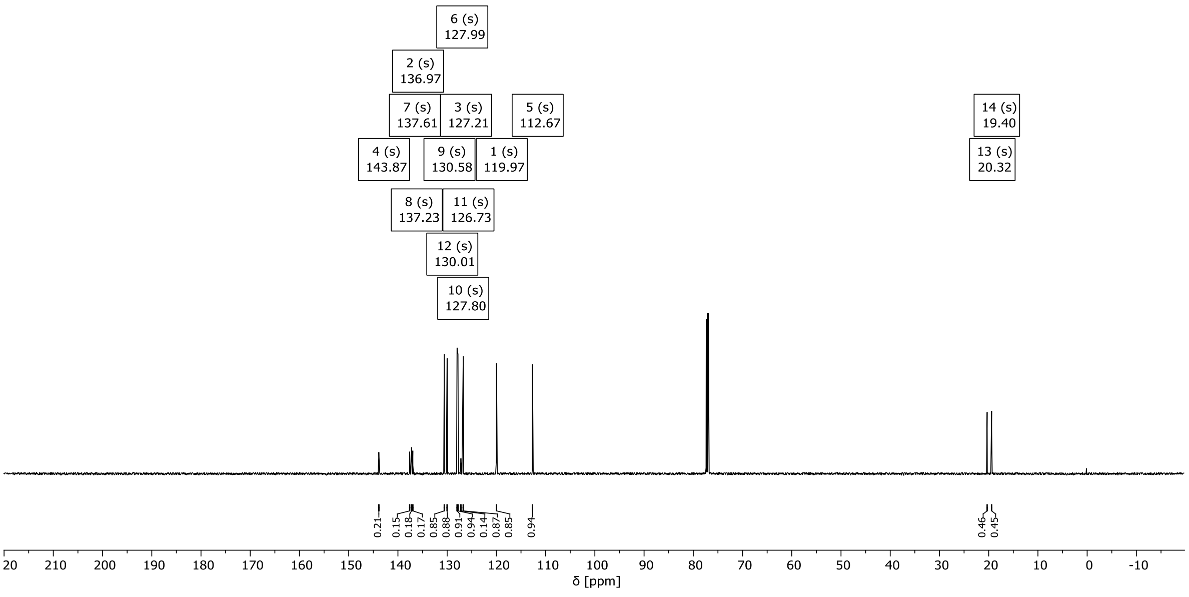


Figure S37: $^{1}H$- and $^{13}C$-NMR spectra (600 / 151 MHz, $CDCl_3$) of 2',6-dimethyl-[1,1'-biphenyl]-2-amine (**5**).

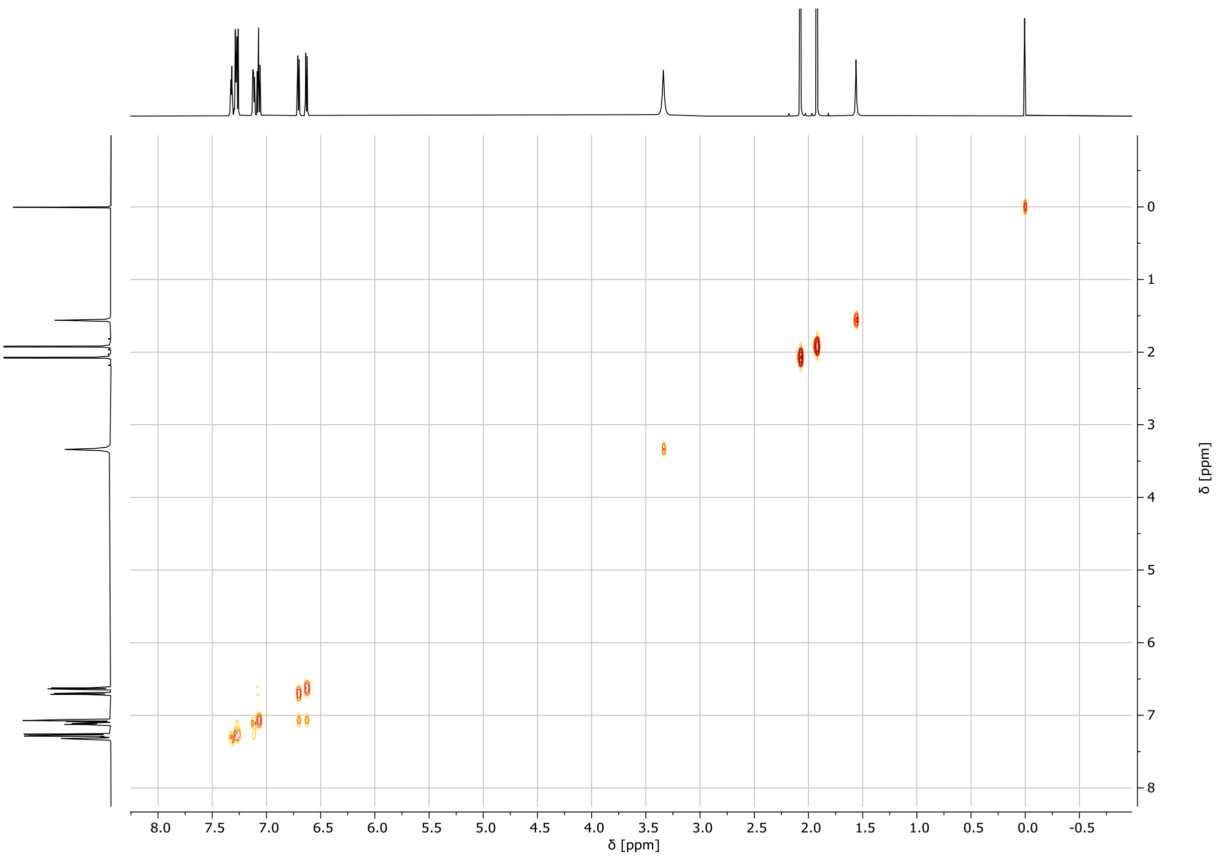


Figure S38: COSY spectrum ($CDCl_3$) of 2',6-dimethyl-[1,1'-biphenyl]-2-amine (**5**).

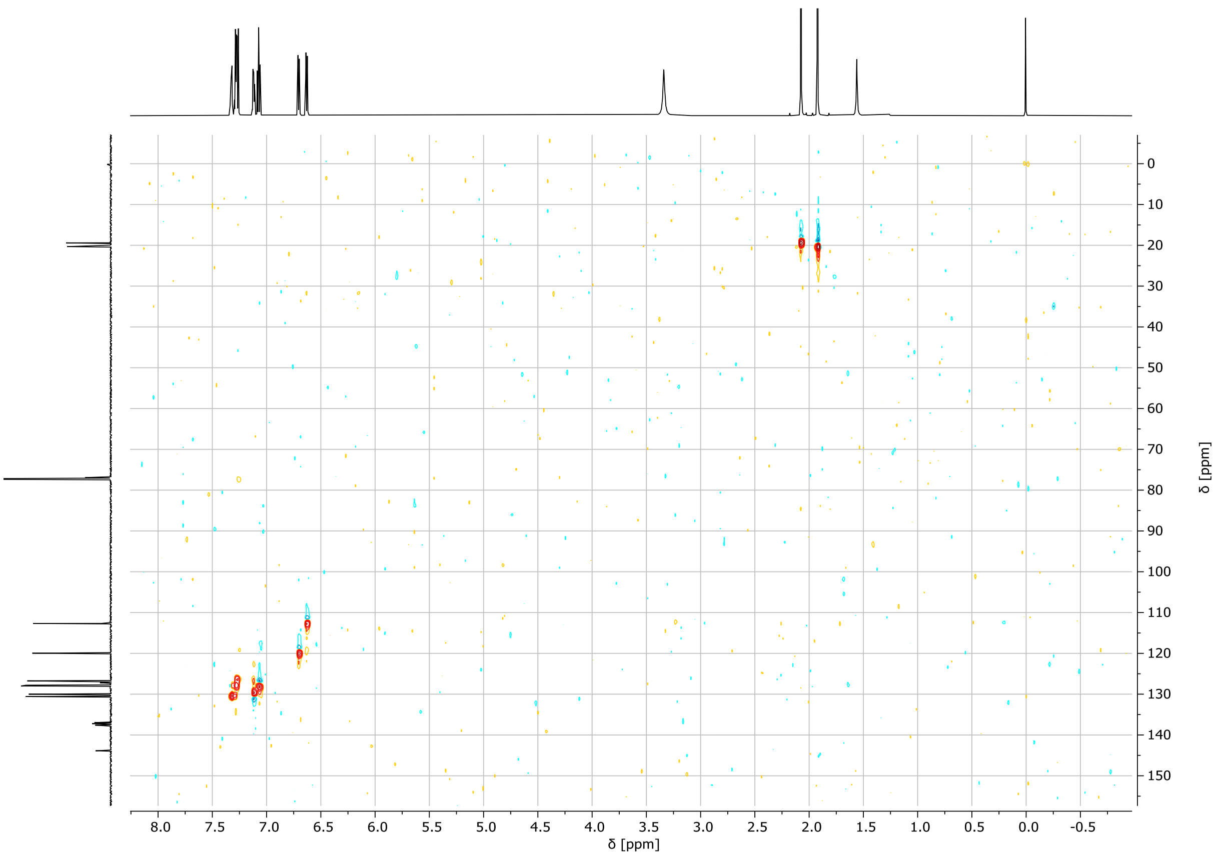


Figure S39: HSQC spectrum ($CDCl_3$) of 2',6-dimethyl-[1,1'-biphenyl]-2-amine (**5**).

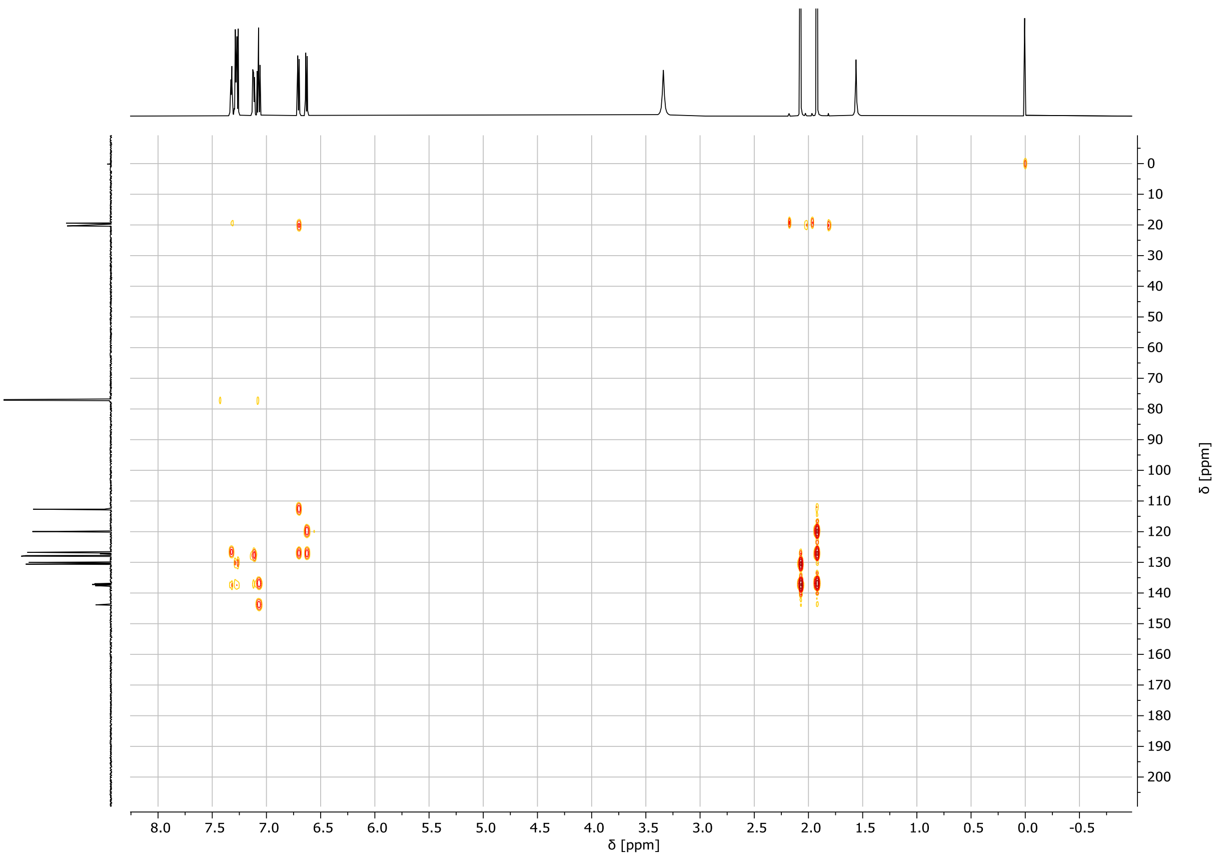


Figure S40: HMBC spectrum ($CDCl_3$) of 2',6-dimethyl-[1,1'-biphenyl]-2-amine (**5**).

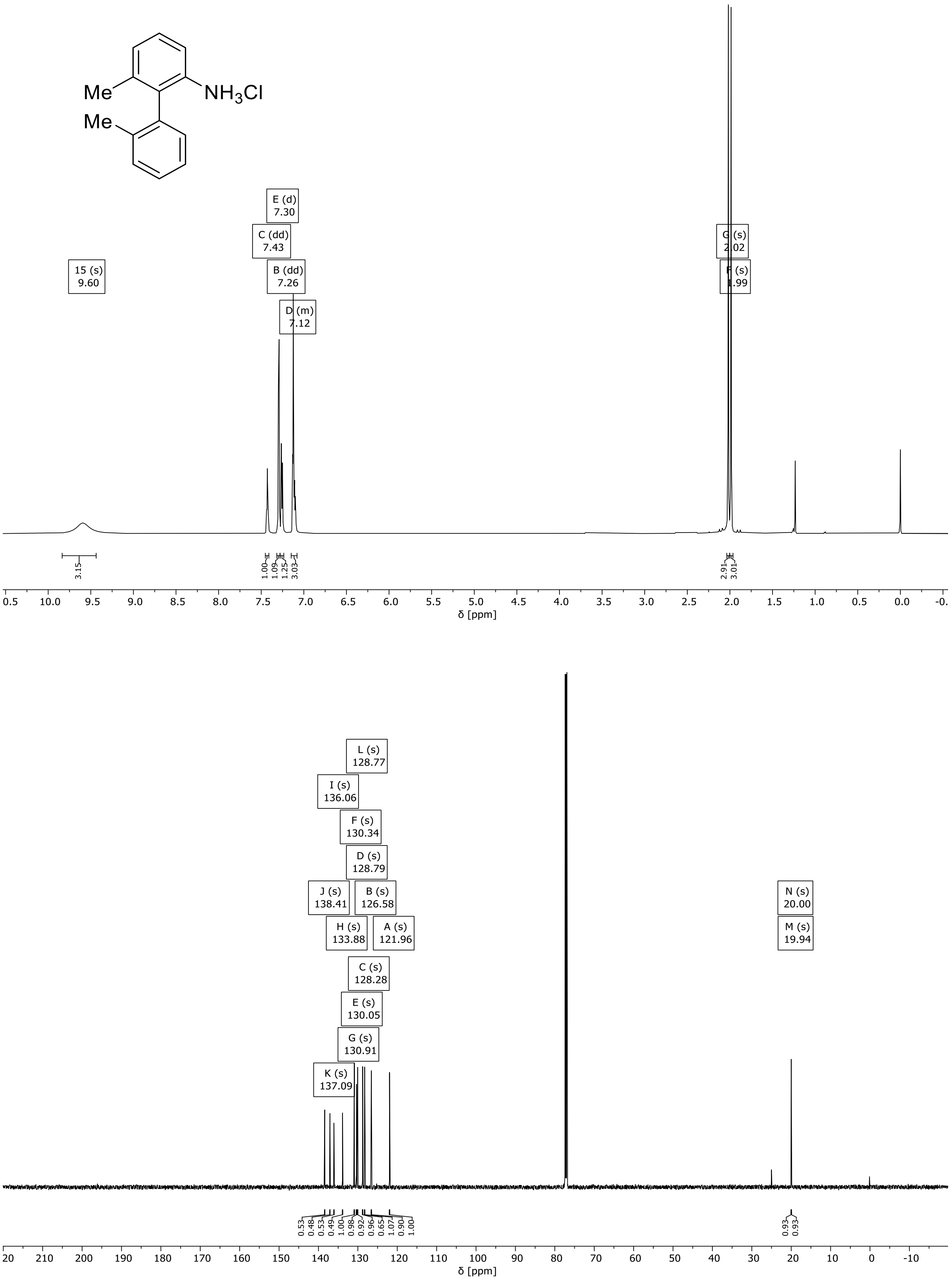


Figure S41: $^{1}$H- and $^{13}$C-NMR spectra (600 / 151 MHz, $CDCl_3$) of 2',6-dimethyl-[1,1'-biphenyl]-2-aminium chloride (**6**).

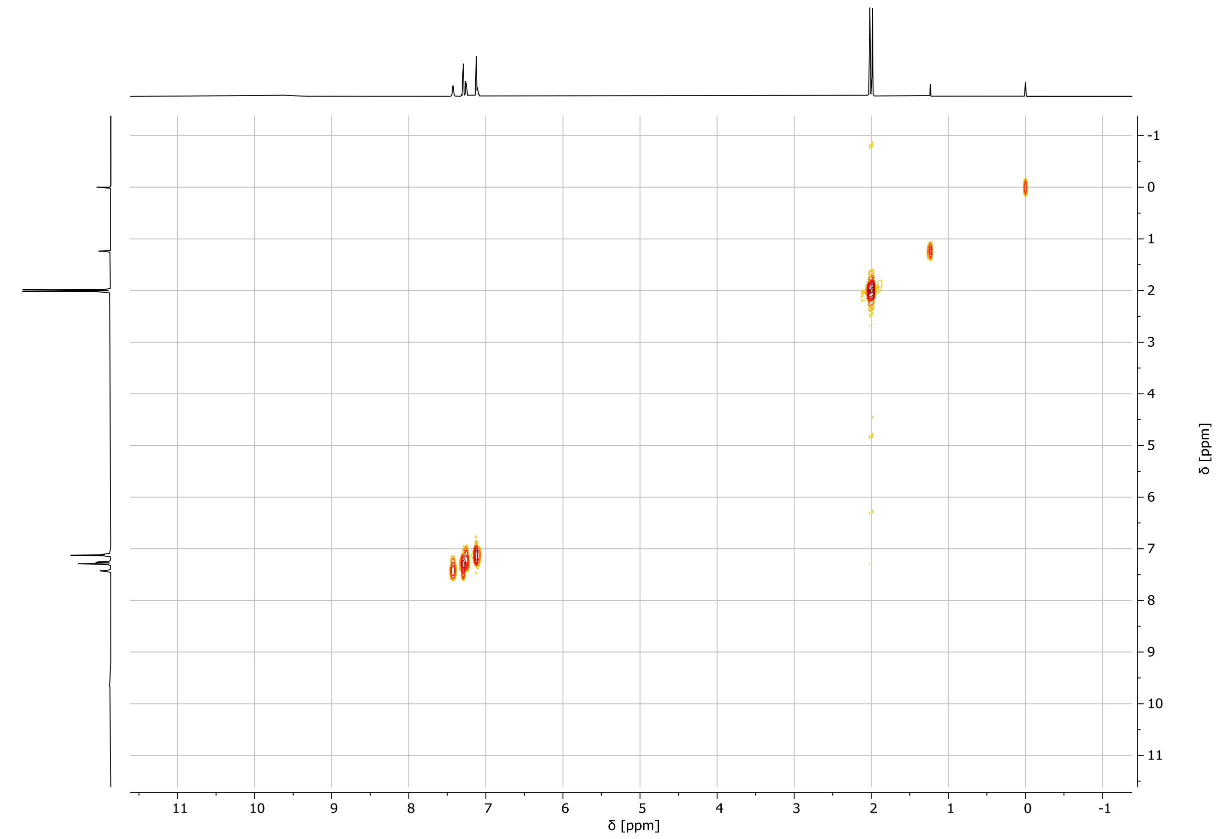


Figure S42: COSY spectrum ($CDCl_3$) of 2',6-dimethyl-[1,1'-biphenyl]-2-aminium chloride (**6**).

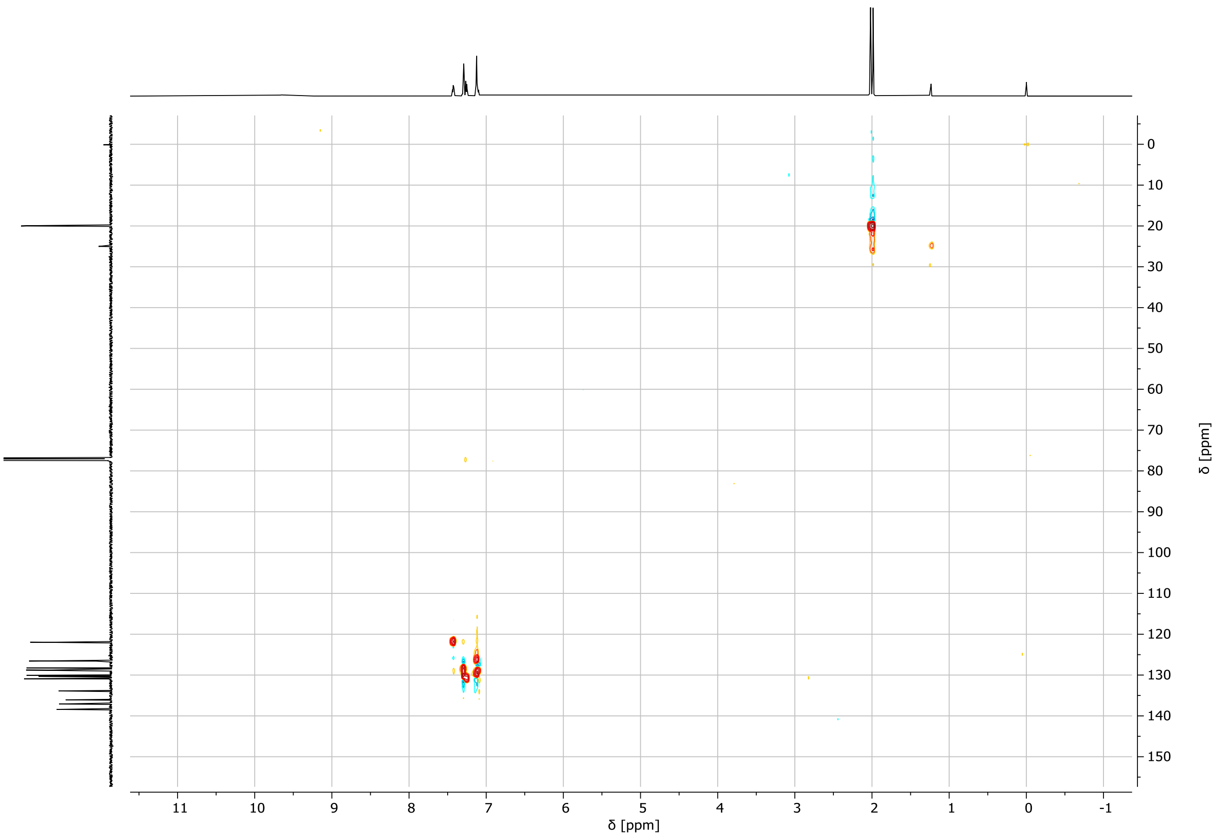


Figure S43: HSQC spectrum ($CDCl_3$) of 2',6-dimethyl-[1,1'-biphenyl]-2-aminium chloride (**6**).

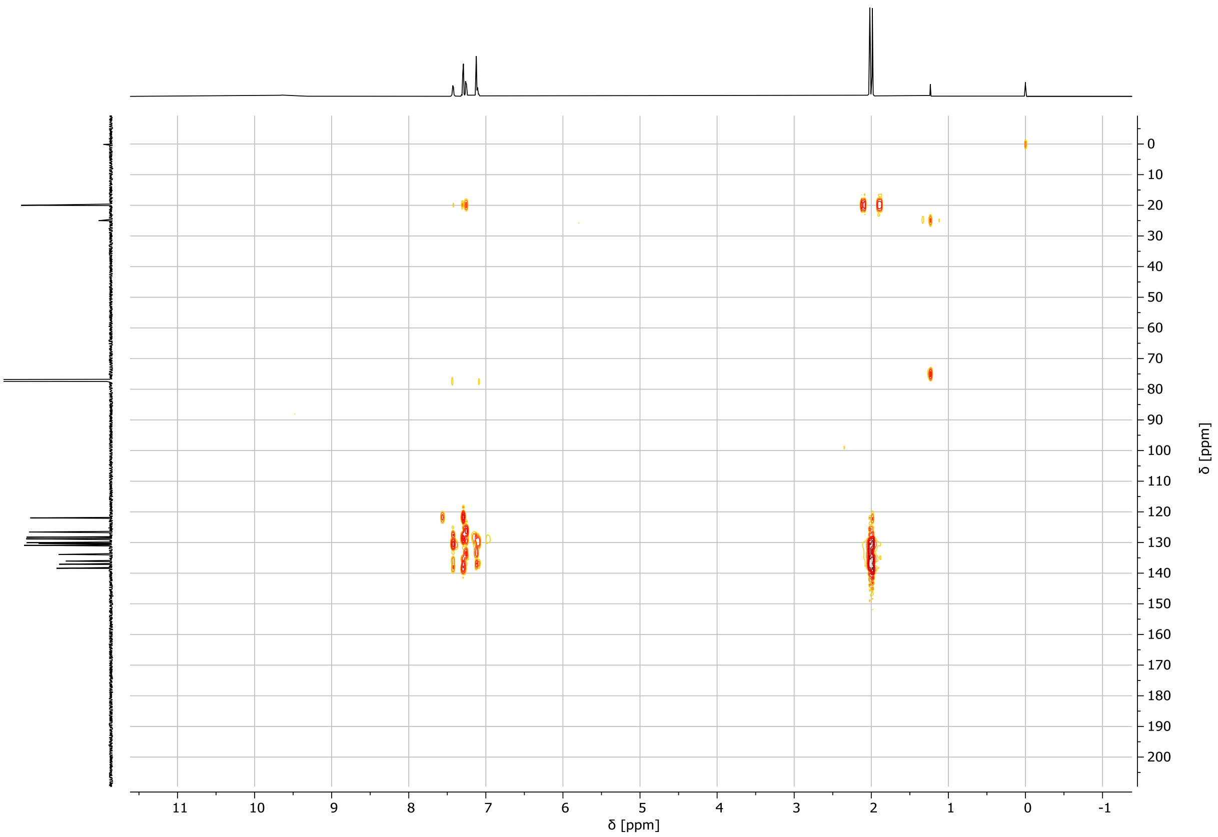


Figure S44: HMBC spectrum ($CDCl_3$) of 2',6-dimethyl-[1,1'-biphenyl]-2-aminium chloride (**6**).

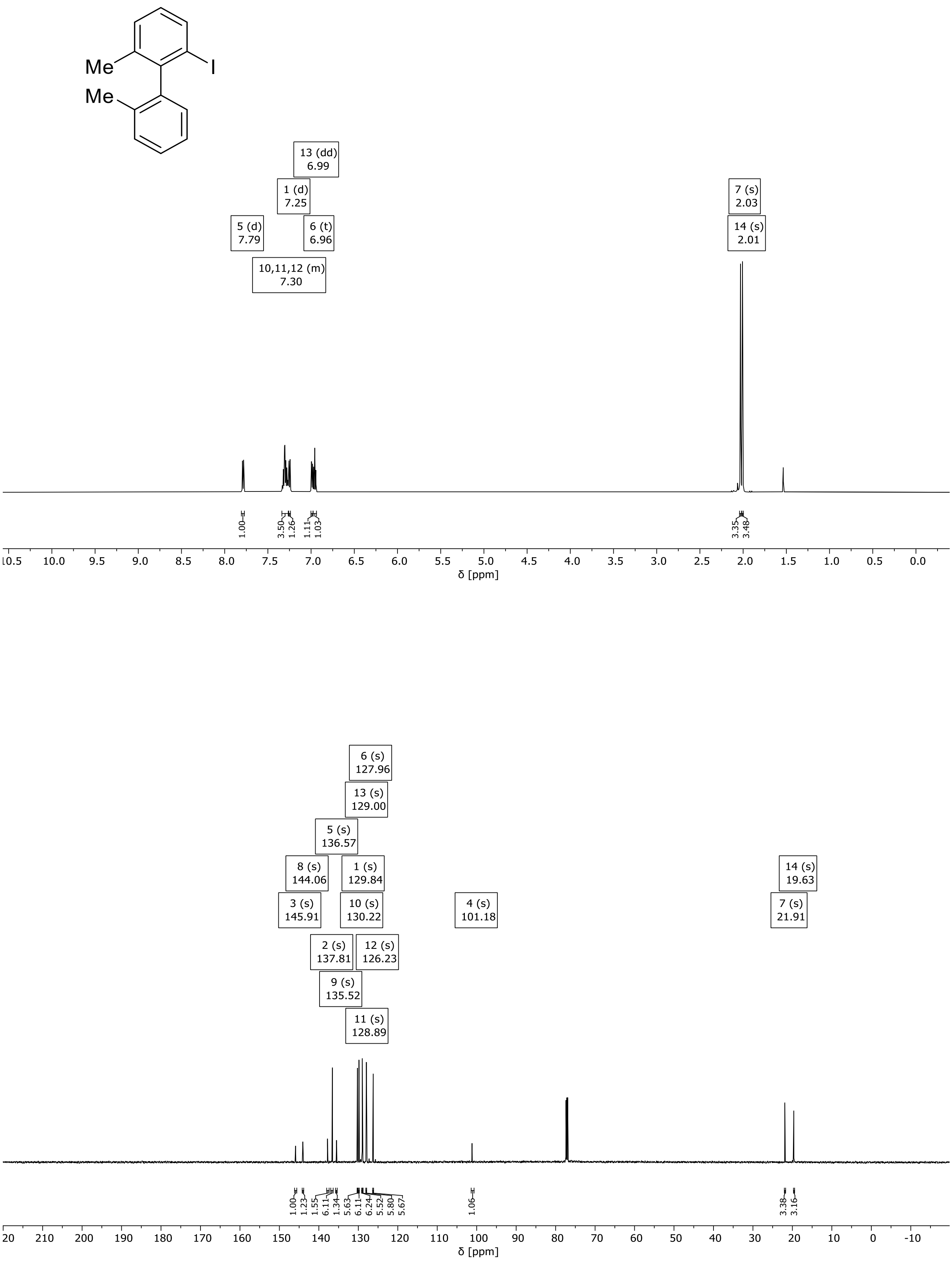


Figure S45: $^{1}$H- and $^{13}$C-NMR spectra (600 / 151 MHz, $CDCl_3$) of 2-iodo-2',6-dimethyl-1,1'-biphenyl (**7**).

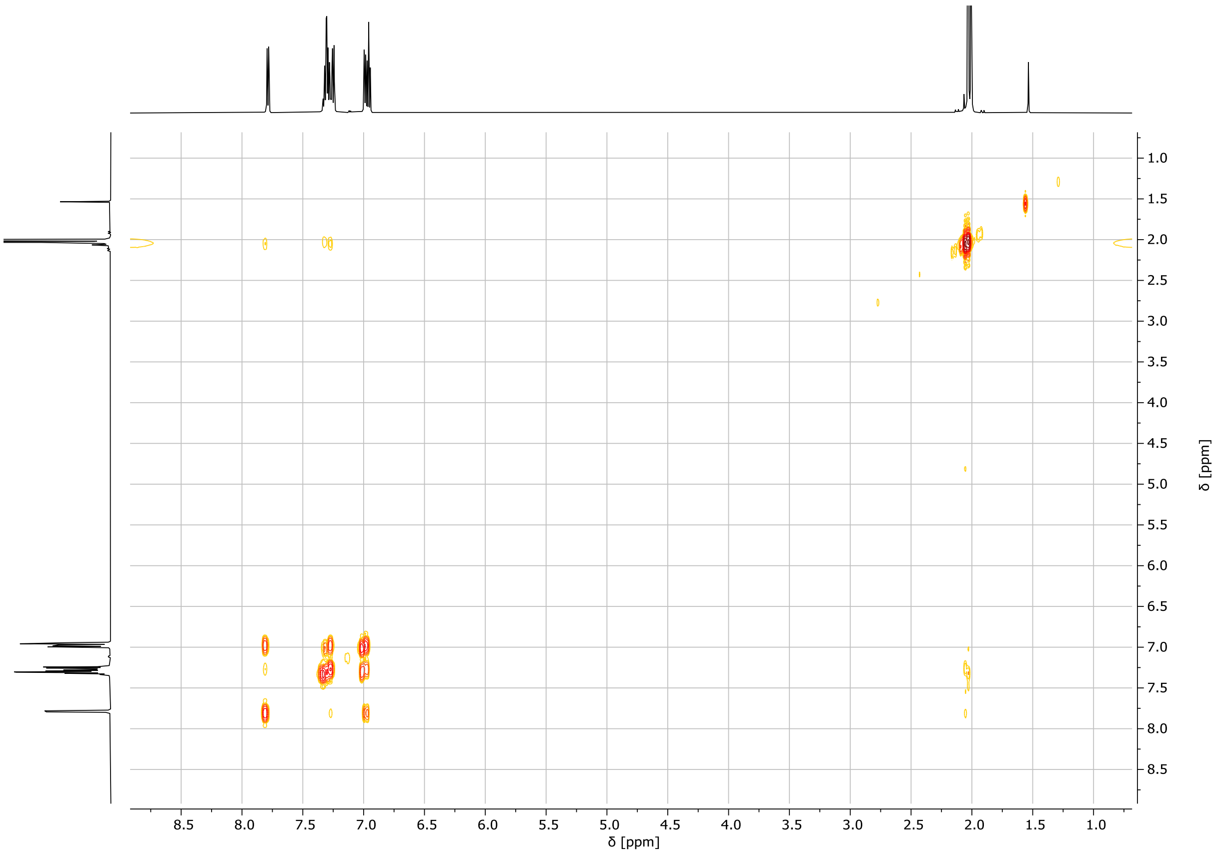


Figure S46: COSY spectrum ($CDCl_3$) of 2-iodo-2',6-dimethyl-1,1'-biphenyl (**7**).

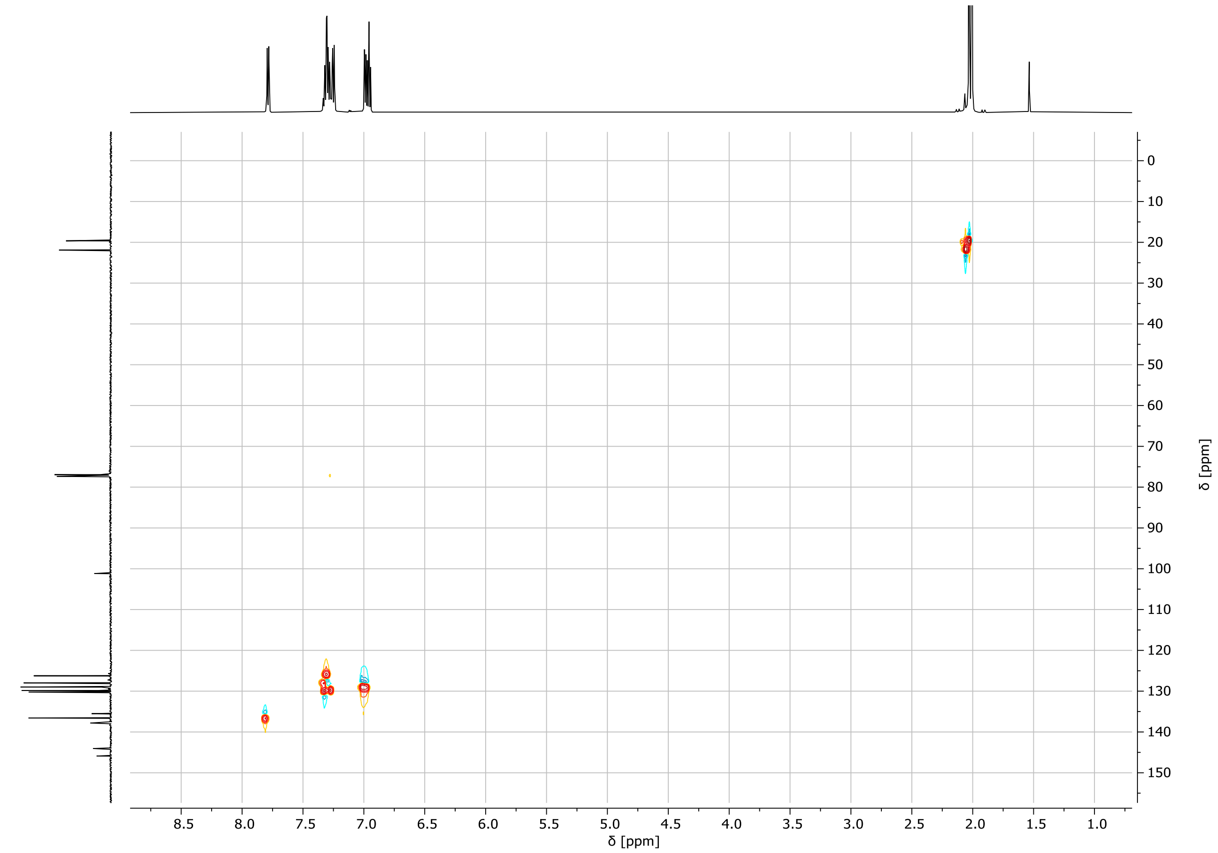


Figure S47: HSQC spectrum ($CDCl_3$) of 2-iodo-2',6-dimethyl-1,1'-biphenyl (**7**).

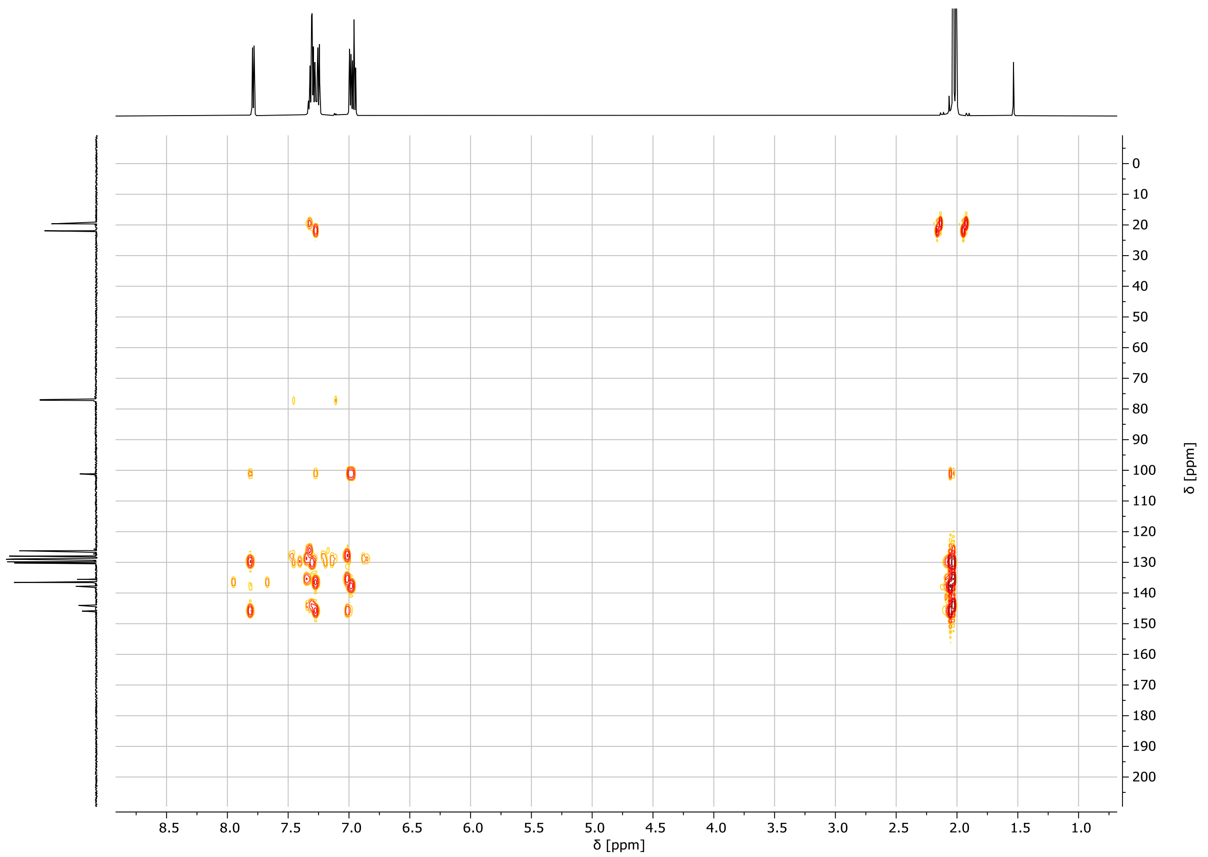


Figure S48: HMBC spectrum ($CDCl_3$) of 2-iodo-2',6-dimethyl-1,1'-biphenyl (**7**).

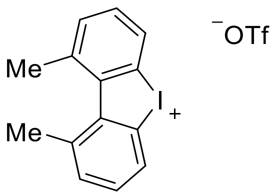
Me
Me
I+
⁻OTf

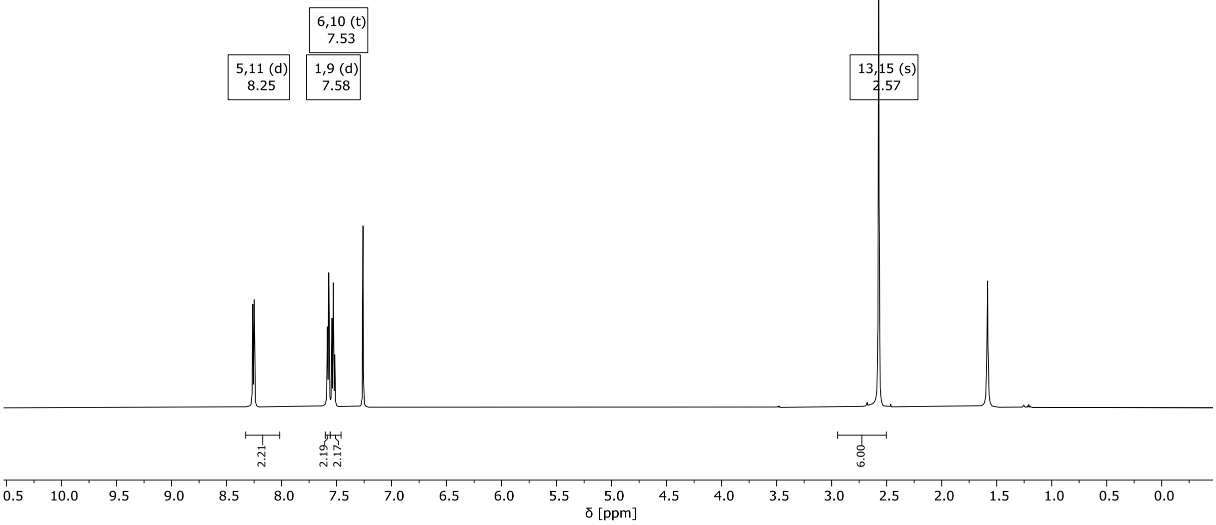
6,10 (t)
7.53
5,11 (d)
8.25
1,9 (d)
7.58
13,15 (s)
2.57
2.21
2.19
2.17
6.00
δ [ppm]

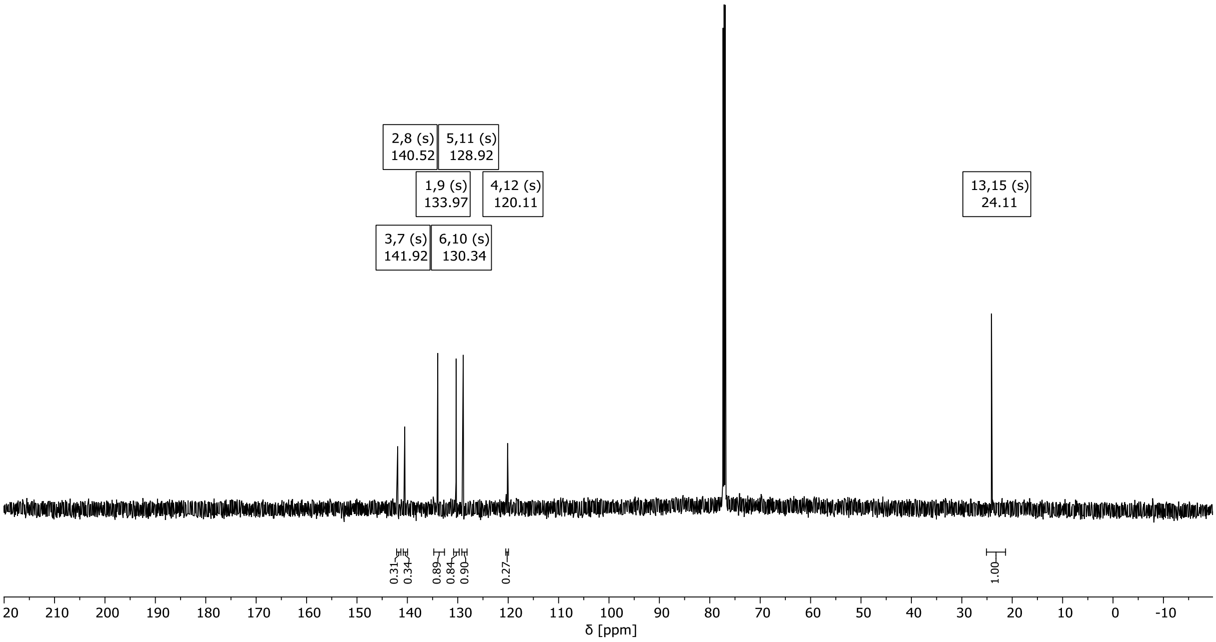
2,8 (s)
140.52
5,11 (s)
128.92
1,9 (s)
133.97
4,12 (s)
120.11
13,15 (s)
24.11
3,7 (s)
141.92
6,10 (s)
130.34
0.31
0.34
0.89
0.84
0.90
0.27
1.00
δ [ppm]

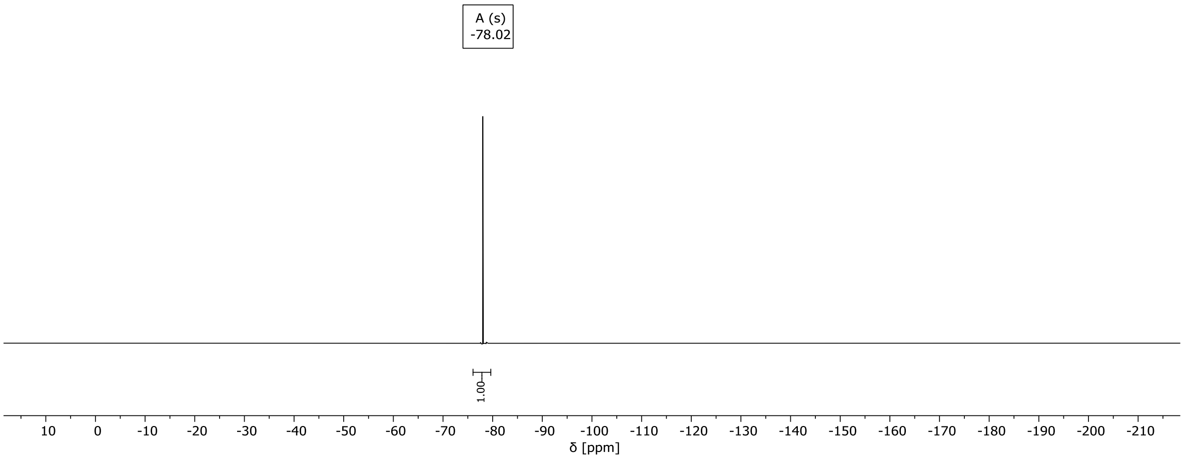


Figure S49: $^{1}H$-, $^{13}C$- and $^{19}F$-NMR spectra (600 / 151 / 282 MHz, $CDCl_3$) of 1,9-dimethyldibenzo[b,d]iodol-5-ium trifluoromethanesulfonate (**8**).

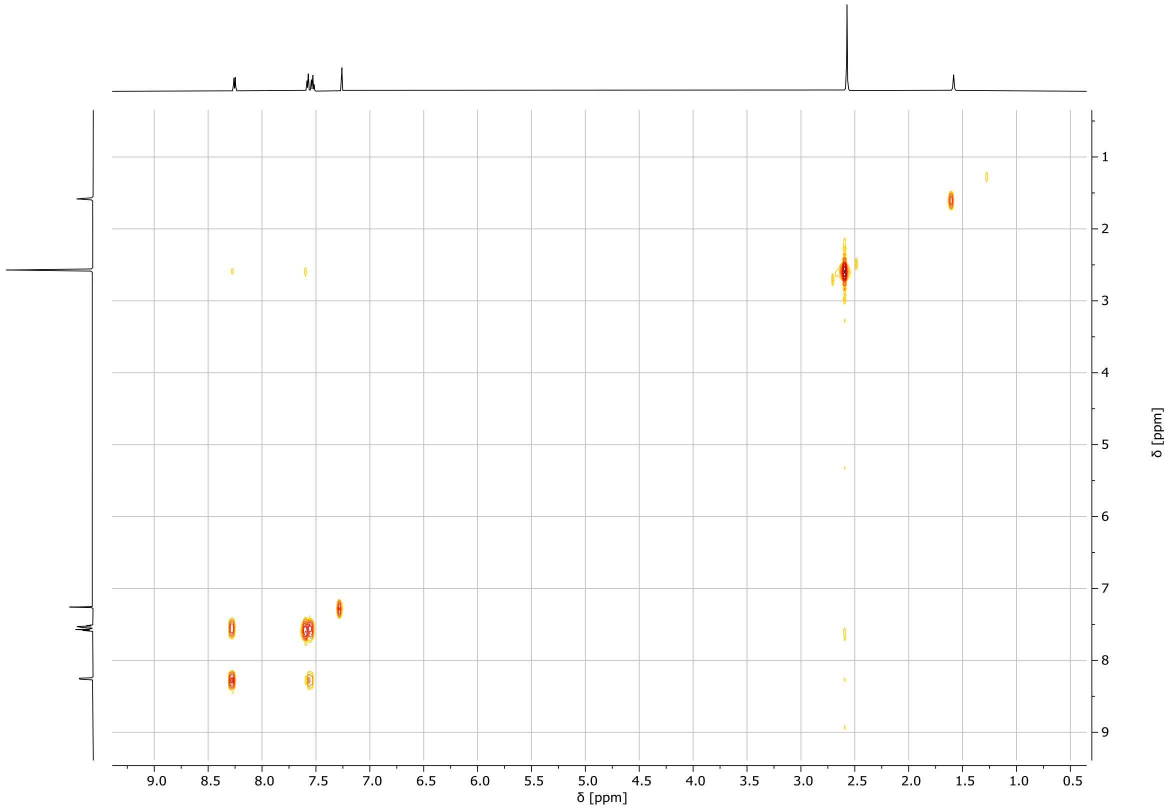


Figure S50: COSY spectrum ($CDCl_3$) of 1,9-dimethyldibenzo[b,d]iodol-5-ium trifluoromethanesulfonate (**8**).

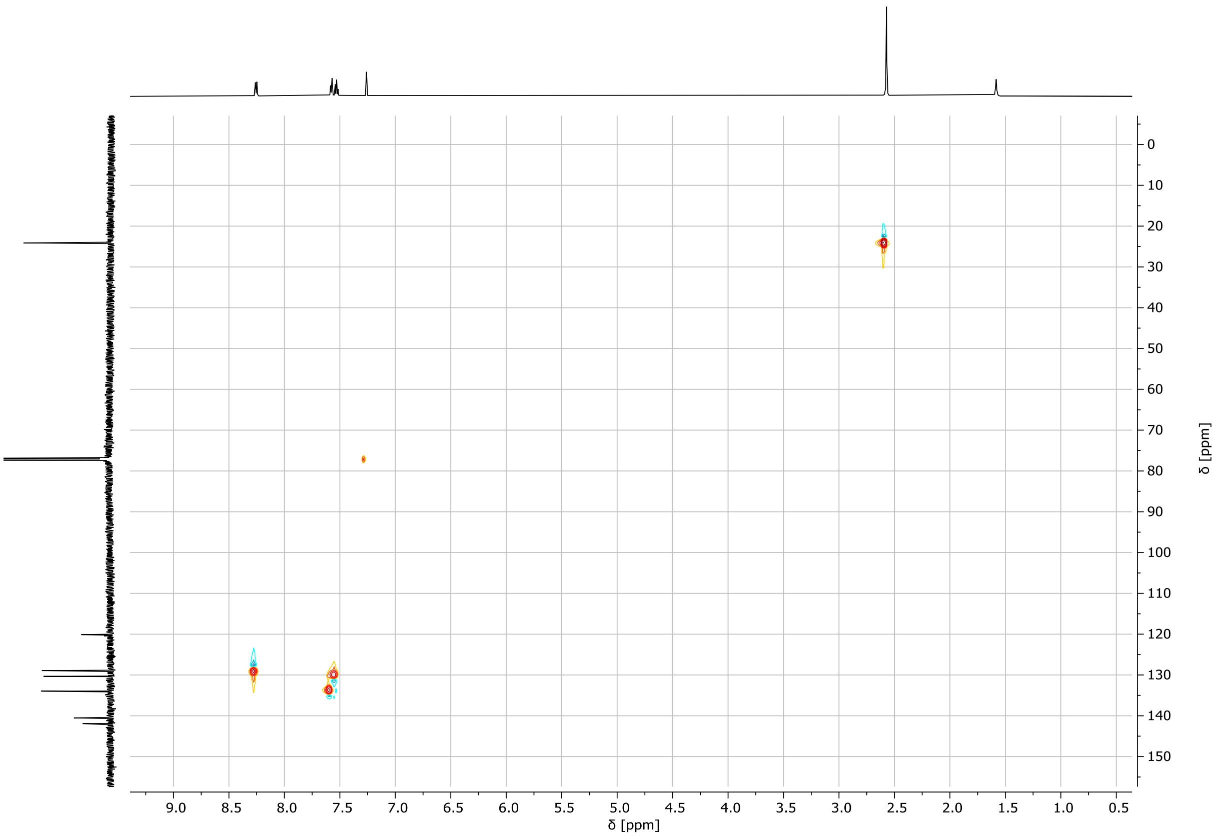


Figure S51: HSQC spectrum ($CDCl_3$) of 1,9-dimethyldibenzo[b,d]iodol-5-ium trifluoromethanesulfonate (**8**).

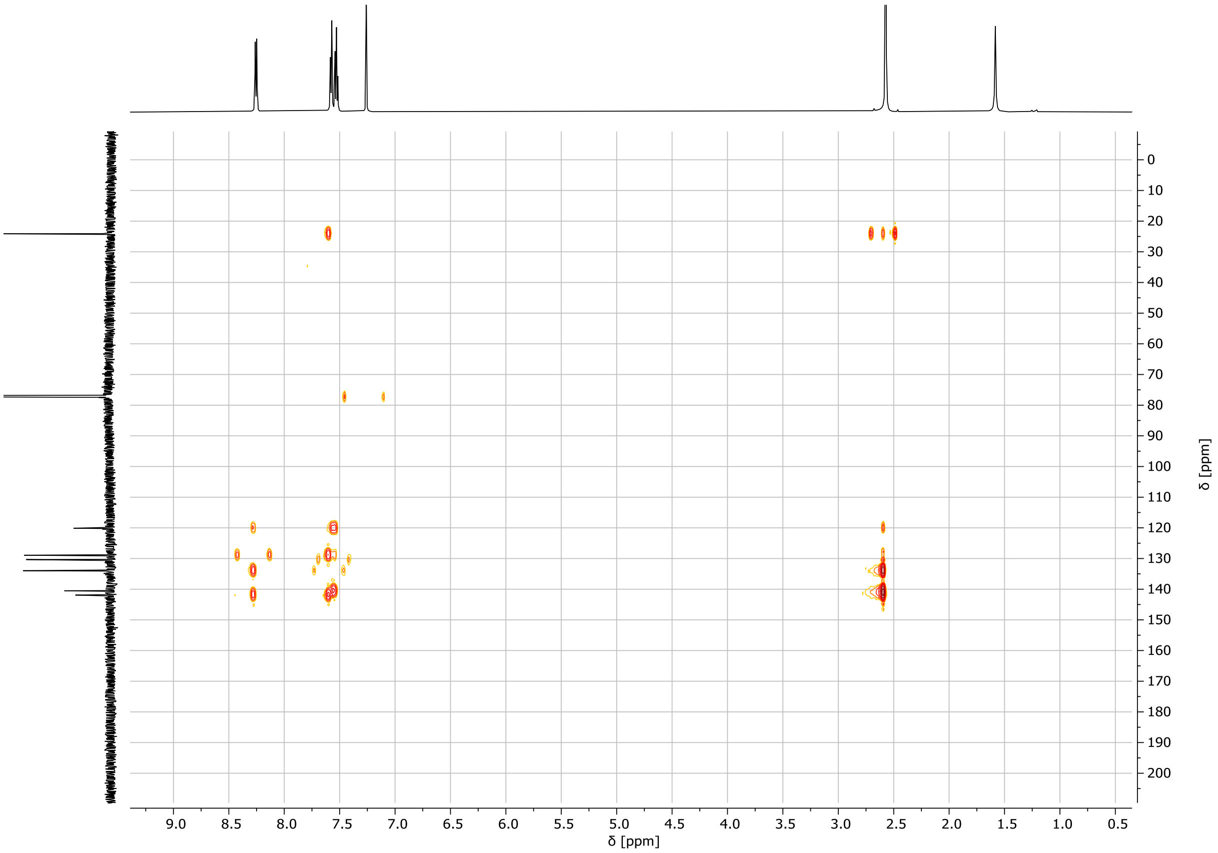


Figure S52: HMBC spectrum ($CDCl_3$) of 1,9-dimethyldibenzo[b,d]iodol-5-ium trifluoromethanesulfonate (**8**).

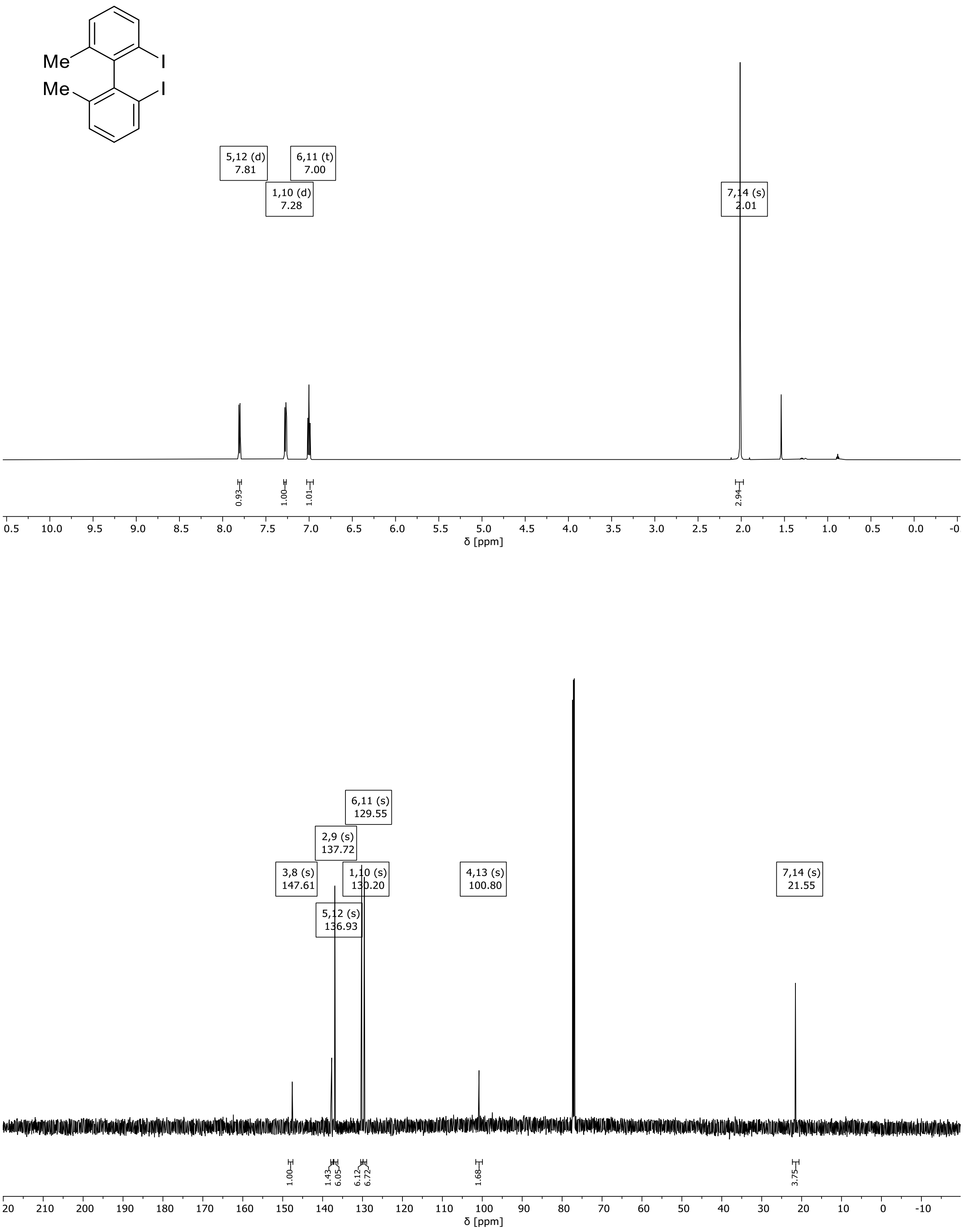


Figure S53: $^{1}$H- and $^{13}$C-NMR spectra (600 / 151 MHz, $CDCl_3$) of 2,2'-diiodo-6,6'-dimethyl-1,1'-biphenyl (**4**).

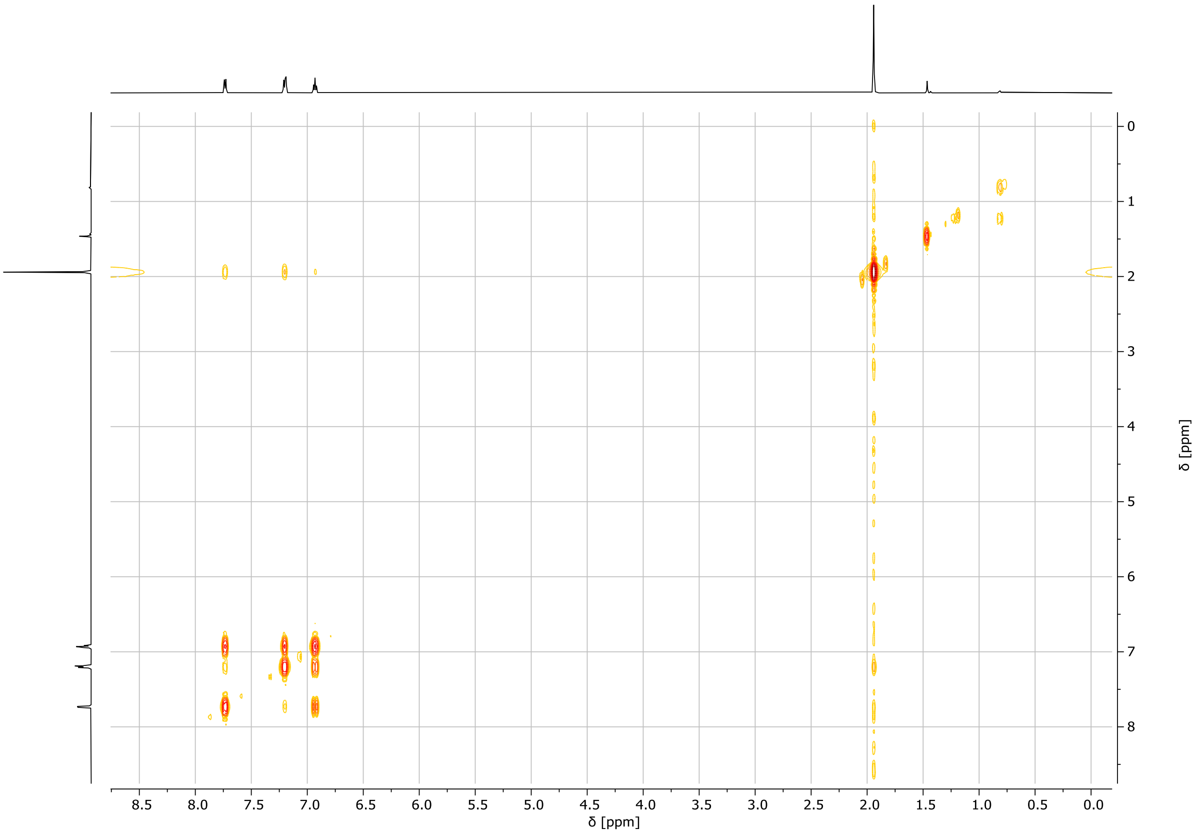


Figure S54: COSY spectrum ($CDCl_3$) of 2,2'-diiodo-6,6'-dimethyl-1,1'-biphenyl (**4**).

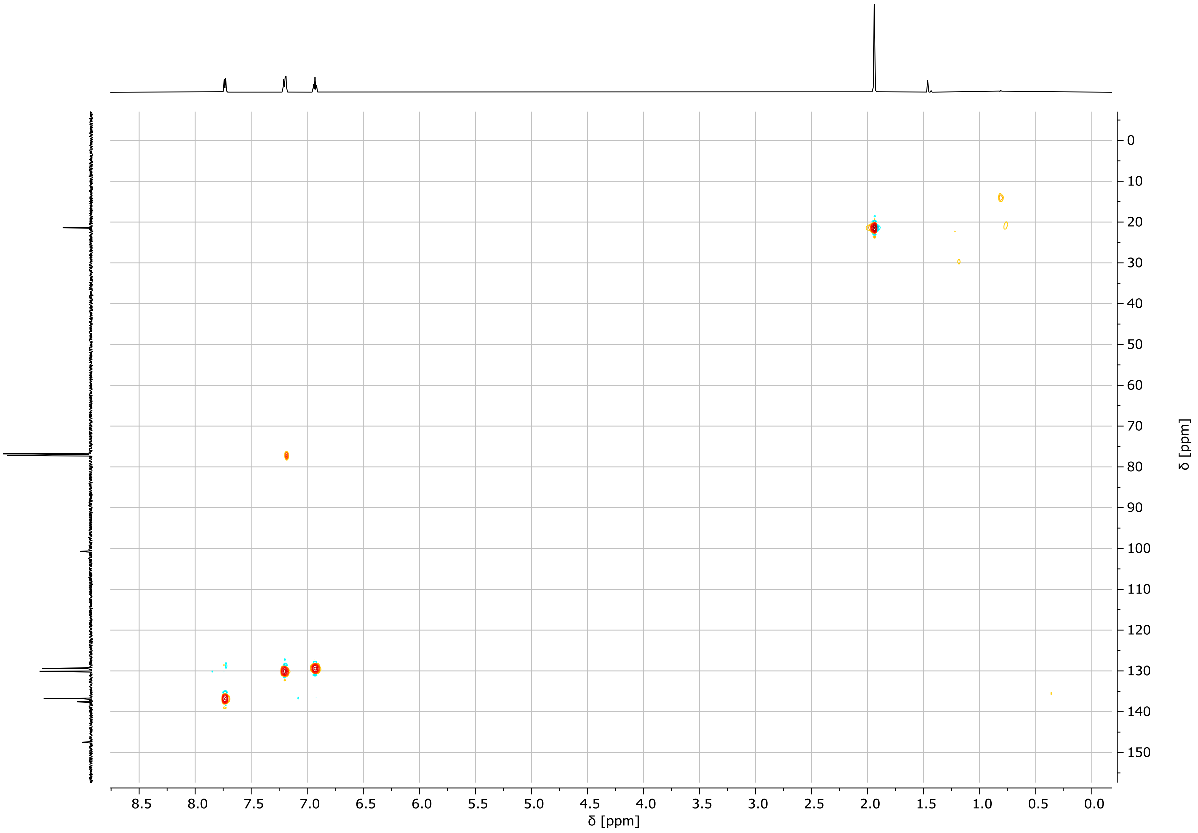


Figure S55: HSQC spectrum ($CDCl_3$) of 2,2'-diiodo-6,6'-dimethyl-1,1'-biphenyl (**4**).

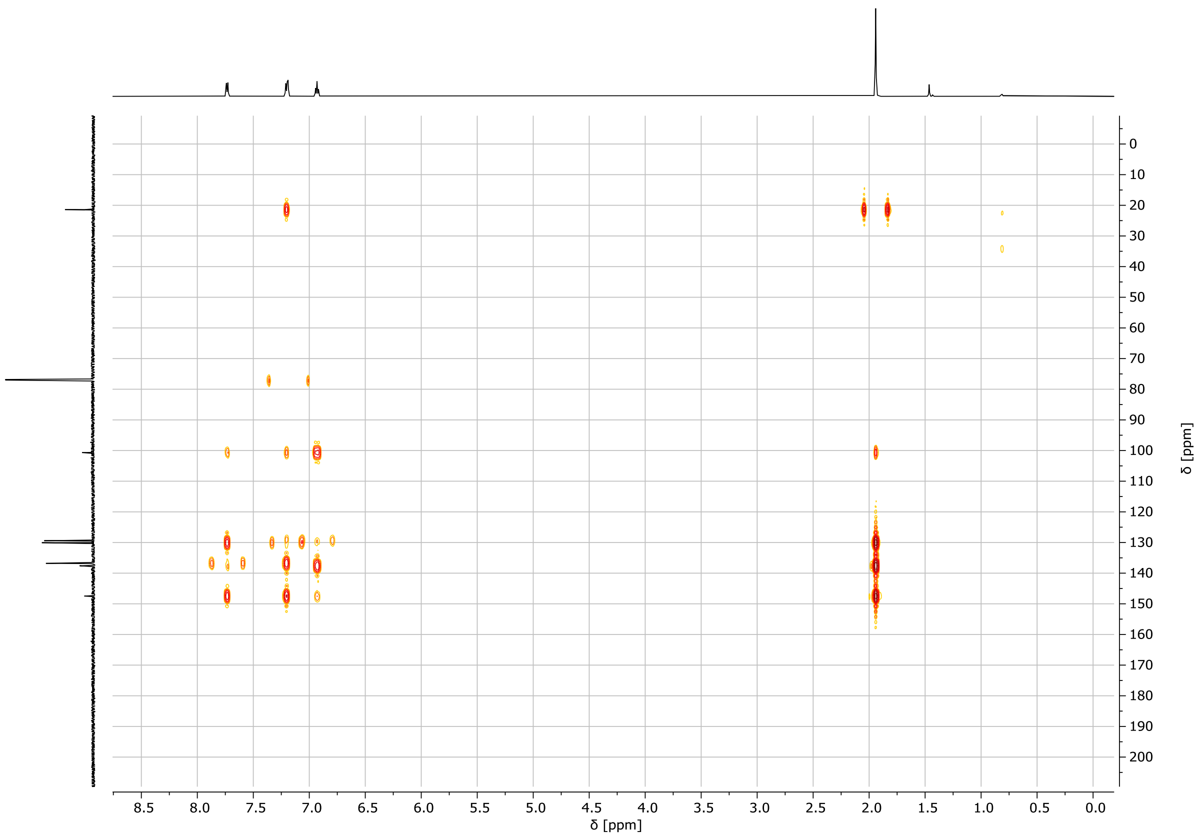


Figure S56: HMBC spectrum ($CDCl_3$) of 2,2'-diiodo-6,6'-dimethyl-1,1'-biphenyl (**4**).

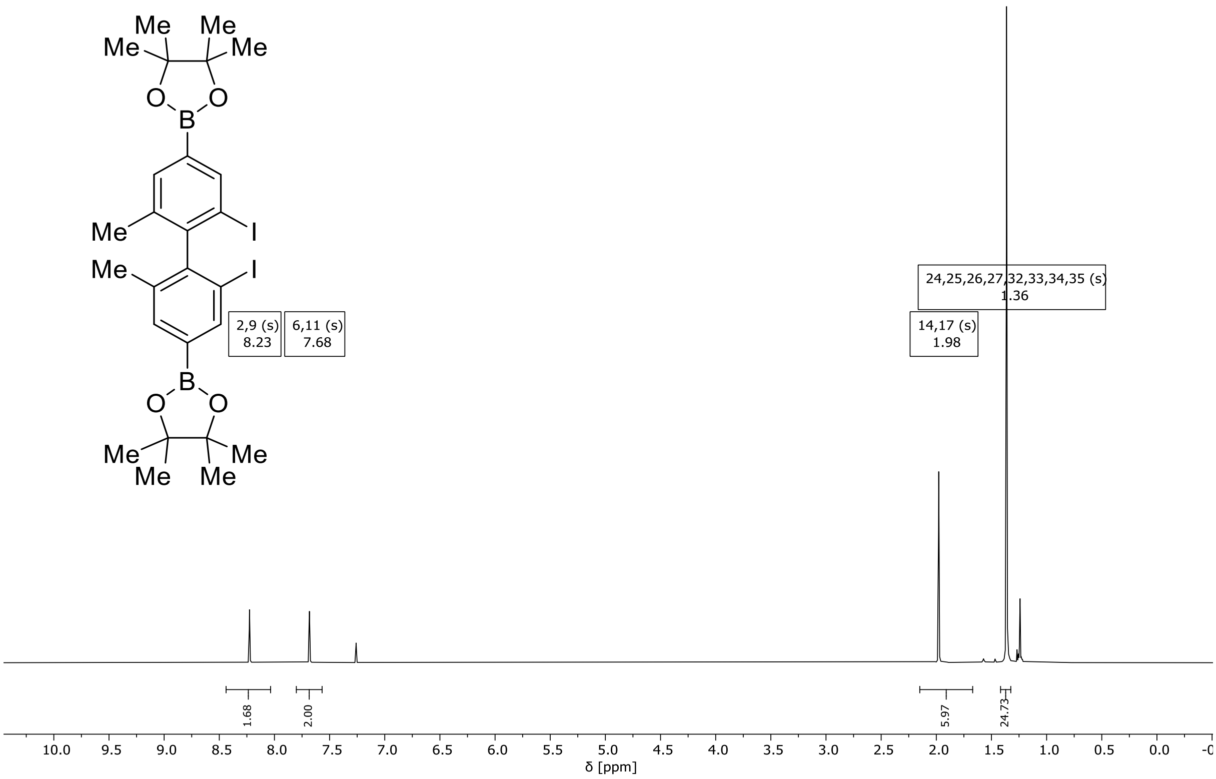

2,9 (s)
8.23
6,11 (s)
7.68
14,17 (s)
1.98
24,25,26,27,32,33,34,35 (s)
1.36
1.68
2.00
5.97
24.73
δ [ppm]


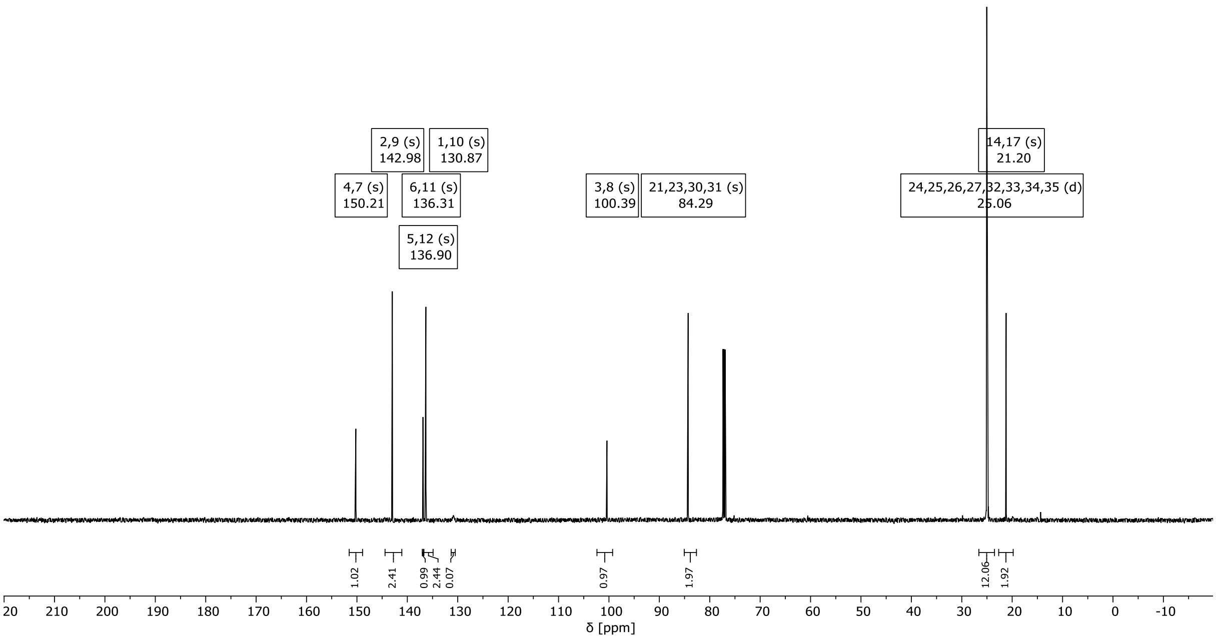

2,9 (s)
142.98
1,10 (s)
130.87
4,7 (s)
150.21
6,11 (s)
136.31
5,12 (s)
136.90
3,8 (s)
100.39
21,23,30,31 (s)
84.29
14,17 (s)
21.20
24,25,26,27,32,33,34,35 (d)
25.06
1.02
2.41
0.99
2.44
0.07
0.97
1.97
12.06
1.92
δ [ppm]

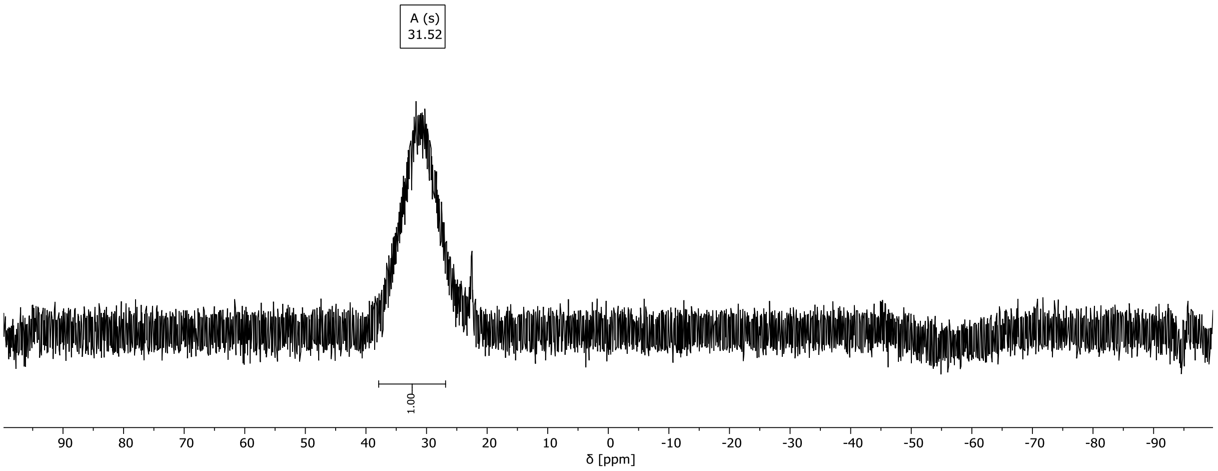


Figure S57: $^{1}H$-, $^{13}C$- and $^{11}B$-NMR spectra (600 / 151 / 96 MHz, $CDCl_3$) of 2,2'-(2,2'-diiodo-6,6'-dimethyl-[1,1'-biphenyl]-4,4'-diyl)bis(4,4,5,5-tetramethyl-1,3,2-dioxaborolane) (**9**).

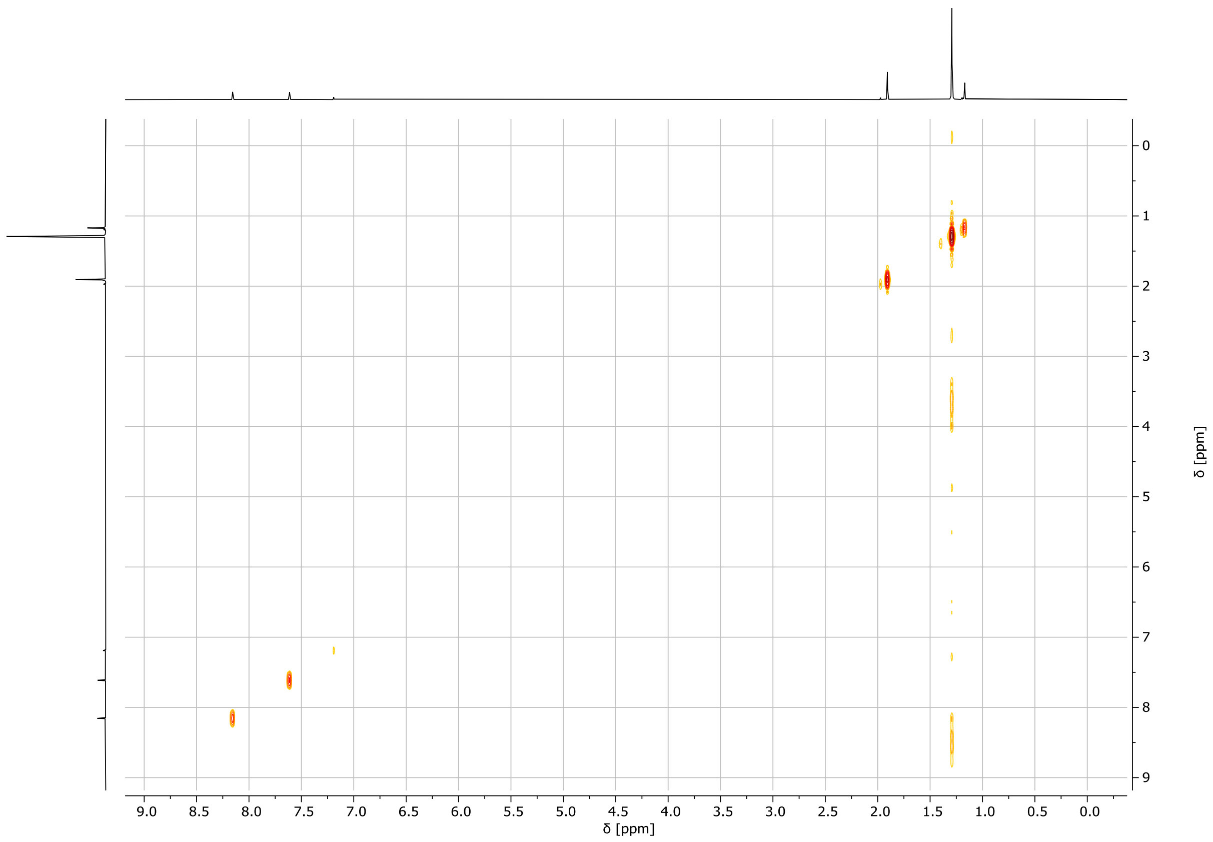


Figure S58: COSY spectrum ($CDCl_3$) of 2,2'-(2,2'-diiodo-6,6'-dimethyl-[1,1'-biphenyl]-4,4'-diyl)bis(4,4,5,5-tetramethyl-1,3,2-dioxaborolane) (**9**).

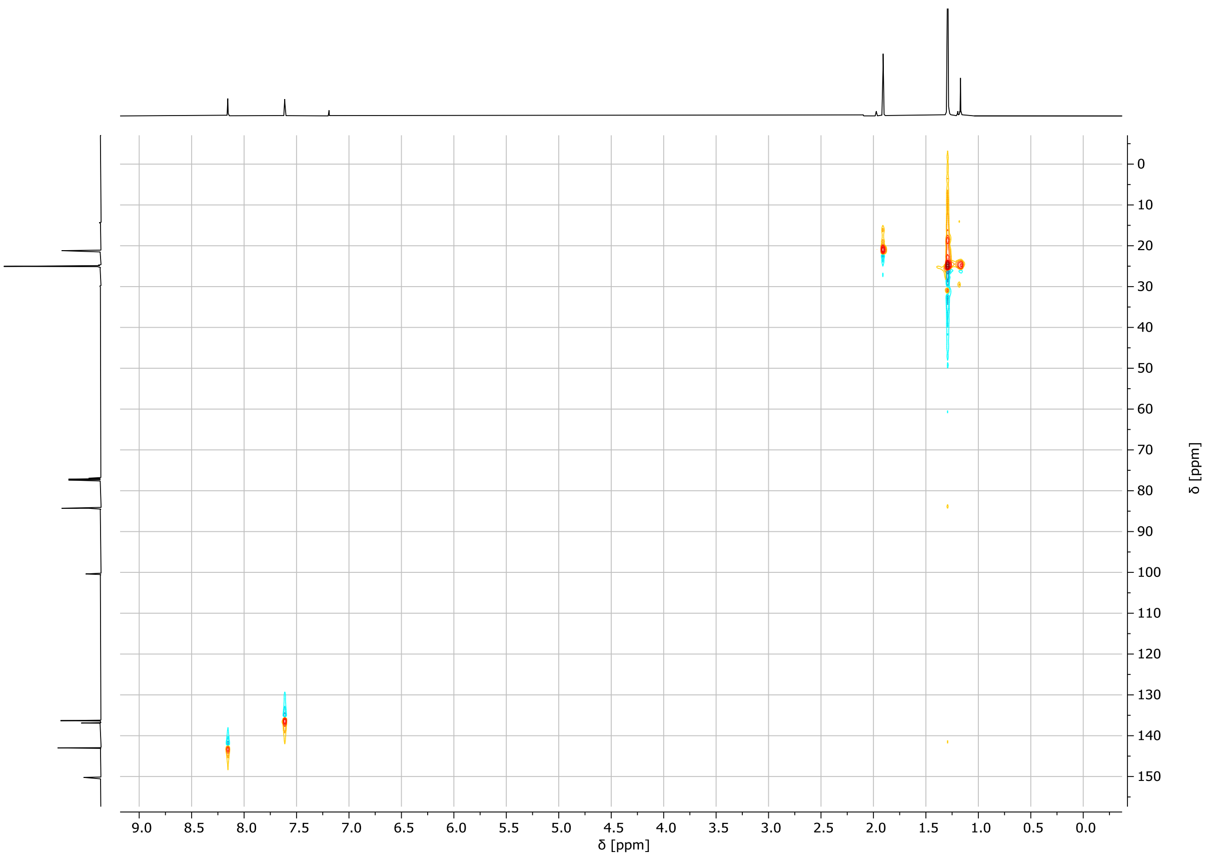


Figure S59: HSQC spectrum ($CDCl_3$) of 2,2'-(2,2'-diiodo-6,6'-dimethyl-[1,1'-biphenyl]-4,4'-diyl)bis(4,4,5,5-tetramethyl-1,3,2-dioxaborolane) (**9**).

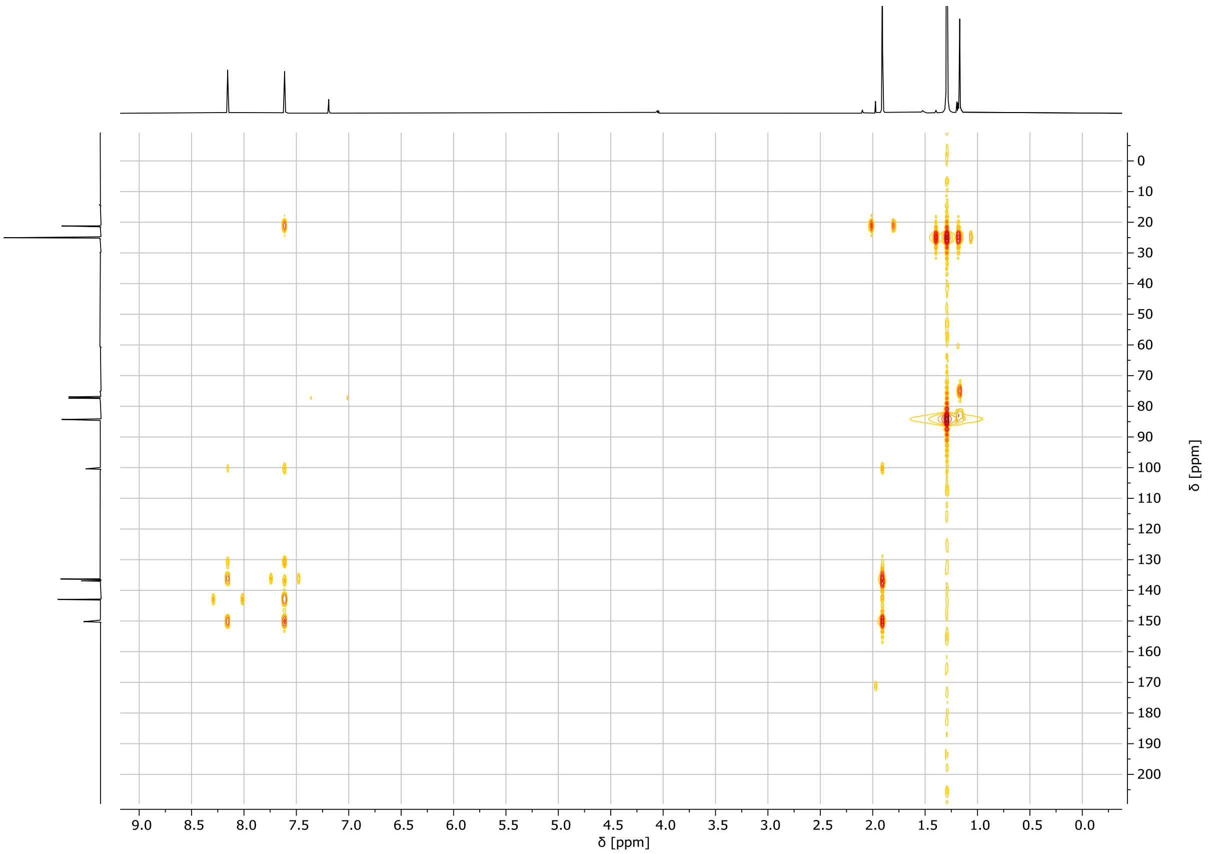


Figure S60: HMBC spectrum ($CDCl_3$) of 2,2'-(2,2'-diiodo-6,6'-dimethyl-[1,1'-biphenyl]-4,4'-diyl)bis(4,4,5,5-tetramethyl-1,3,2-dioxaborolane) (**9**).

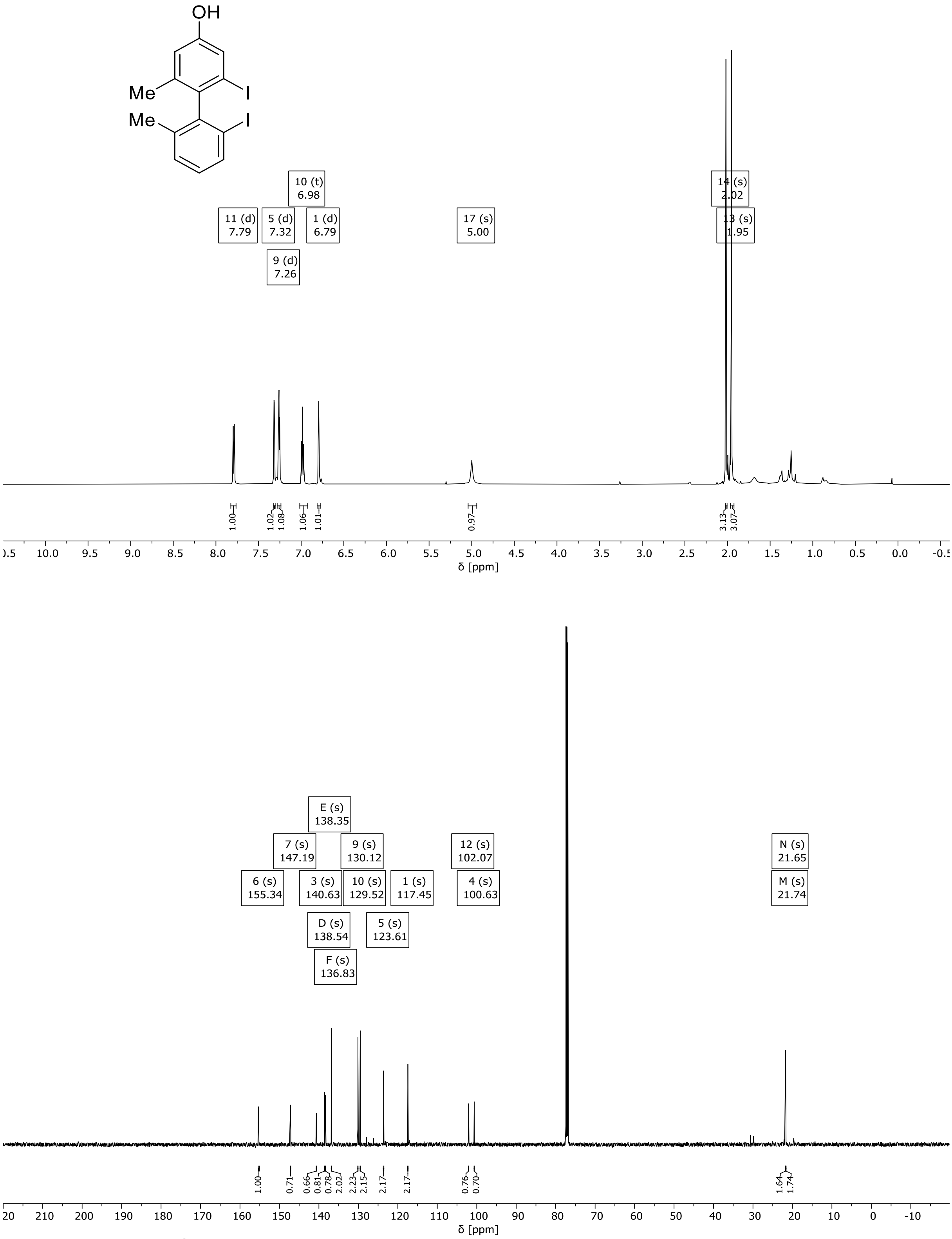


Figure S61: $^{1}$H- and $^{13}$C-NMR spectra (600 / 151 MHz, $CDCl_3$) of 2,2'-diiodo-6,6'-dimethyl-[1,1'-biphenyl]-4-ol (**24**).

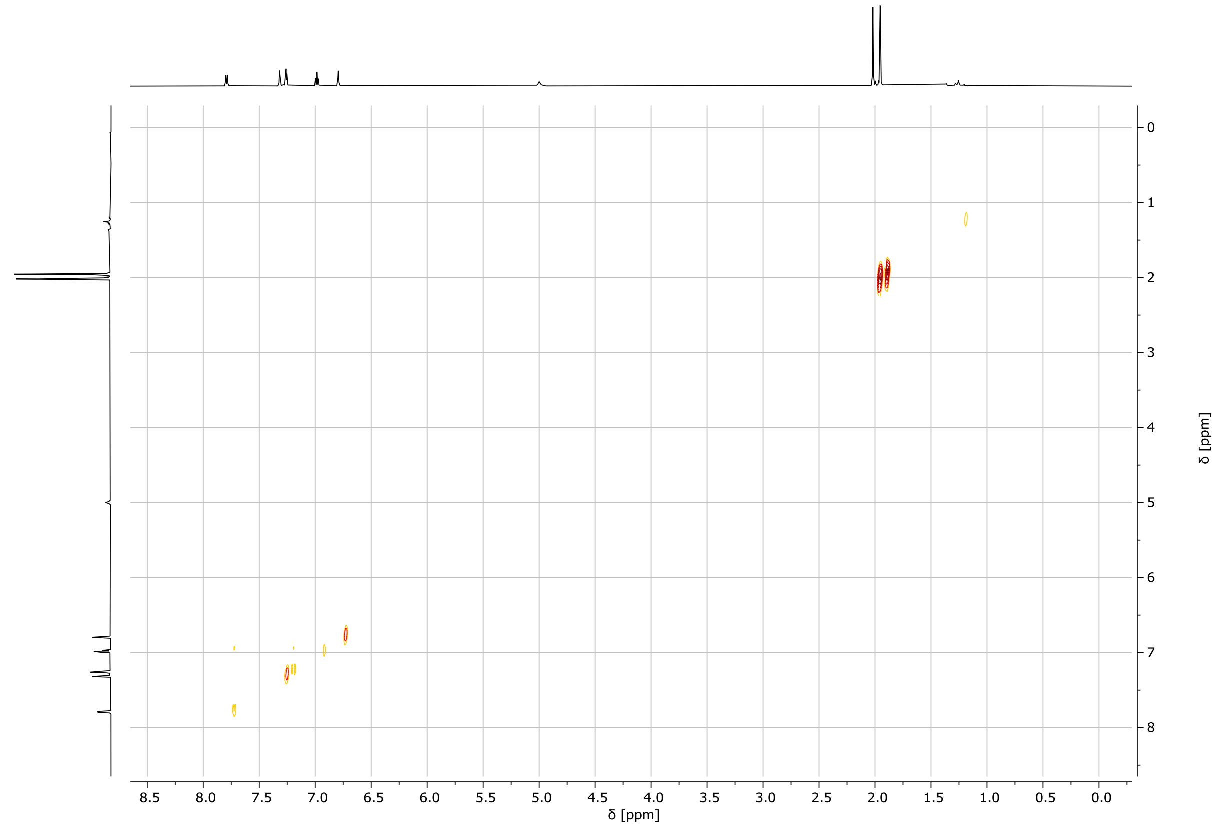


Figure S62: COSY spectrum ($CDCl_3$) of 2,2'-diiodo-6,6'-dimethyl-[1,1'-biphenyl]-4-ol (**24**).

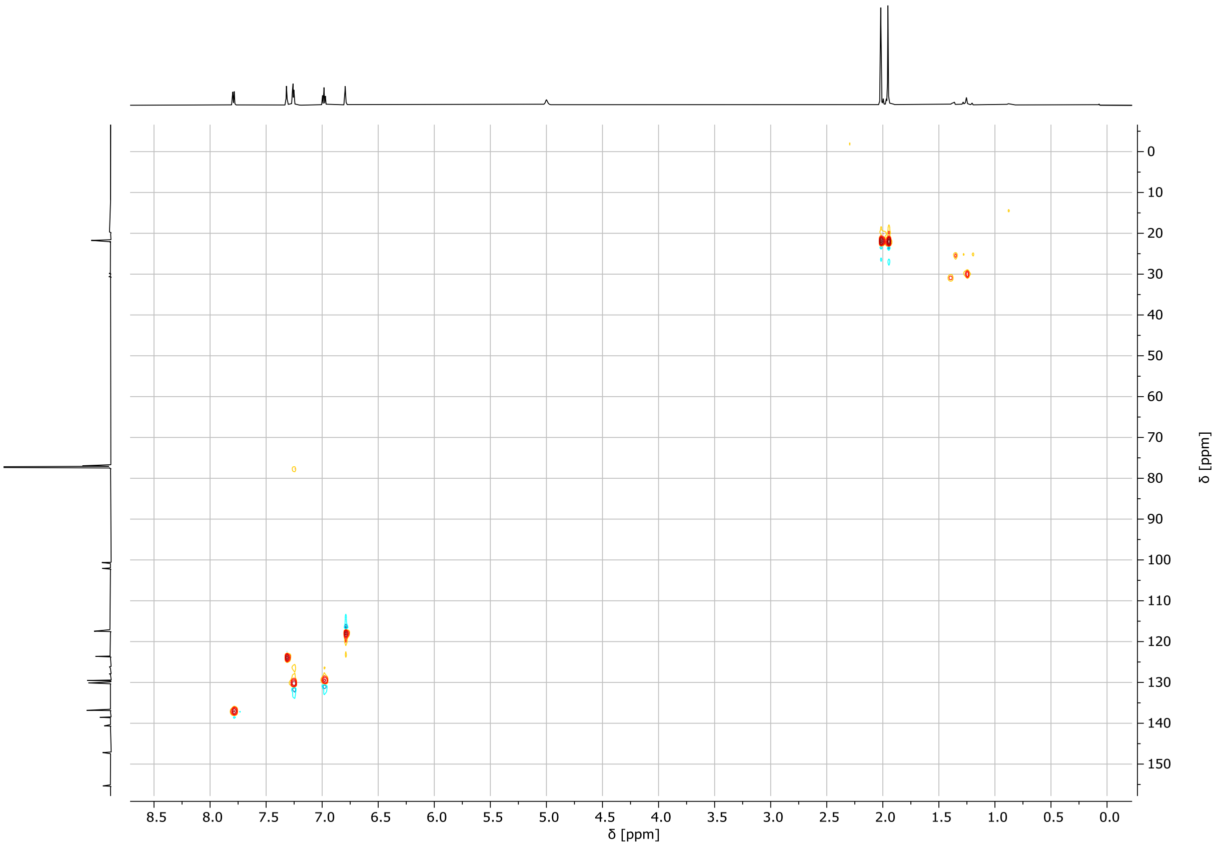


Figure S63: HSQC spectrum ($CDCl_3$) of 2,2'-diiodo-6,6'-dimethyl-[1,1'-biphenyl]-4-ol (**24**).

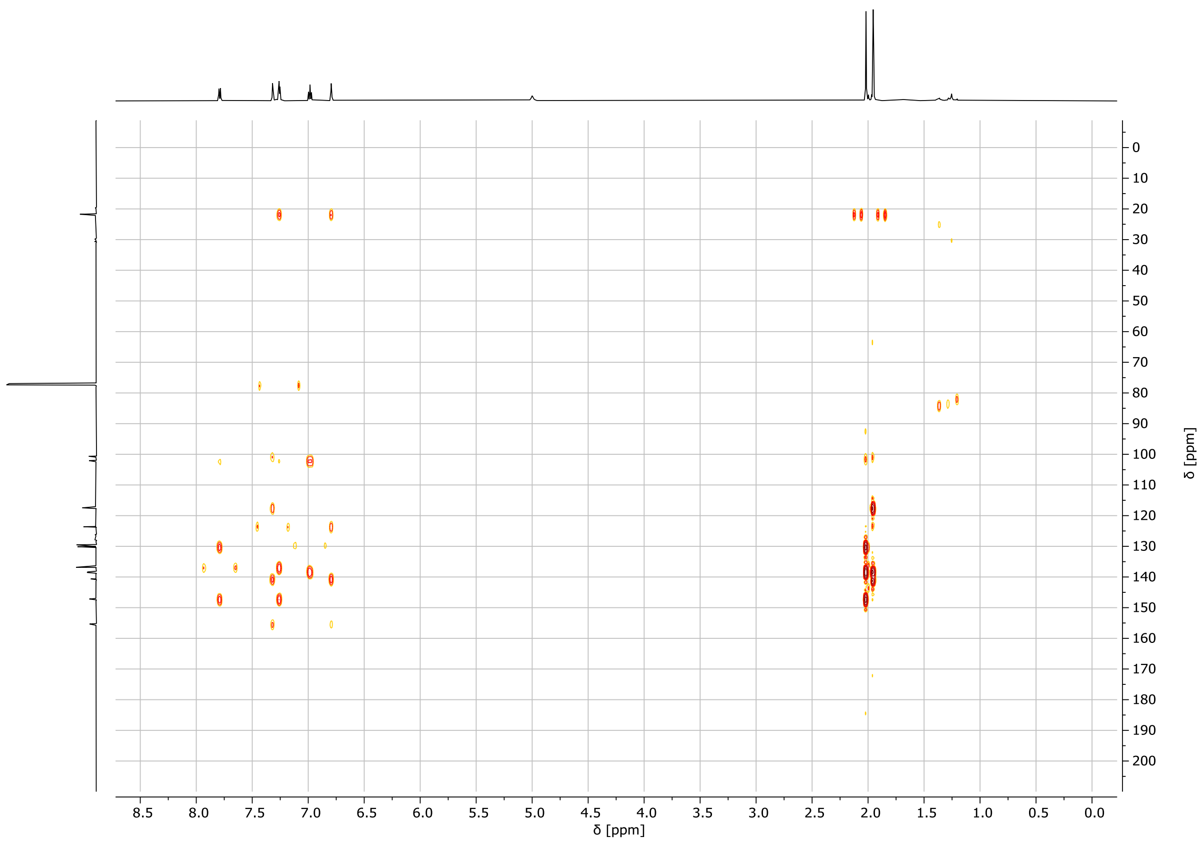


Figure S64: HMBC spectrum ($CDCl_3$) of 2,2'-diiodo-6,6'-dimethyl-[1,1'-biphenyl]-4-ol (**24**).

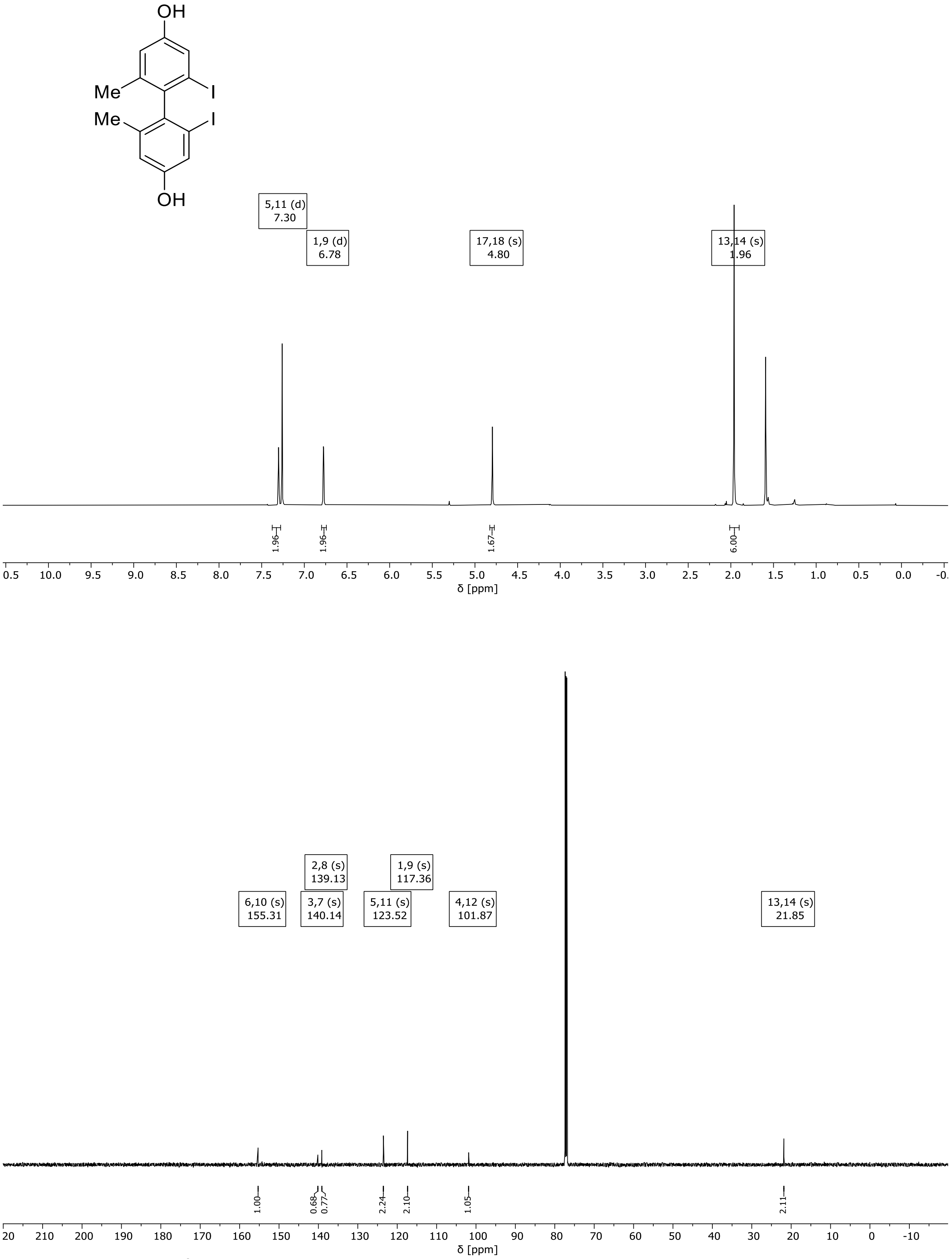


Figure S65: $^{1}$H- and $^{13}$C-NMR spectra (600 / 151 MHz, $CDCl_3$) of 2,2'-diiodo-6,6'-dimethyl-[1,1'-biphenyl]-4,4'-diol (**10**).

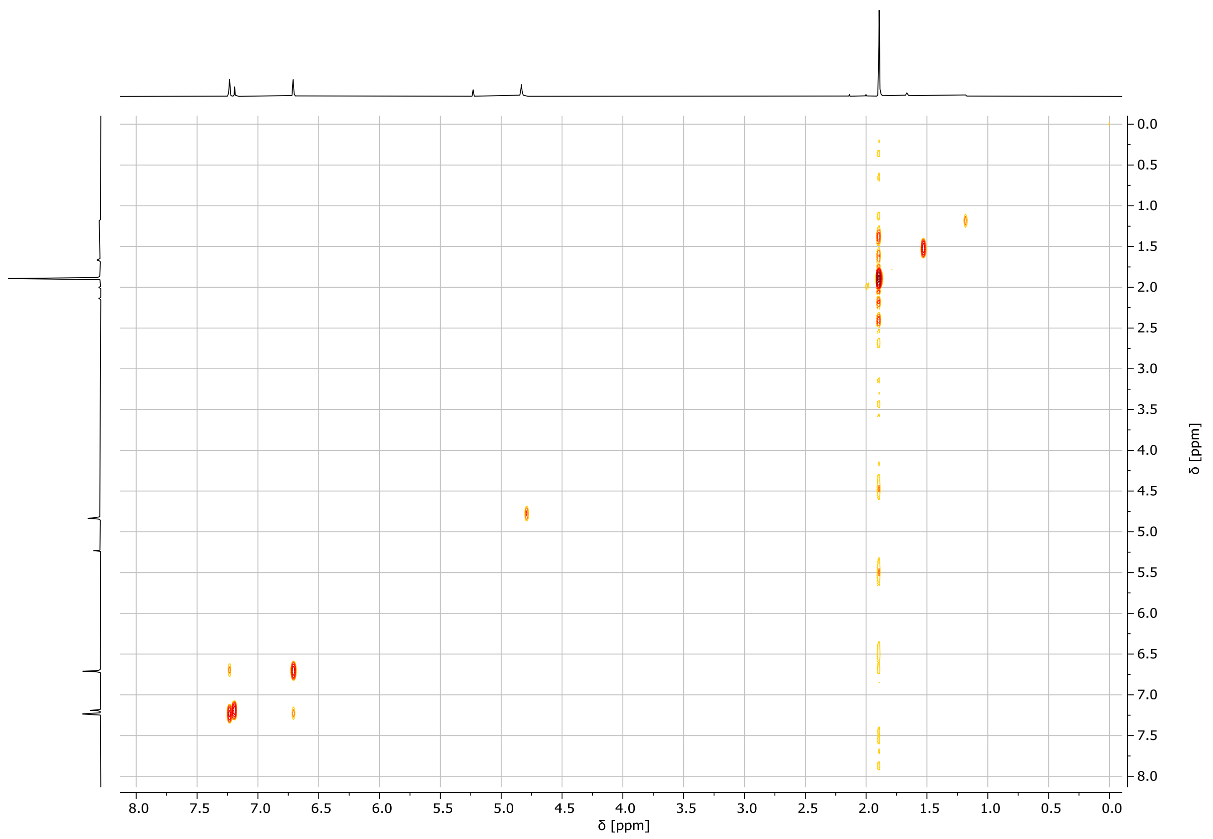


Figure S66: COSY spectrum ($CDCl_3$) of 2,2'-diiodo-6,6'-dimethyl-[1,1'-biphenyl]-4,4'-diol (**10**).

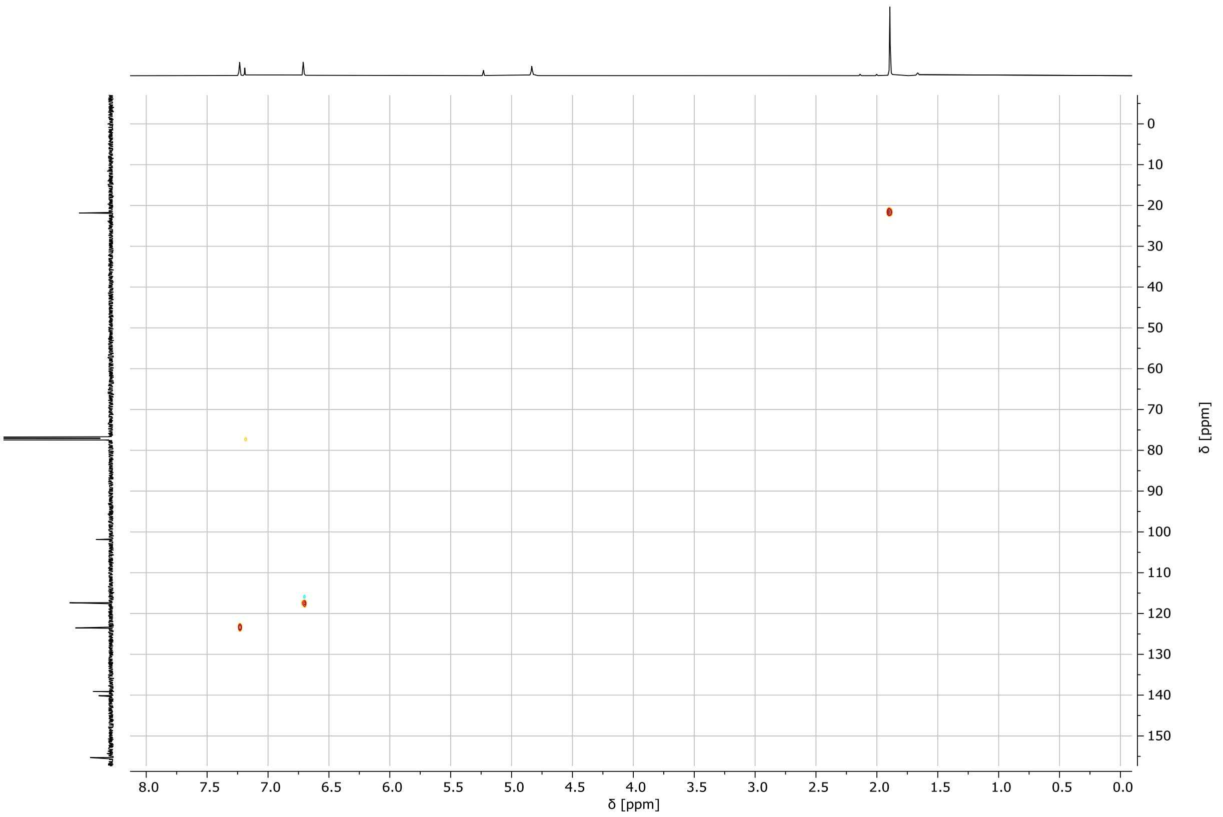


Figure S67: HSQC spectrum ($CDCl_3$) of 2,2'-diiodo-6,6'-dimethyl-[1,1'-biphenyl]-4,4'-diol (**10**).

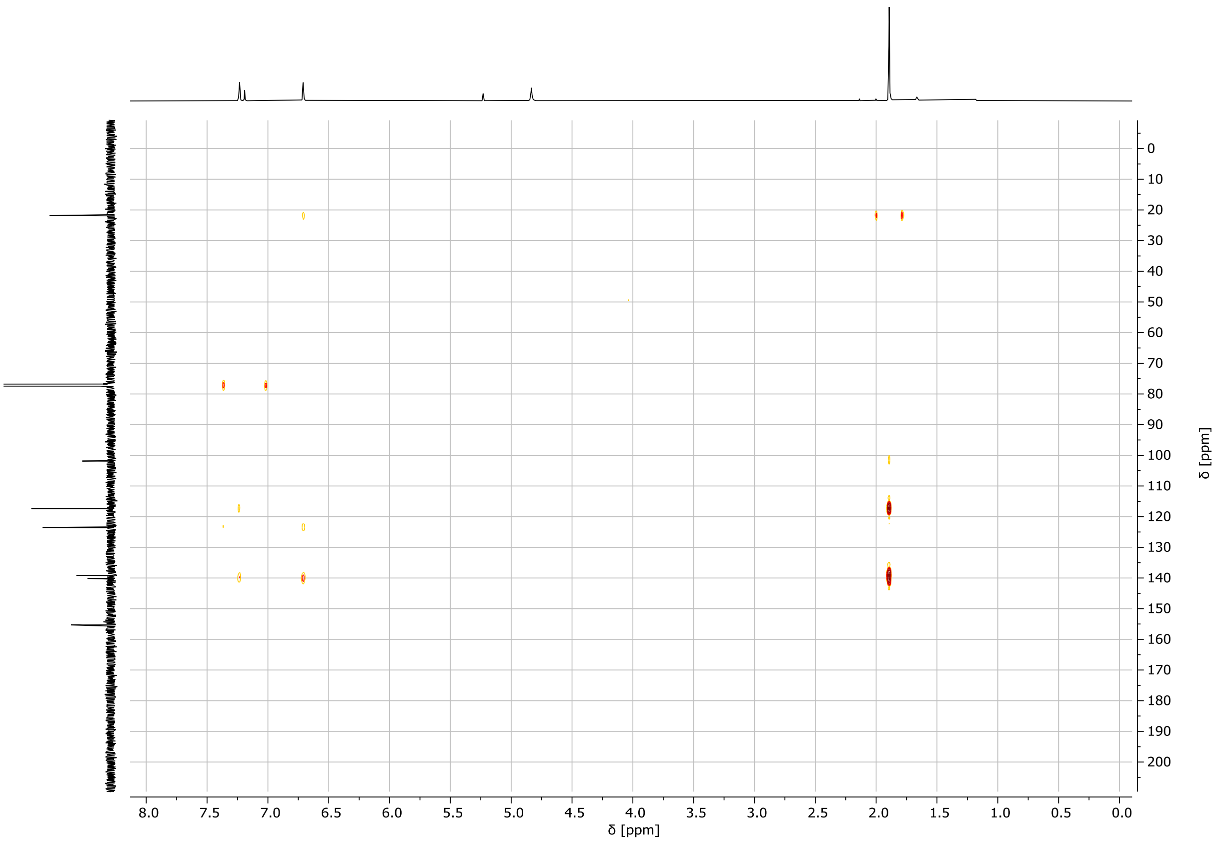


Figure S68: HMBC spectrum ($CDCl_3$) of 2,2'-diiodo-6,6'-dimethyl-[1,1'-biphenyl]-4,4'-diol (**10**).

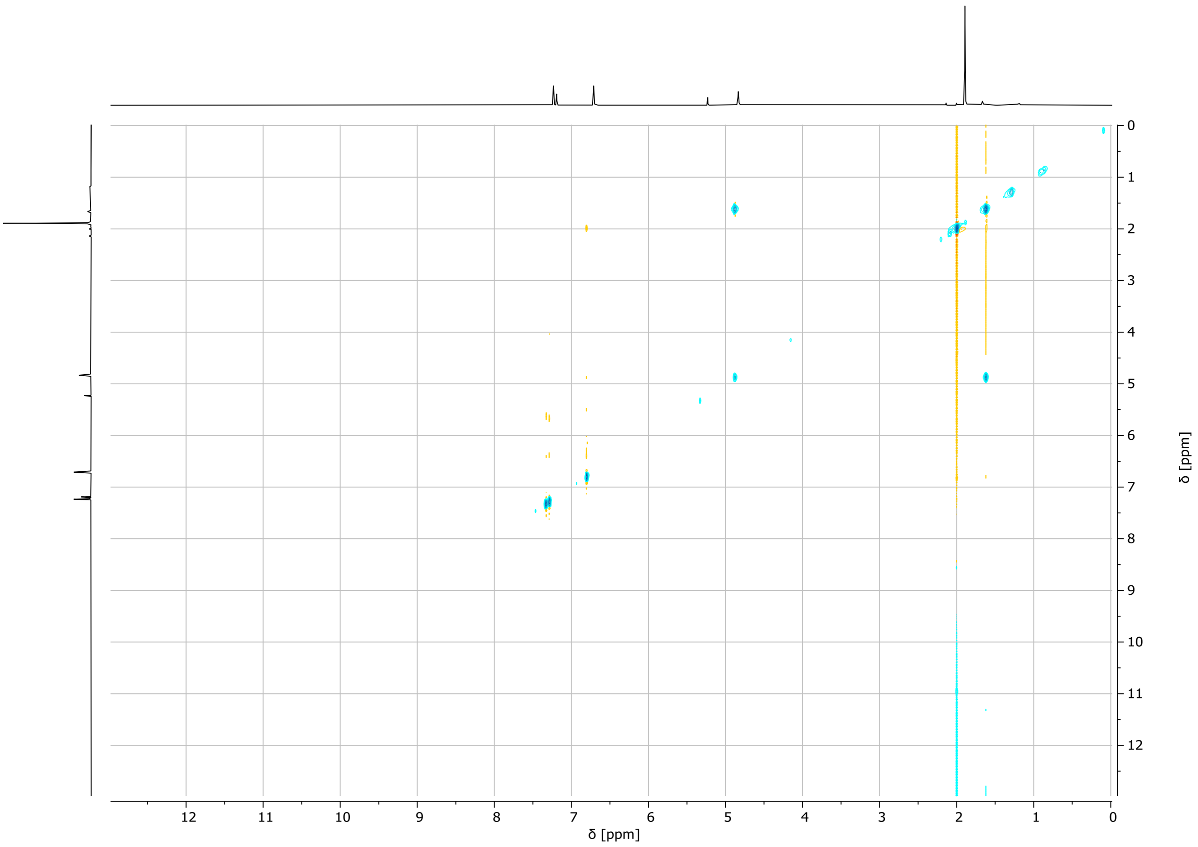


Figure S69: ROESY spectrum ($CDCl_3$) of 2,2'-diiodo-6,6'-dimethyl-[1,1'-biphenyl]-4,4'-diol (**10**).

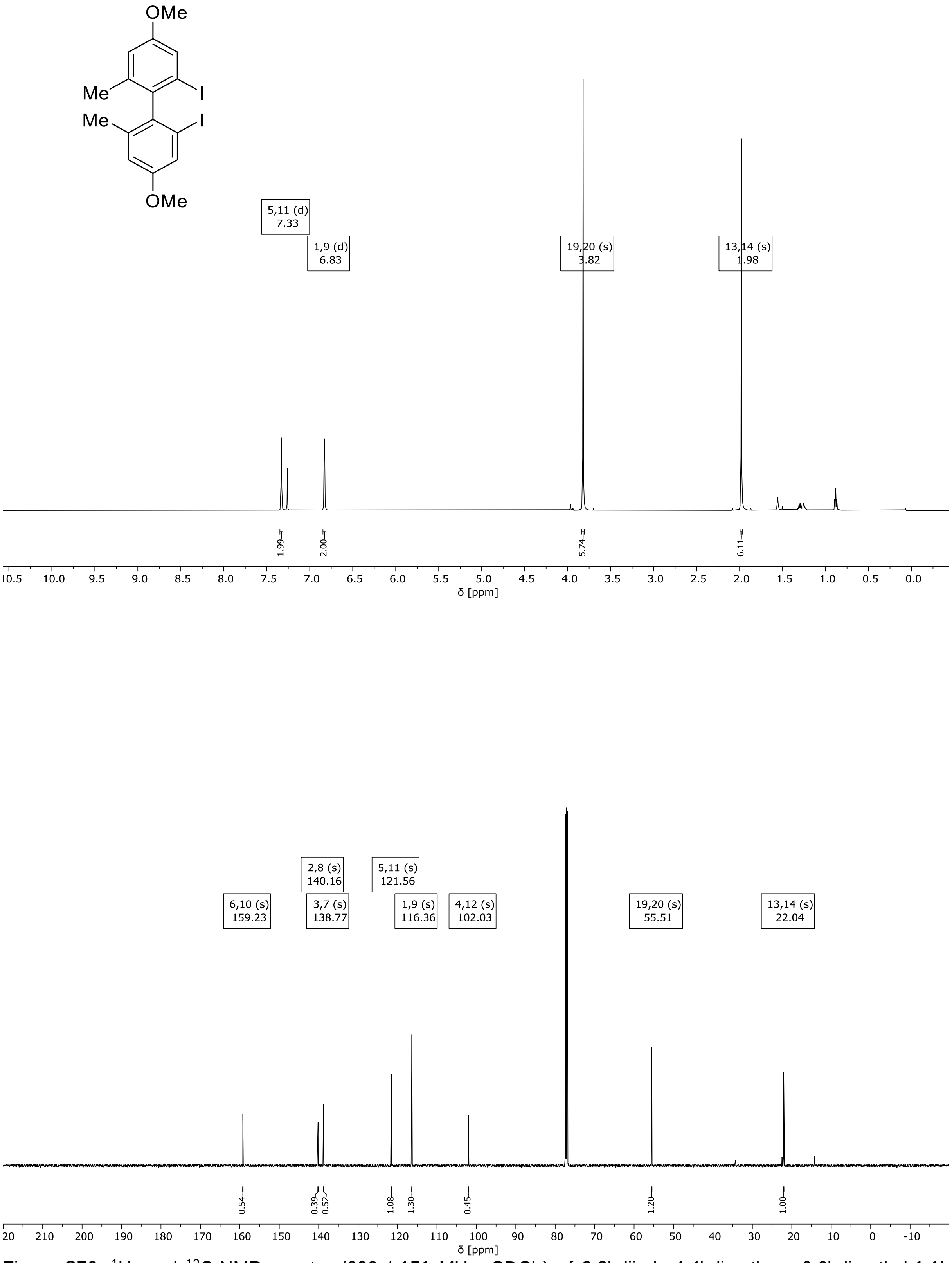

Figure S70: $^{1}$H- and $^{13}$C-NMR spectra (600 / 151 MHz, $CDCl_3$) of 2,2'-diiodo-4,4'-dimethoxy-6,6'-dimethyl-1,1'-biphenyl (**11**).

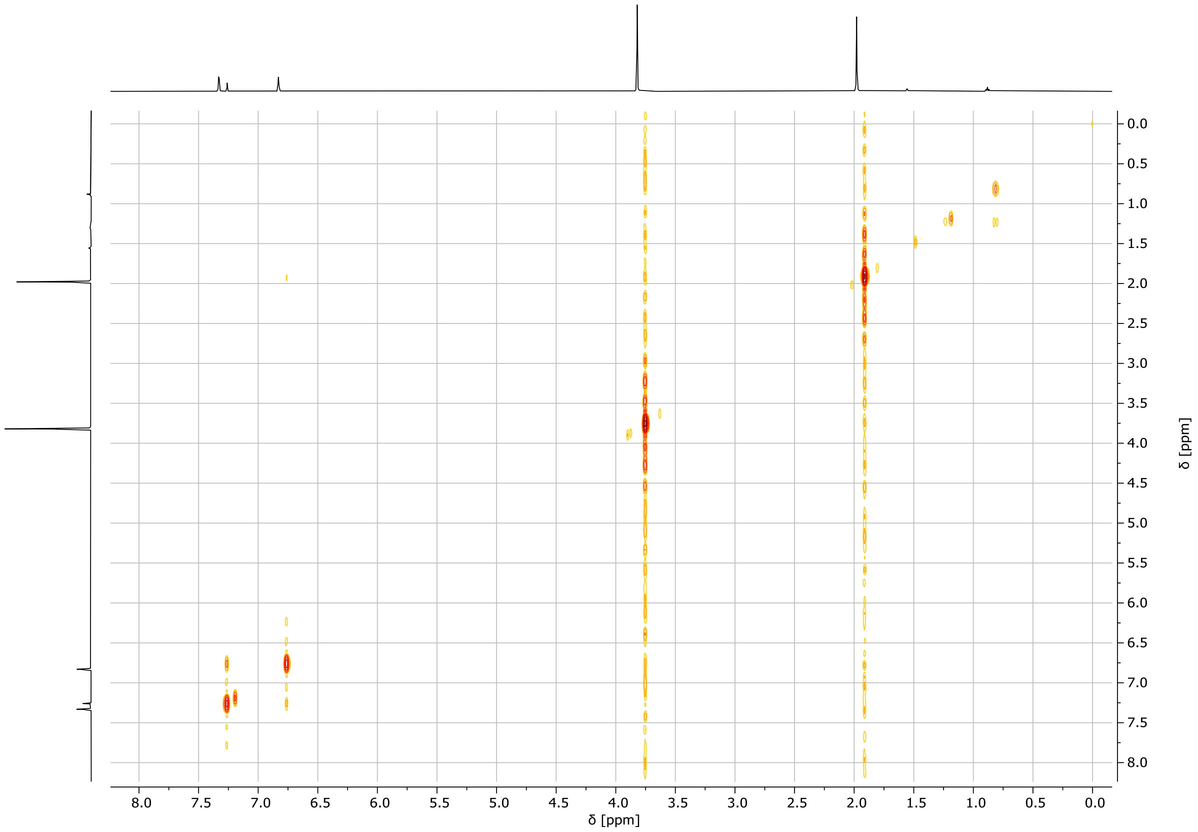


Figure S71: COSY spectrum ($CDCl_3$) of 2,2'-diiodo-4,4'-dimethoxy-6,6'-dimethyl-1,1'-biphenyl (**11**).

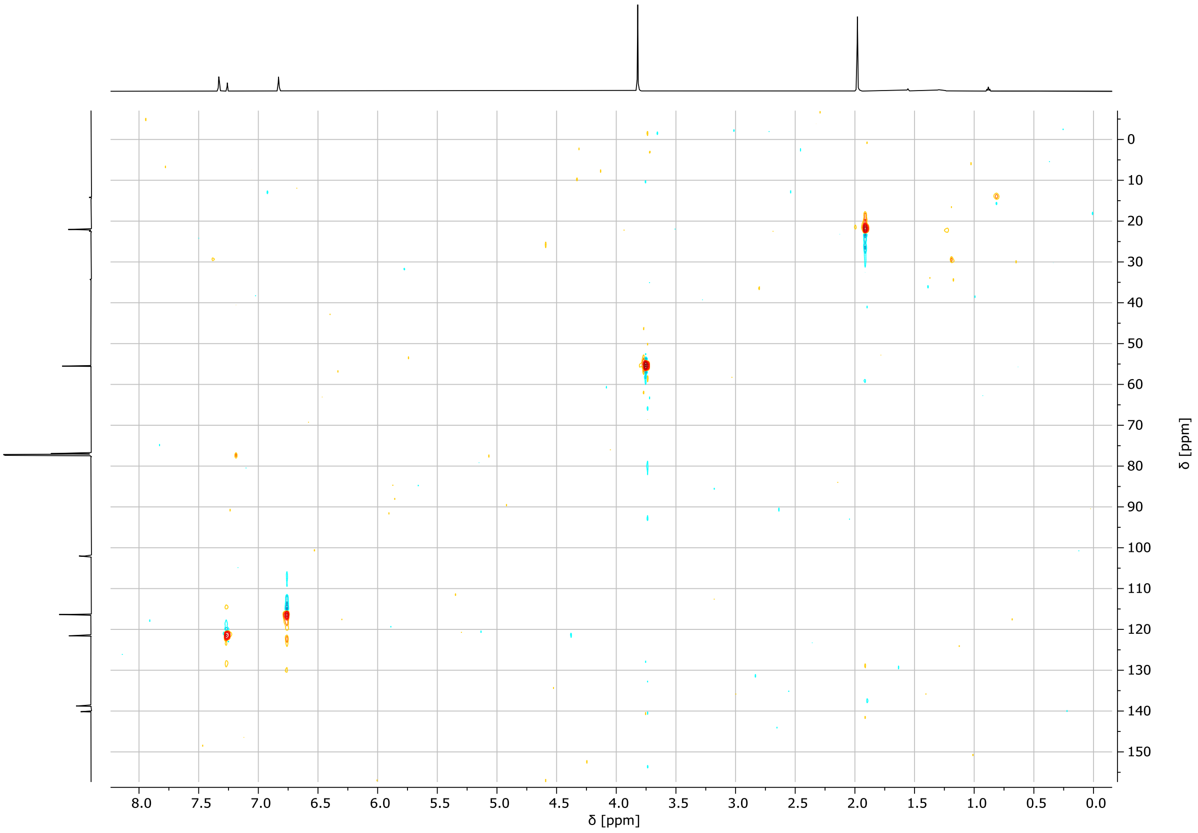


Figure S72: HSQC spectrum ($CDCl_3$) of 2,2'-diiodo-4,4'-dimethoxy-6,6'-dimethyl-1,1'-biphenyl (**11**).

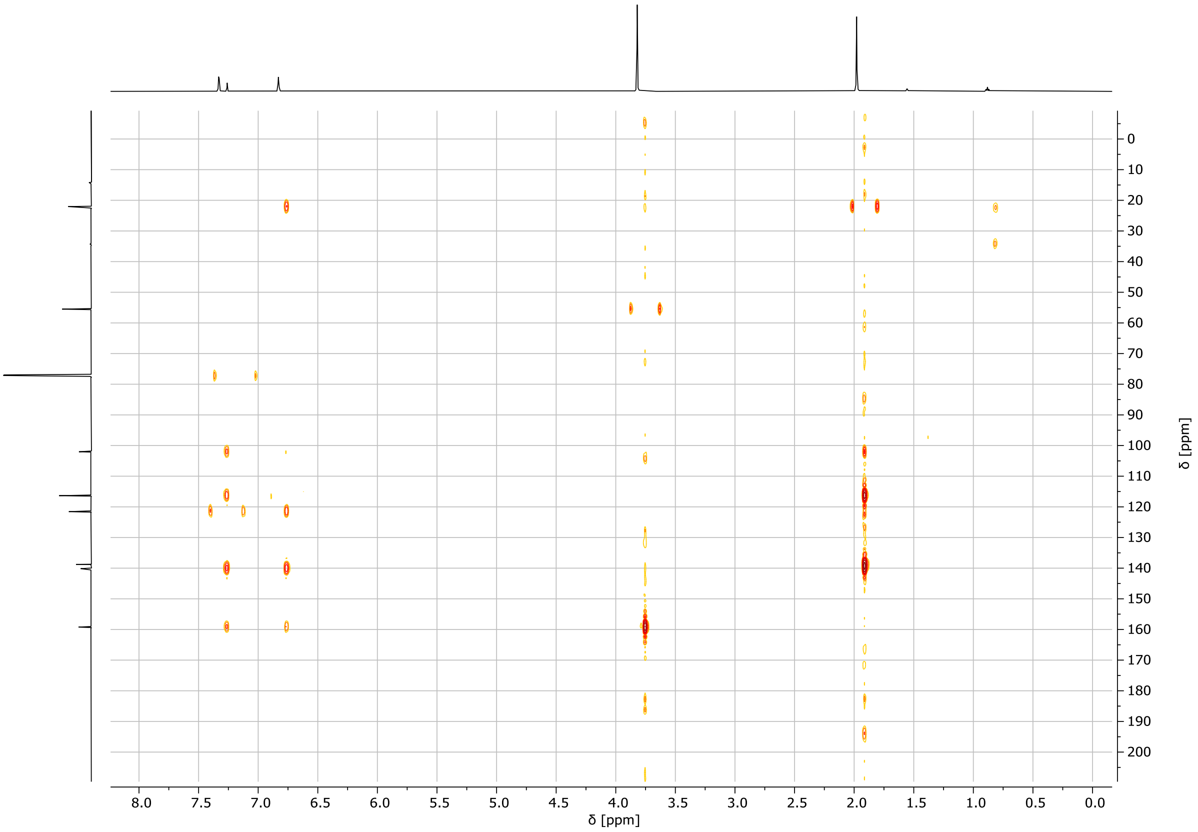


Figure S73: HMBC spectrum ($CDCl_3$) of 2,2'-diiodo-4,4'-dimethoxy-6,6'-dimethyl-1,1'-biphenyl (**11**).

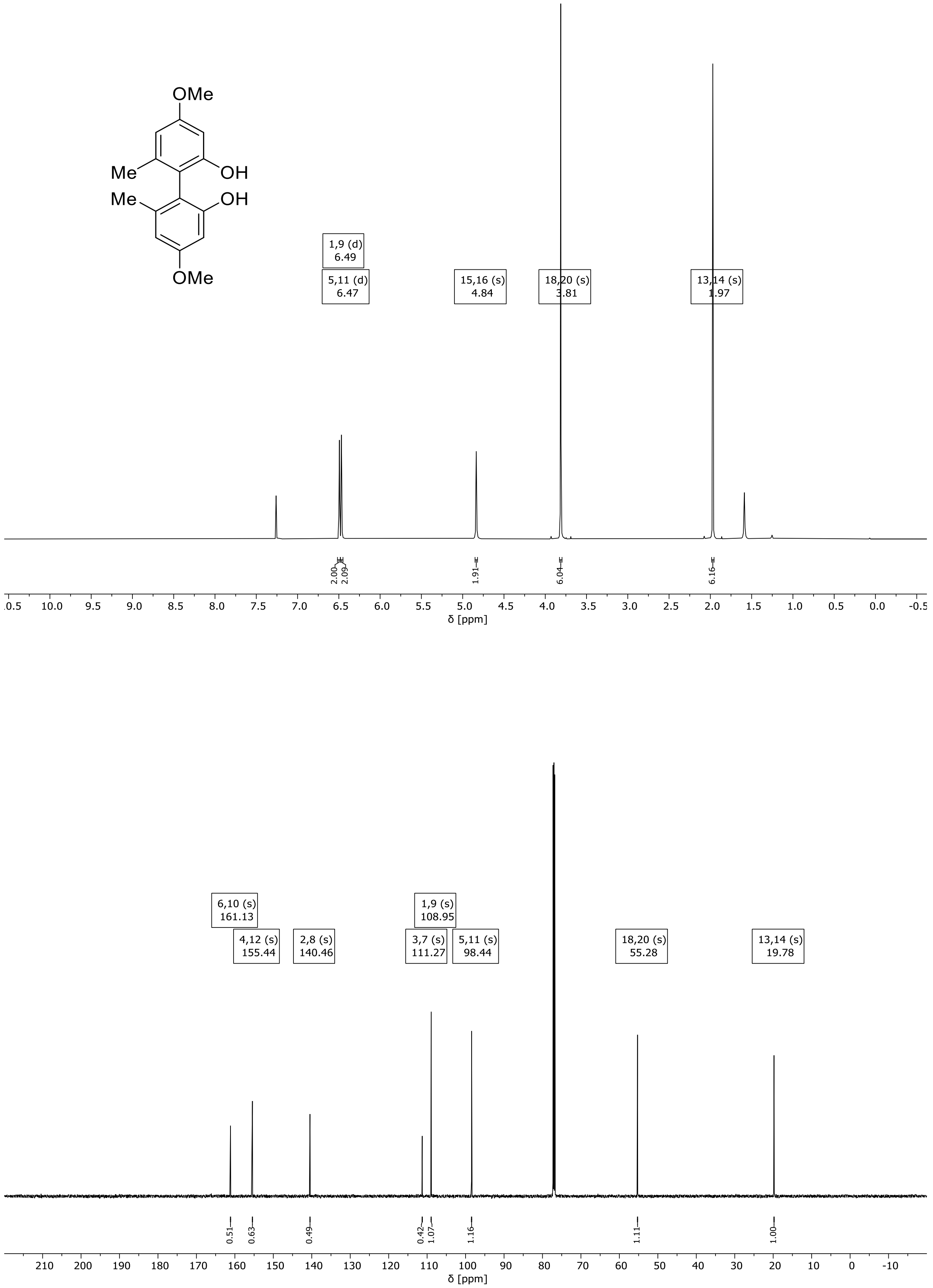


Figure S74: $^{1}H$- and $^{13}C$-NMR spectra (600 / 151 MHz, $CDCl_3$) of 4,4'-dimethoxy-6,6'-dimethyl-[1,1'-biphenyl]-2,2'-diol (**1**).

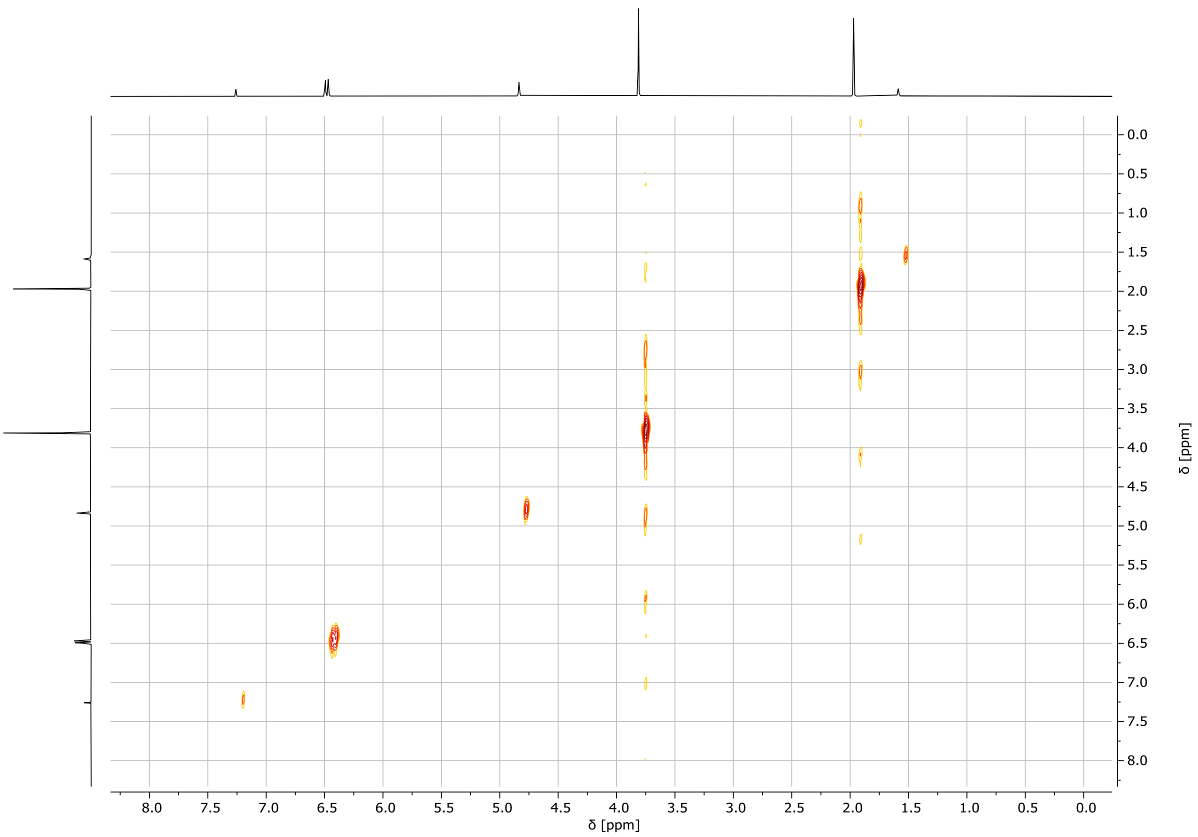


Figure S75: COSY spectrum ($CDCl_3$) of 4,4'-dimethoxy-6,6'-dimethyl-[1,1'-biphenyl]-2,2'-diol (**1**).

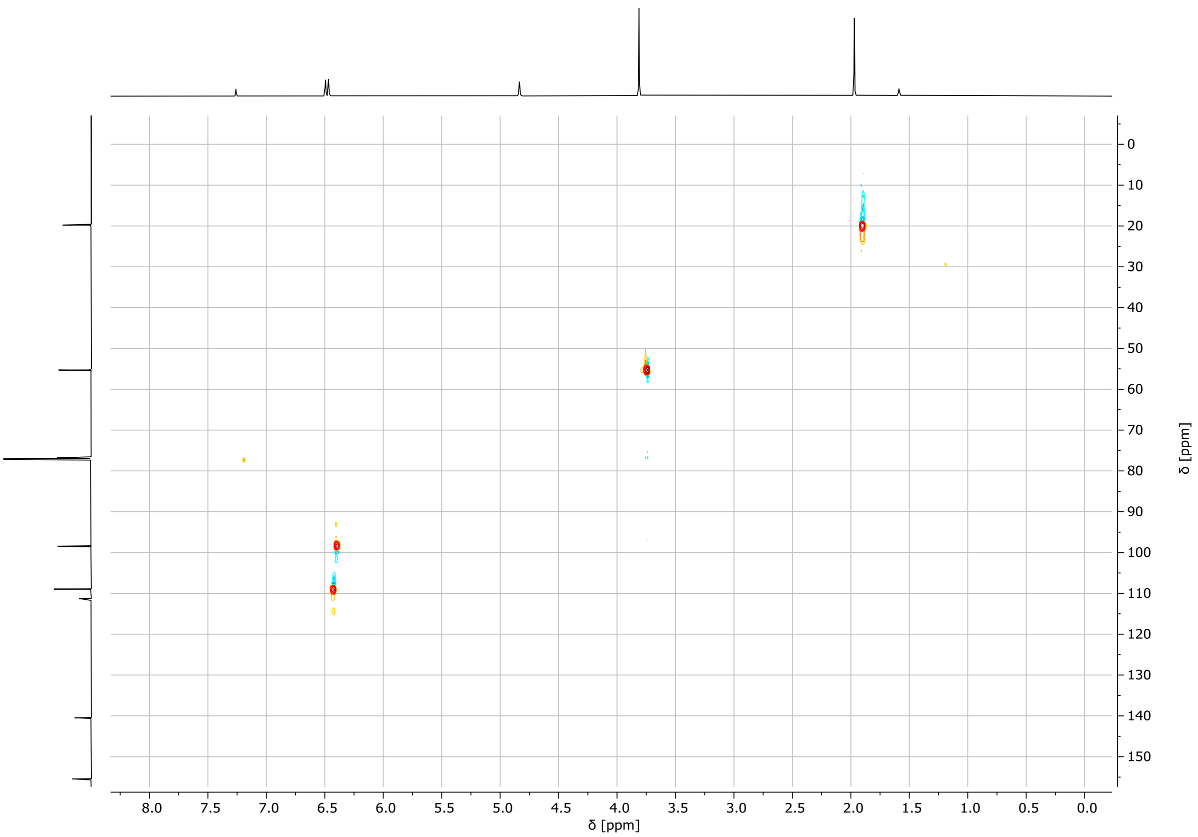


Figure S76: HSQC spectrum ($CDCl_3$) of 4,4'-dimethoxy-6,6'-dimethyl-[1,1'-biphenyl]-2,2'-diol (**1**).

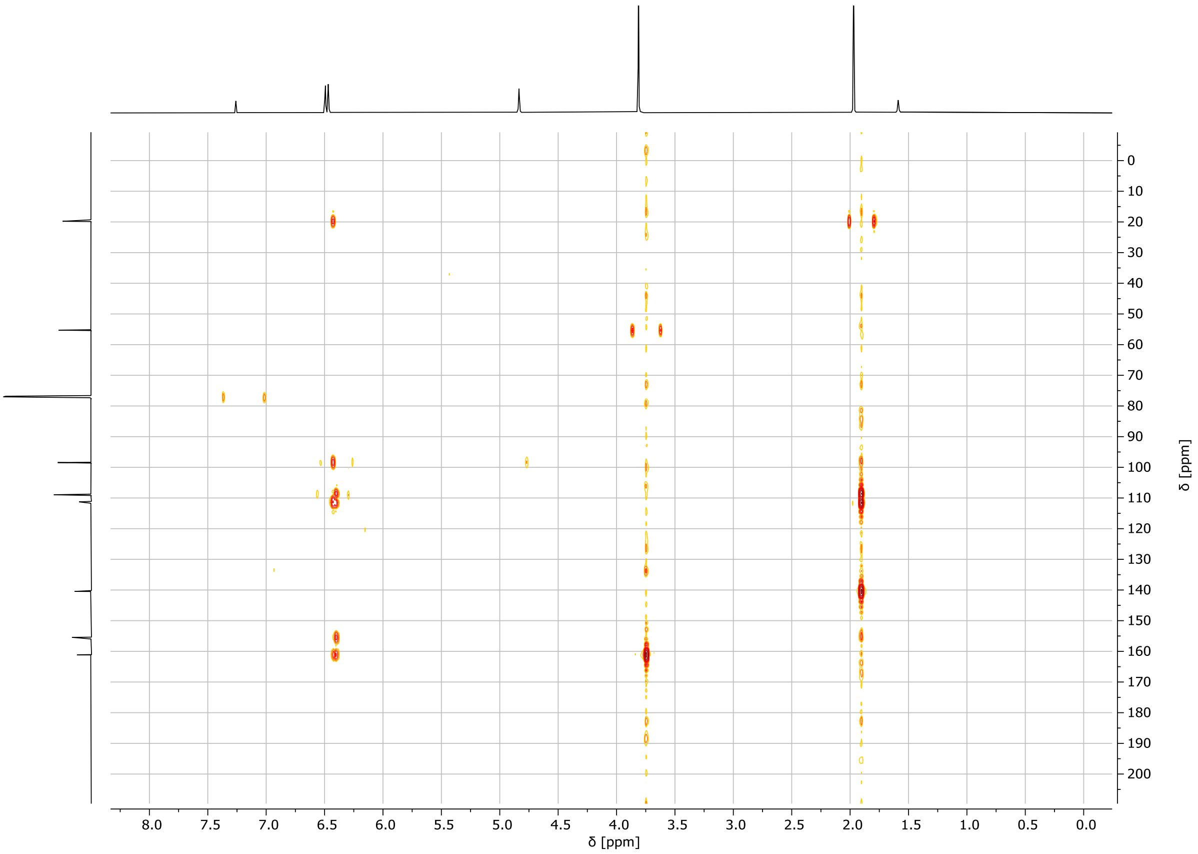


Figure S77: HMBC spectrum ($CDCl_3$) of 4,4'-dimethoxy-6,6'-dimethyl-[1,1'-biphenyl]-2,2'-diol (**1**).

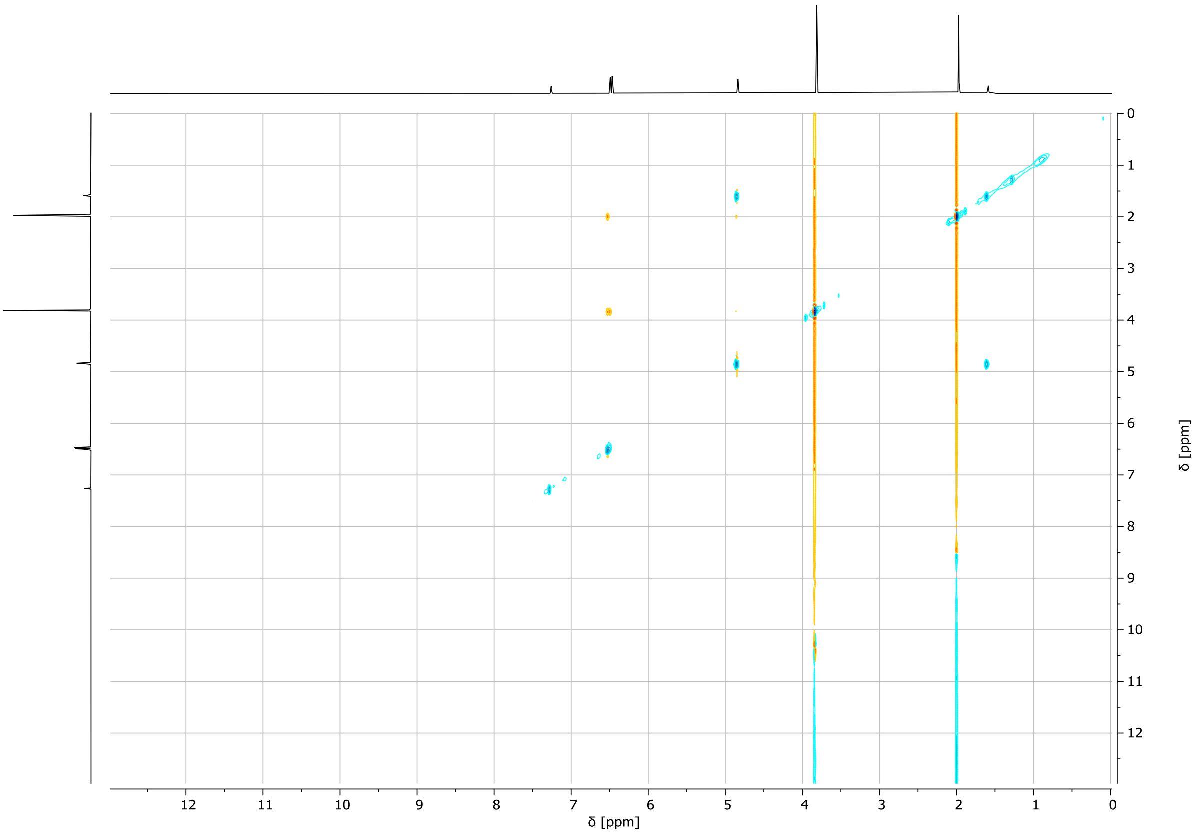


Figure S78: ROESY spectrum ($CDCl_3$) of 4,4'-dimethoxy-6,6'-dimethyl-[1,1'-biphenyl]-2,2'-diol (**1**).

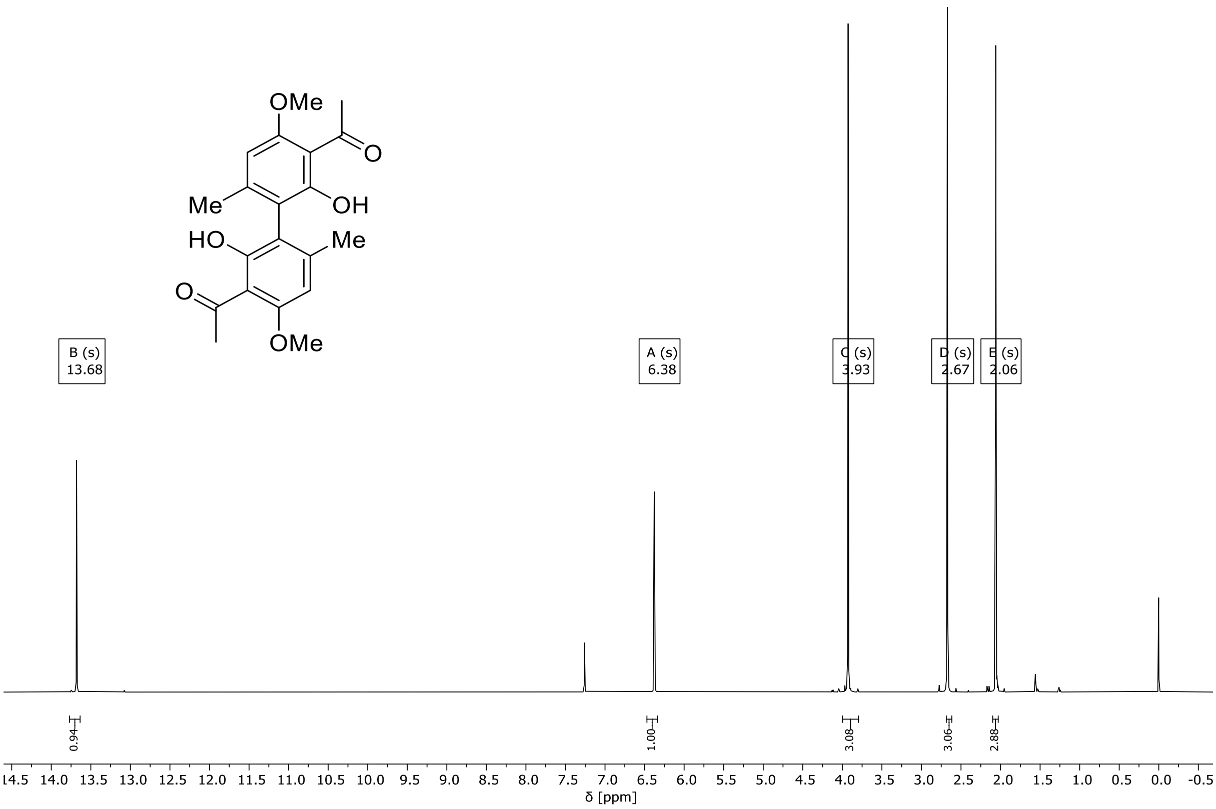


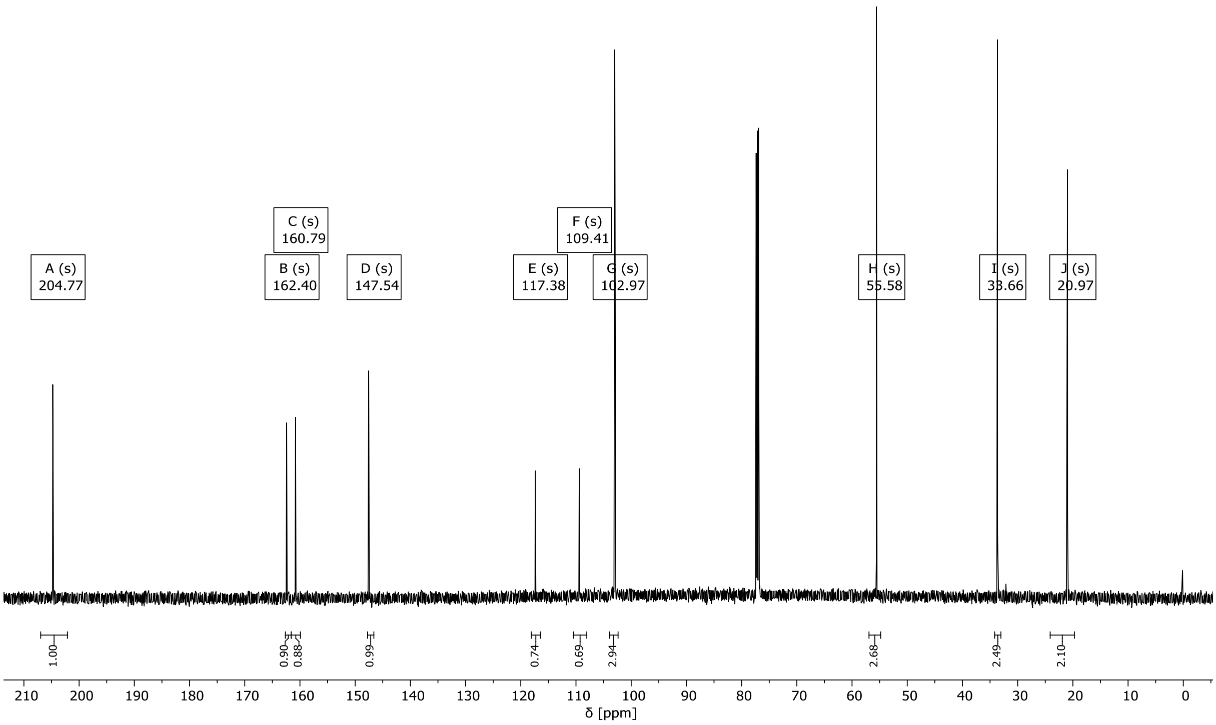


Figure S79: $^{1}H$- and $^{13}C$-NMR spectrum (600 / 151 MHz, $CDCl_3$) of 1-(2-hydroxy-6-methoxy-4-methylphenyl)ethan-1-one (**12**)

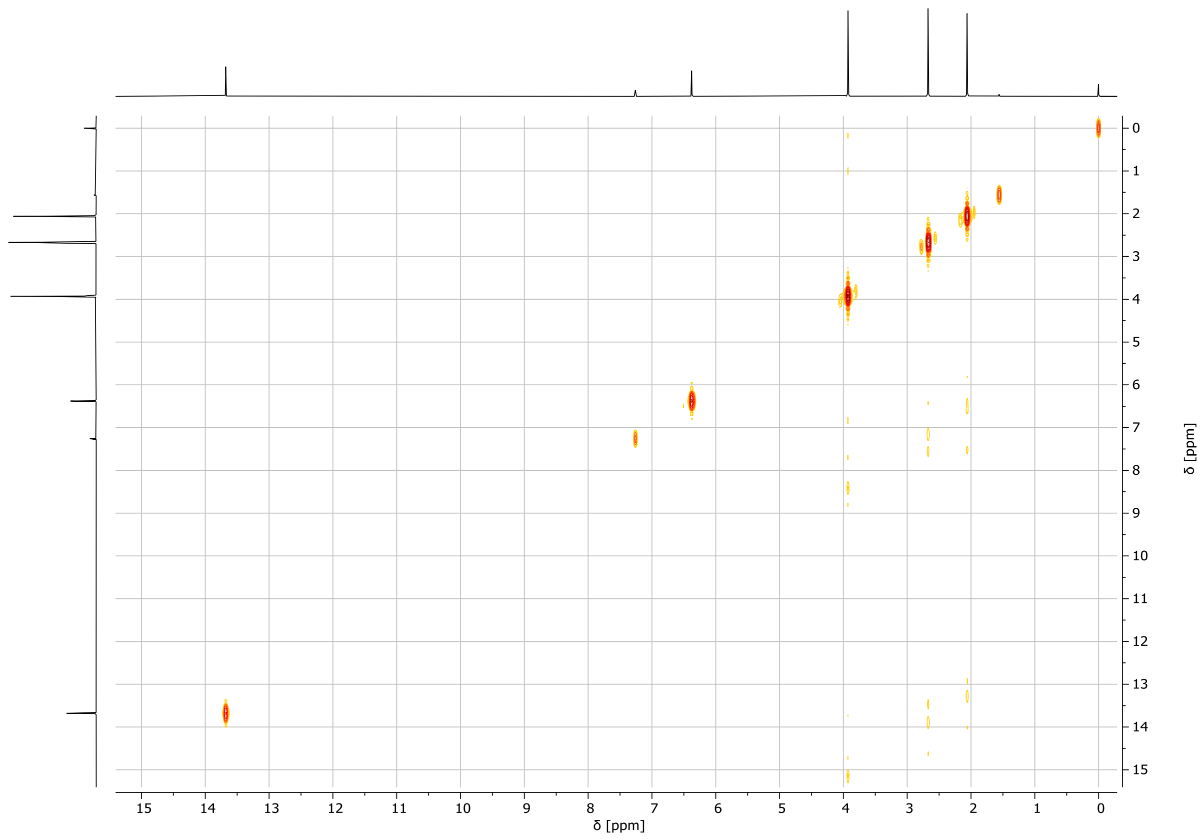


Figure S80: COSY spectrum ($CDCl_3$) of 1-(2-hydroxy-6-methoxy-4-methylphenyl)ethan-1-one (**12**).

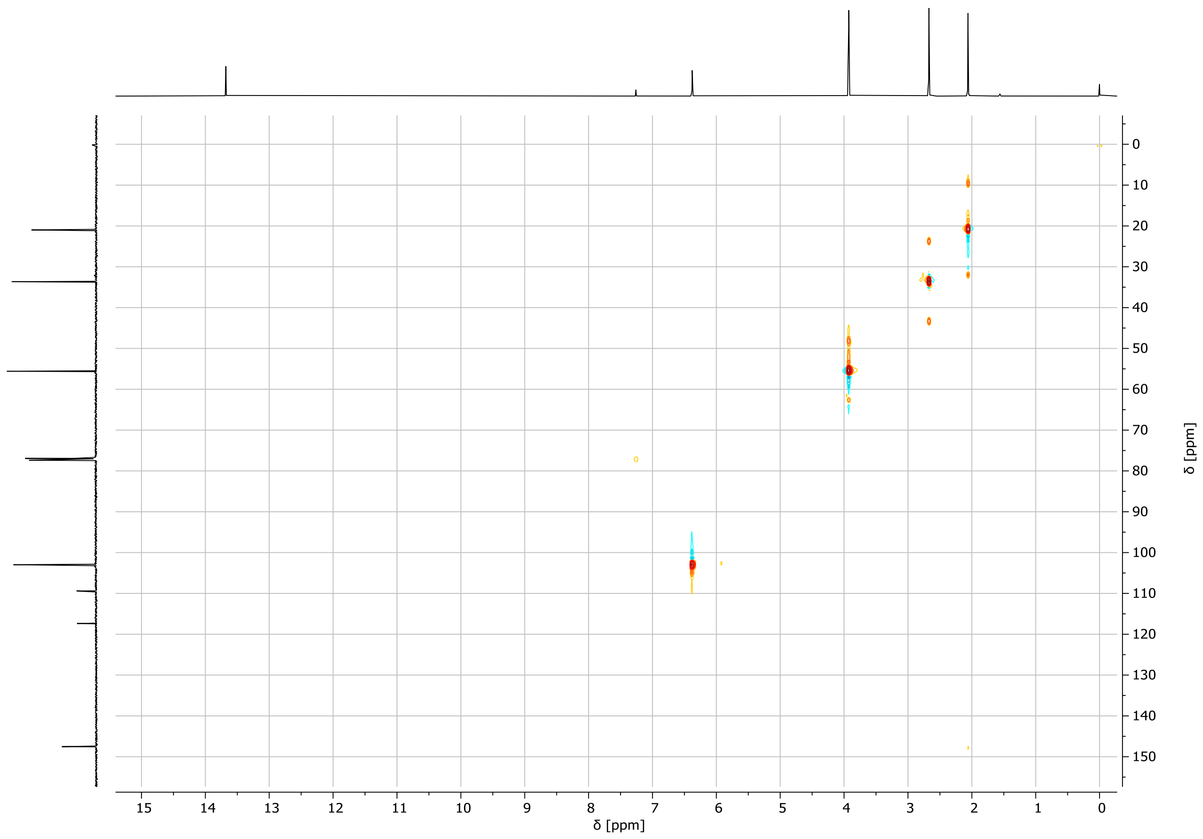


Figure S81: HSQC spectrum ($CDCl_3$) of 1-(2-hydroxy-6-methoxy-4-methylphenyl)ethan-1-one (**12**).

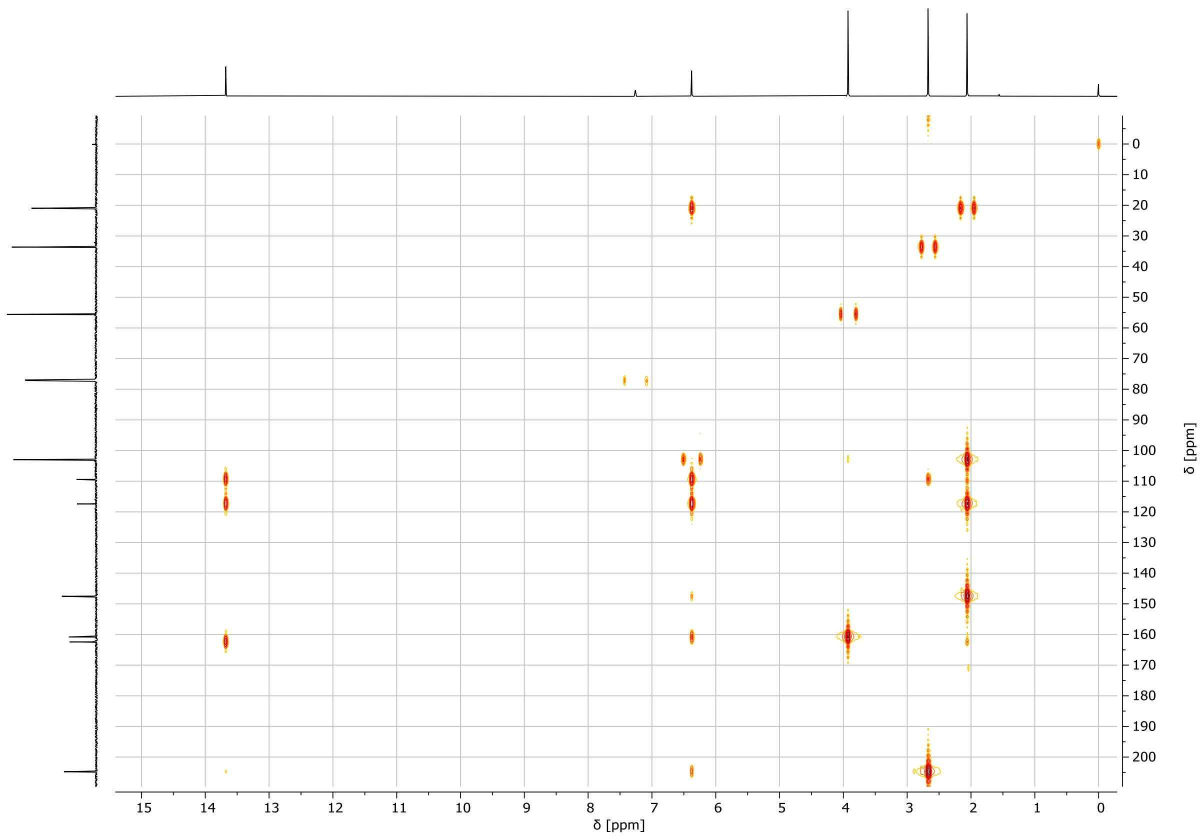


Figure S82: HMBC spectrum (CDCl₃) of 1-(2-hydroxy-6-methoxy-4-methylphenyl)ethan-1-one (**12**).

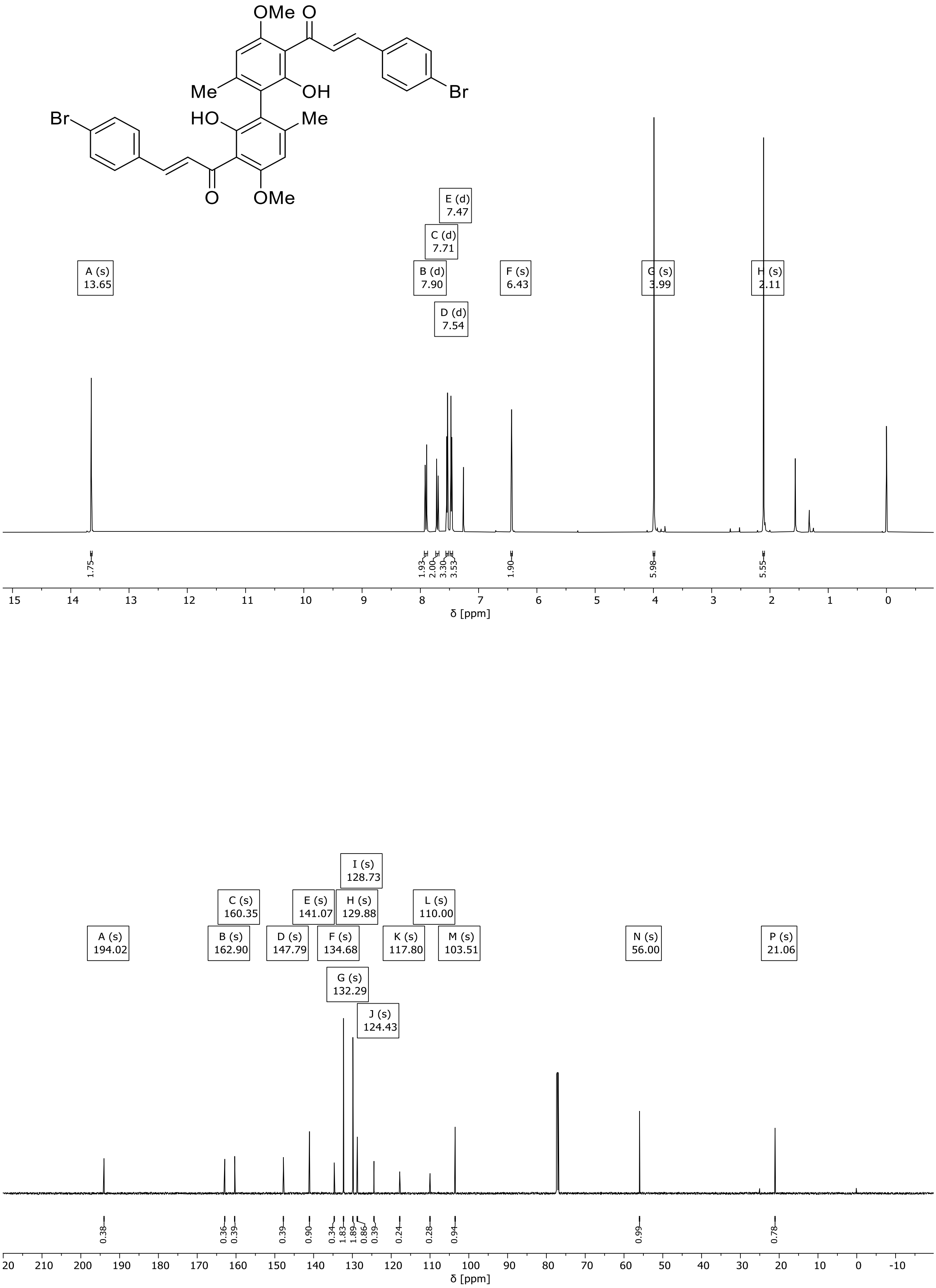


Figure S83: $^{1}H$- and $^{13}C$-NMR spectrum (600 / 151 MHz, $CDCl_3$) of (2E,2'E)-1,1'-(2,2'-dihydroxy-4,4'-dimethoxy-6,6'-dimethyl-[1,1'-biphenyl]-3,3'-diyl)bis(3-(4-bromophenyl)prop-2-en-1-one) (**13**).

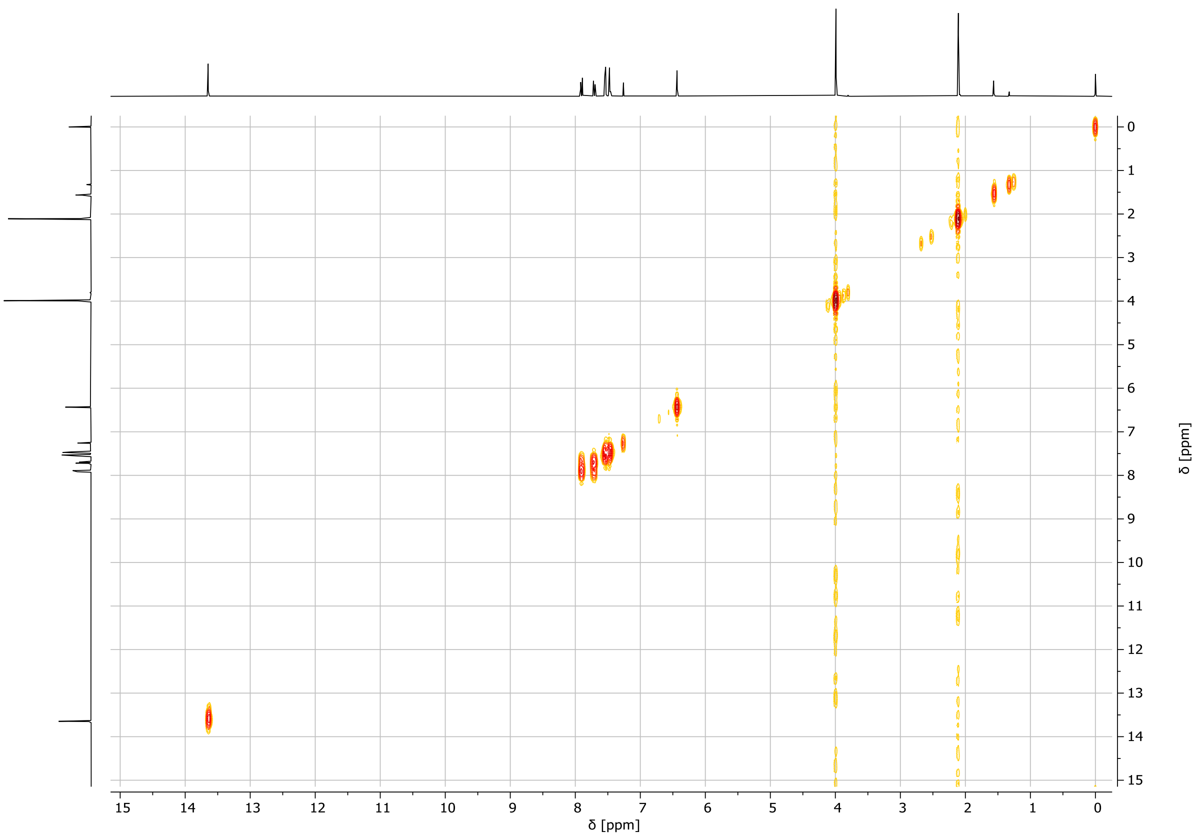


Figure S84: COSY spectrum ($CDCl_3$) of (2E,2'E)-1,1'-(2,2'-dihydroxy-4,4'-dimethoxy-6,6'-dimethyl-[1,1'-biphenyl]-3,3'-diyl)bis(3-(4-bromophenyl)prop-2-en-1-one) (**13**).

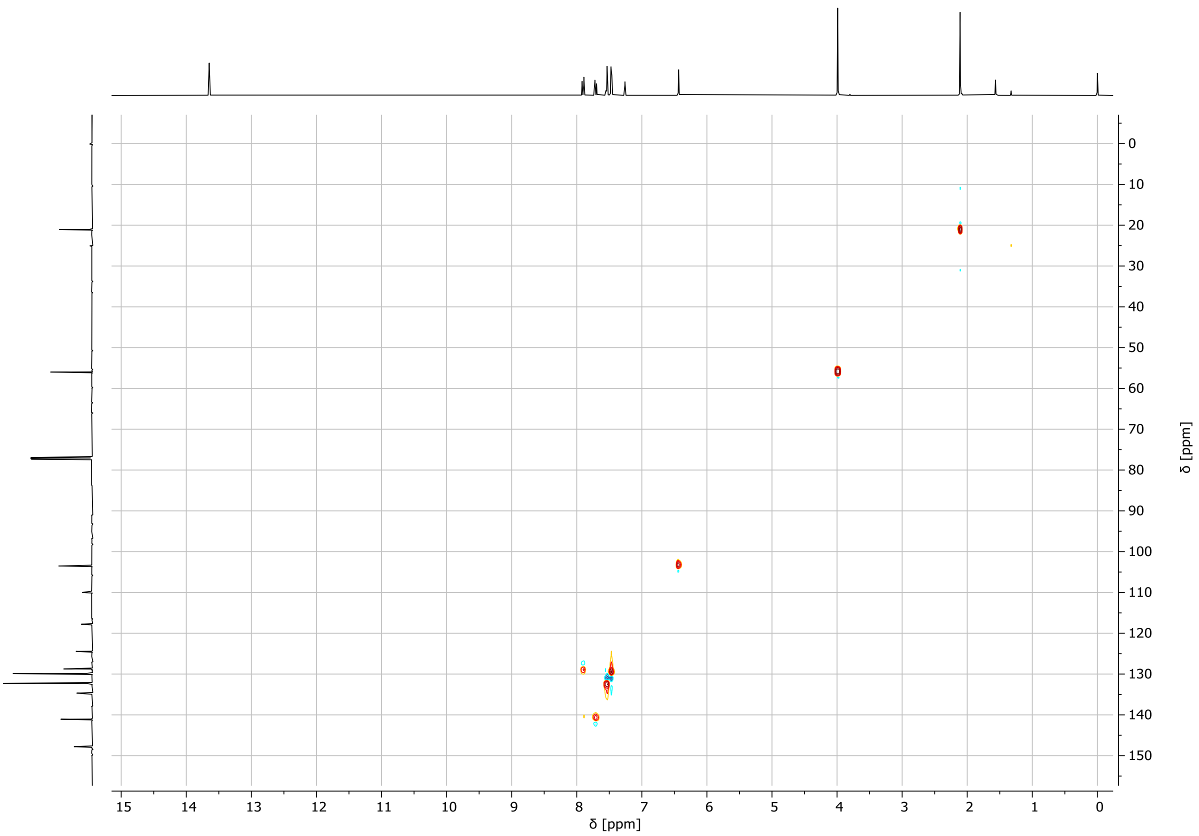


Figure S85: HSQC spectrum ($CDCl_3$) of (2E,2'E)-1,1'-(2,2'-dihydroxy-4,4'-dimethoxy-6,6'-dimethyl-[1,1'-biphenyl]-3,3'-diyl)bis(3-(4-bromophenyl)prop-2-en-1-one) (**13**).

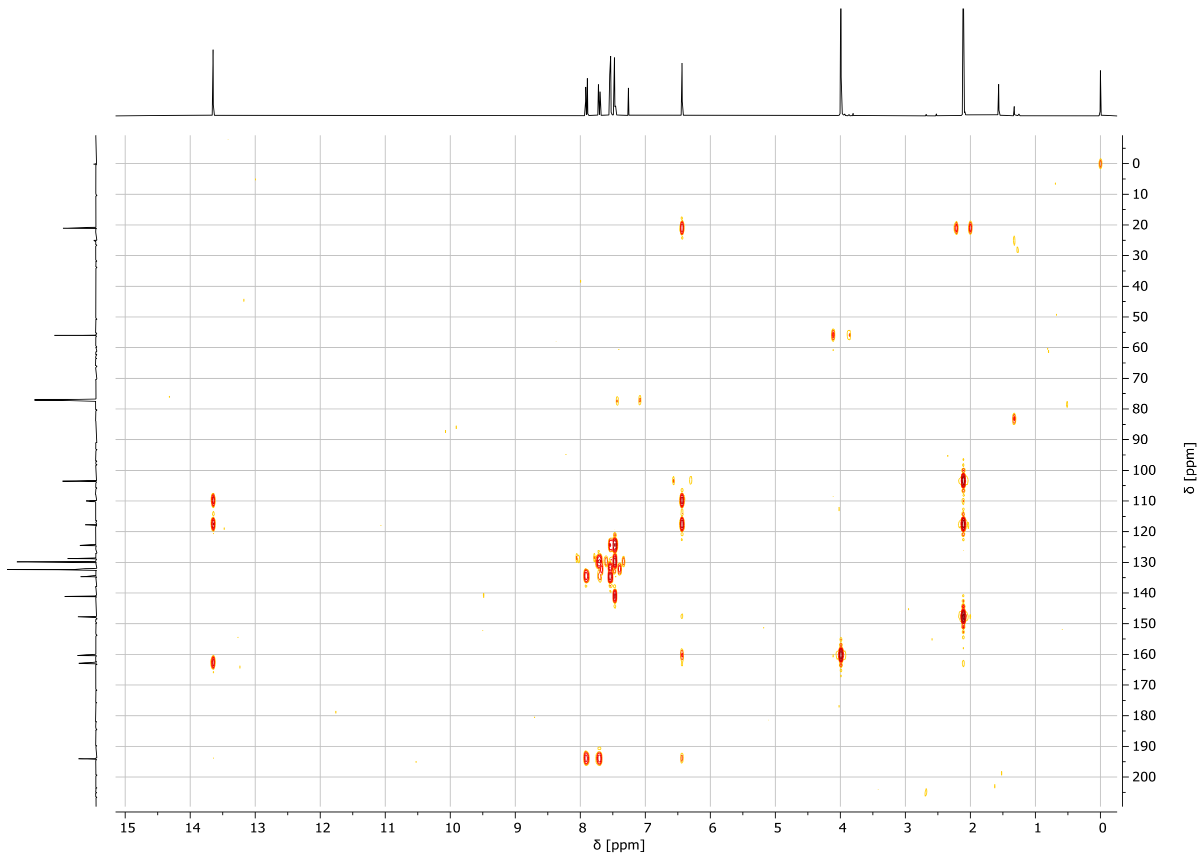


Figure S86: HMBC spectrum ($CDCl_3$) of (2E,2'E)-1,1'-(2,2'-dihydroxy-4,4'-dimethoxy-6,6'-dimethyl-[1,1'-biphenyl]-3,3'-diyl)bis(3-(4-bromophenyl)prop-2-en-1-one) (**13**).

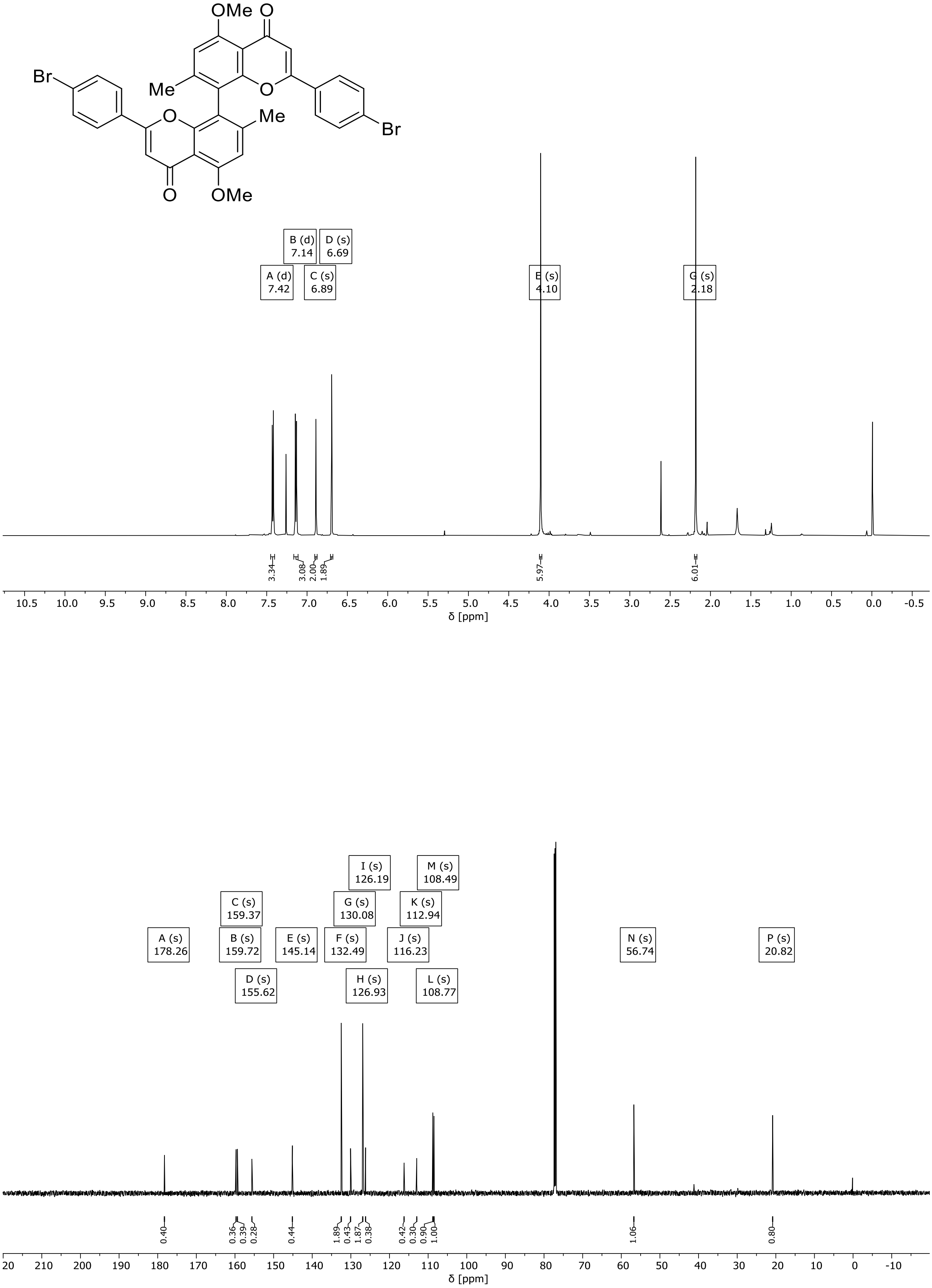


Figure S87: $^{1}H$- and $^{13}C$-NMR spectrum (600 / 151 MHz, $CDCl_3$) of 2,2'-bis(4-bromophenyl)-5,5'-dimethoxy-7,7'-dimethyl-4H,4'H-[8,8'-bichromene]-4,4'-dione (**2**).

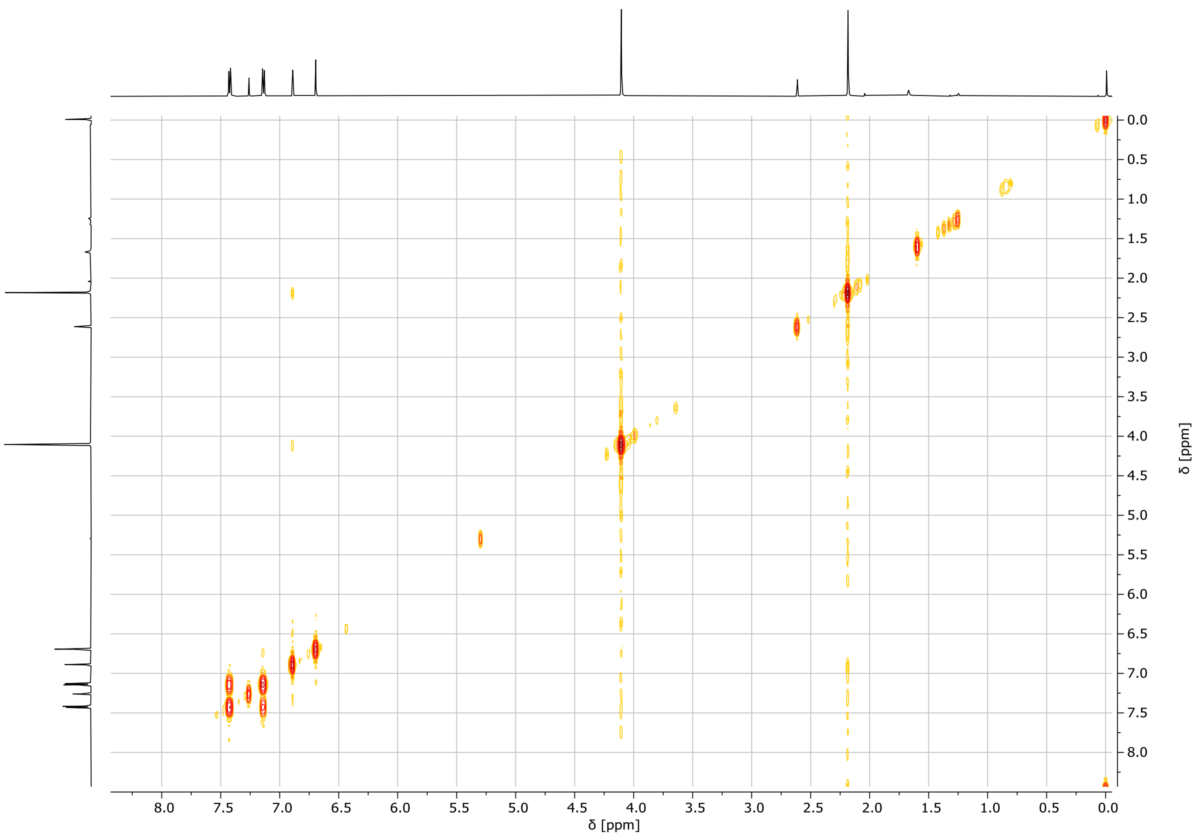


Figure S88: COSY spectrum ($CDCl_3$) of 2,2'-bis(4-bromophenyl)-5,5'-dimethoxy-7,7'-dimethyl-4H,4'H-[8,8'-bichromene]-4,4'-dione (**2**).

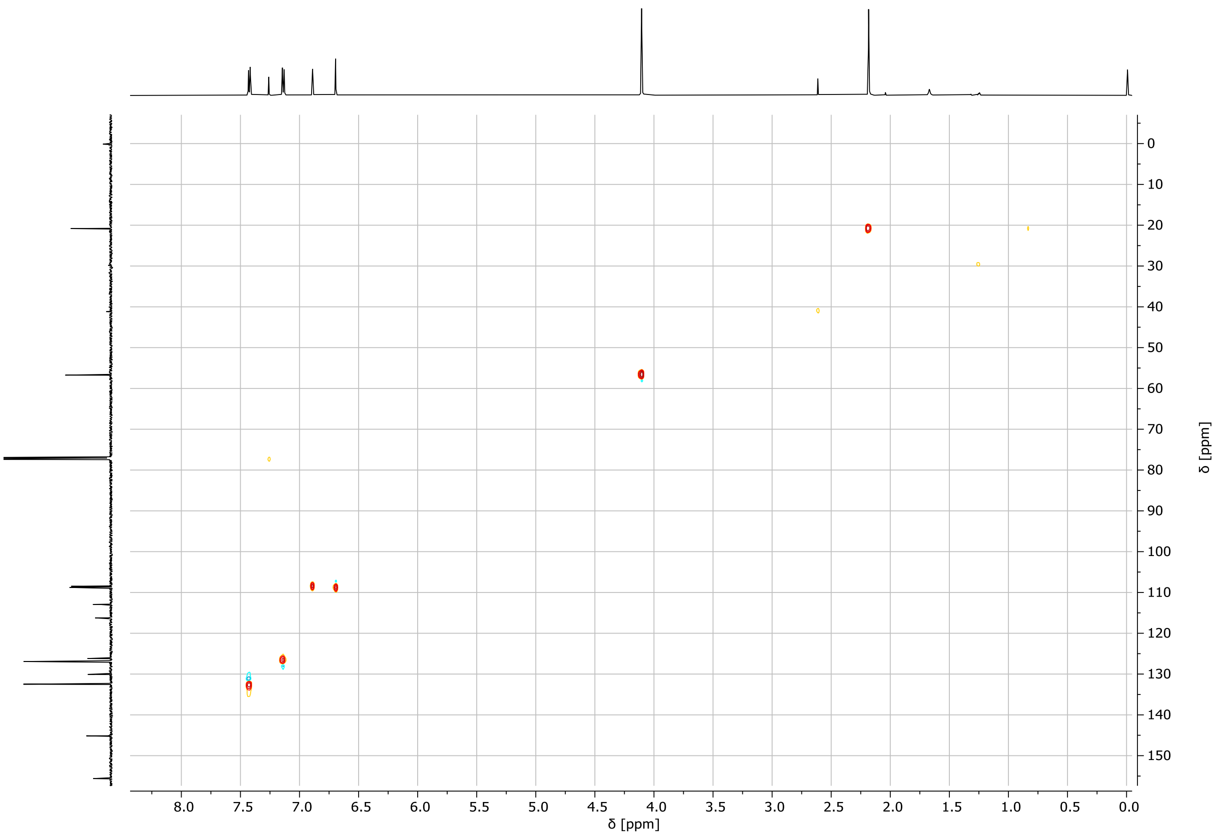


Figure S89: HSQC spectrum ($CDCl_3$) of 2,2'-bis(4-bromophenyl)-5,5'-dimethoxy-7,7'-dimethyl-4H,4'H-[8,8'-bichromene]-4,4'-dione (**2**).

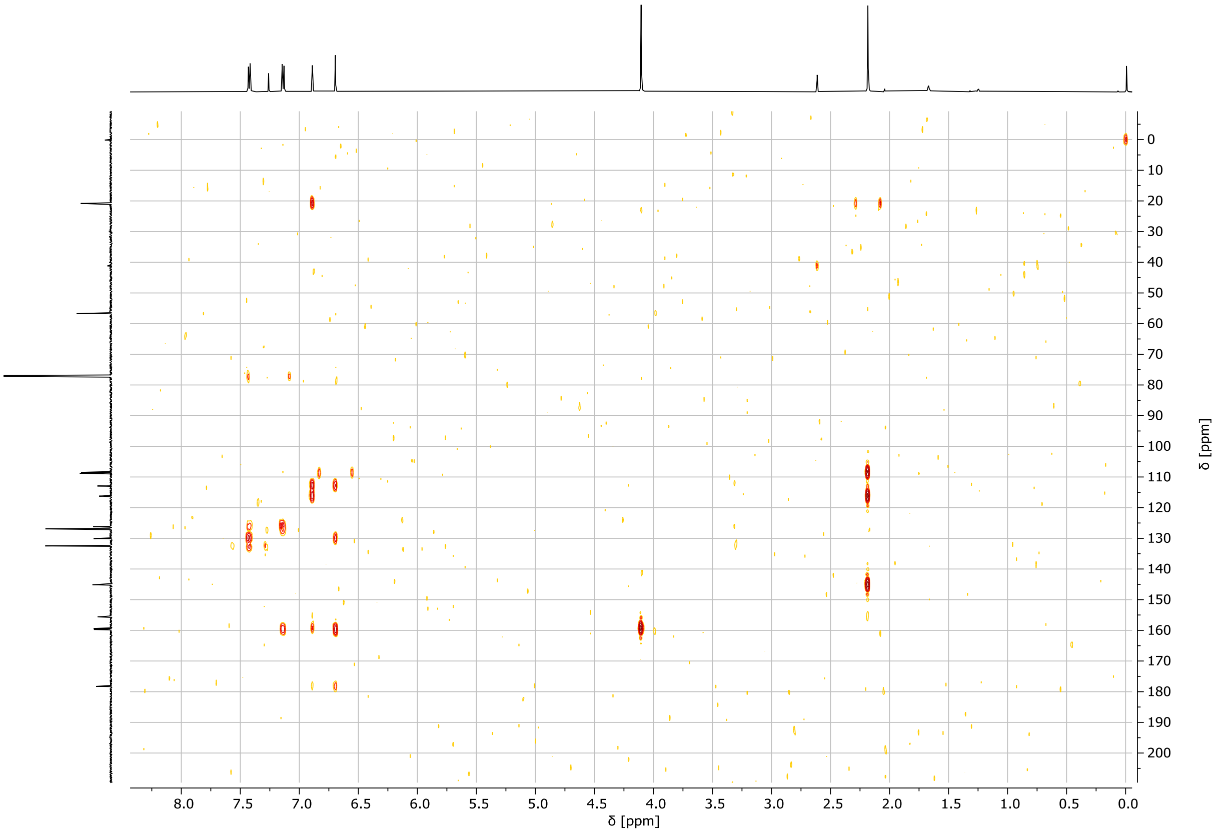


Figure S90: HMBC spectrum ($CDCl_3$) of 2,2'-bis(4-bromophenyl)-5,5'-dimethoxy-7,7'-dimethyl-4H,4'H-[8,8'-bichromene]-4,4'-dione (**2**).

### 3.1.2 Biflavones

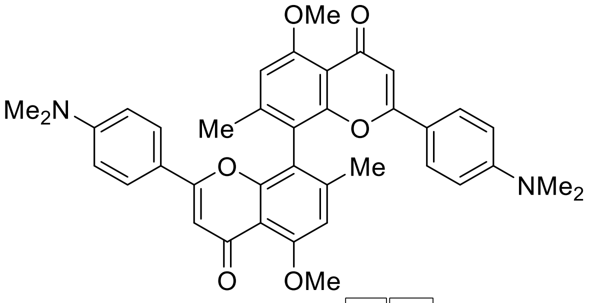


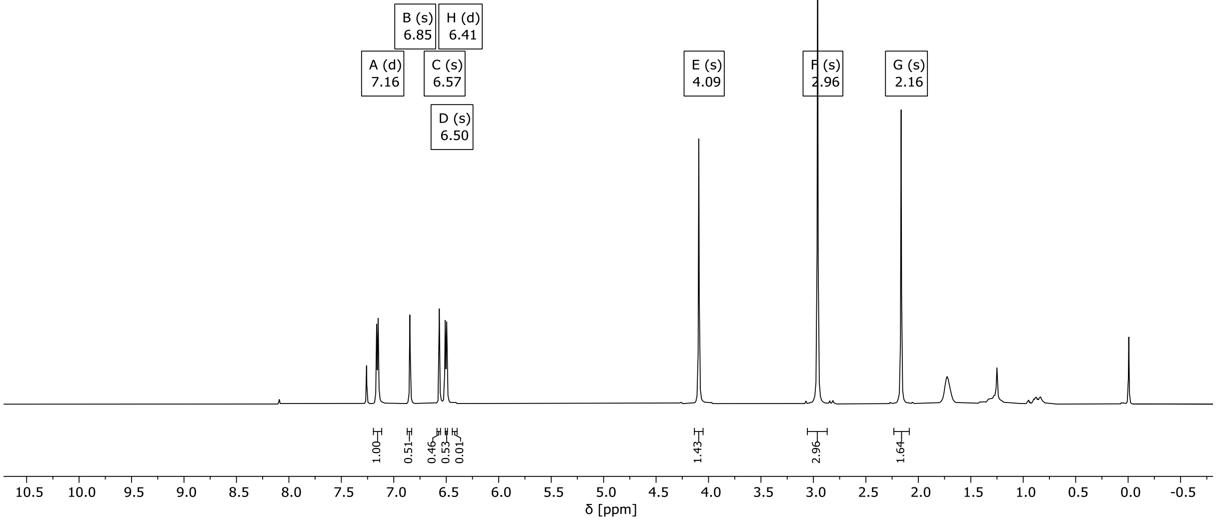


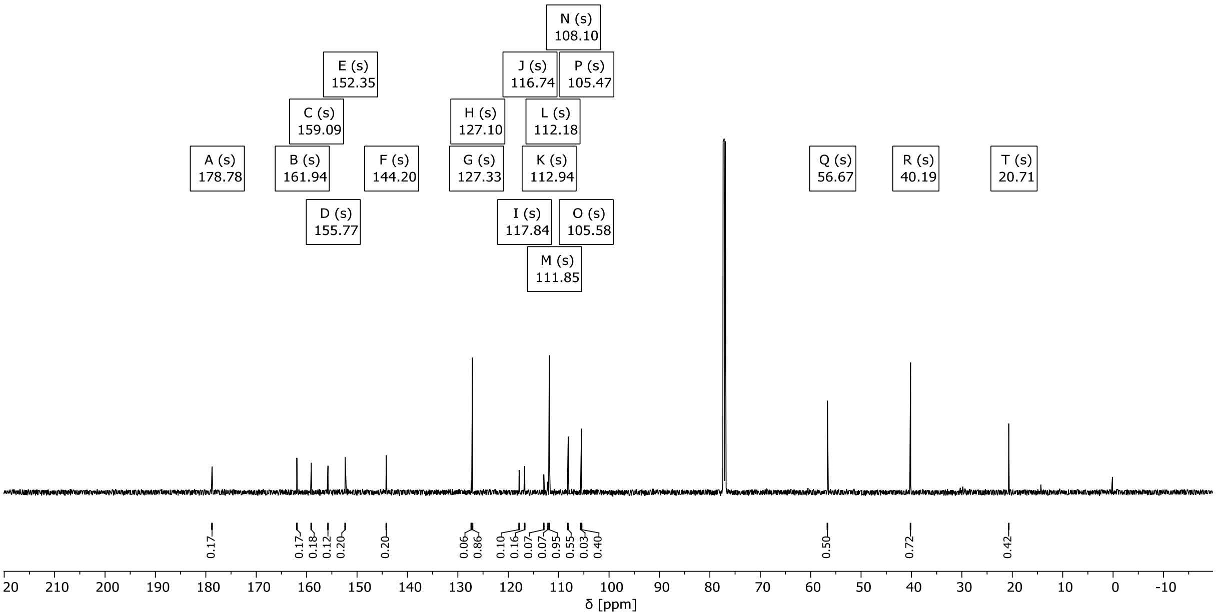


Figure S91: $^{1}H$- and $^{13}C$-NMR spectrum (600 / 151 MHz, $CDCl_3$) of *rac*-2,2'-bis(4-(dimethylamino)phenyl)-5,5'-dimethoxy-7,7'-dimethyl-4H,4'H-[8,8'-bichromene]-4,4'-dione (**14**).

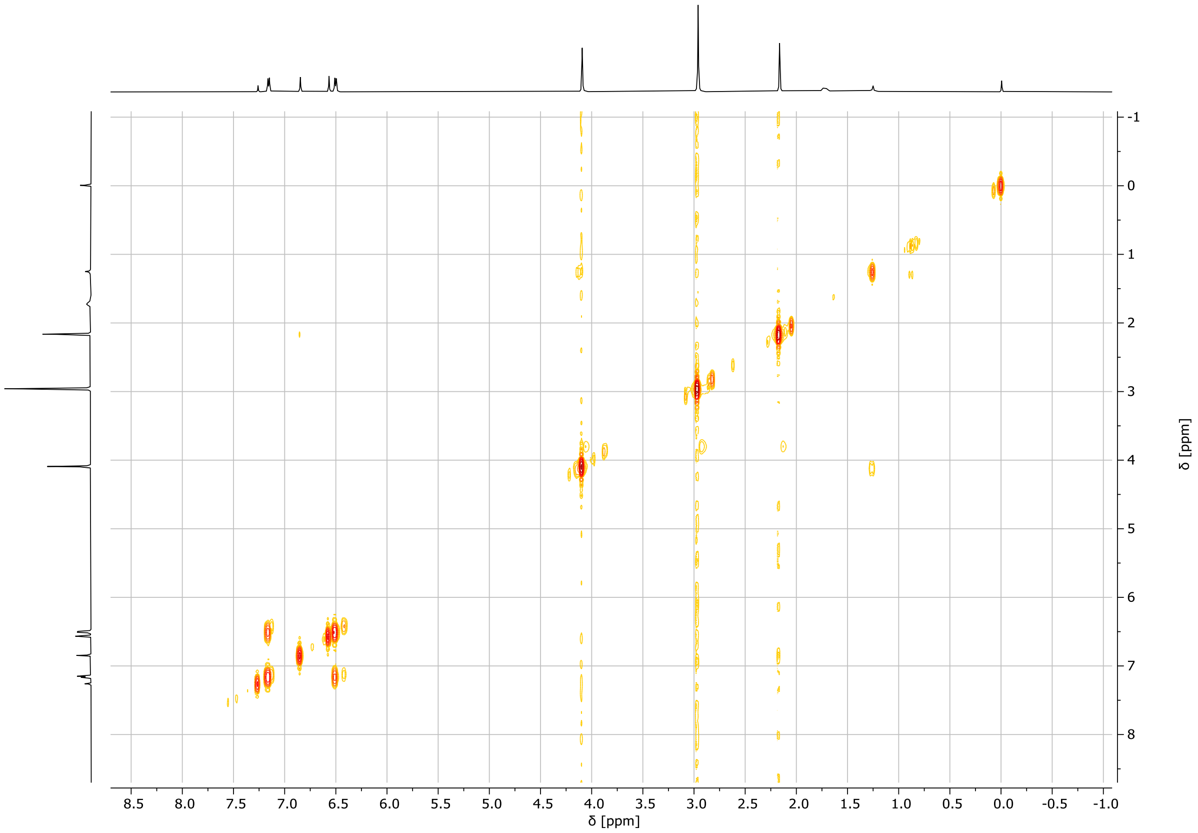


Figure S92: COSY spectrum ($CDCl_3$) of *rac*-2,2'-bis(4-(dimethylamino)phenyl)-5,5'-dimethoxy-7,7'-dimethyl-4H,4'H-[8,8'-bichromene]-4,4'-dione (**14**).

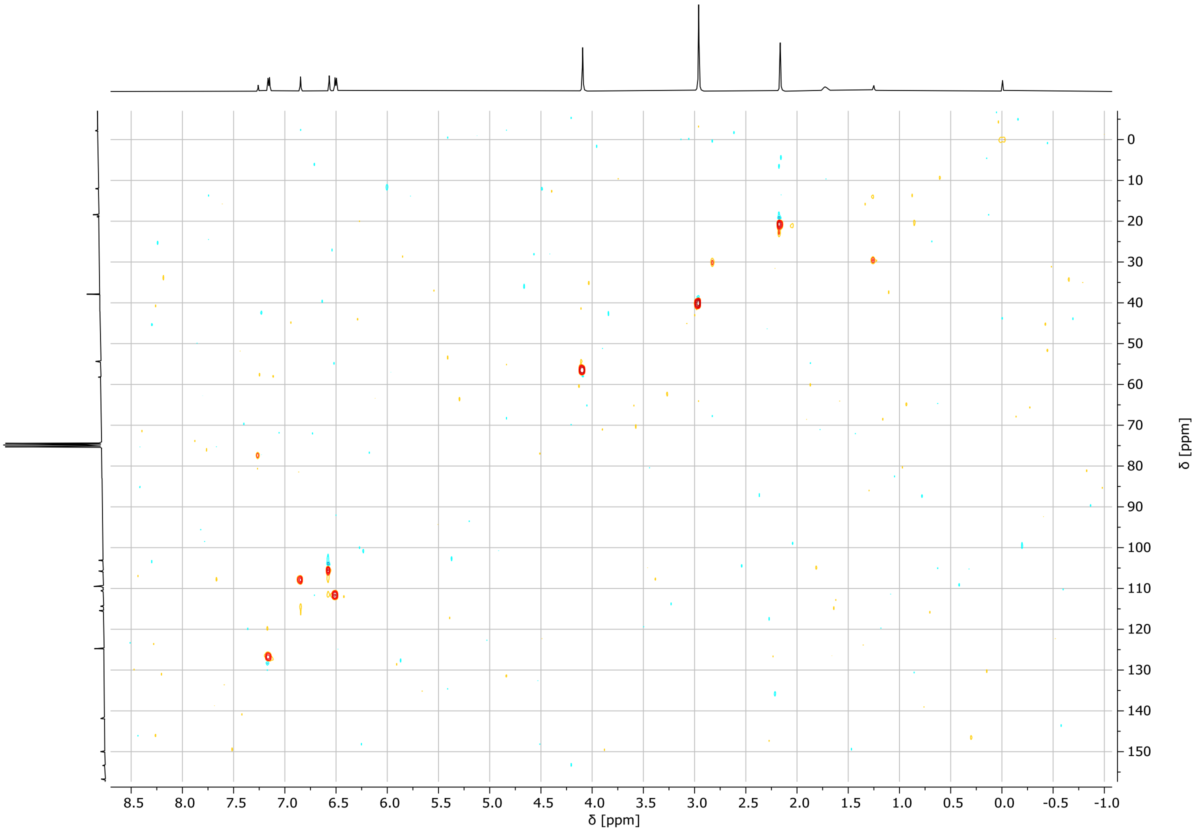


Figure S93: HSQC spectrum ($CDCl_3$) of *rac*-2,2'-bis(4-(dimethylamino)phenyl)-5,5'-dimethoxy-7,7'-dimethyl-4H,4'H-[8,8'-bichromene]-4,4'-dione (**14**).

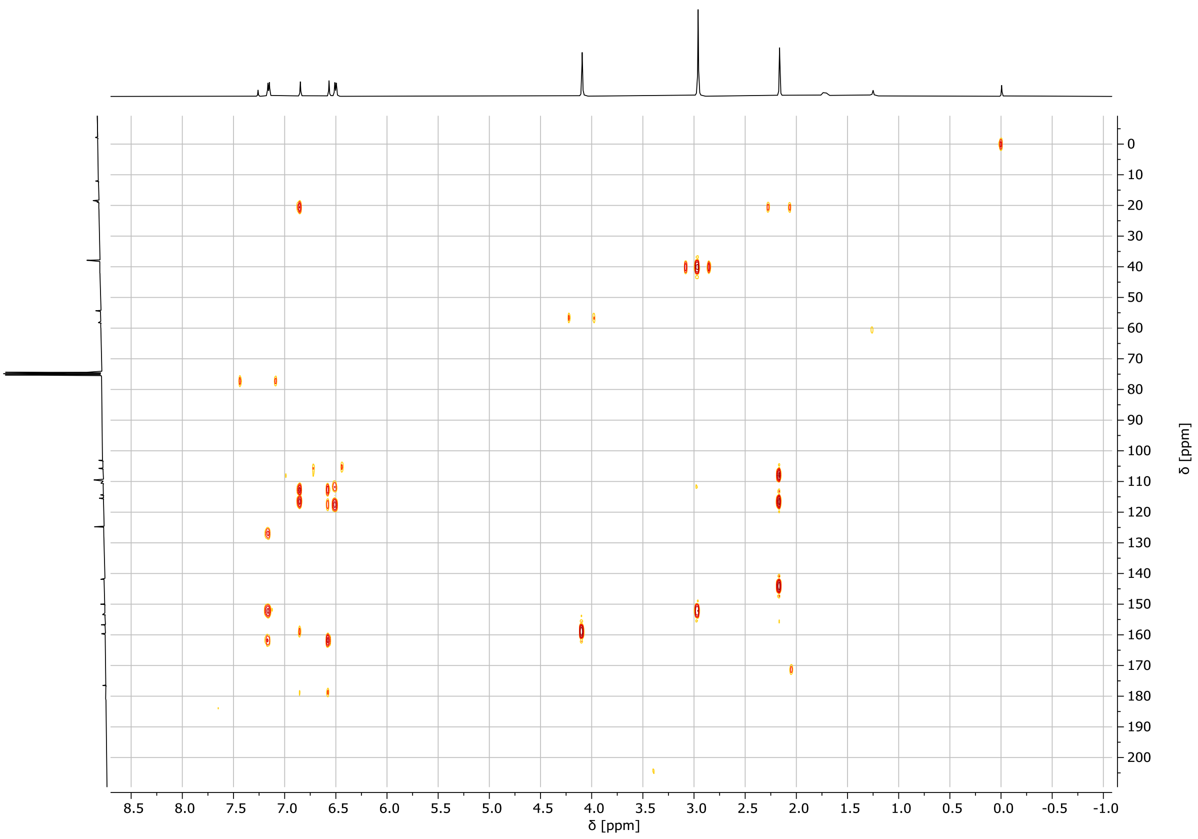


Figure S94: HMBC spectrum ($CDCl_3$) of *rac*-2,2'-bis(4-(dimethylamino)phenyl)-5,5'-dimethoxy-7,7'-dimethyl-4H,4'H-[8,8'-bichromene]-4,4'-dione (**14**).

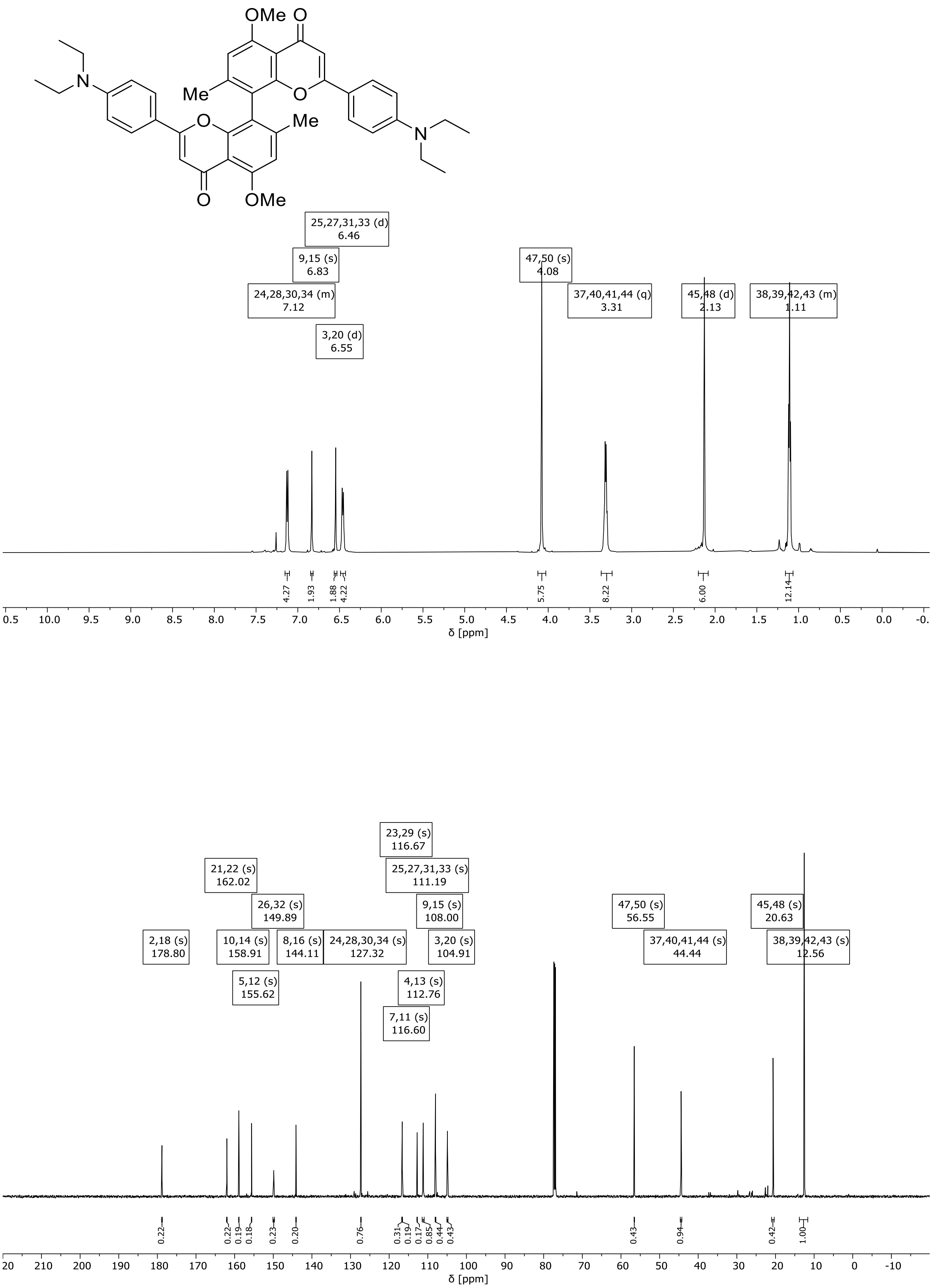


Figure S95: $^{1}H$- and $^{13}C$-NMR spectra (600 / 151 MHz, $CDCl_3$) of 2,2'-bis(4-(diethylamino)phenyl)-5,5'-dimethoxy-7,7'-dimethyl-4H,4'H-[8,8'-bichromene]-4,4'-dione (**15**).

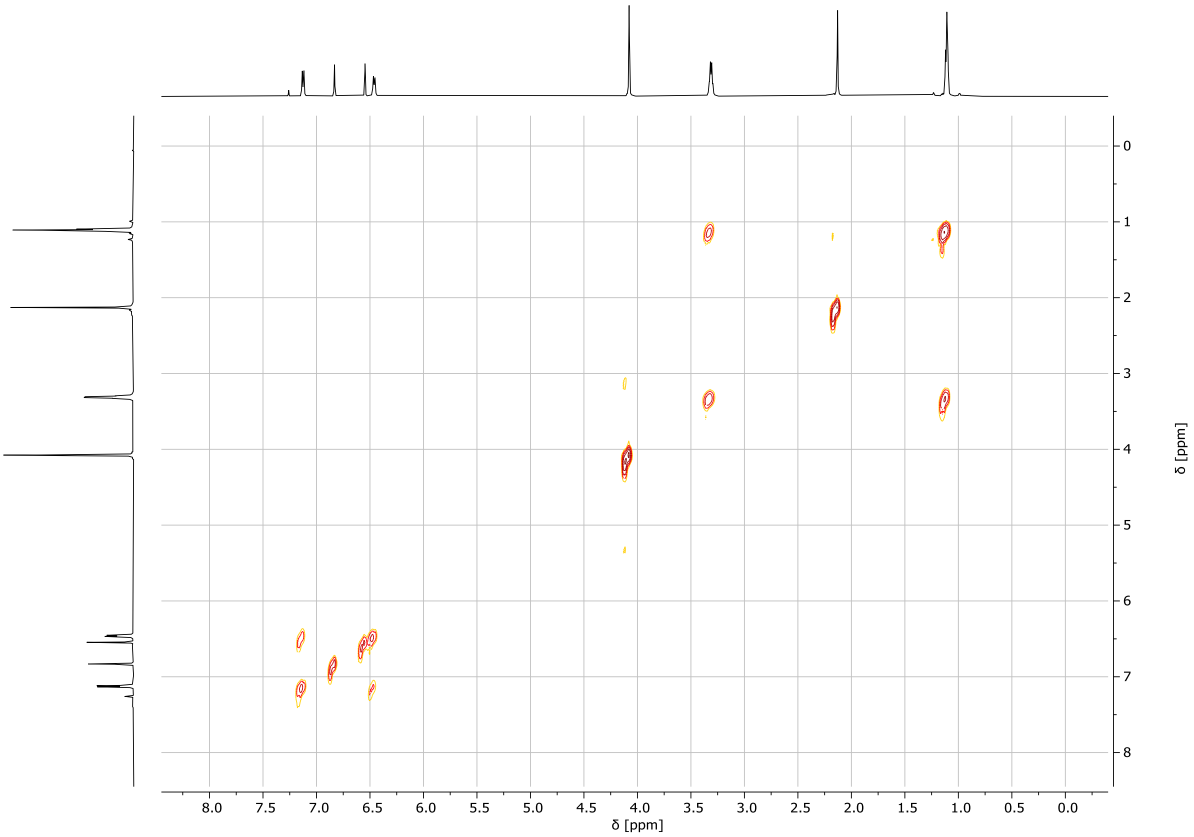


Figure S96: COSY spectrum ($CDCl_3$) of 2,2'-bis(4-(diethylamino)phenyl)-5,5'-dimethoxy-7,7'-dimethyl-4H,4'H-[8,8'-bichromene]-4,4'-dione (**15**).

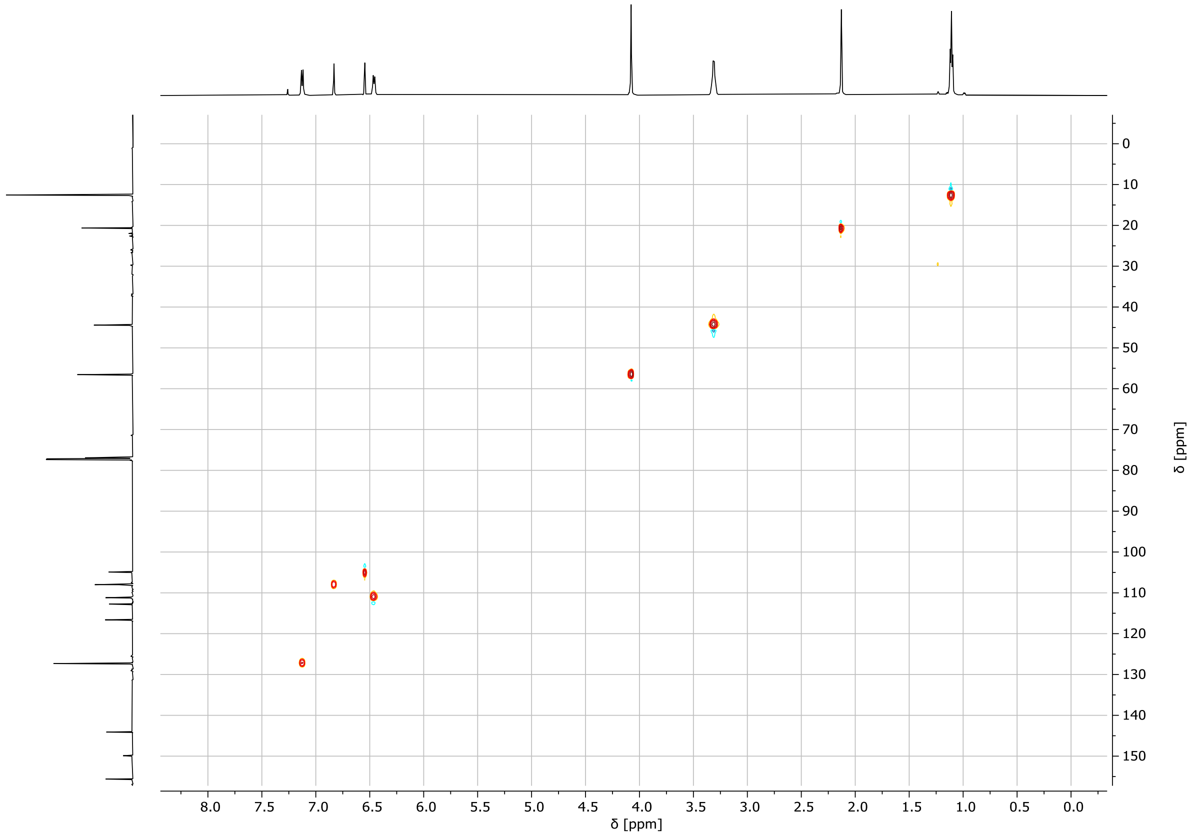


Figure S97: HSQC spectrum ($CDCl_3$) of 2,2'-bis(4-(diethylamino)phenyl)-5,5'-dimethoxy-7,7'-dimethyl-4H,4'H-[8,8'-bichromene]-4,4'-dione (**15**).

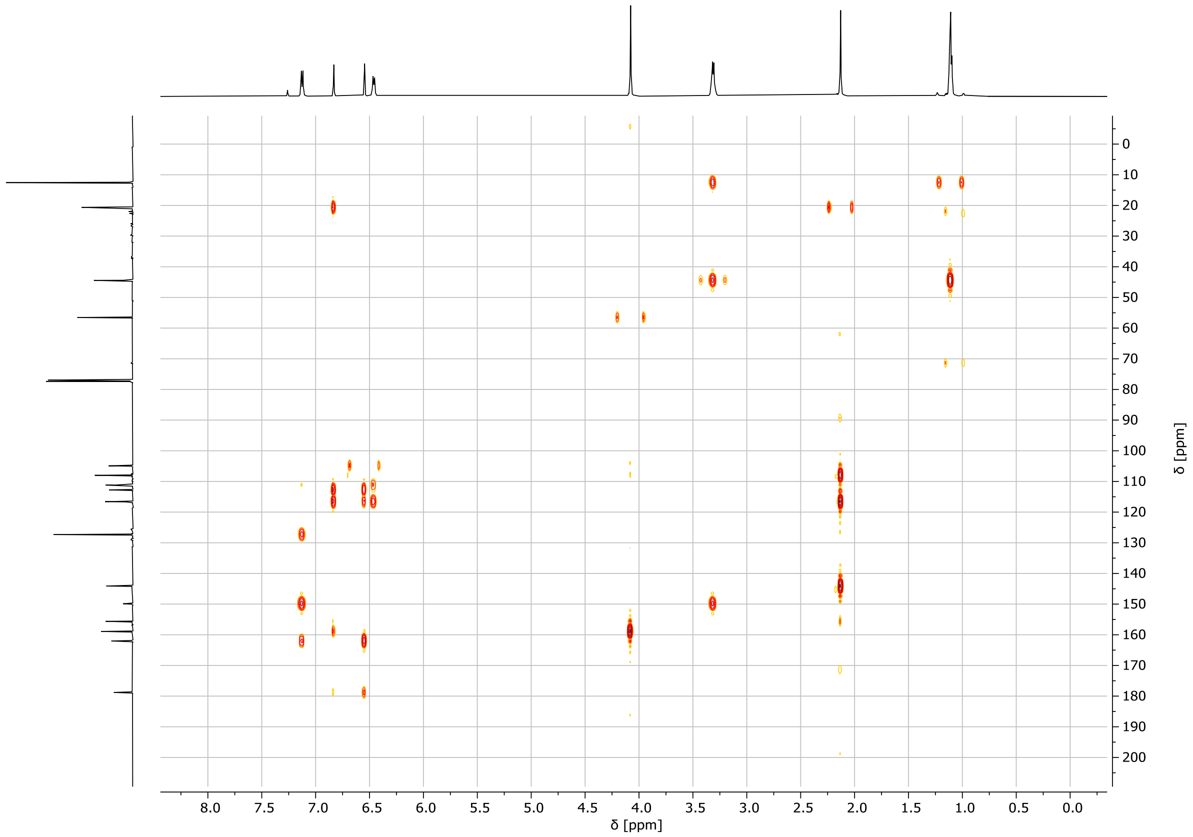


Figure S98: HMBC spectrum (CDCl3) of 2,2'-bis(4-(diethylamino)phenyl)-5,5'-dimethoxy-7,7'-dimethyl-4H,4'H-[8,8'-bichromene]-4,4'-dione (**15**).

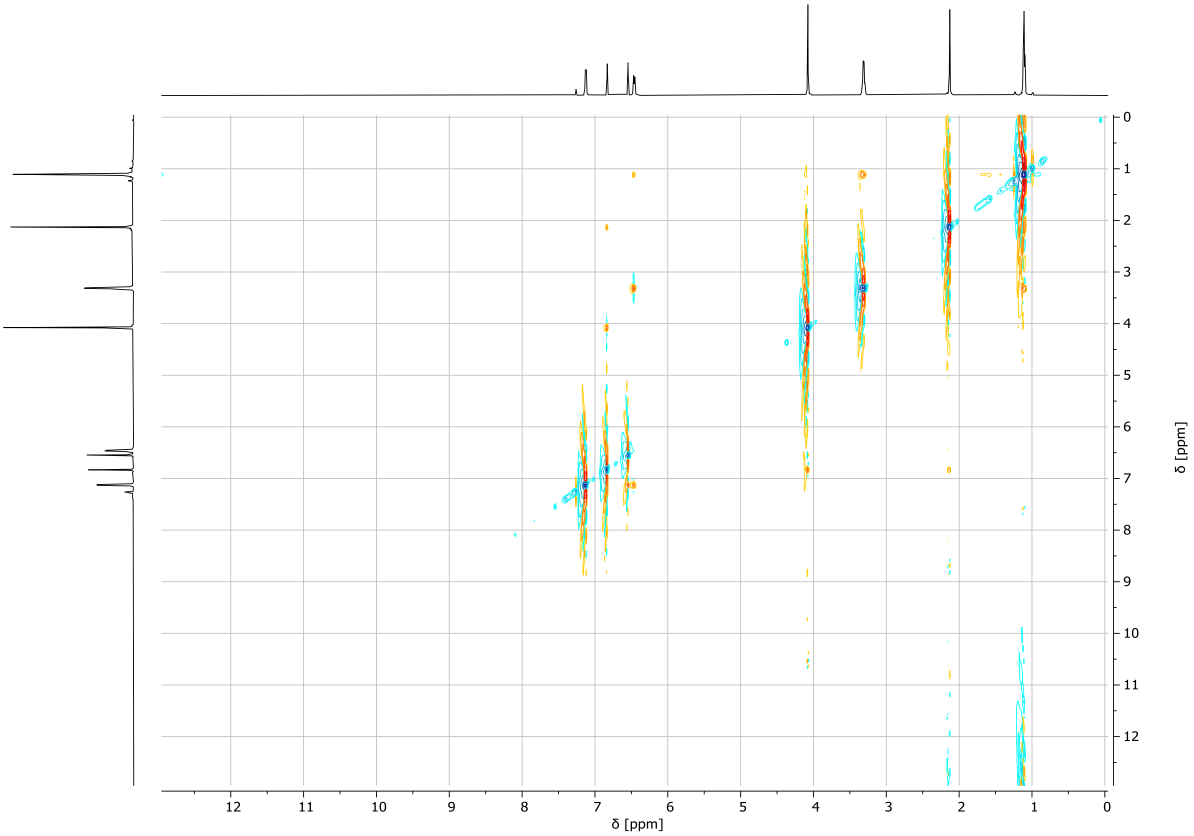


Figure S99: ROESY spectrum (CDCl3) of 2,2'-bis(4-(diethylamino)phenyl)-5,5'-dimethoxy-7,7'-dimethyl-4H,4'H-[8,8'-bichromene]-4,4'-dione (**15**).

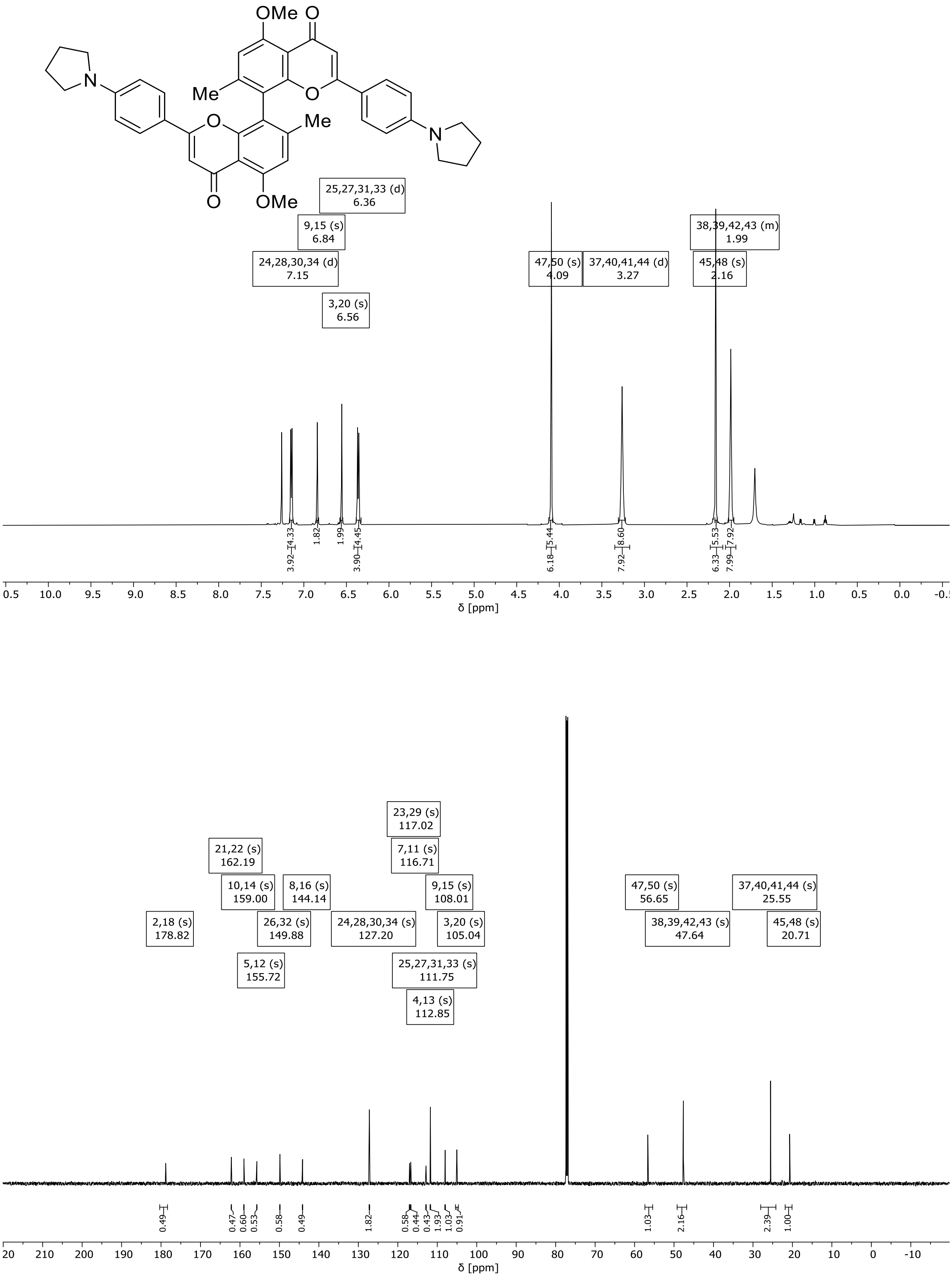


Figure S100: $^{1}$H- and $^{13}$C-NMR spectra (600 / 151 MHz, $CDCl_3$) of 5,5'-dimethoxy-7,7'-dimethyl-2,2'-bis(4-(pyrrolidin-1-yl)phenyl)-4H,4'H-[8,8'-bichromene]-4,4'-dione (**16**).

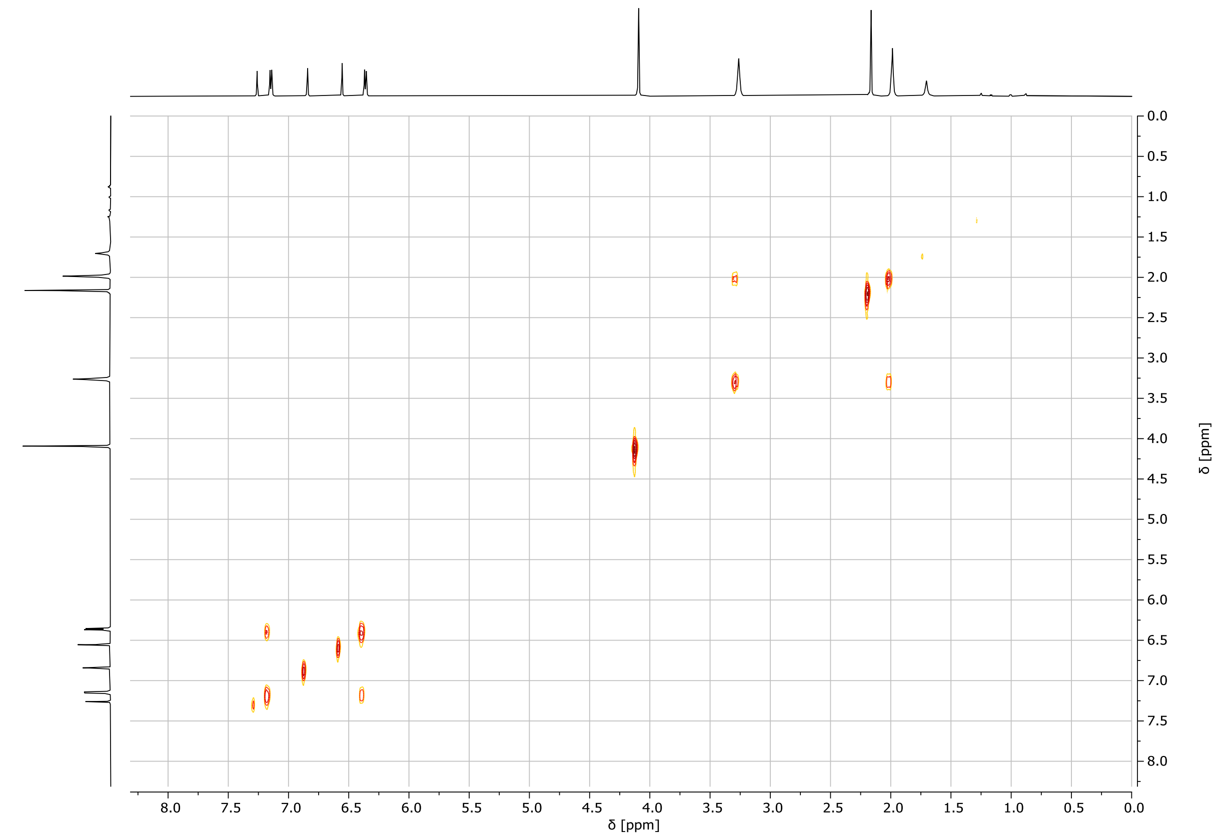


Figure S101: COSY spectrum (CDCl3) of 5,5'-dimethoxy-7,7'-dimethyl-2,2'-bis(4-(pyrrolidin-1-yl)phenyl)-4H,4'H-[8,8'-bichromene]-4,4'-dione (**16)**.

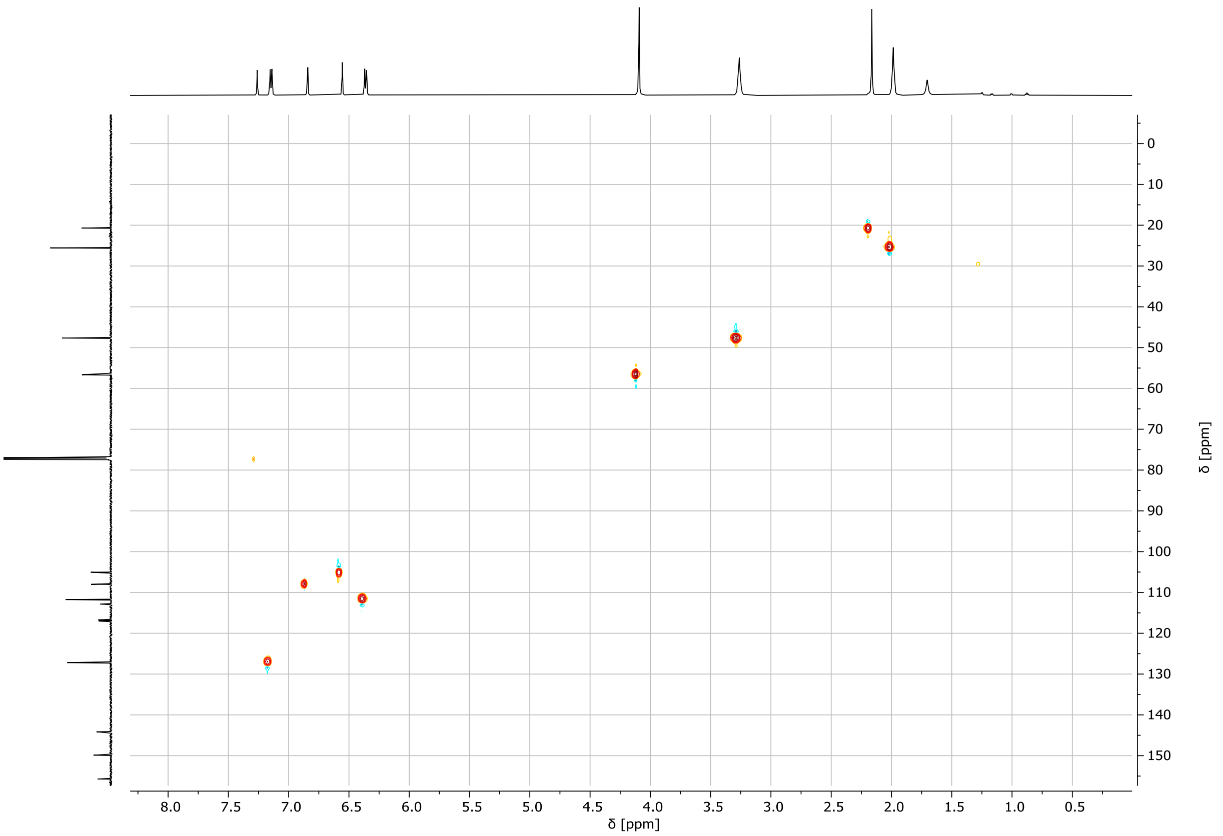


Figure S102: HSQC spectrum (CDCl3) of 5,5'-dimethoxy-7,7'-dimethyl-2,2'-bis(4-(pyrrolidin-1-yl)phenyl)-4H,4'H-[8,8'-bichromene]-4,4'-dione (**16)**.

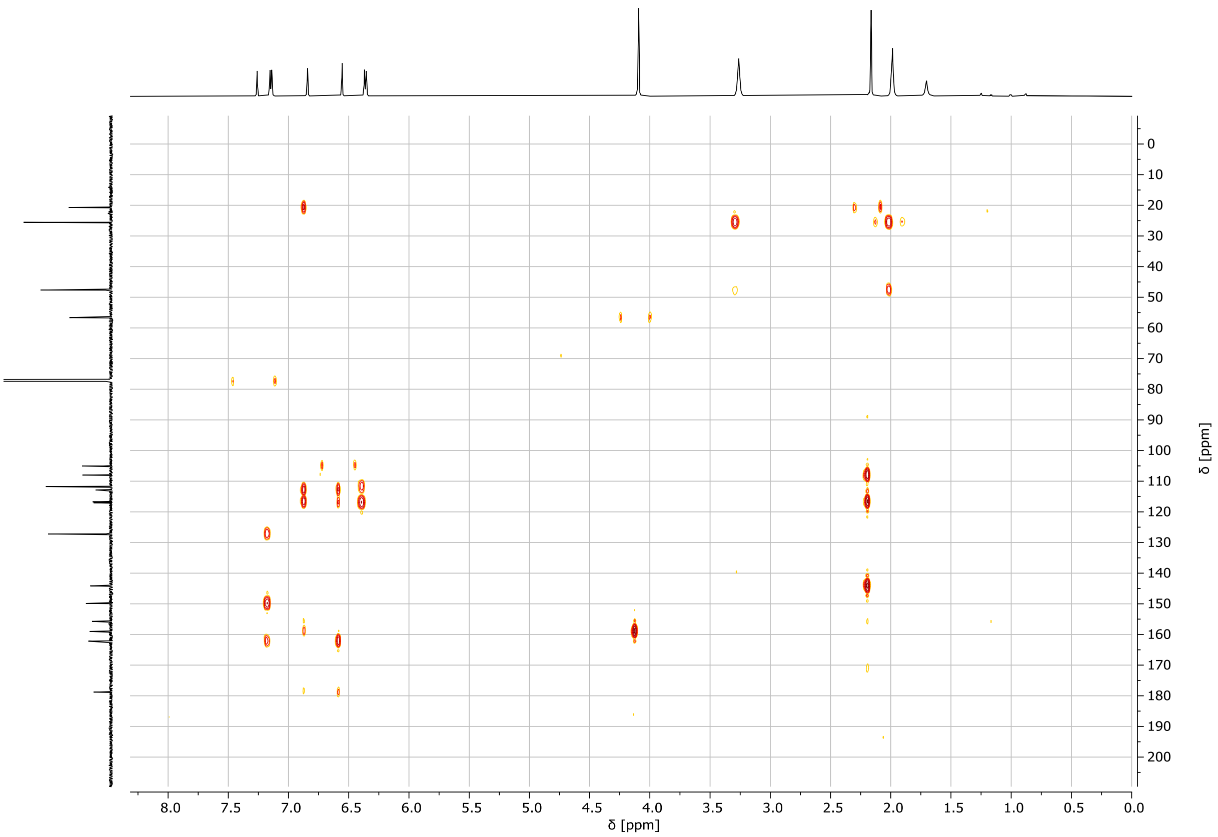


Figure S103: HMBC spectrum ($CDCl_3$) of 5,5'-dimethoxy-7,7'-dimethyl-2,2'-bis(4-(pyrrolidin-1-yl)phenyl)-4H,4'H-[8,8'-bichromene]-4,4'-dione (**16**).

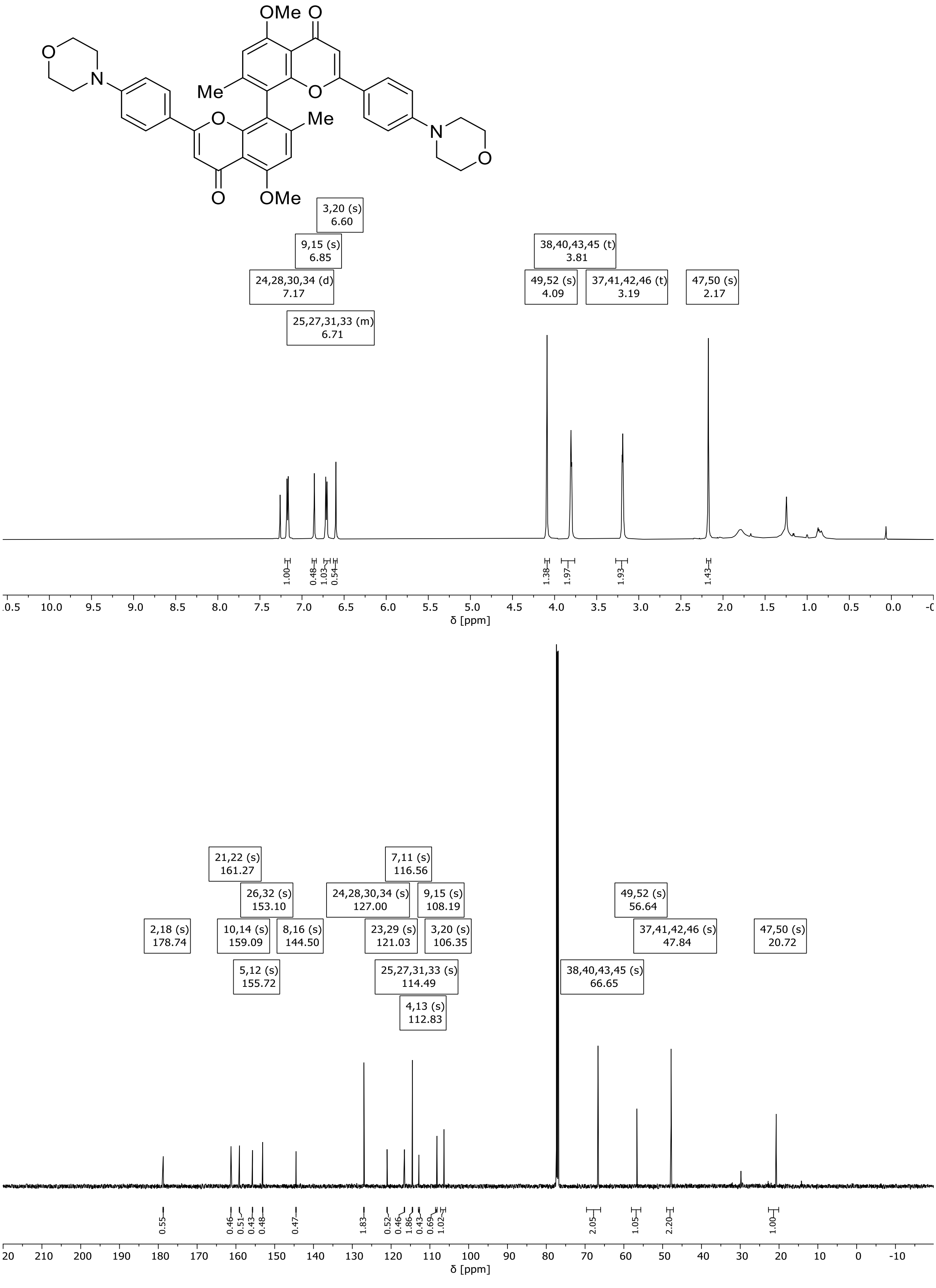


Figure S104: $^{1}$H- and $^{13}$C-NMR spectra (600 / 151 MHz, $CDCl_3$) of 5,5'-dimethoxy-7,7'-dimethyl-2,2'-bis(4-morpholinophenyl)-4H,4'H-[8,8'-bichromene]-4,4'-dione (**17**).

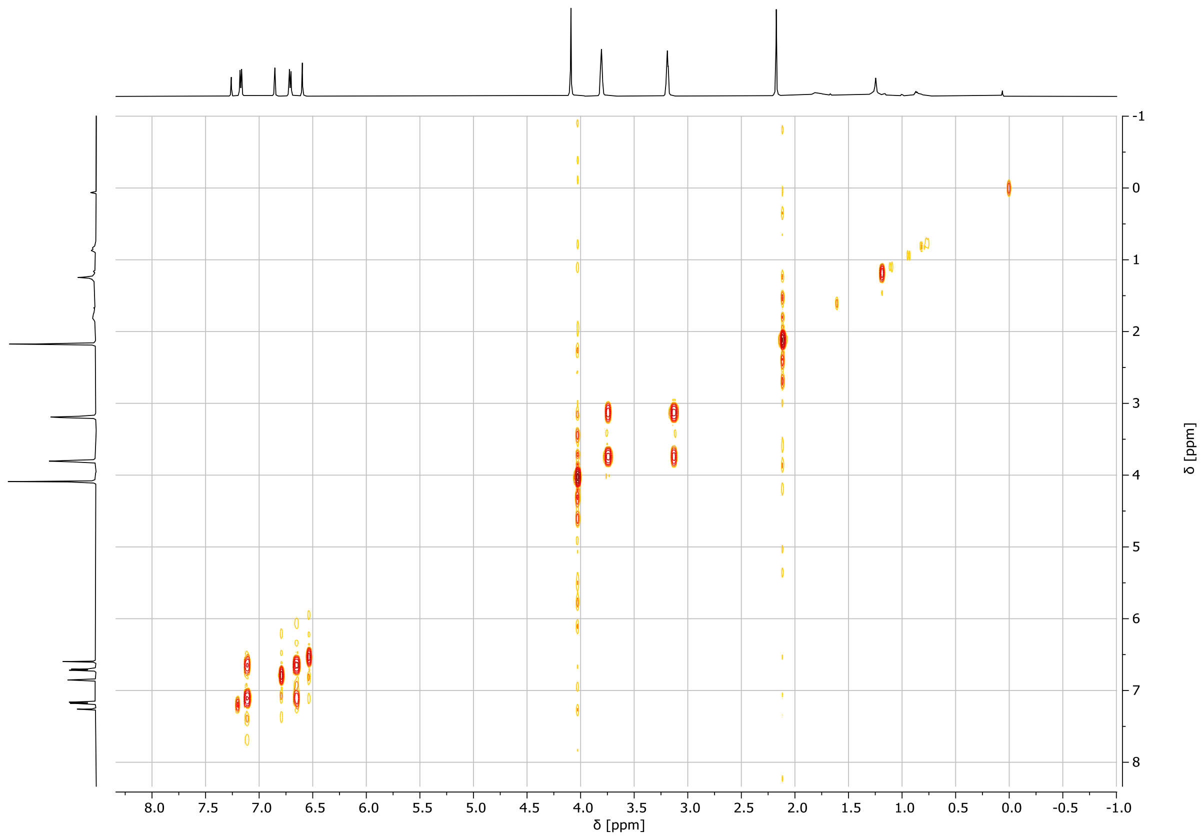


Figure S105: COSY spectrum (CDCl3) of 5,5'-dimethoxy-7,7'-dimethyl-2,2'-bis(4-morpholinophenyl)-4H,4'H-[8,8'-bichromene]-4,4'-dione (**17**).

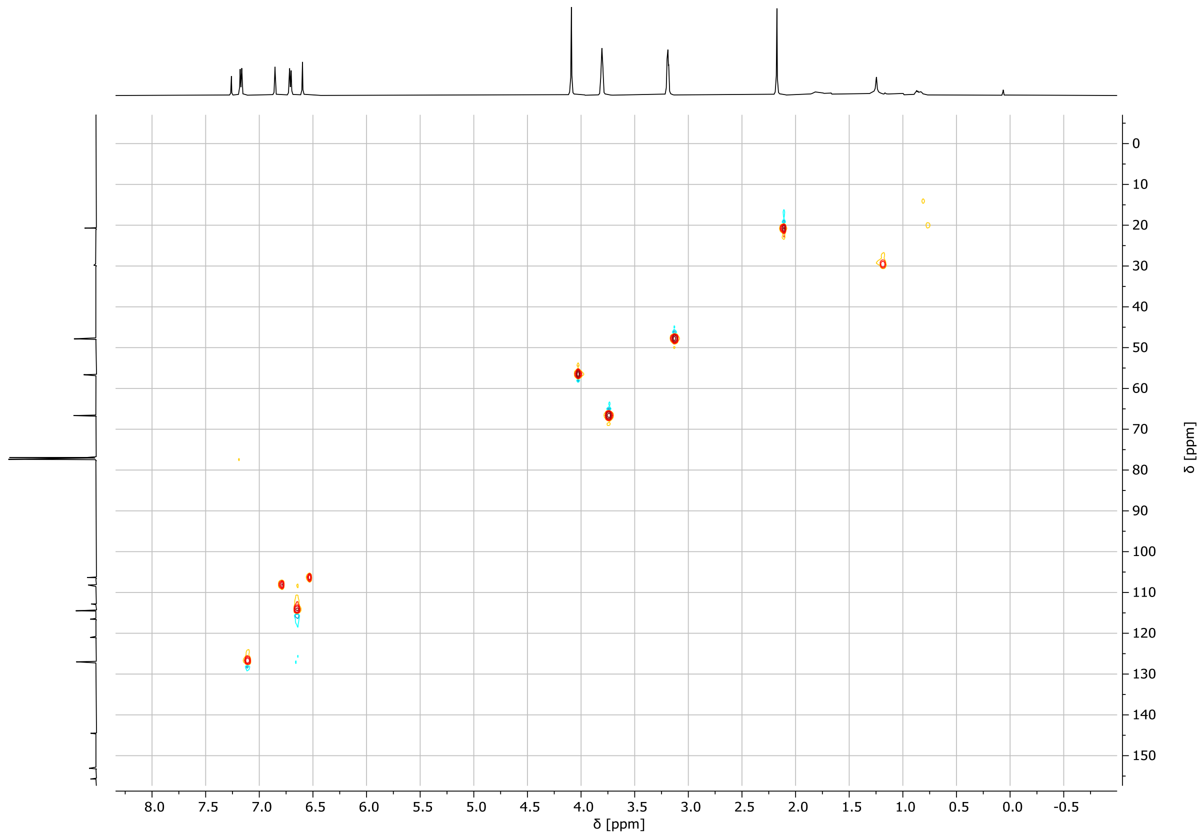


Figure S106: HSQC spectrum (CDCl3) of 5,5'-dimethoxy-7,7'-dimethyl-2,2'-bis(4-morpholinophenyl)-4H,4'H-[8,8'-bichromene]-4,4'-dione (**17**).

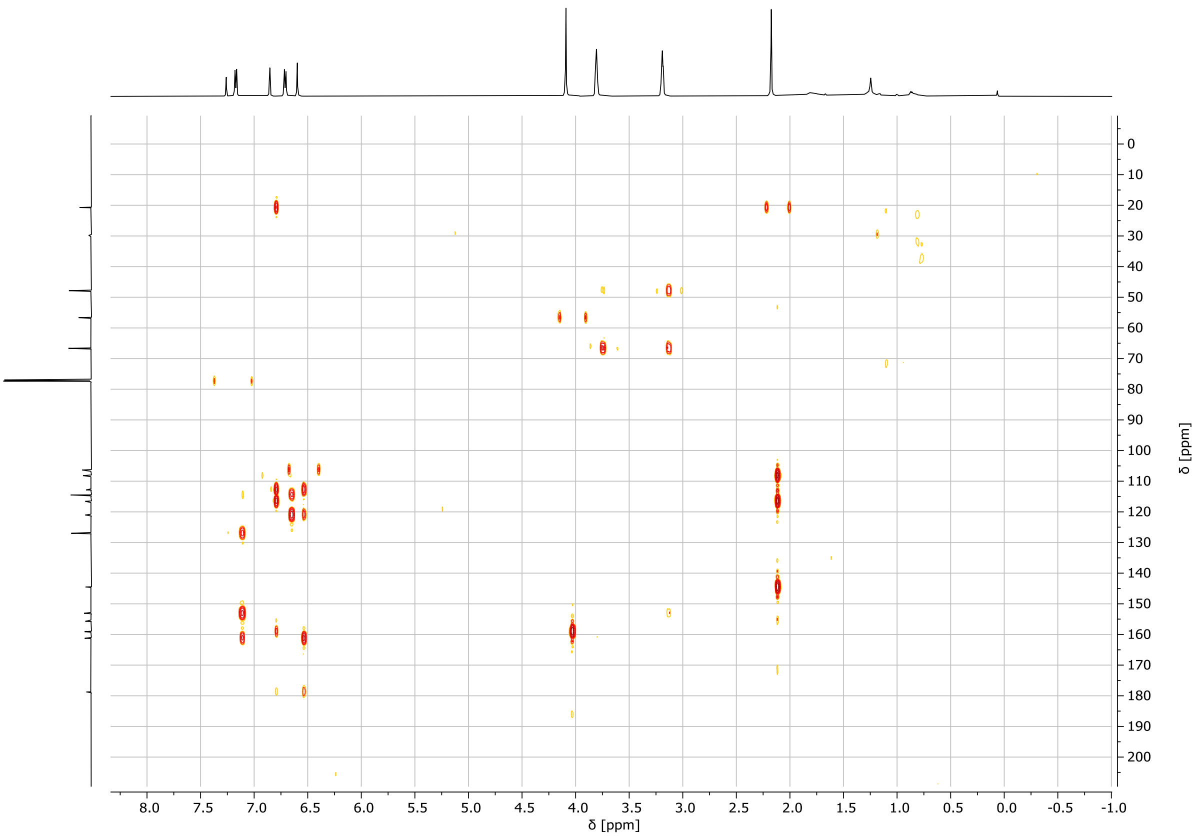


Figure S107: HMBC spectrum ($CDCl_3$) of 5,5'-dimethoxy-7,7'-dimethyl-2,2'-bis(4-morpholinophenyl)-4H,4'H-[8,8'-bichromene]-4,4'-dione (**17**).

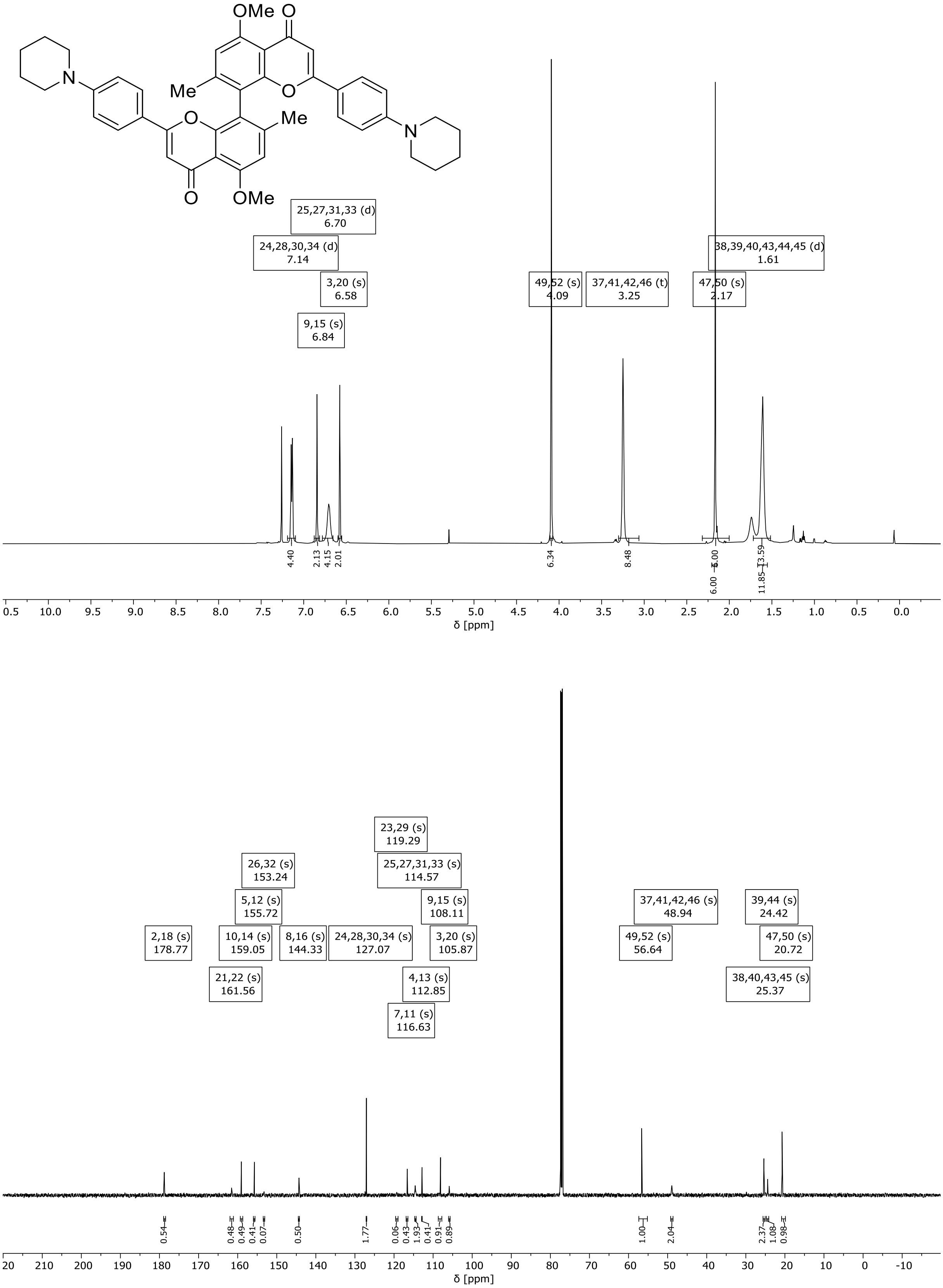


Figure S108: $^{1}H$- and $^{13}C$-NMR spectra (600 / 151 MHz, $CDCl_3$) of 5,5'-dimethoxy-7,7'-dimethyl-2,2'-bis(4-(piperidin-1-yl)phenyl)-4H,4'H-[8,8'-bichromene]-4,4'-dione (**18**).

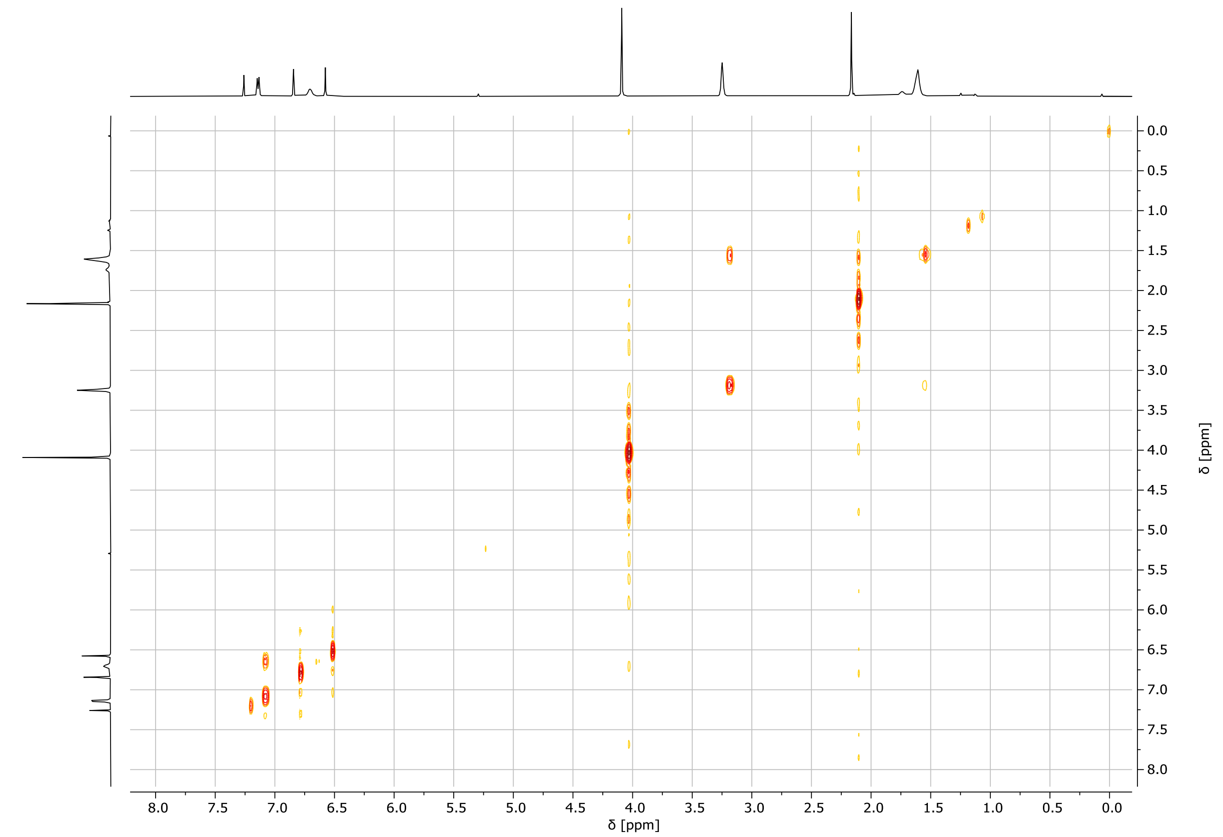


Figure S109: COSY spectrum ($CDCl_3$) of 5,5'-dimethoxy-7,7'-dimethyl-2,2'-bis(4-(piperidin-1-yl)phenyl)-4H,4'H-[8,8'-bichromene]-4,4'-dione (**18**).

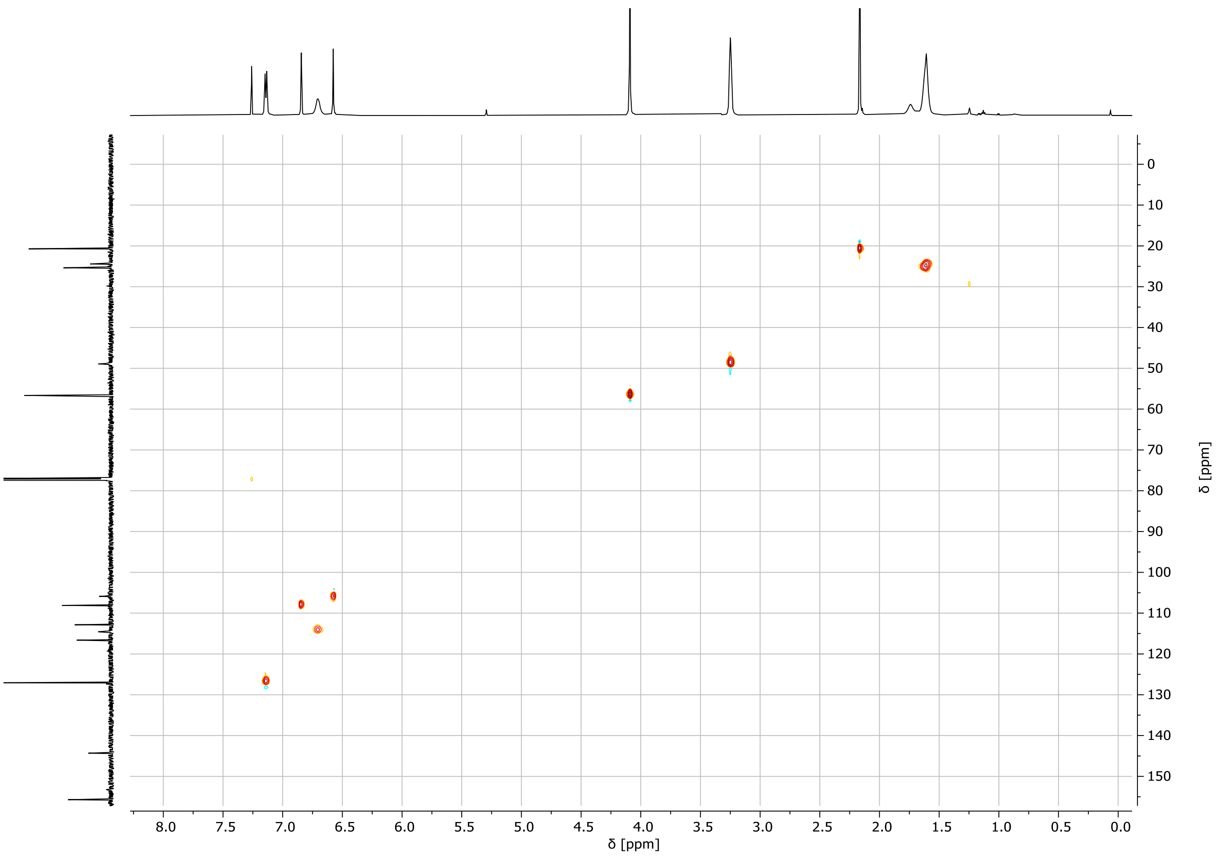


Figure S110: HSQC spectrum ($CDCl_3$) of 5,5'-dimethoxy-7,7'-dimethyl-2,2'-bis(4-(piperidin-1-yl)phenyl)-4H,4'H-[8,8'-bichromene]-4,4'-dione (**18**).

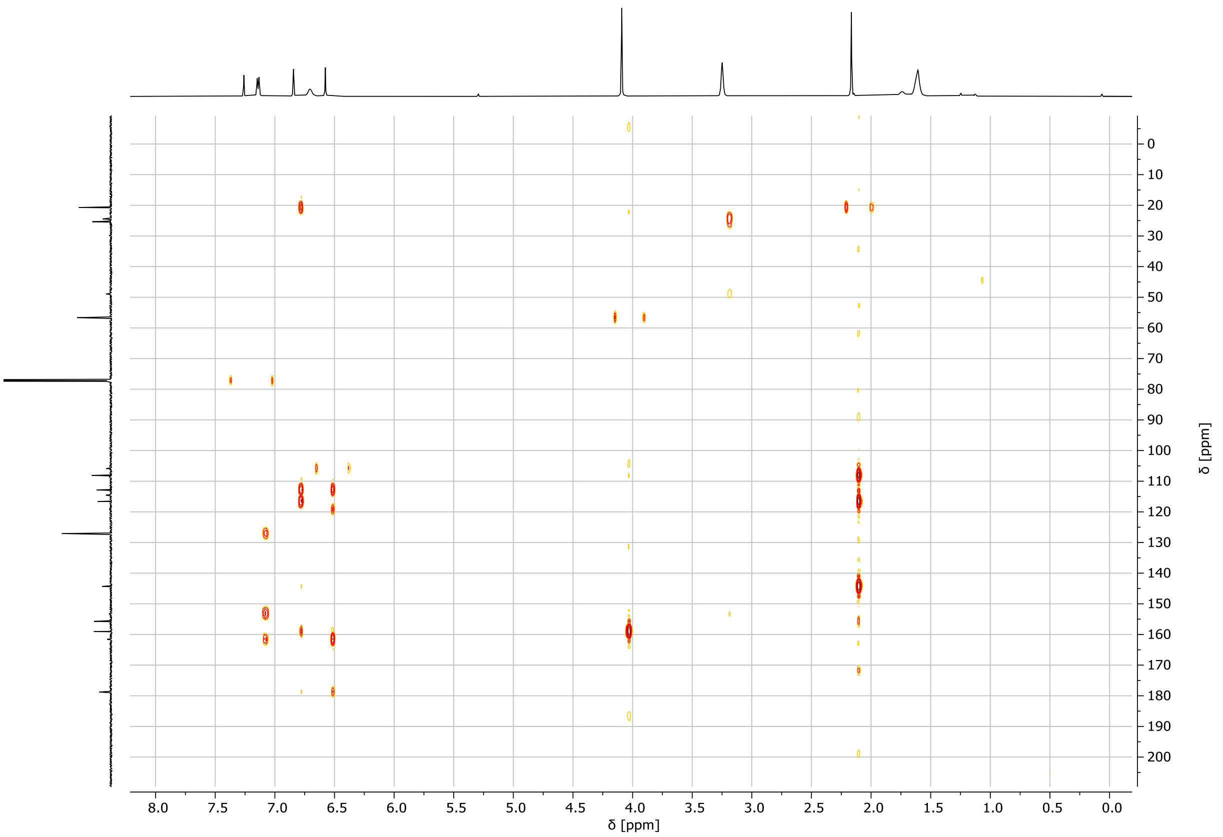


Figure S111: HMBC spectrum ($CDCl_3$) of 5,5'-dimethoxy-7,7'-dimethyl-2,2'-bis(4-(piperidin-1-yl)phenyl)-4H,4'H-[8,8'-bichromene]-4,4'-dione (**18**).

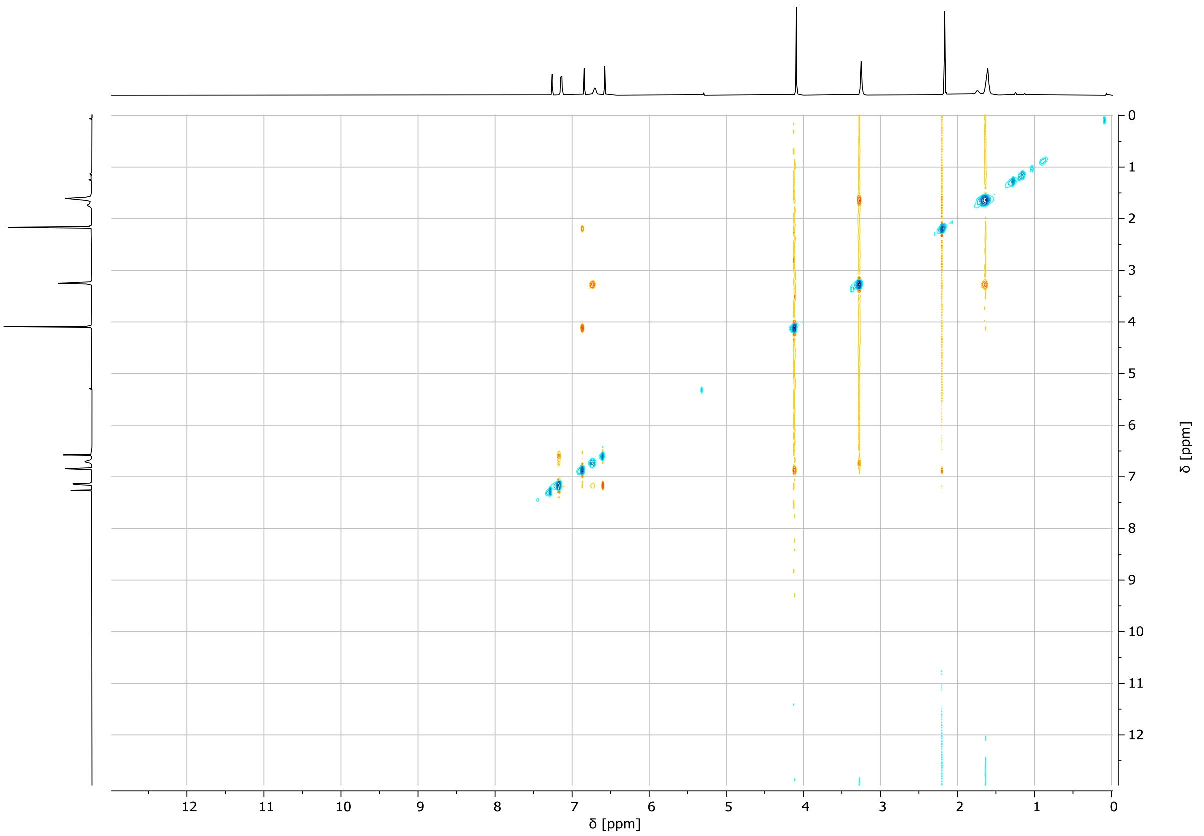


Figure S112: ROESY spectrum ($CDCl_3$) of 5,5'-dimethoxy-7,7'-dimethyl-2,2'-bis(4-(piperidin-1-yl)phenyl)-4H,4'H-[8,8'-bichromene]-4,4'-dione (**18**).

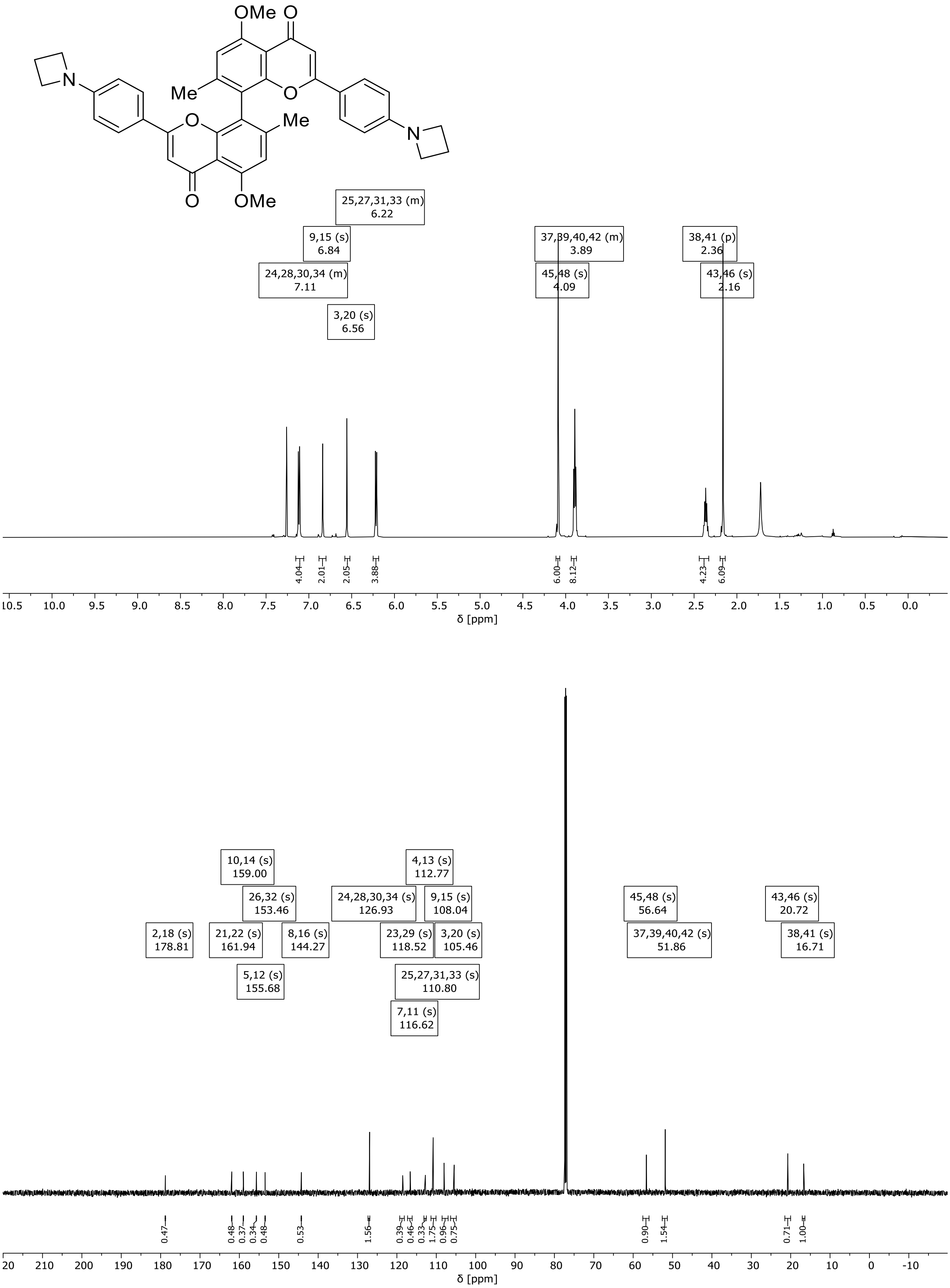


Figure S113: 1H- and 13C-NMR spectra (600 / 151 MHz, CDCl3) of 2,2'-bis(4-(azetidin-1-yl)phenyl)-5,5'-dimethoxy-7,7'-dimethyl-4H,4'H-[8,8'-bichromene]-4,4'-dione (**19**).

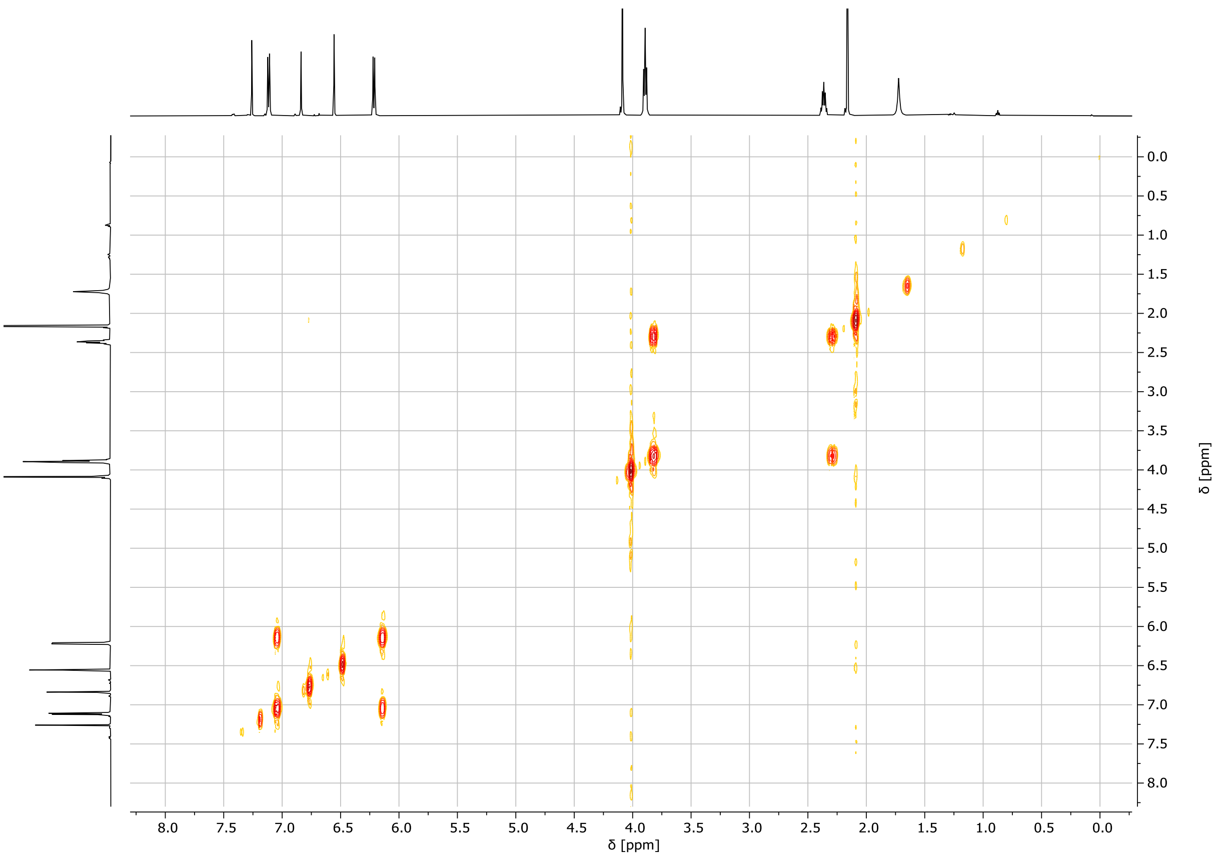


Figure S114: COSY spectrum (CDCl₃) of 2,2'-bis(4-(azetidin-1-yl)phenyl)-5,5'-dimethoxy-7,7'-dimethyl-4H,4'H-[8,8'-bichromene]-4,4'-dione (**19**).

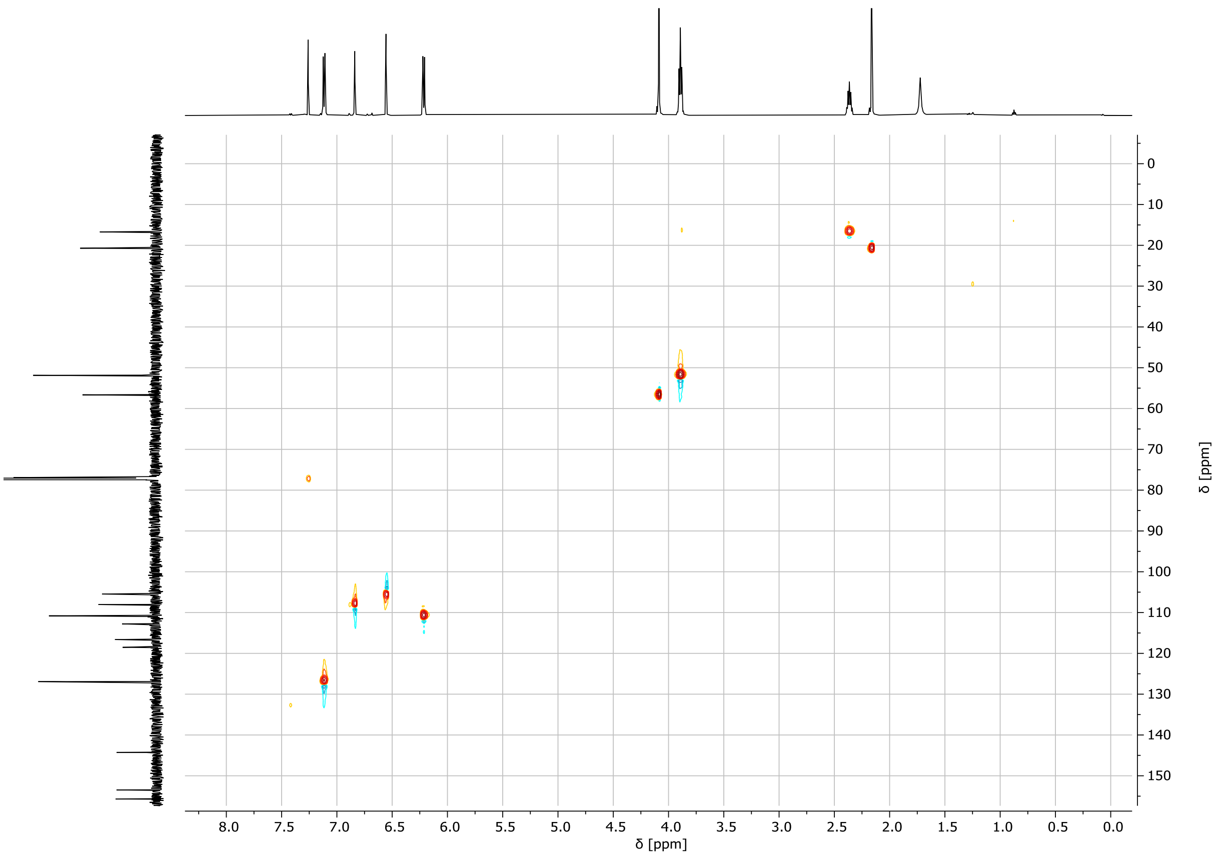


Figure S115: HSQC spectrum (CDCl₃) of 2,2'-bis(4-(azetidin-1-yl)phenyl)-5,5'-dimethoxy-7,7'-dimethyl-4H,4'H-[8,8'-bichromene]-4,4'-dione (**19**).

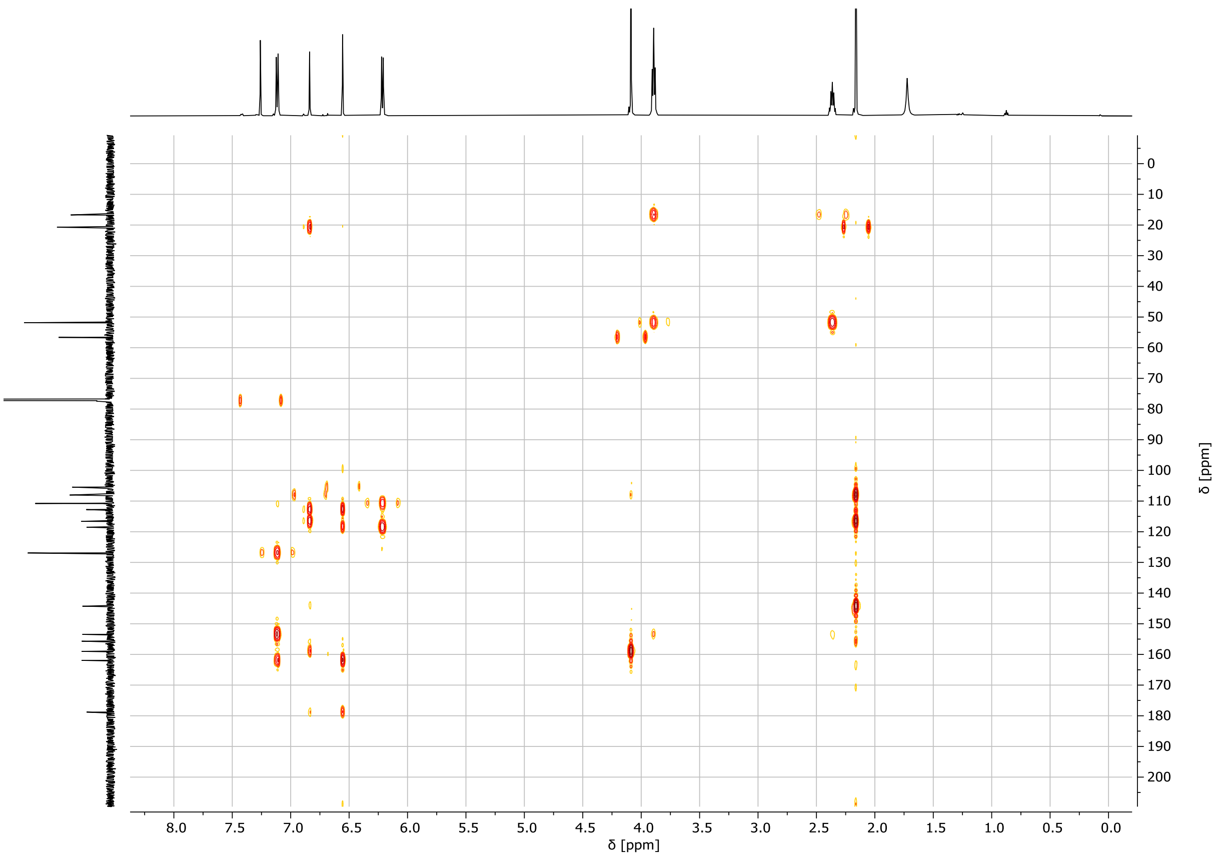


Figure S116: HMBC spectrum ($CDCl_3$) of 2,2'-bis(4-(azetidin-1-yl)phenyl)-5,5'-dimethoxy-7,7'-dimethyl-4H,4'H-[8,8'-bichromene]-4,4'-dione (**19**).

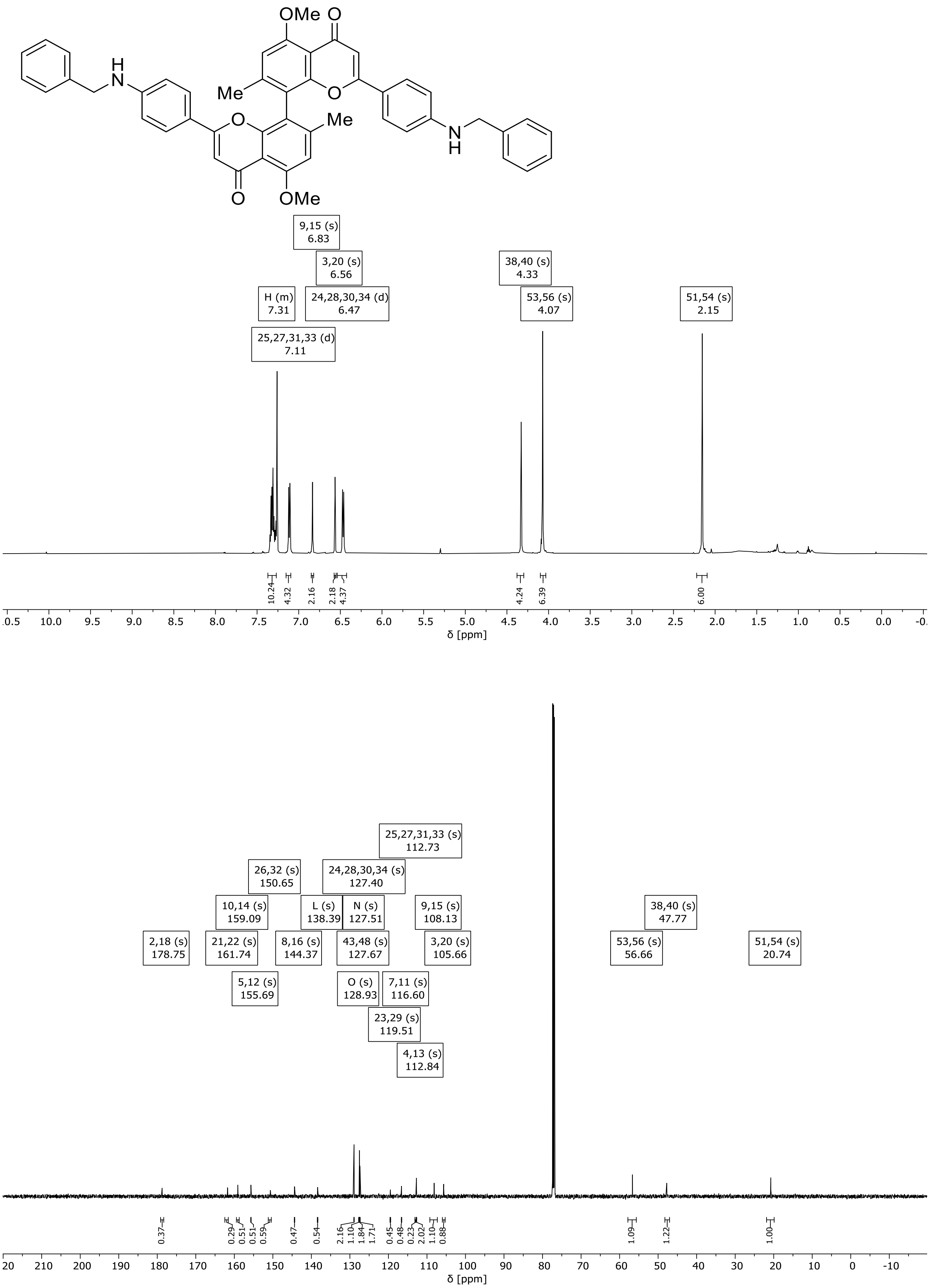


Figure S117: $^{1}H$- and $^{13}C$-NMR spectra (600 / 151 MHz, $CDCl_3$) of 2,2'-bis(4-(benzylamino)phenyl)-5,5'-dimethoxy-7,7'-dimethyl-4H,4'H-[8,8'-bichromene]-4,4'-dione (**20**).

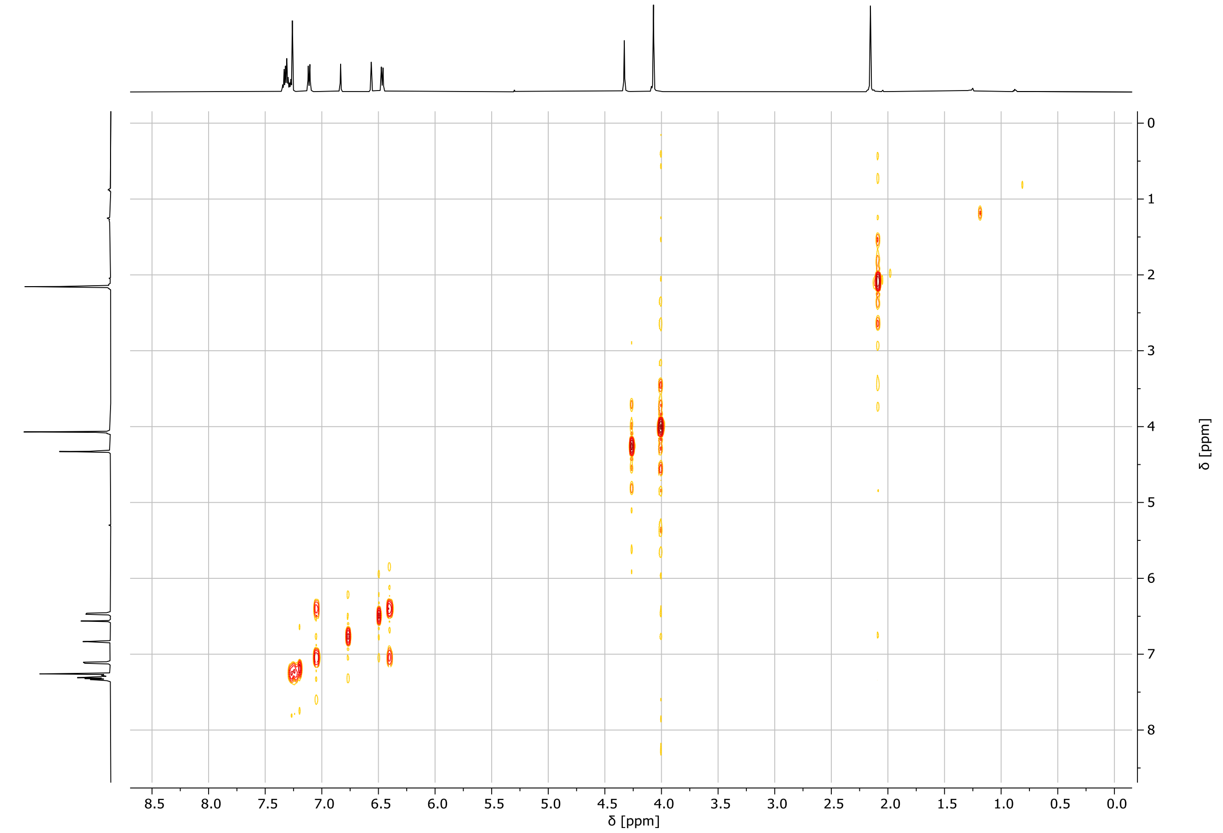


Figure S118: COSY spectrum ($CDCl_3$) of 2,2'-bis(4-(benzylamino)phenyl)-5,5'-dimethoxy-7,7'-dimethyl-4H,4'H-[8,8'-bichromene]-4,4'-dione (**20**).

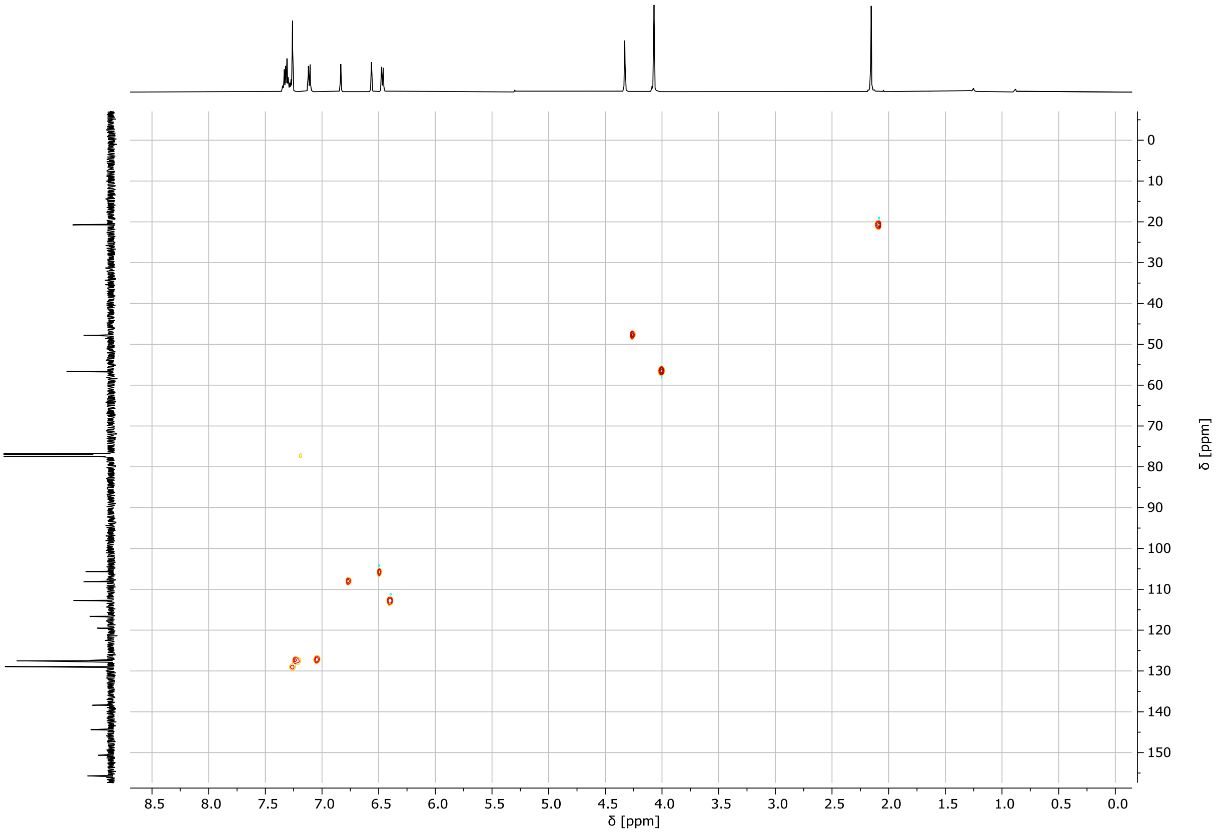


Figure S119: HSQC spectrum ($CDCl_3$) of 2,2'-bis(4-(benzylamino)phenyl)-5,5'-dimethoxy-7,7'-dimethyl-4H,4'H-[8,8'-bichromene]-4,4'-dione (**20**).

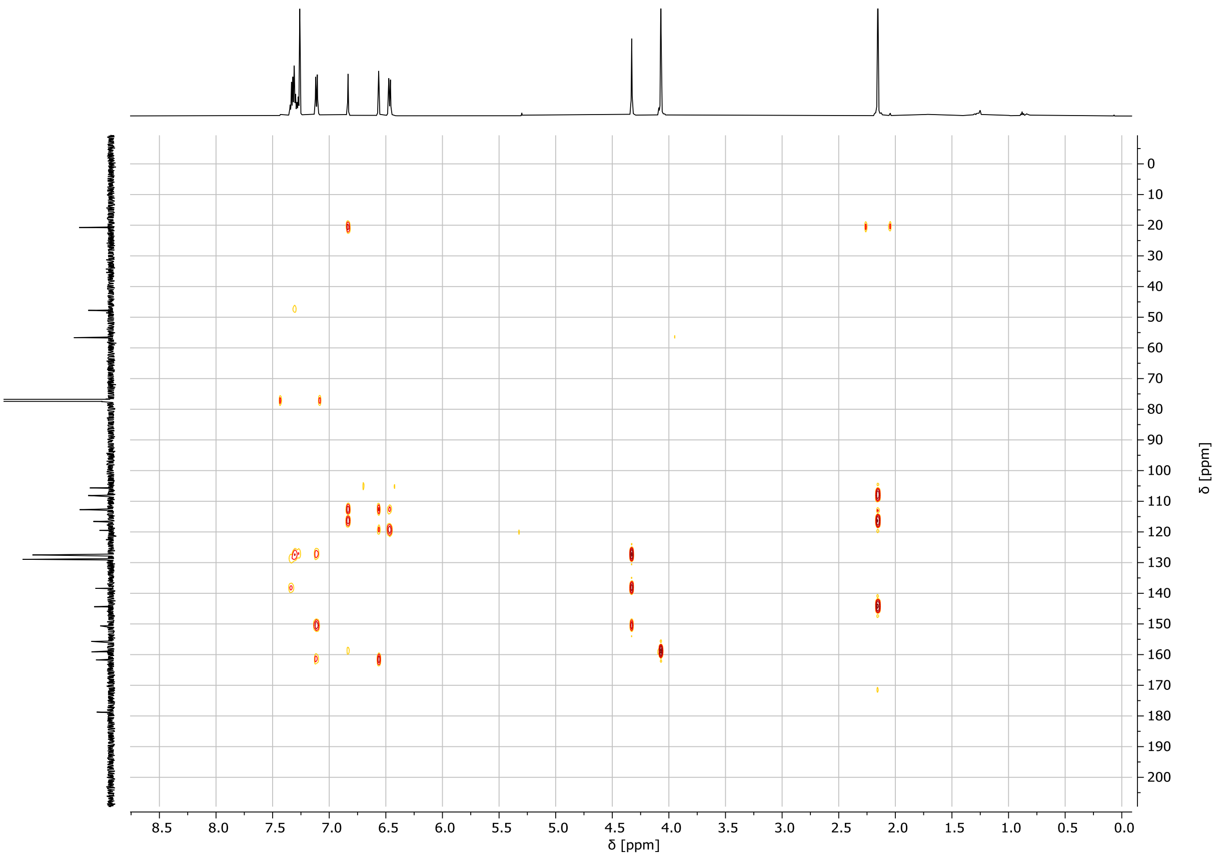


Figure S120: HMBC spectrum ($CDCl_3$) of 2,2'-bis(4-(benzylamino)phenyl)-5,5'-dimethoxy-7,7'-dimethyl-4H,4'H-[8,8'-bichromene]-4,4'-dione (**20**).

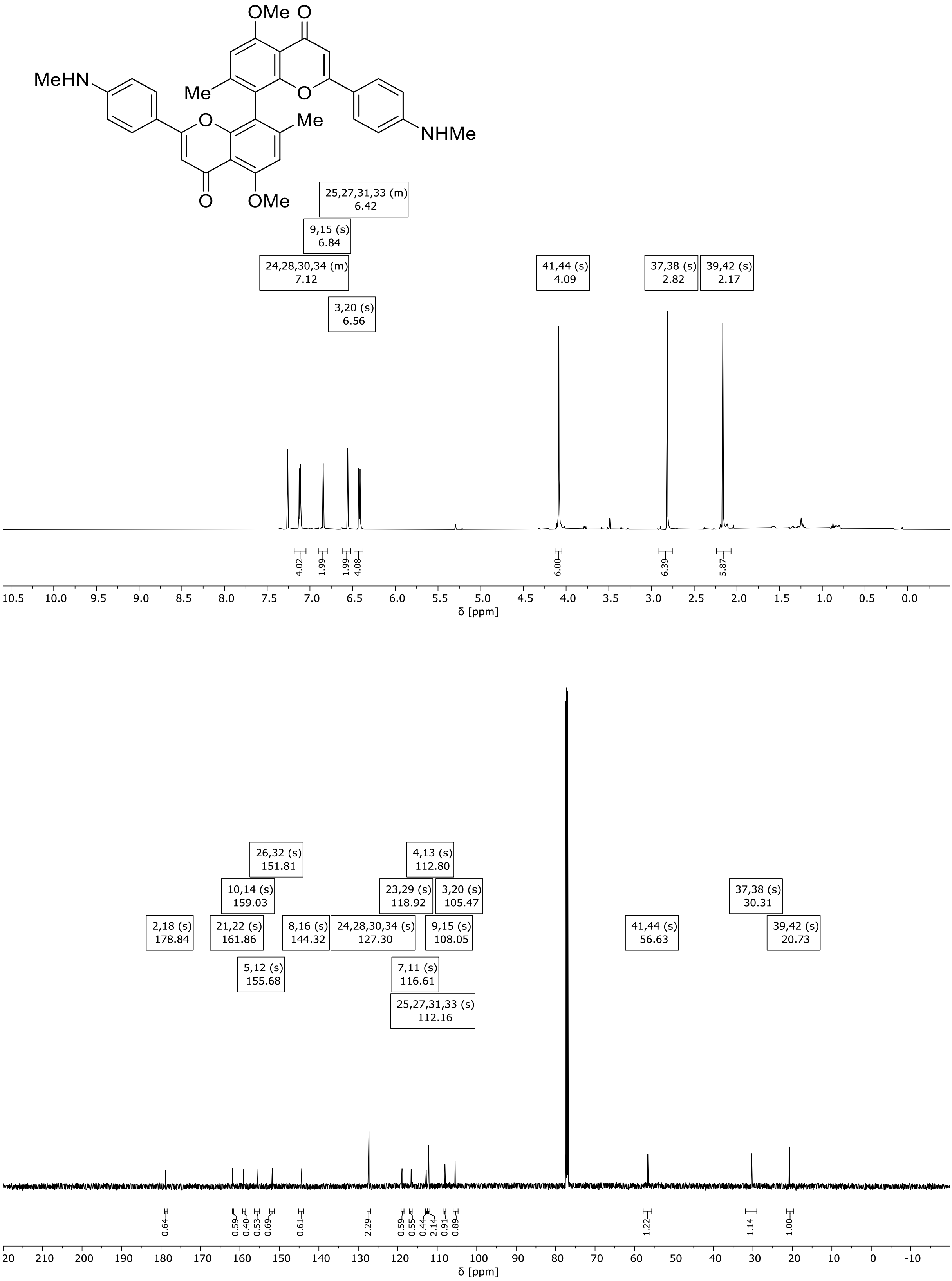


Figure S121: $^{1}H$- and $^{13}C$-NMR spectra (600 / 151 MHz, $CDCl_3$) of 5,5'-dimethoxy-7,7'-dimethyl-2,2'-bis(4-(methylamino)phenyl)-4H,4'H-[8,8'-bichromene]-4,4'-dione (**21**).

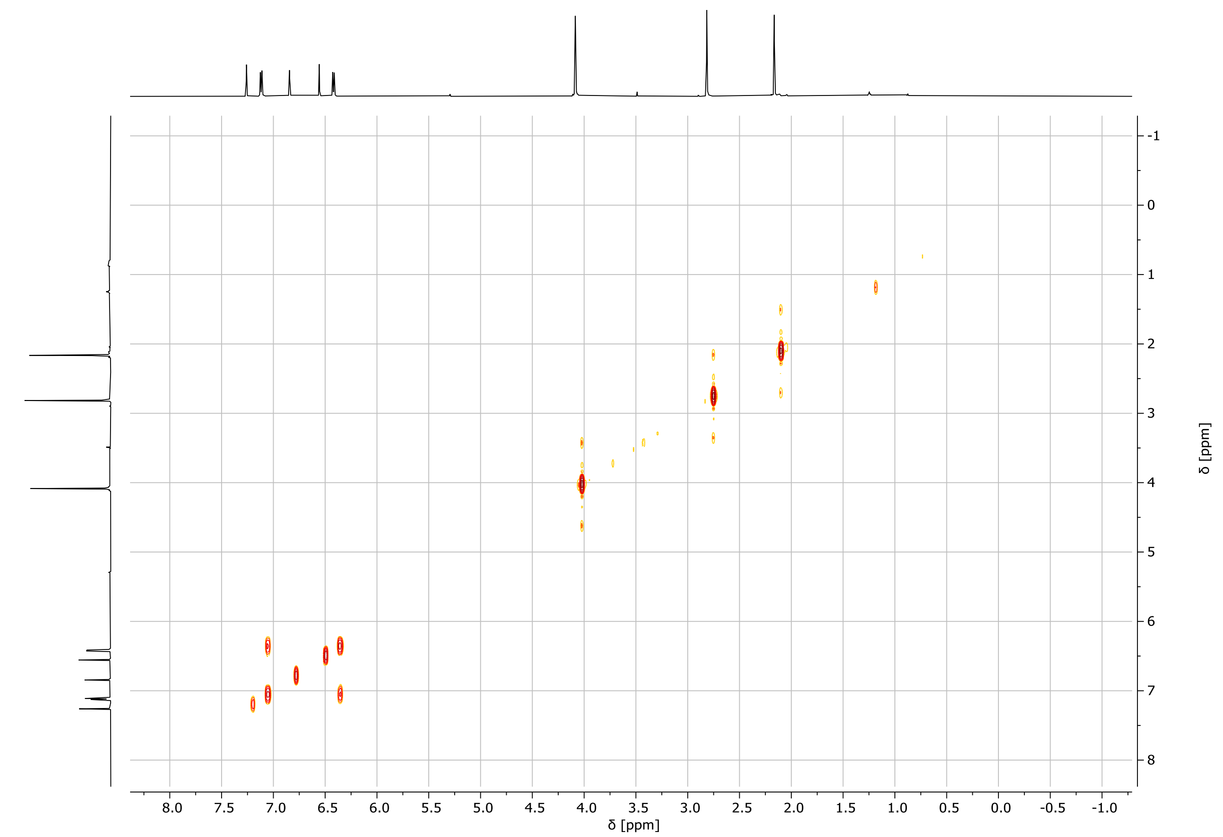


Figure S122: COSY spectrum ($CDCl_3$) of 5,5'-dimethoxy-7,7'-dimethyl-2,2'-bis(4-(methylamino)phenyl)-4H,4'H-[8,8'-bichromene]-4,4'-dione (**21**).

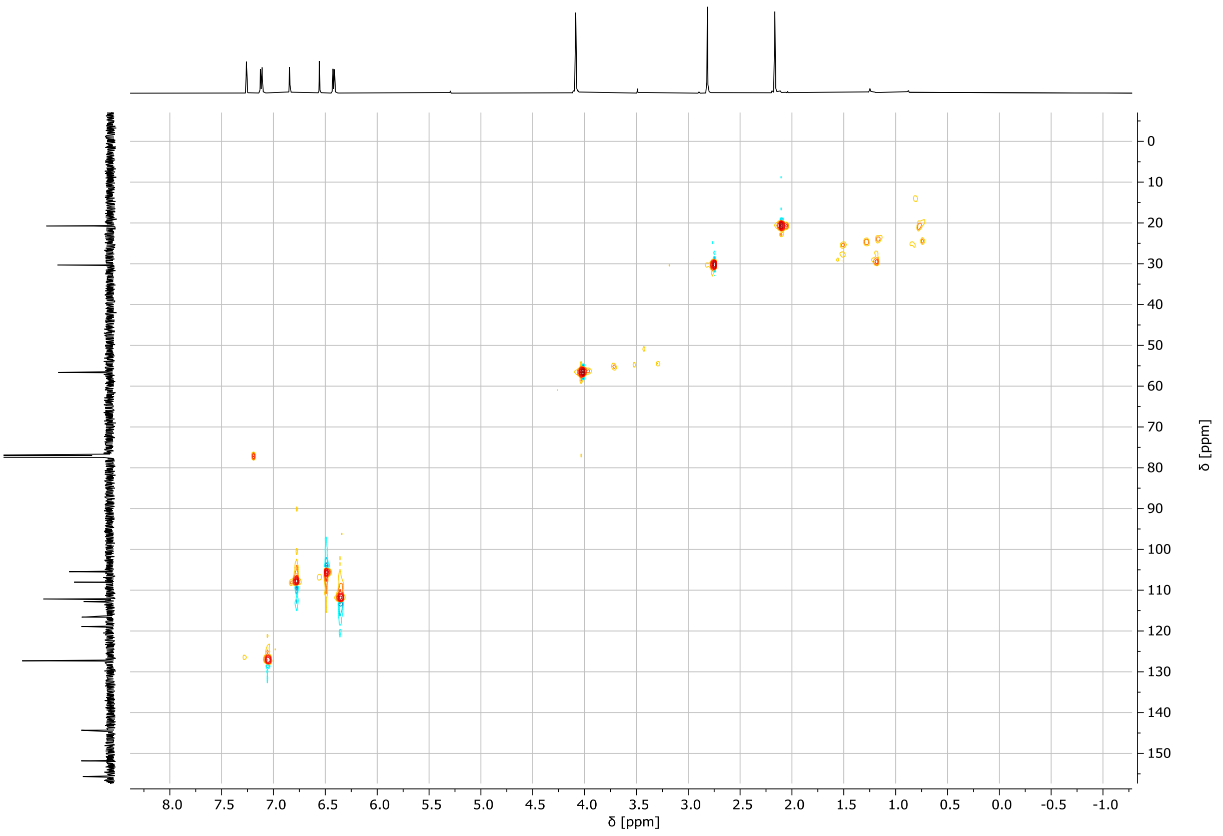


Figure S123: HSQC spectrum ($CDCl_3$) of 5,5'-dimethoxy-7,7'-dimethyl-2,2'-bis(4-(methylamino)phenyl)-4H,4'H-[8,8'-bichromene]-4,4'-dione (**21**).

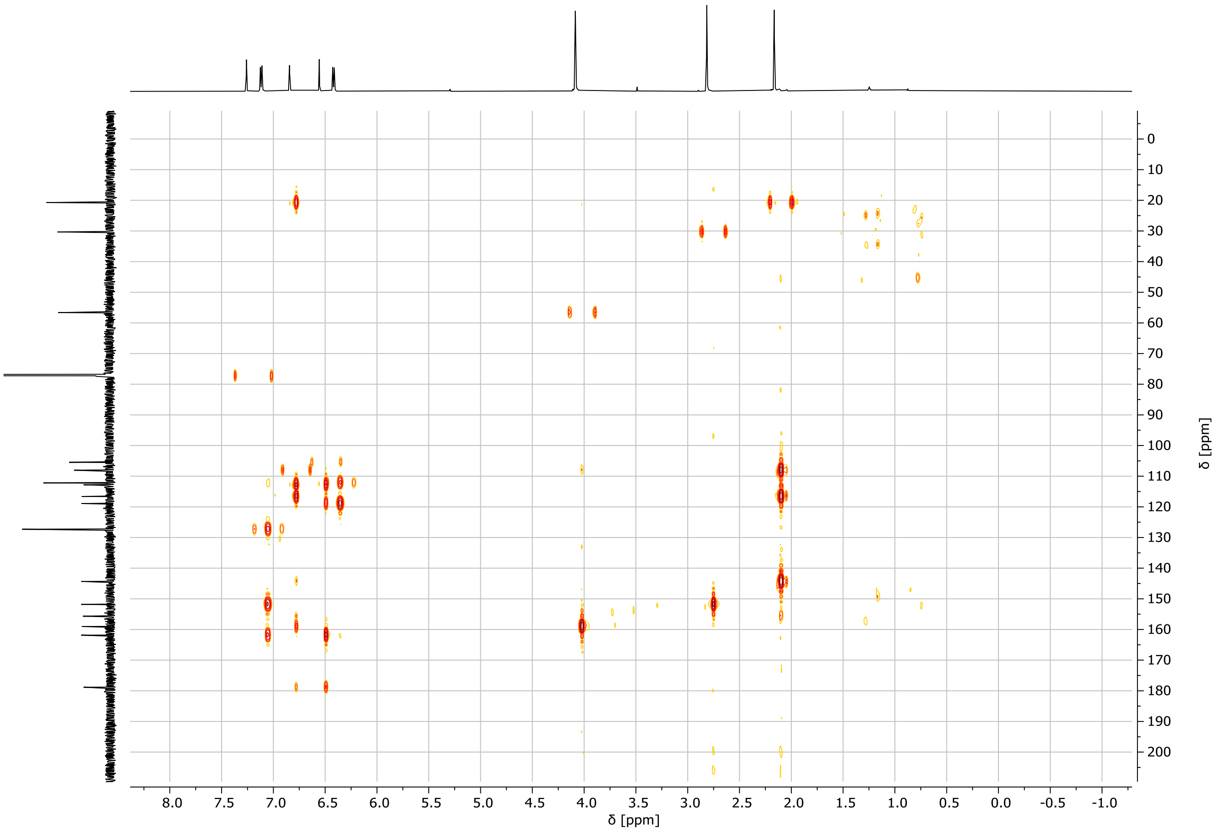


Figure S124: HMBC spectrum ($CDCl_3$) of 5,5'-dimethoxy-7,7'-dimethyl-2,2'-bis(4-(methylamino)phenyl)-4H,4'H-[8,8'-bichromene]-4,4'-dione (**21**).

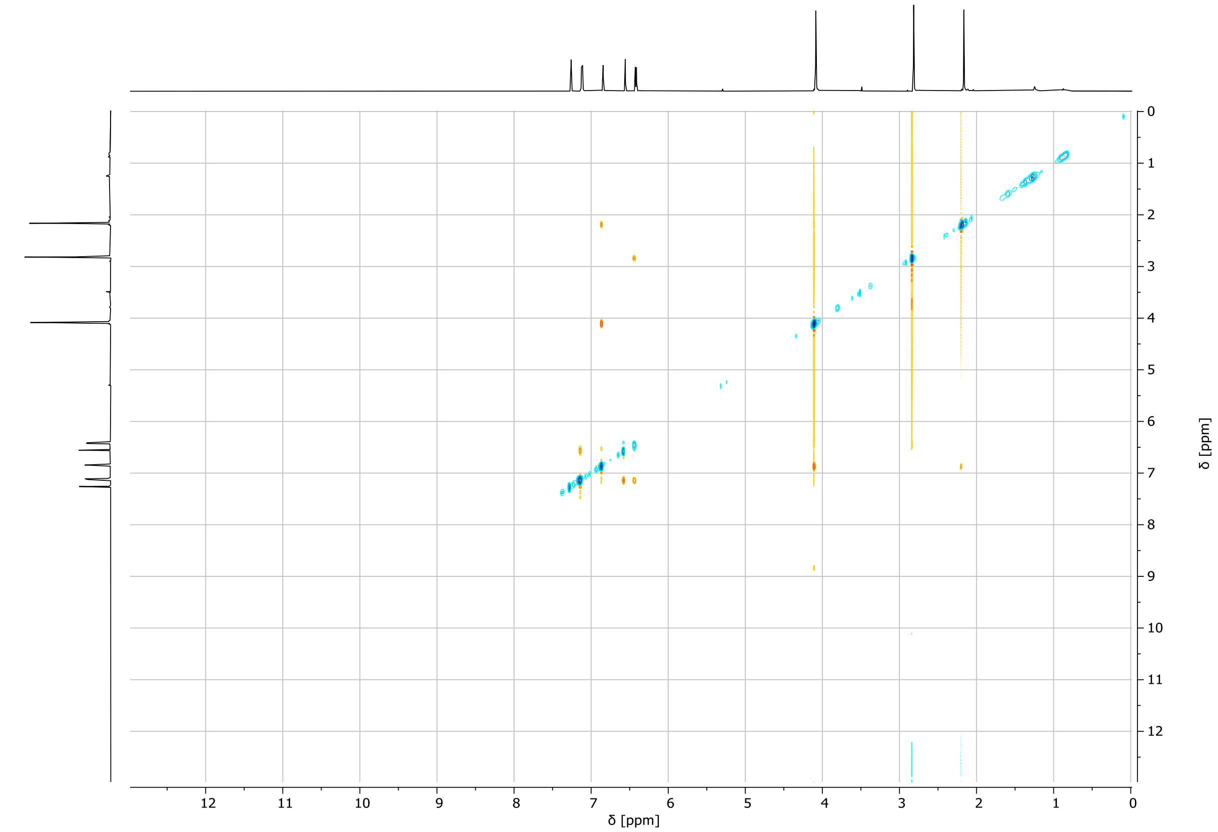


Figure S125: ROESY spectrum ($CDCl_3$) of 5,5'-dimethoxy-7,7'-dimethyl-2,2'-bis(4-(methylamino)phenyl)-4H,4'H-[8,8'-bichromene]-4,4'-dione (**21**).

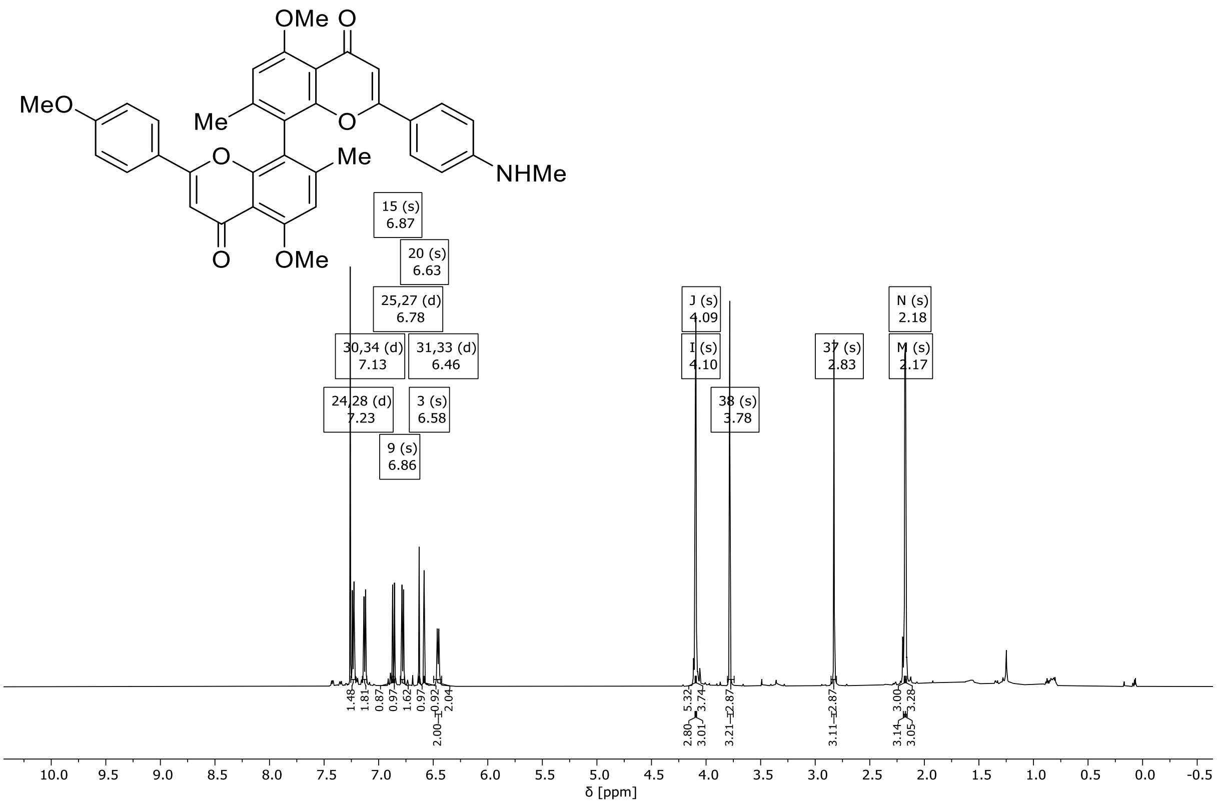


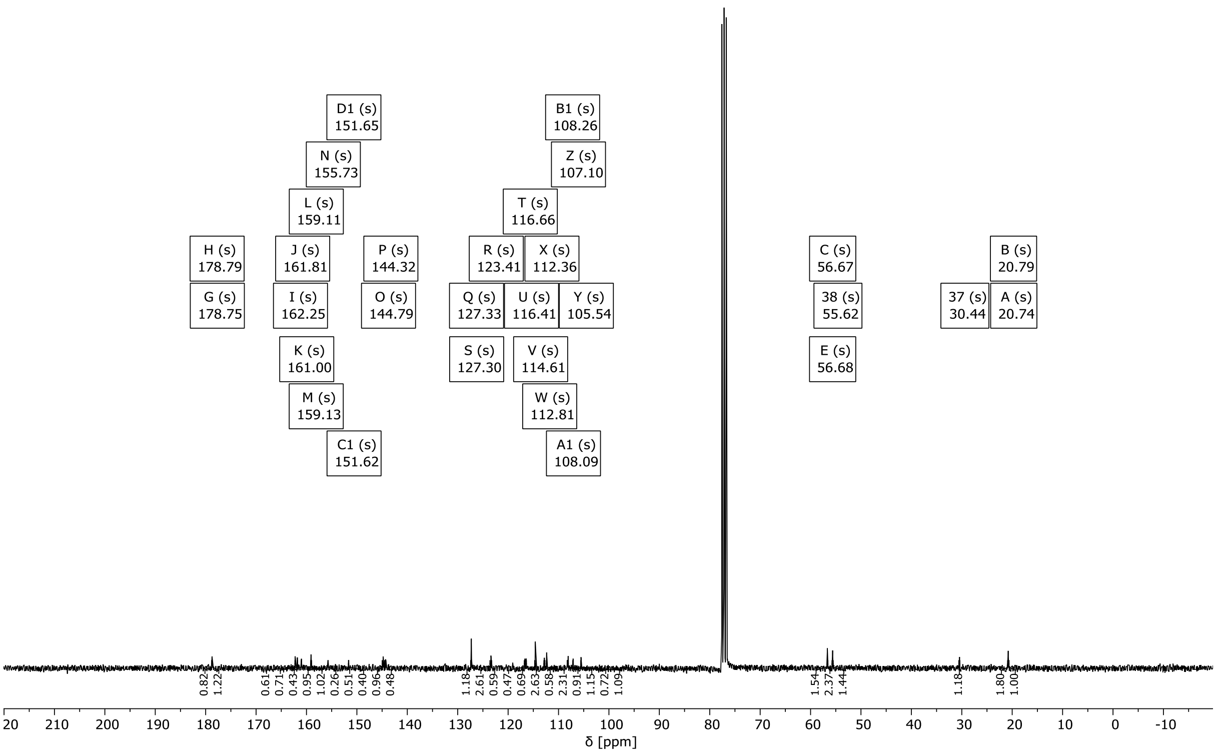


Figure S126: $^{1}$H- and $^{13}$C-NMR spectra (600 / 151 MHz, $CDCl_3$) of 5,5'-dimethoxy-2-(4-methoxyphenyl)-7,7'-dimethyl-2'-(4-(methylamino)phenyl)-4H,4'H-[8,8'-bichromene]-4,4'-dione (**22**).

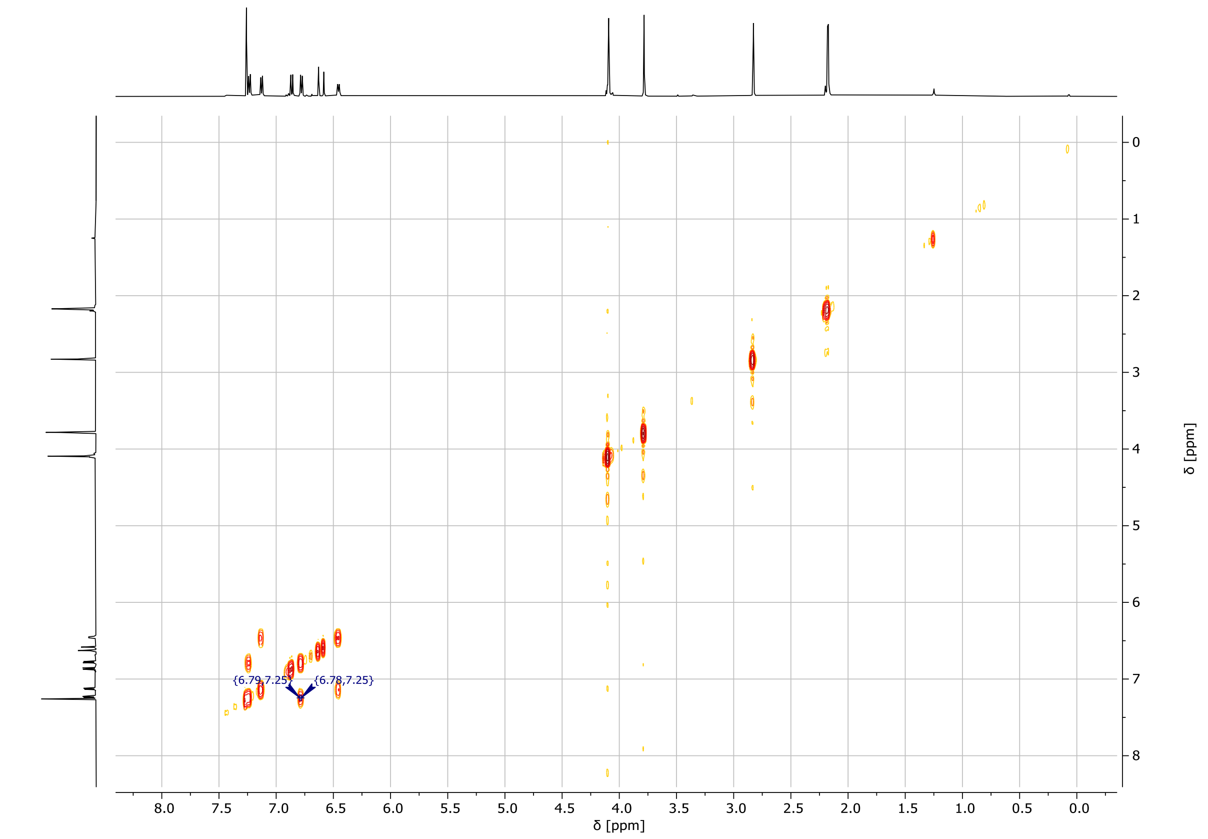


Figure S127: COSY spectrum ($CDCl_3$) of 5,5'-dimethoxy-2-(4-methoxyphenyl)-7,7'-dimethyl-2'-(4-(methylamino)phenyl)-4H,4'H-[8,8'-bichromene]-4,4'-dione (**22**).

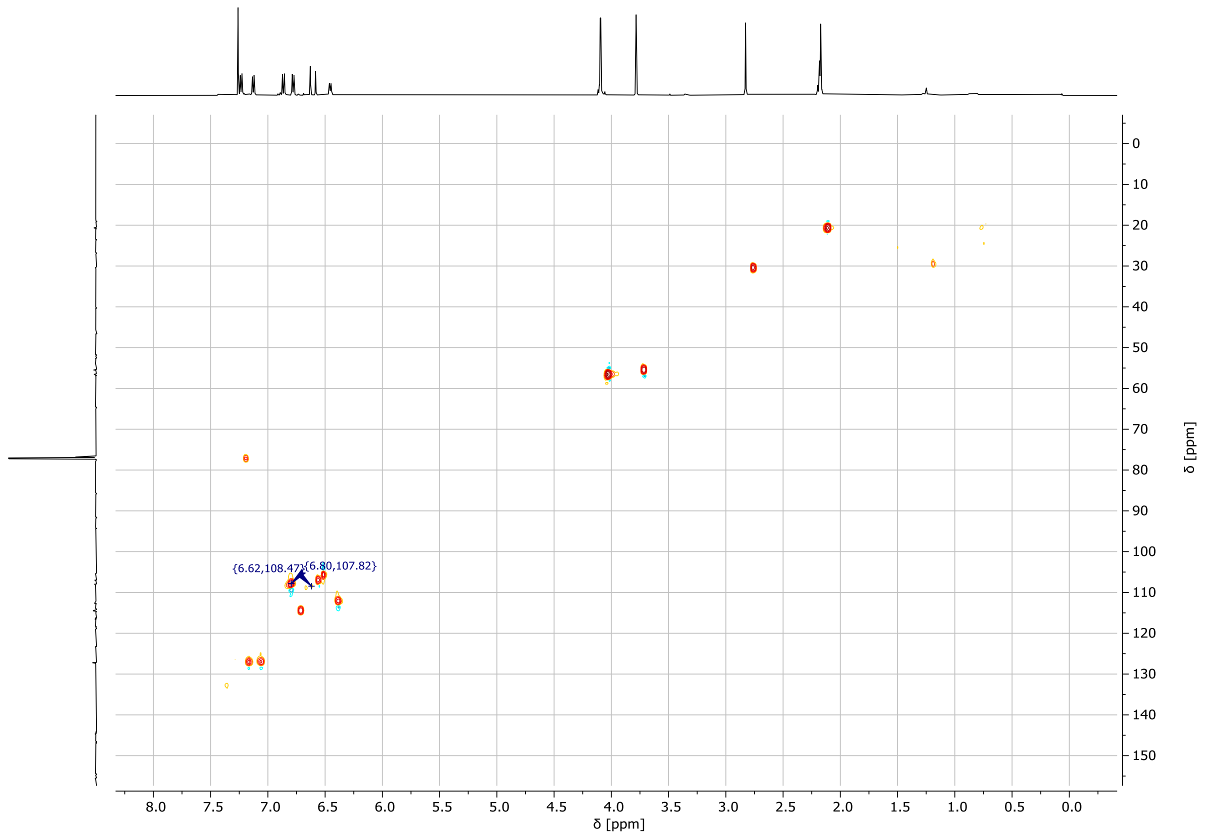


Figure S128: HSQC spectrum ($CDCl_3$) of 5,5'-dimethoxy-2-(4-methoxyphenyl)-7,7'-dimethyl-2'-(4-(methylamino)phenyl)-4H,4'H-[8,8'-bichromene]-4,4'-dione (**22**).

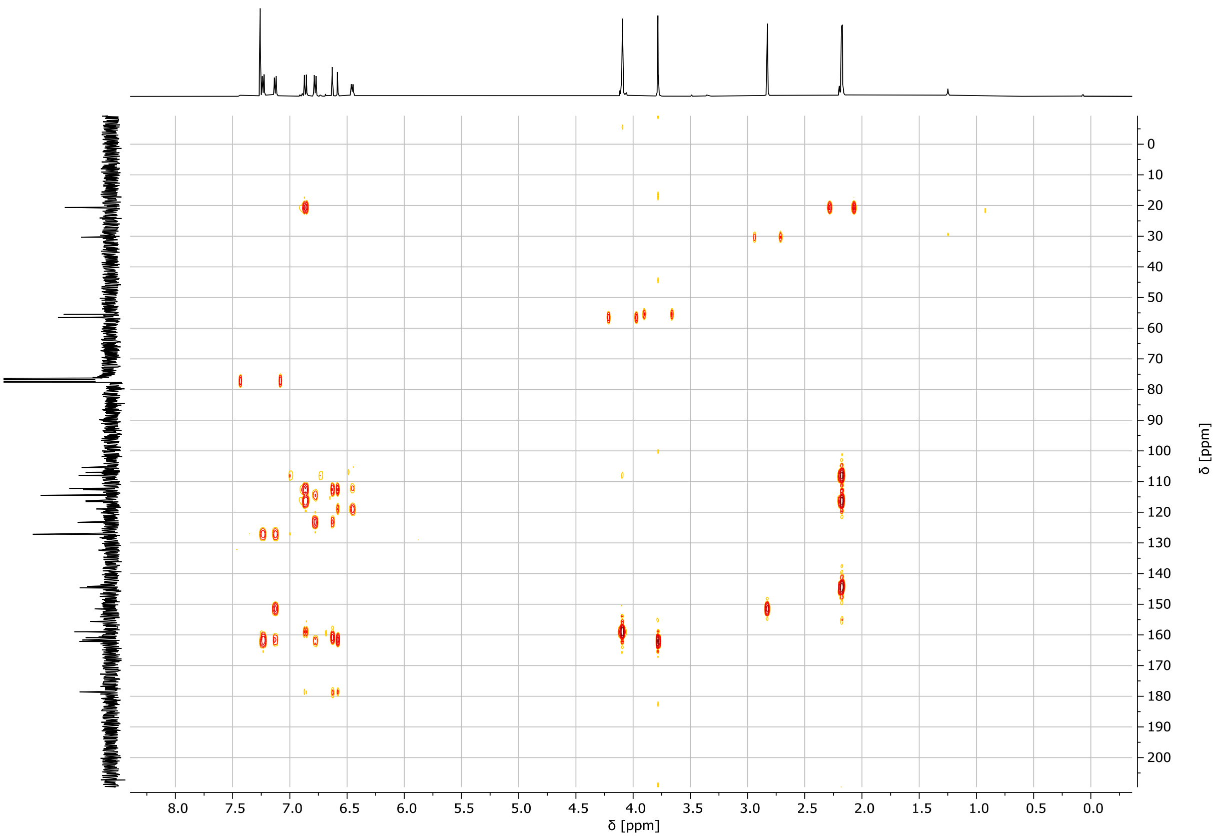


Figure S129: HMBC spectrum ($CDCl_3$) of 5,5'-dimethoxy-2-(4-methoxyphenyl)-7,7'-dimethyl-2'-(4-(methylamino)phenyl)-4H,4'H-[8,8'-bichromene]-4,4'-dione (**22**).

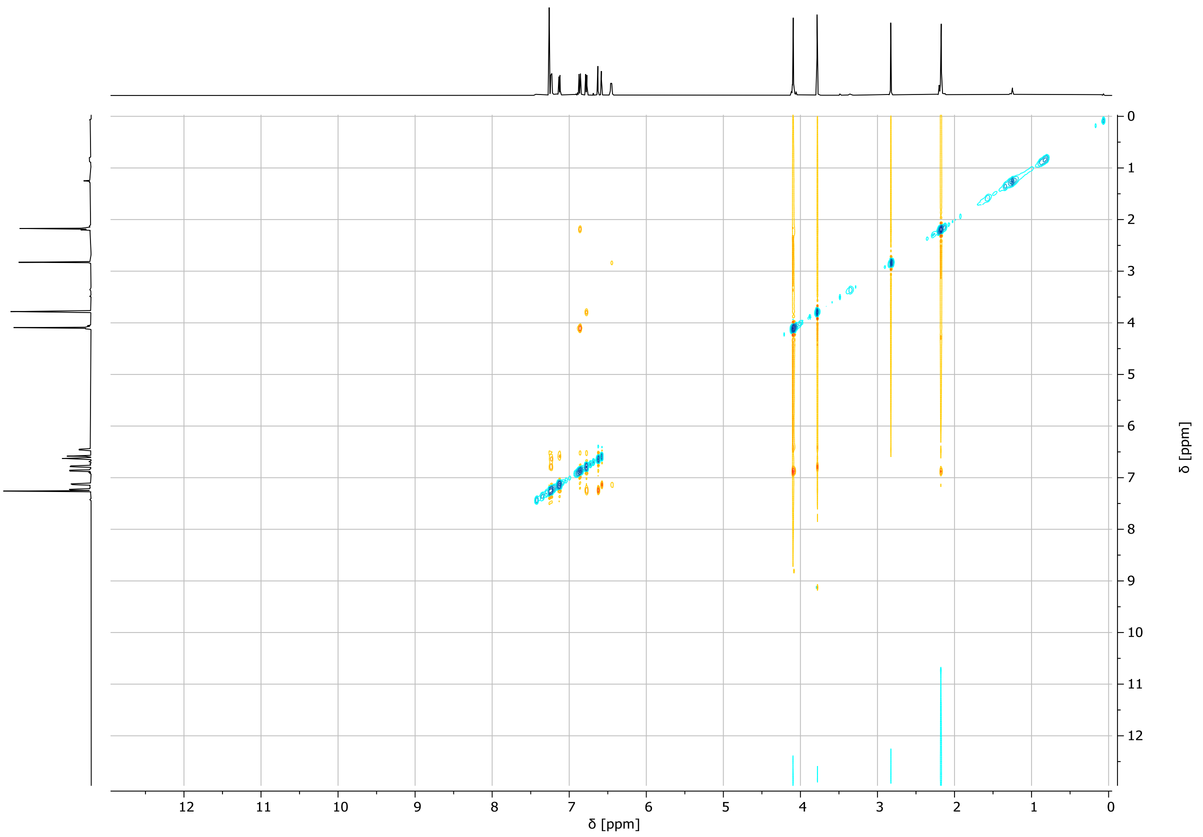


Figure S130: ROESY spectrum ($CDCl_3$) of 5,5'-dimethoxy-2-(4-methoxyphenyl)-7,7'-dimethyl-2'-(4-(methylamino)phenyl)-4H,4'H-[8,8'-bichromene]-4,4'-dione (**22**).

## Miscellaneous

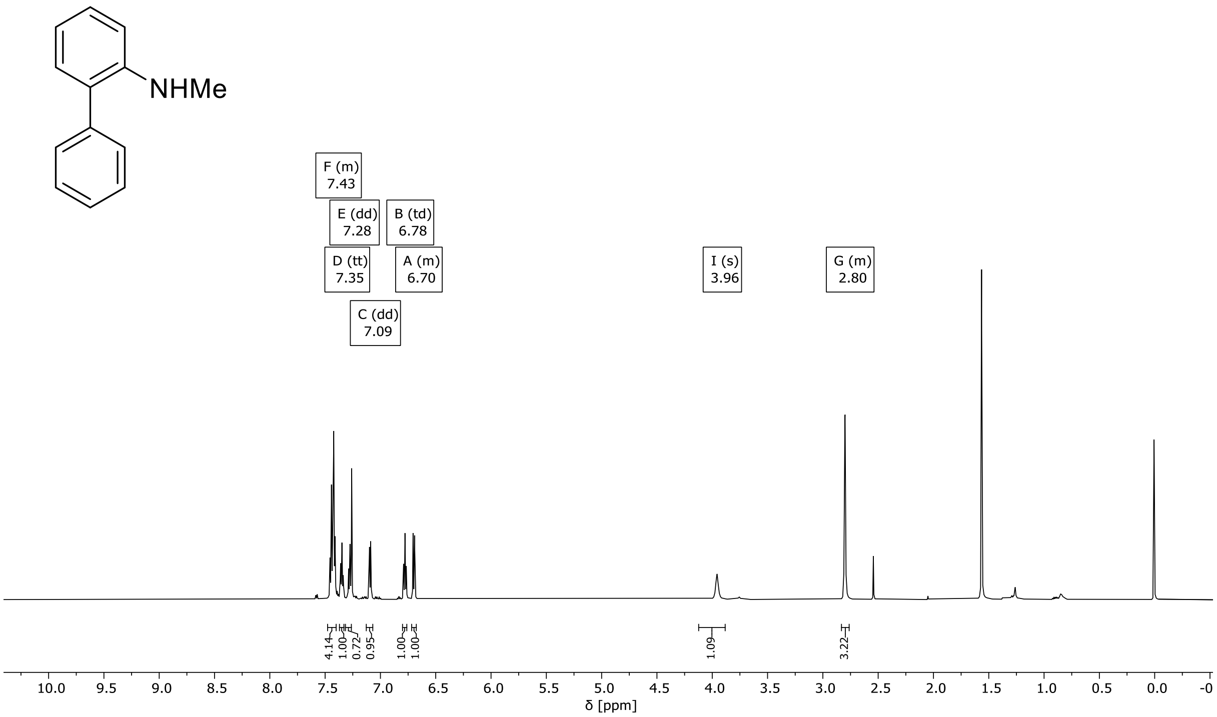


Figure S131: $^1$H-NMR spectrum (400 MHz, $CDCl_3$) of N-methyl-[1,1'-biphenyl]-2-amine (**25**).

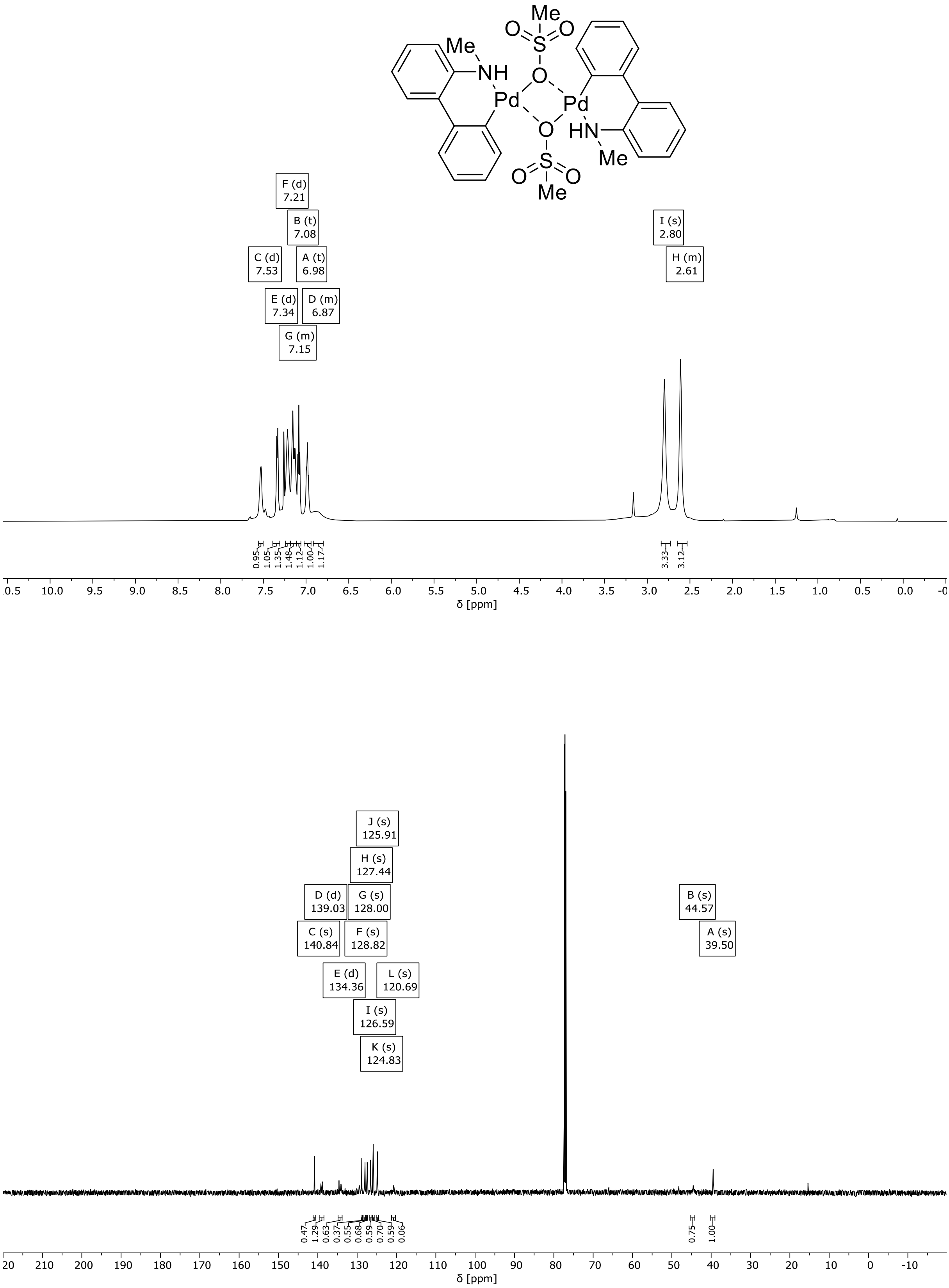


Figure S132: $^{1}H$- and $^{13}C$-NMR spectra (600 / 151 MHz, $CDCl_3$) of Pd complex **26**.

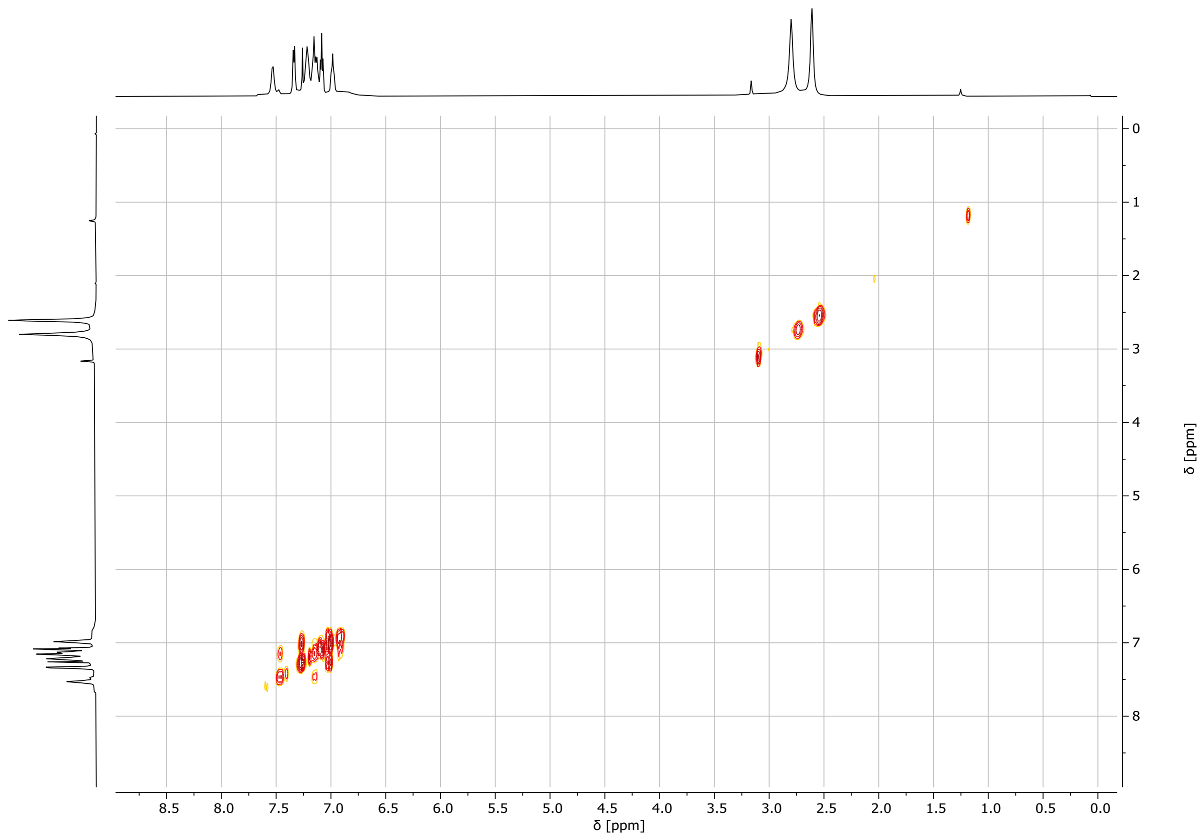


Figure S133: COSY spectrum ($CDCl_3$) of Pd complex **26**.

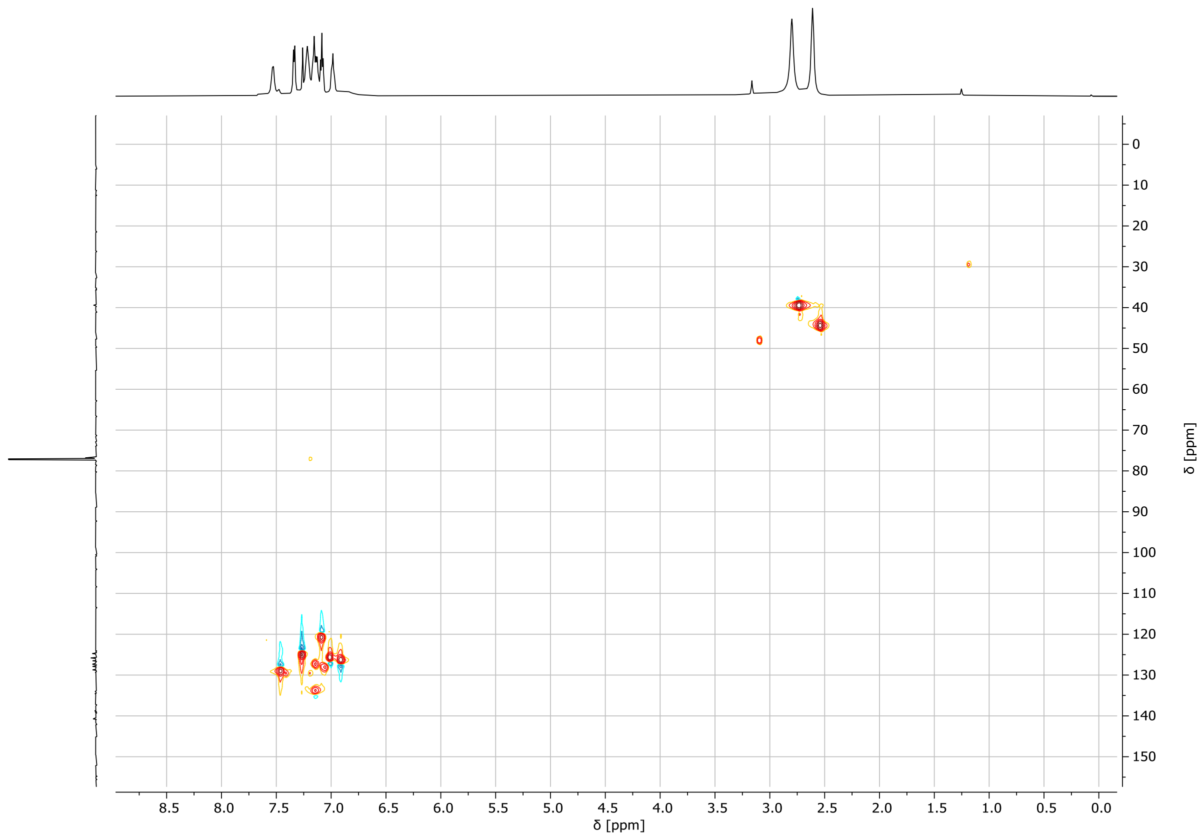


Figure S134: HSQC spectrum ($CDCl_3$) of Pd complex **26**.

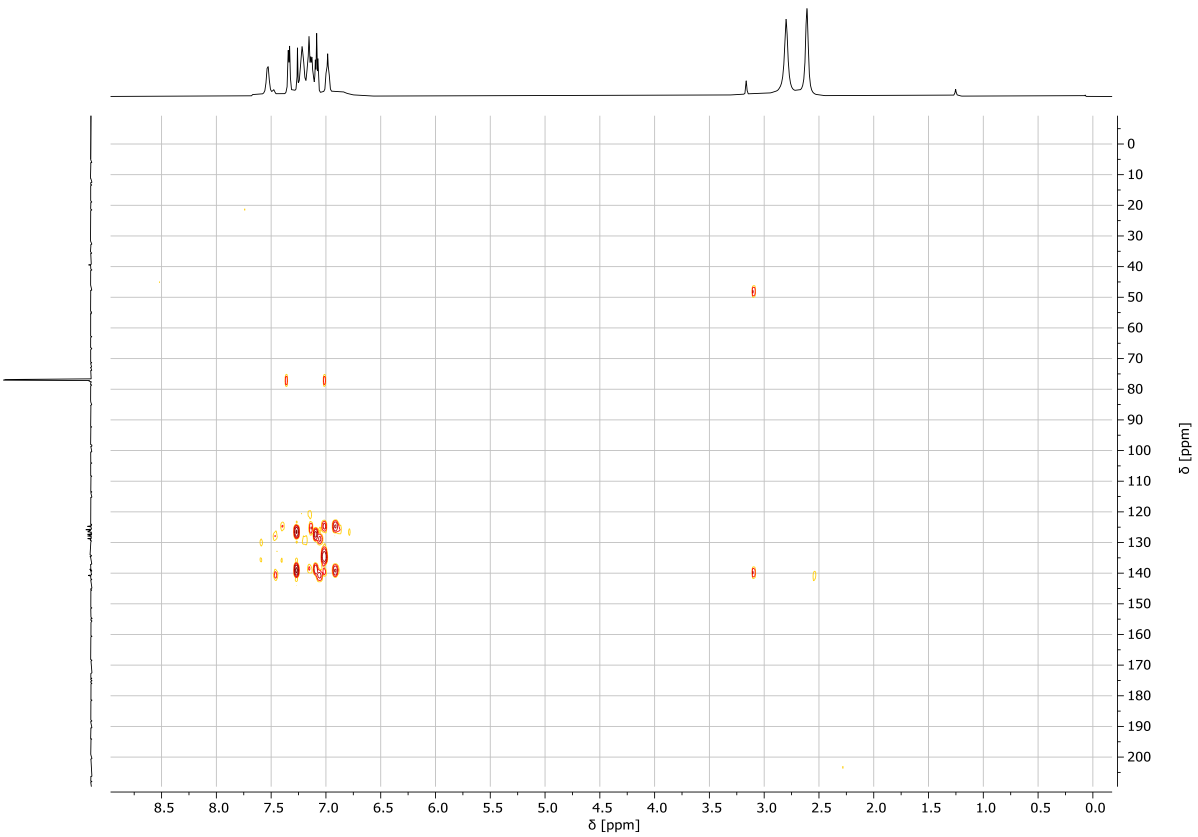


Figure S135: HMBC spectrum ($CDCl_3$) of Pd complex **26**.

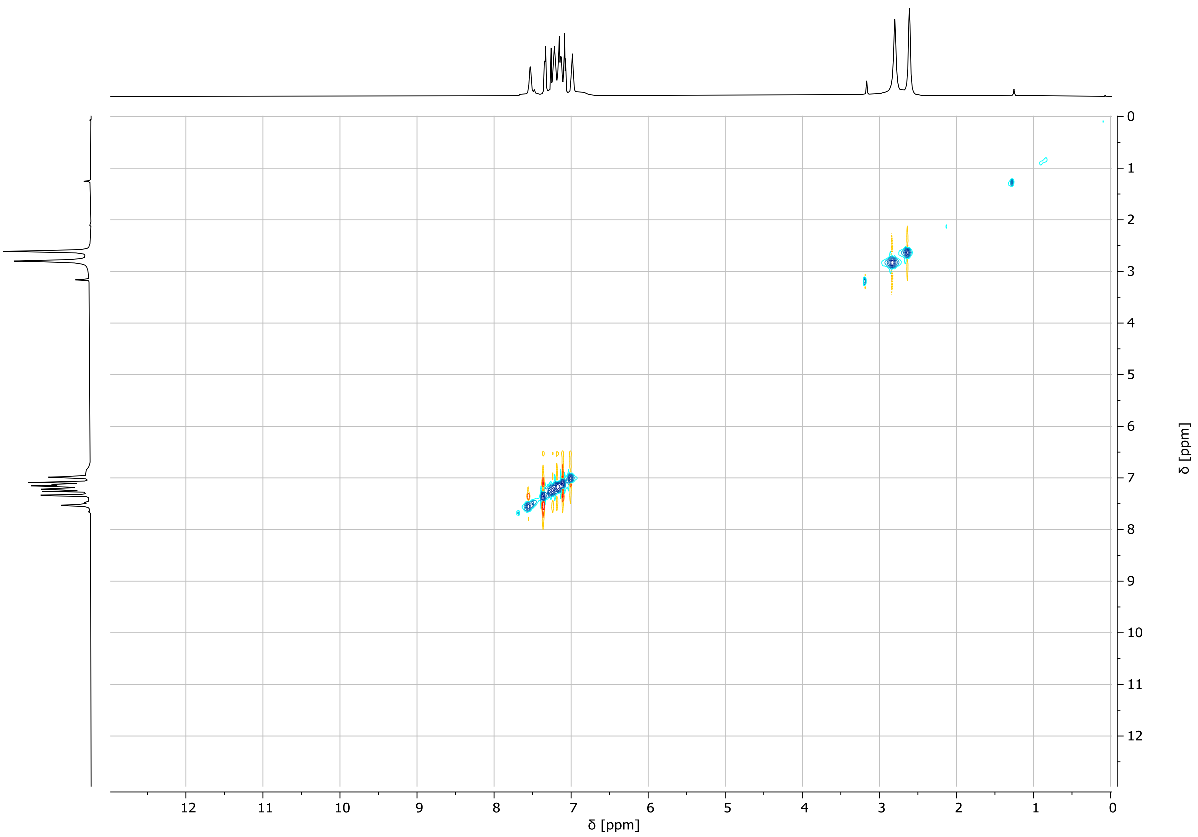


Figure S136: ROESY spectrum ($CDCl_3$) of Pd complex **26**.

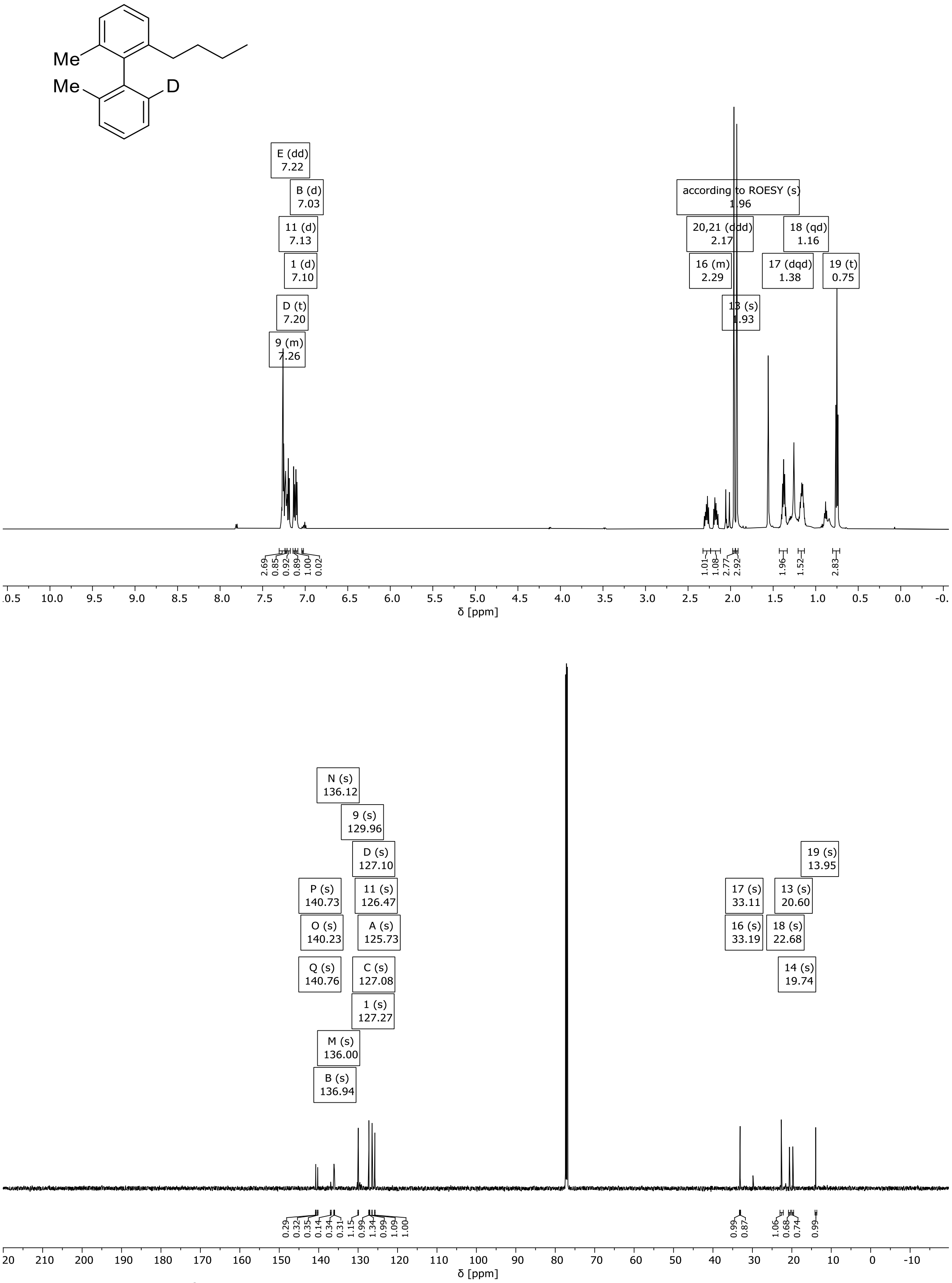


Figure S137: $^{1}H$- and $^{13}C$-NMR spectra (600 / 151 MHz, $CDCl_3$) of 2-butyl-2',6-dimethyl-1,1'-biphenyl-6'-d (**28**).

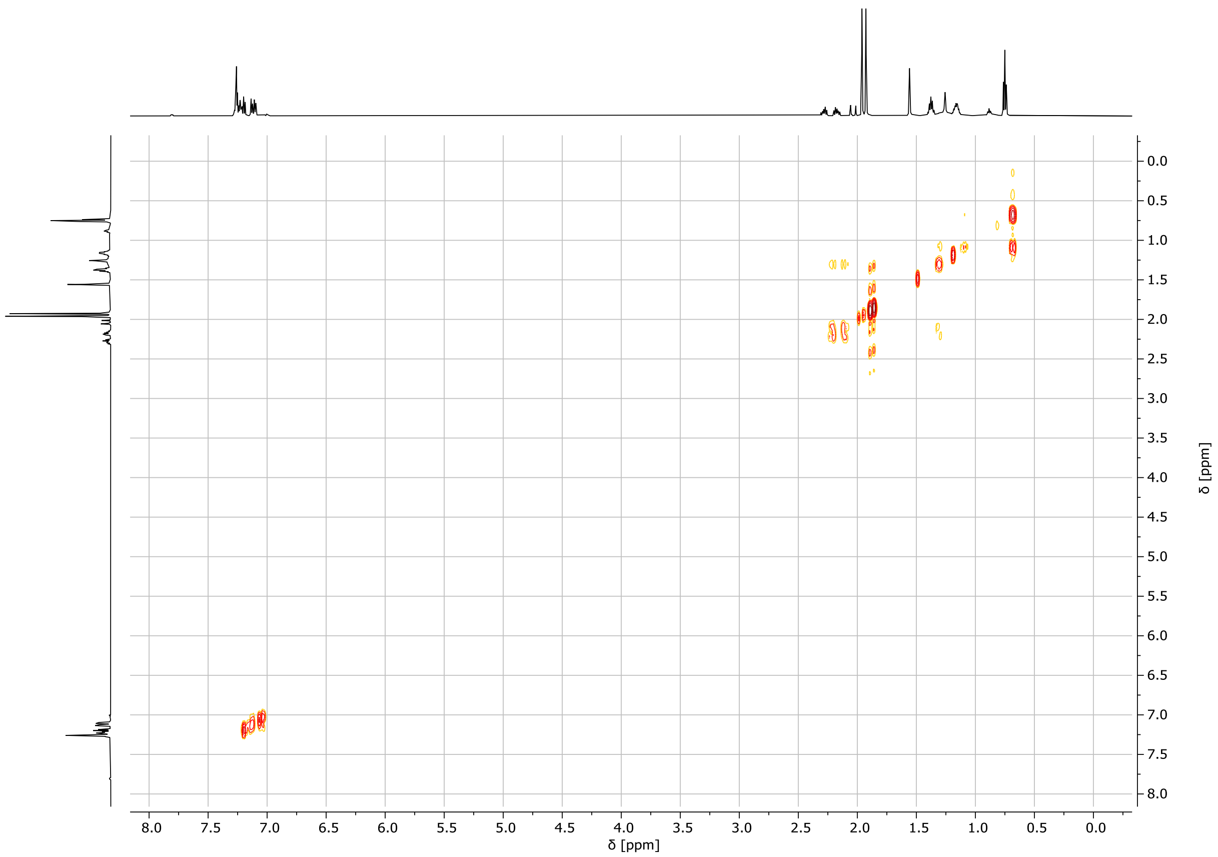

Figure S138: COSY spectrum ($CDCl_3$) of 2-butyl-2',6-dimethyl-1,1'-biphenyl-6'-d (**28**).

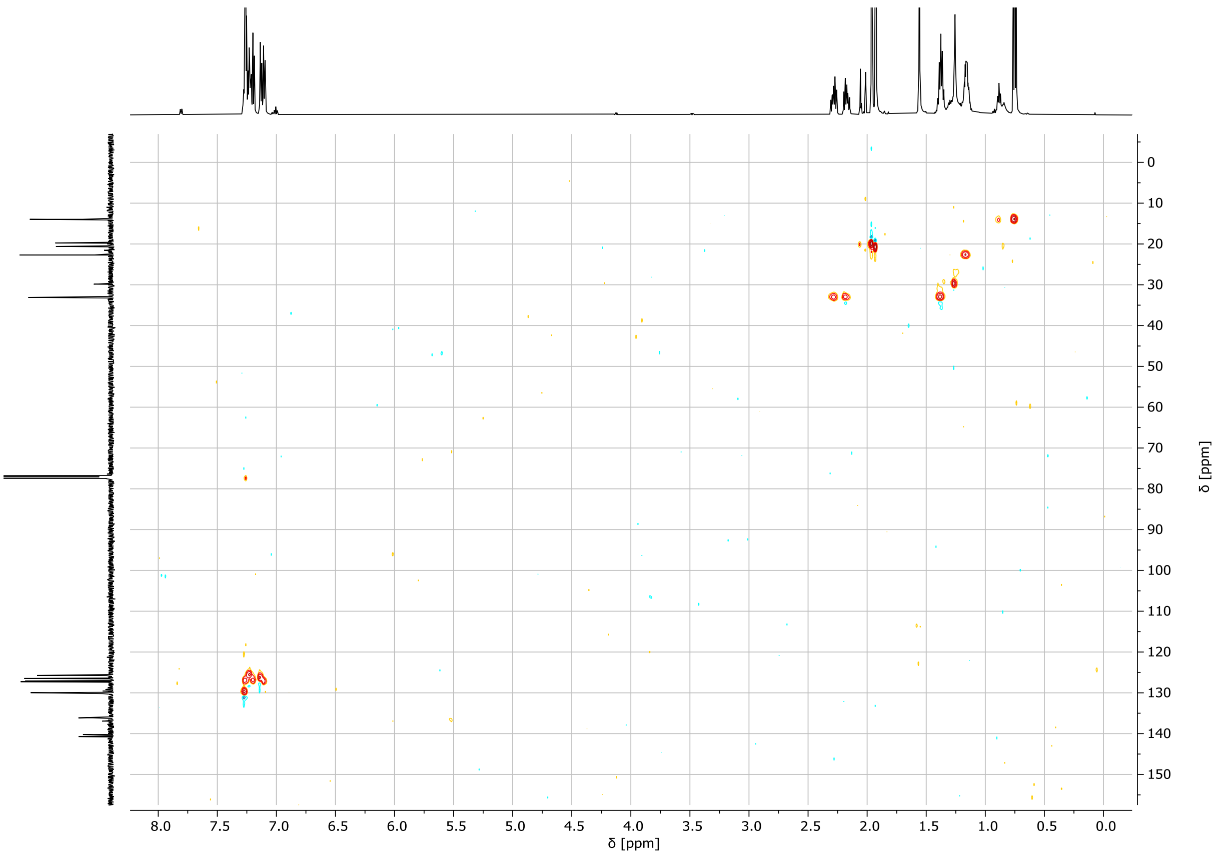

Figure S139: HSQC spectrum ($CDCl_3$) of 2-butyl-2',6-dimethyl-1,1'-biphenyl-6'-d (**28**).

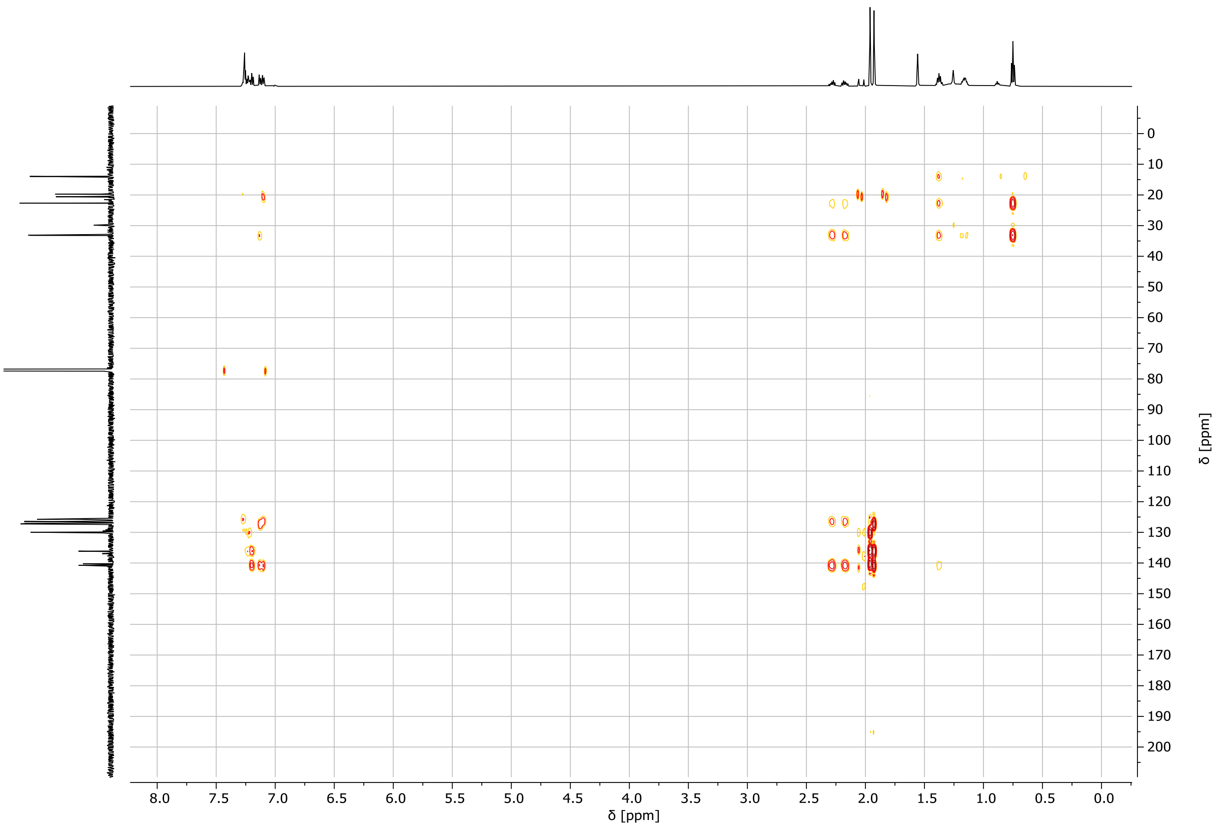

Figure S140: HMBC spectrum ($CDCl_3$) of 2-butyl-2',6-dimethyl-1,1'-biphenyl-6'-d (**28**).

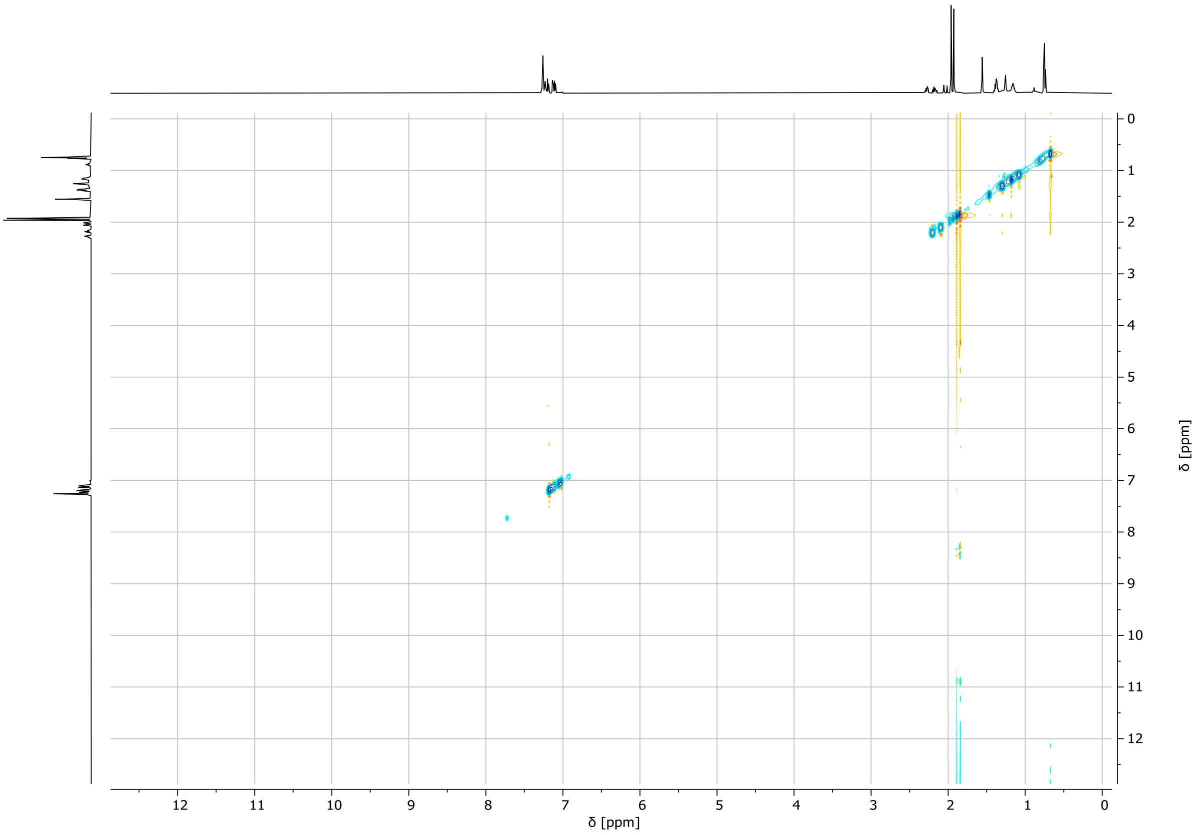

Figure S141: ROESY spectrum ($CDCl_3$) of 2-butyl-2',6-dimethyl-1,1'-biphenyl-6'-d (**28**).

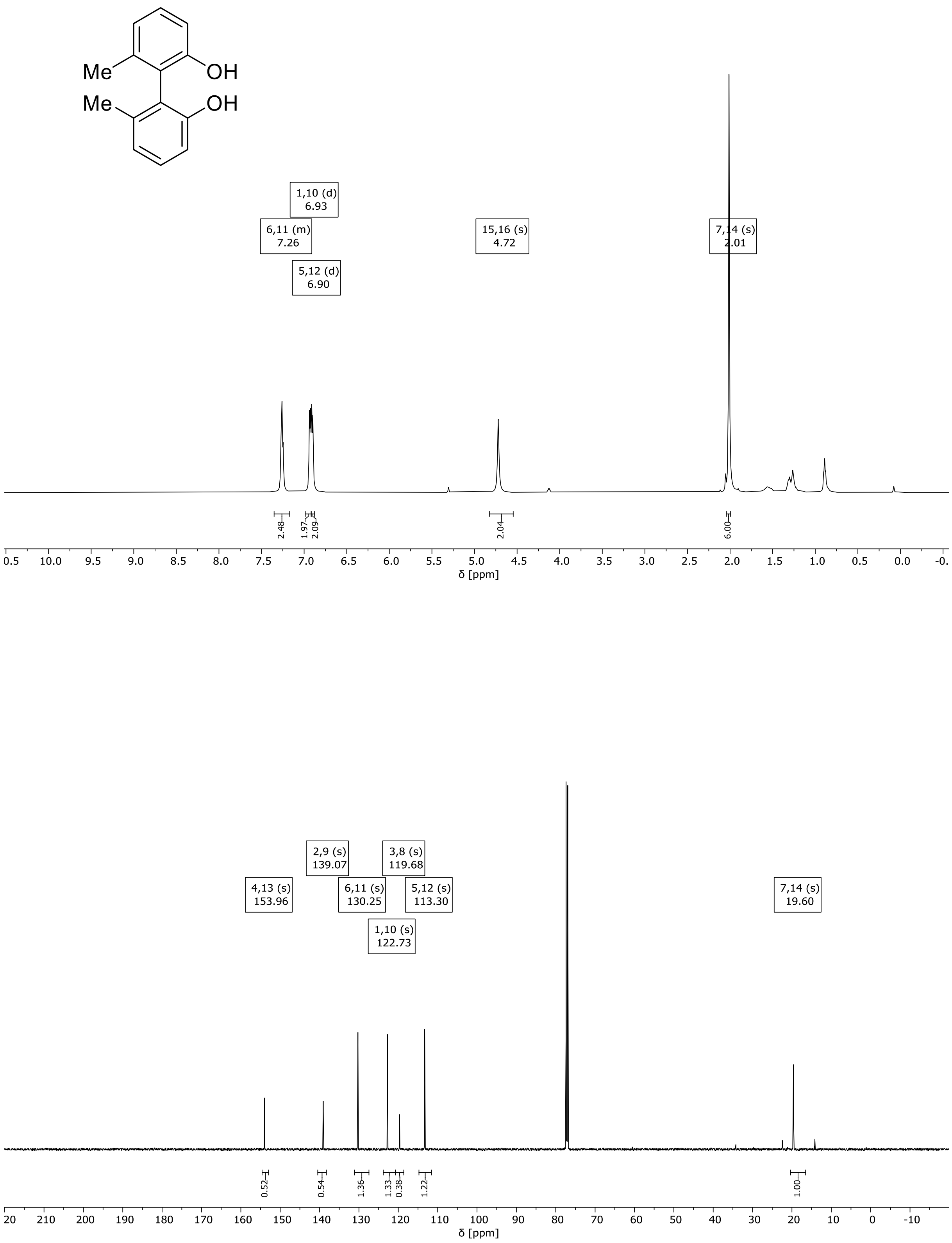


Figure S142: $^{1}$H- and $^{13}$C-NMR spectra (600 / 151 MHz, $CDCl_3$) of 6,6'-dimethyl-[1,1'-biphenyl]-2,2'-diol (**27**).

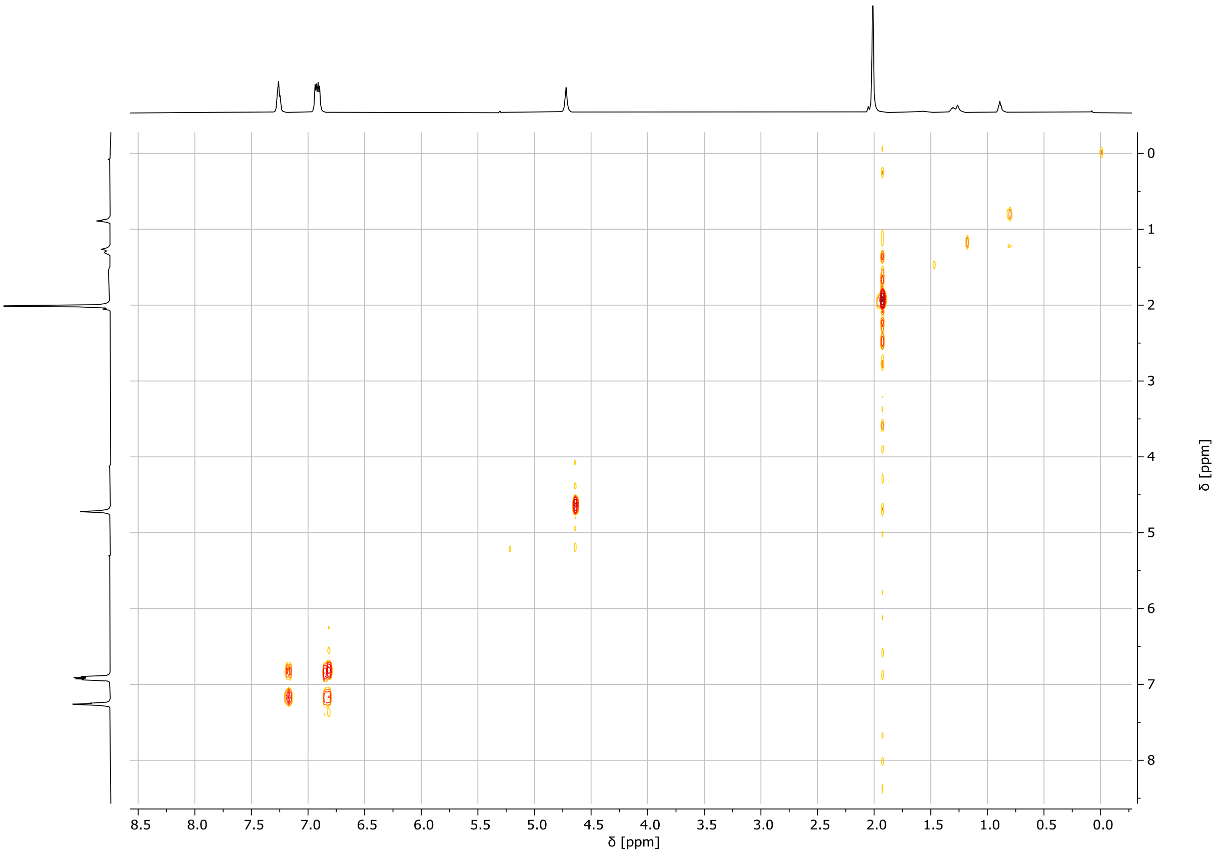


Figure S143: COSY spectrum ($CDCl_3$) of 6,6'-dimethyl-[1,1'-biphenyl]-2,2'-diol (**27**).

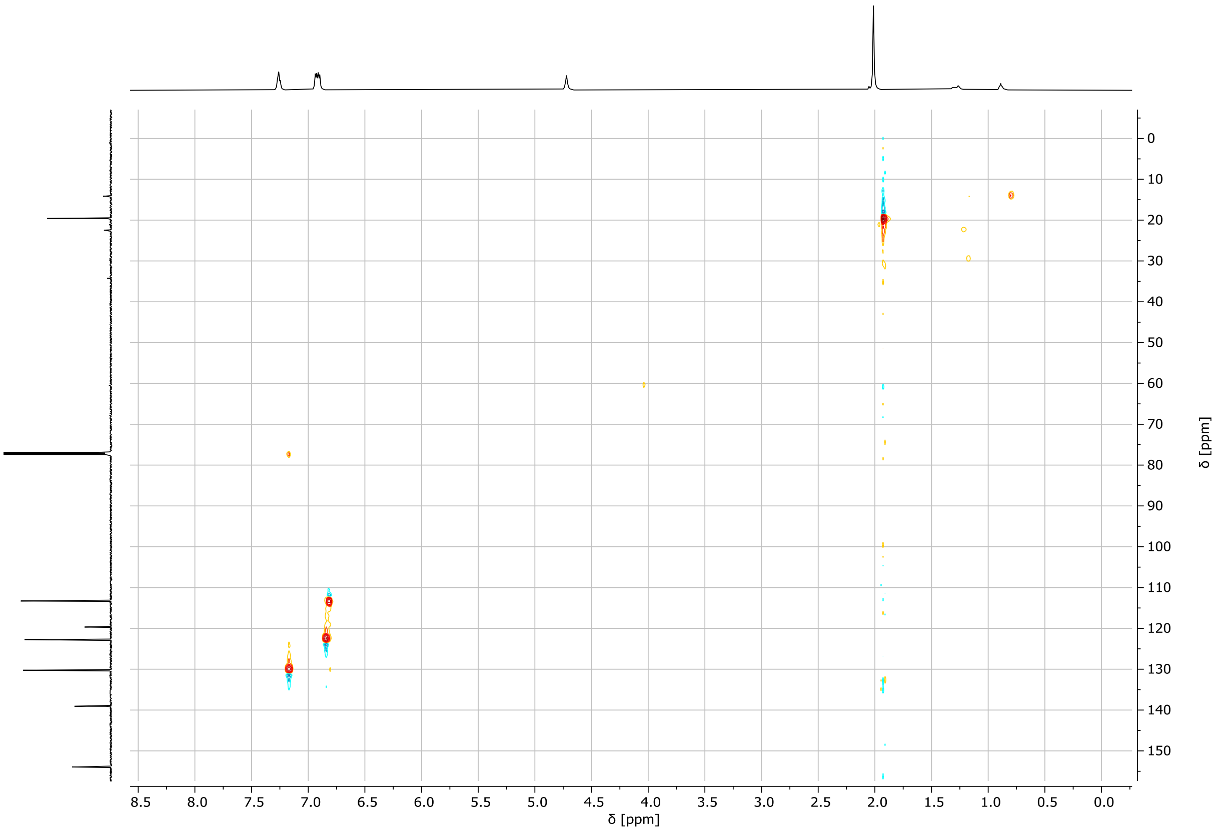


Figure S144: HSQC spectrum ($CDCl_3$) of 6,6'-dimethyl-[1,1'-biphenyl]-2,2'-diol (**27**).

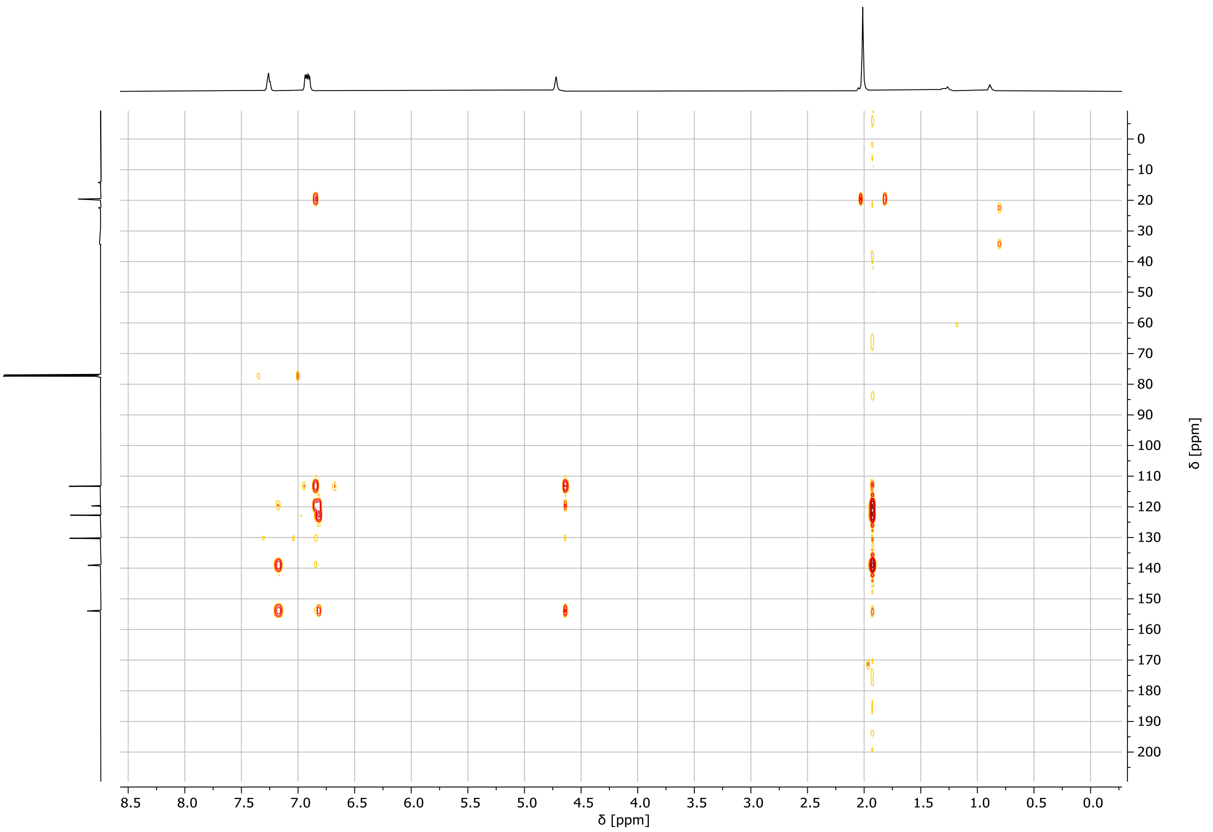


Figure S145: HMBC spectrum ($CDCl_3$) of 6,6'-dimethyl-[1,1'-biphenyl]-2,2'-diol (**27**).

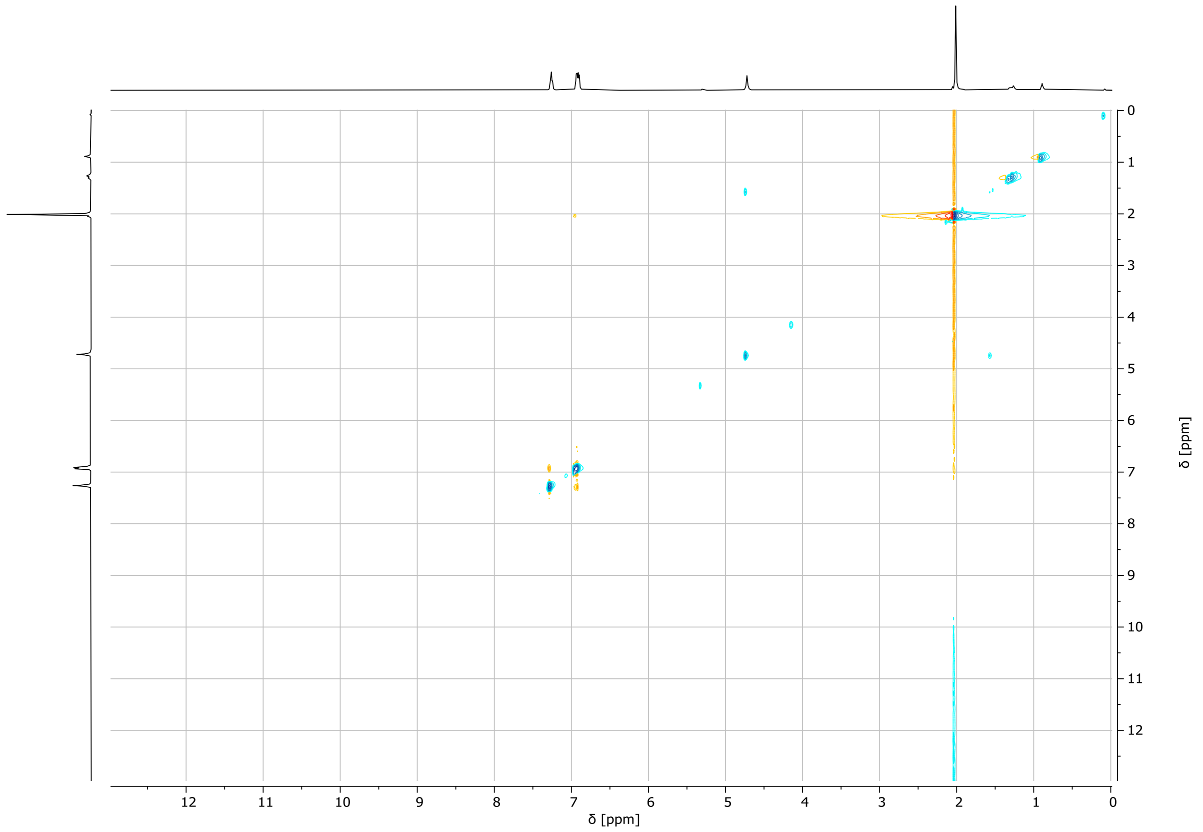


Figure S146: ROESY spectrum ($CDCl_3$) of 6,6'-dimethyl-[1,1'-biphenyl]-2,2'-diol (**27**).

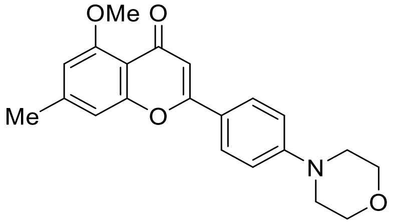


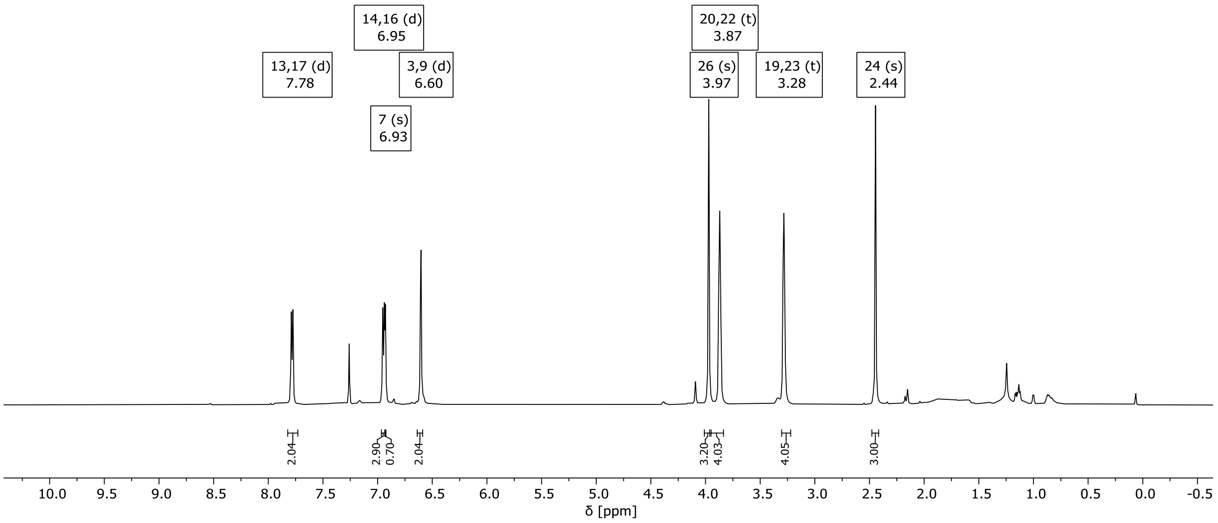


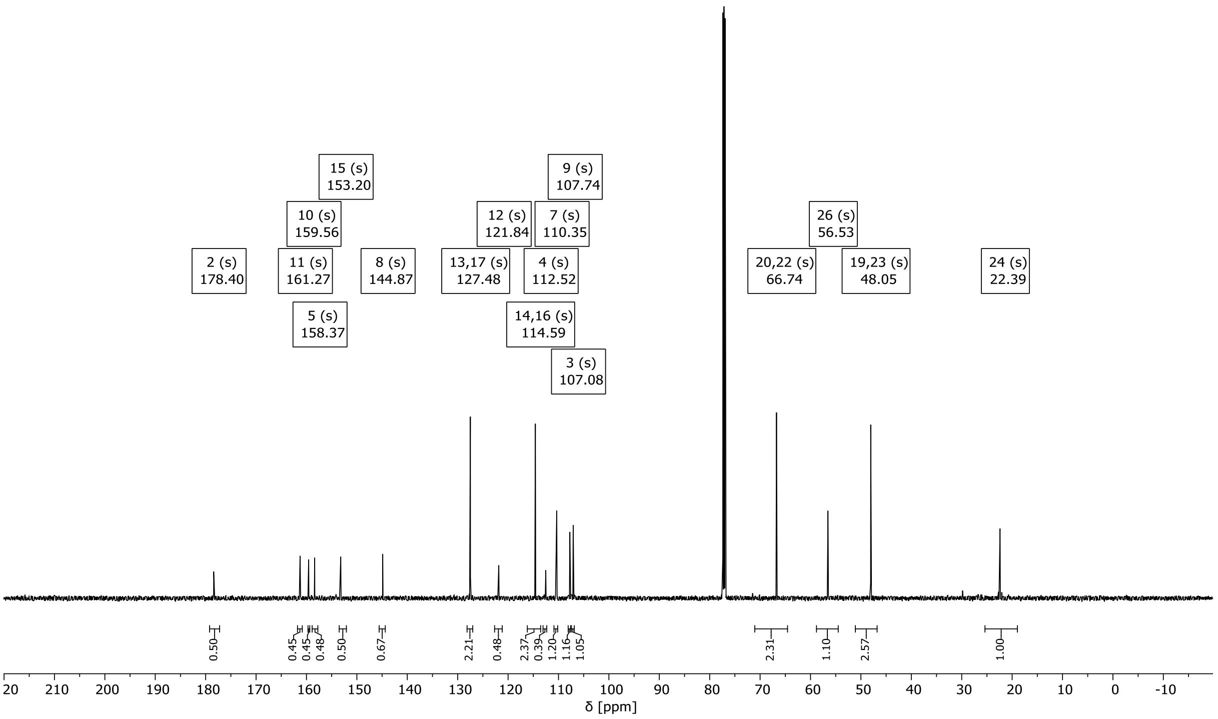


Figure S147: $^{1}H$- and $^{13}C$-NMR spectra (600 / 151 MHz, $CDCl_3$) of 5-methoxy-7-methyl-2-(4-morpholinophenyl)-4H-chromen-4-one (**29**).

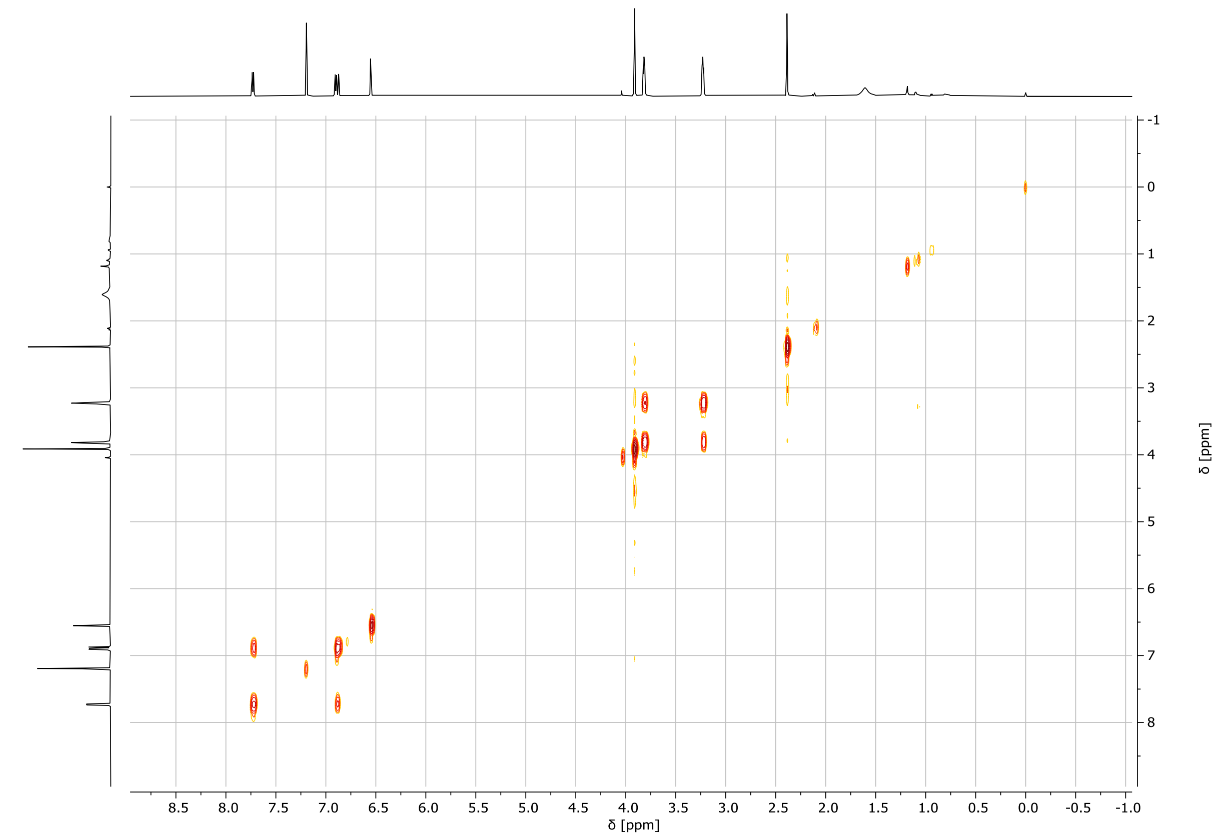


Figure S148: COSY spectrum (CDCl3) of 5-methoxy-7-methyl-2-(4-morpholinophenyl)-4H-chromen-4-one (**29**).

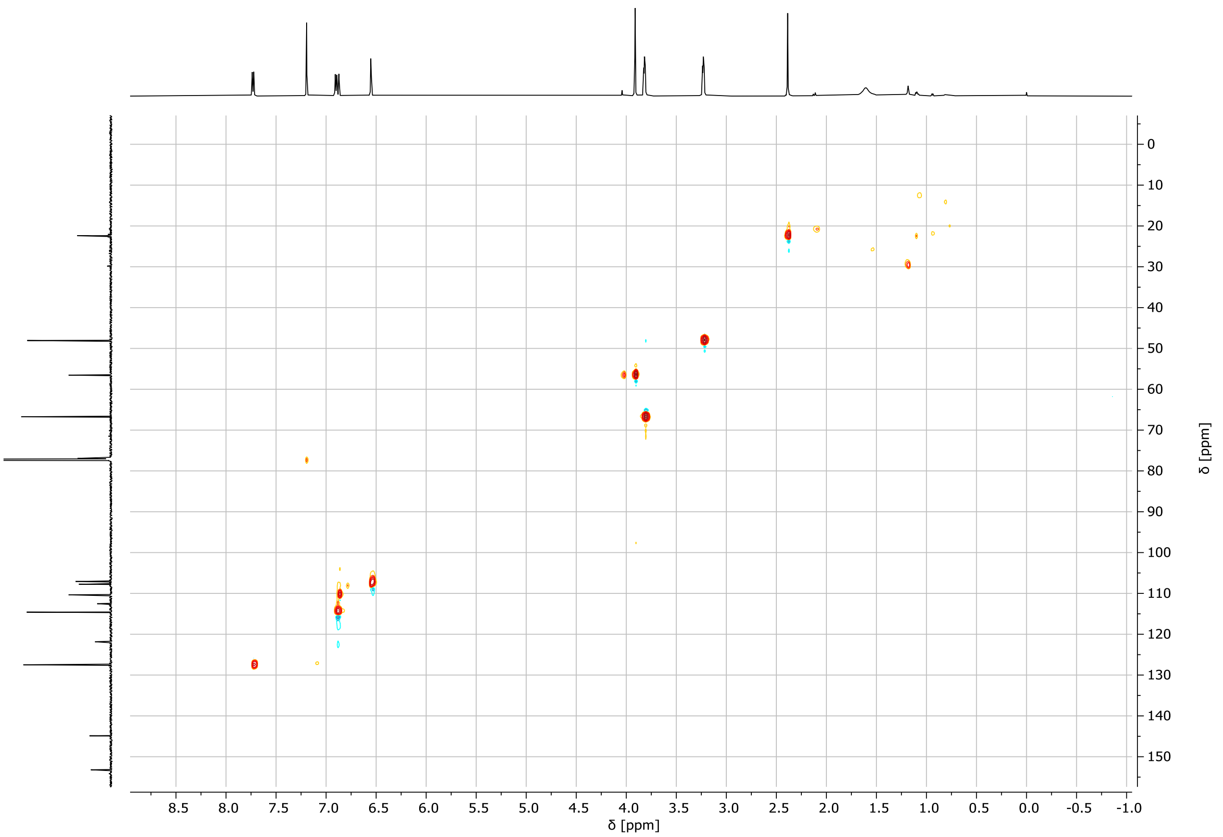


Figure S149: HSQC spectrum (CDCl3) of 5-methoxy-7-methyl-2-(4-morpholinophenyl)-4H-chromen-4-one (**29**).

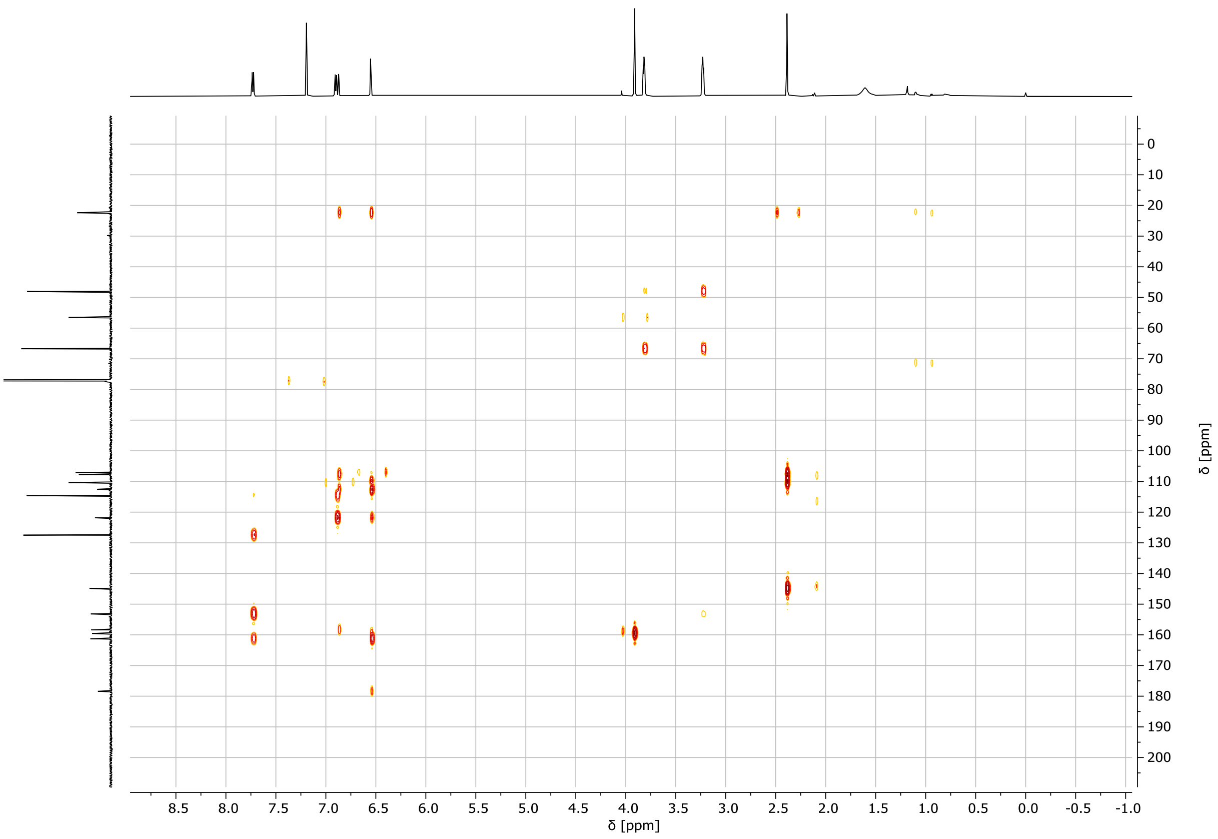


Figure S150: HMBC spectrum ($CDCl_3$) of 5-methoxy-7-methyl-2-(4-morpholinophenyl)-4H-chromen-4-one (**29**).

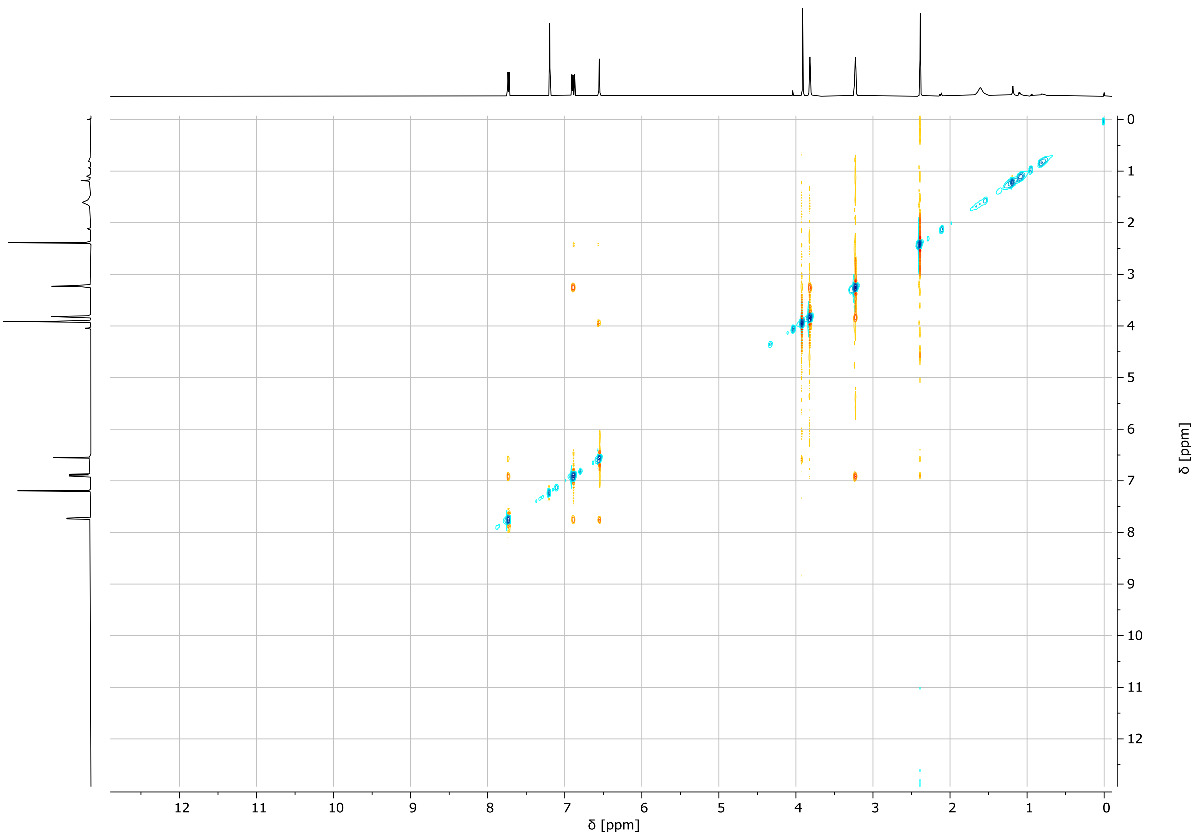


Figure S151: ROESY spectrum ($CDCl_3$) of 5-methoxy-7-methyl-2-(4-morpholinophenyl)-4H-chromen-4-one (**29**).

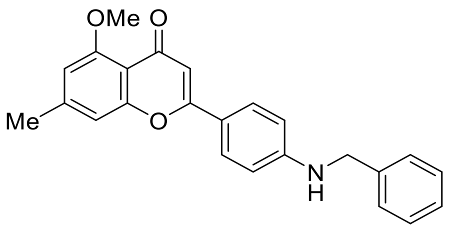


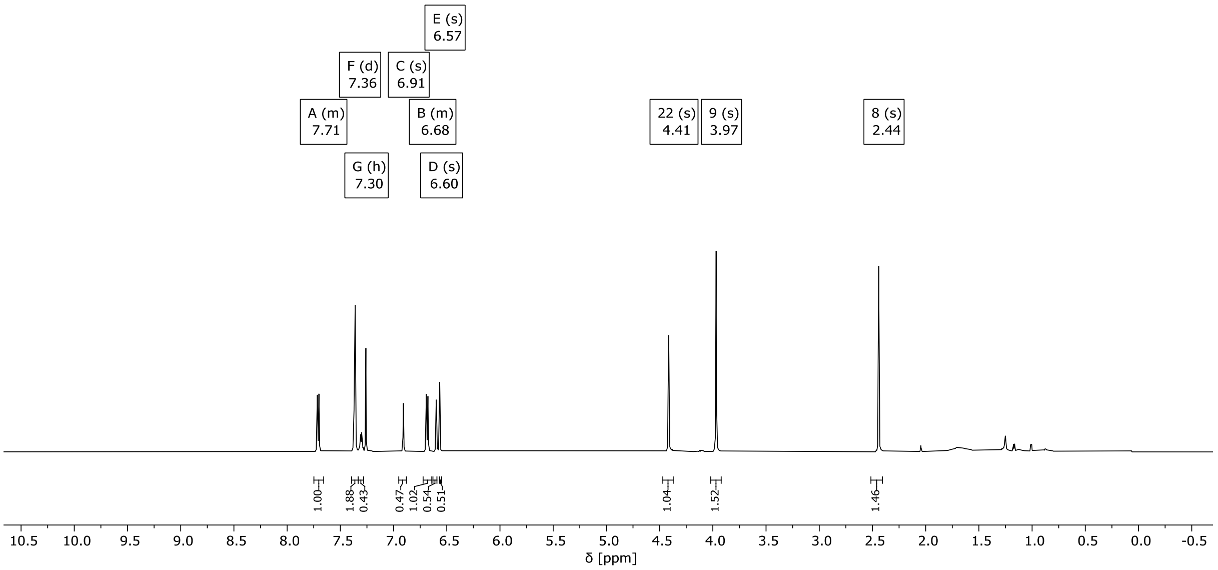


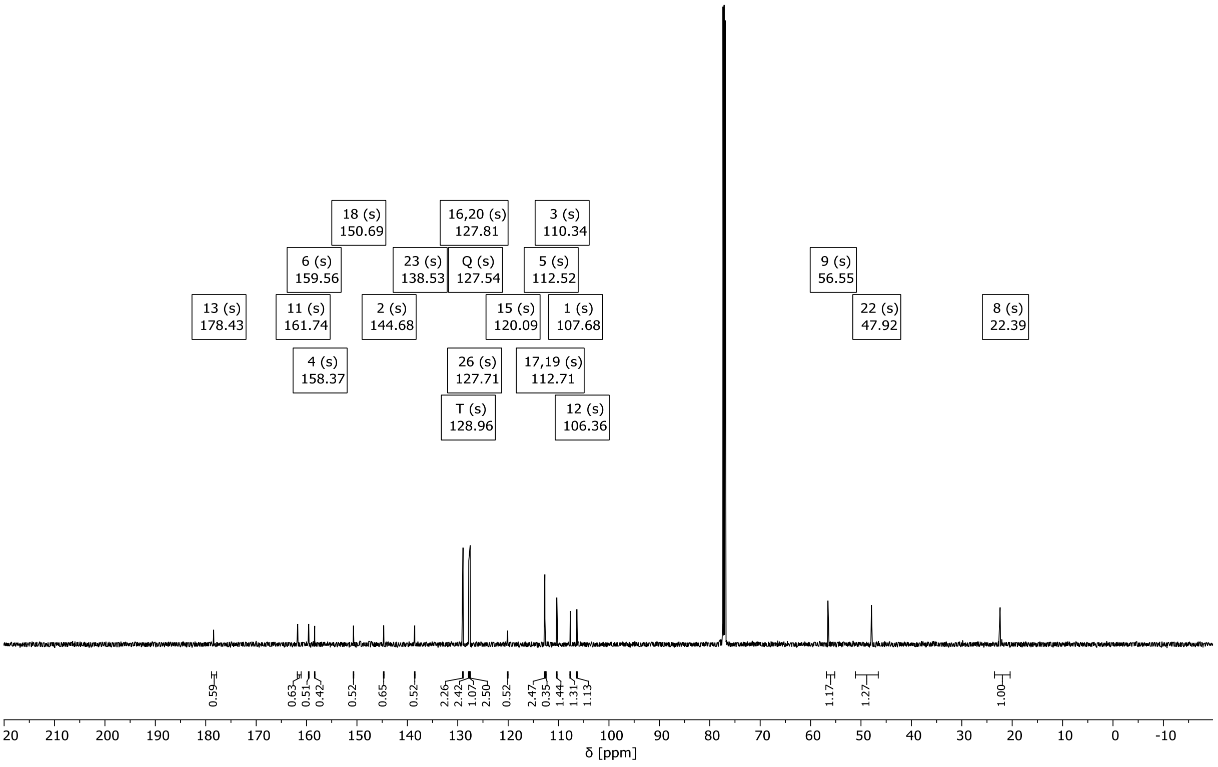


Figure S152: $^{1}$H- and $^{13}$C-NMR spectra (600 / 151 MHz, $CDCl_3$) of 2-(4-(benzylamino)phenyl)-5-methoxy-7-methyl-4H-chromen-4-one (**30**).

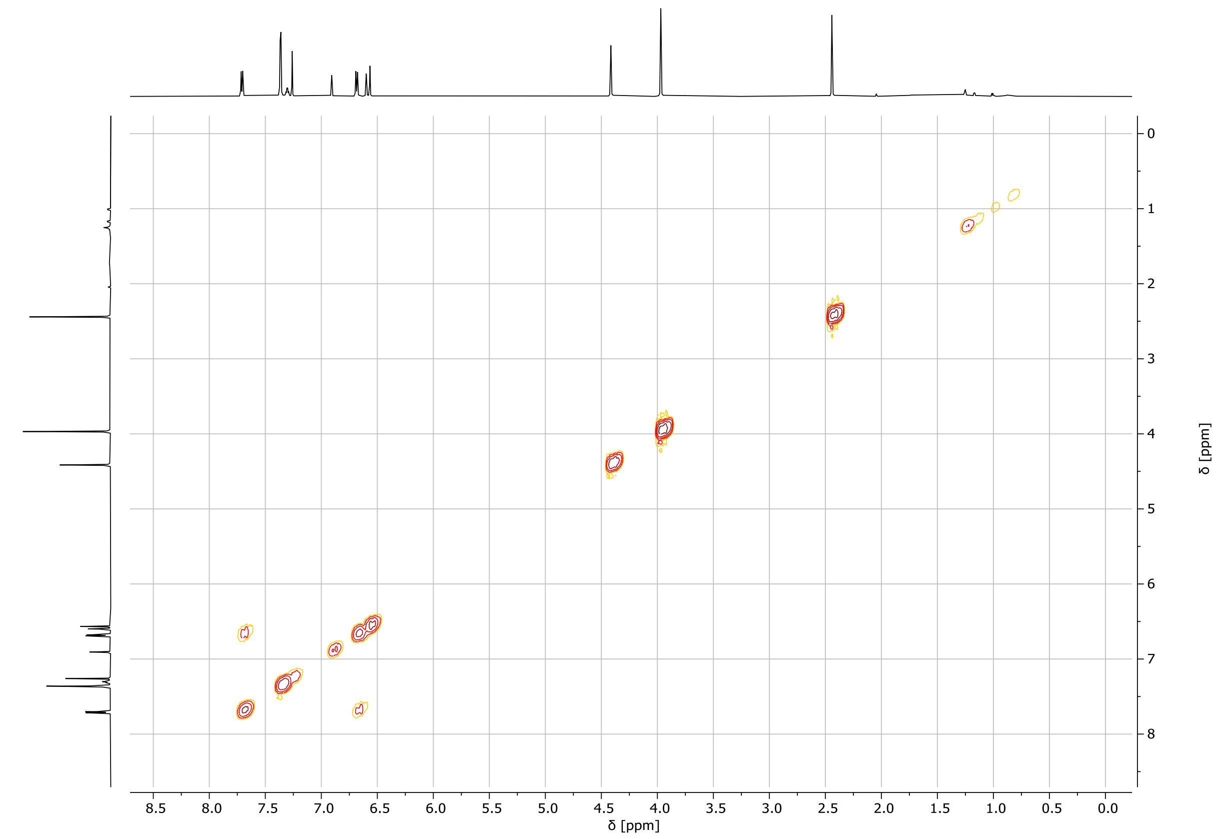


Figure S153: COSY spectrum ($CDCl_3$) of 2-(4-(benzylamino)phenyl)-5-methoxy-7-methyl-4H-chromen-4-one (**30**).

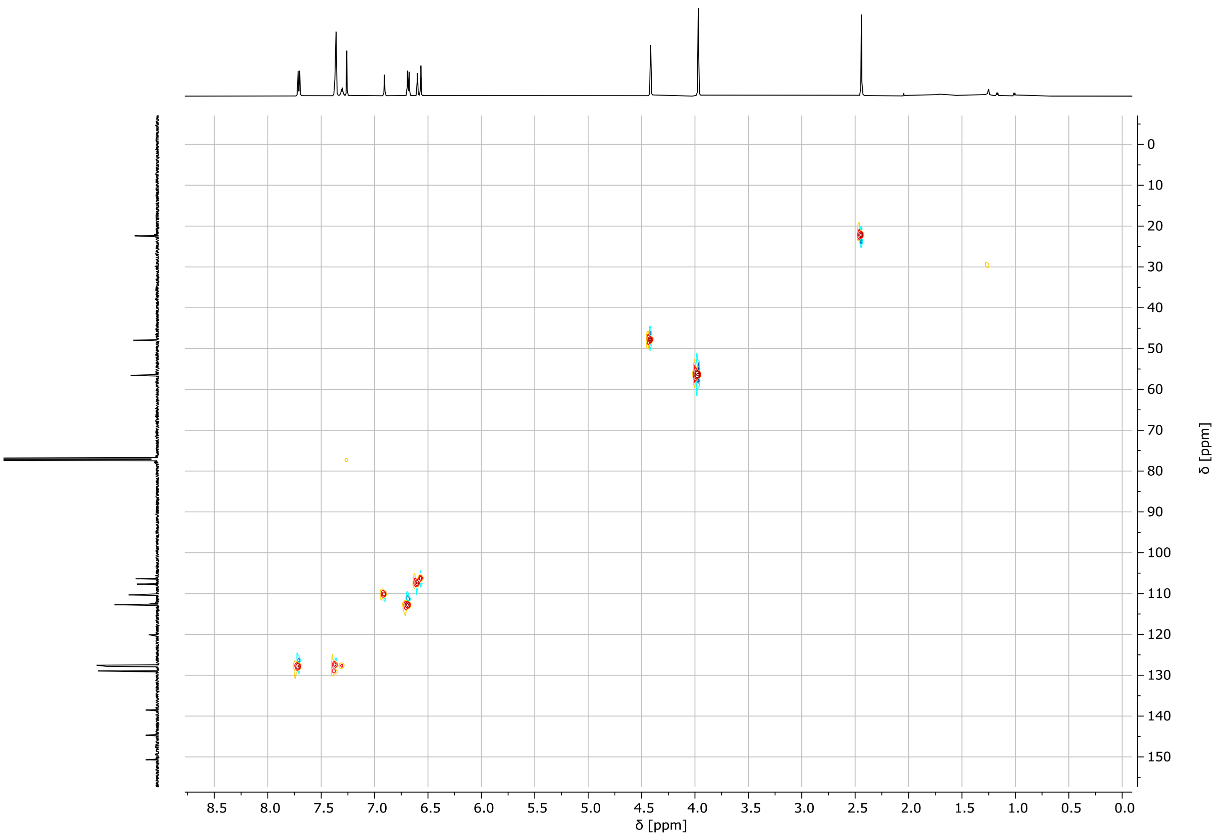


Figure S154: HSQC spectrum ($CDCl_3$) of 2-(4-(benzylamino)phenyl)-5-methoxy-7-methyl-4H-chromen-4-one (**30**).

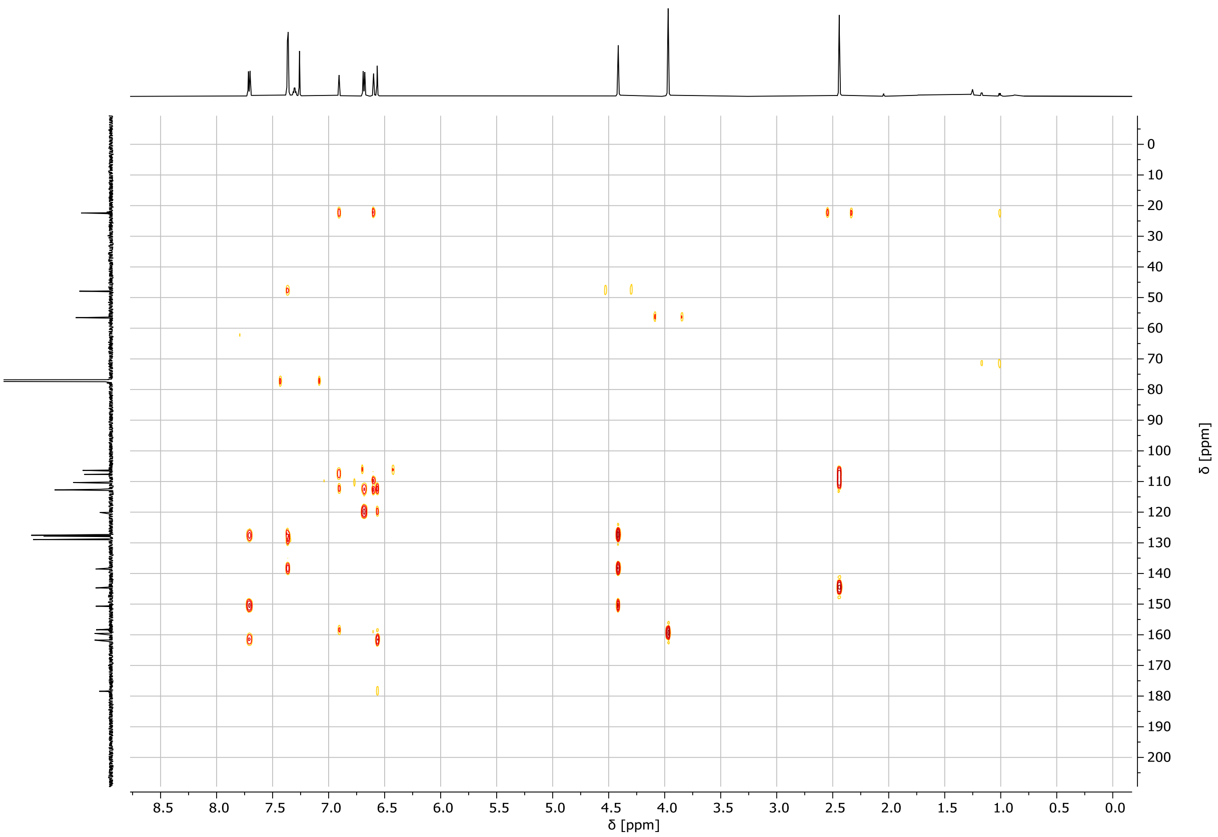


Figure S155: HMBC spectrum ($CDCl_3$) of 2-(4-(benzylamino)phenyl)-5-methoxy-7-methyl-4H-chromen-4-one (**30**).

# 4 X-Ray

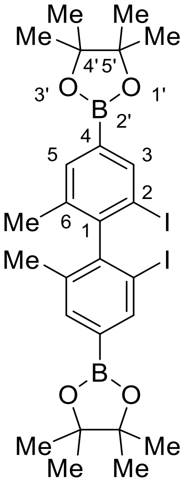


**9**

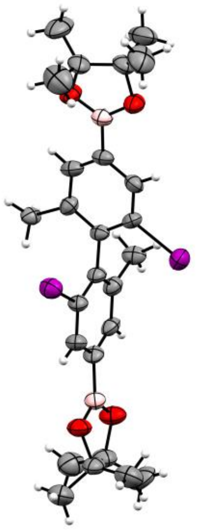

(*S*a)-**9**

CCDC 2342278

Figure S156: 2,2′-diiodobiaryl **9**. Left: Lewis structure interpretation Right: Crystal structure shown as Oak Ridge Thermal Ellipsoid Plot (ORTEP) of the (*S*)-enantiomer, ellipsoids are shown at 50% probability. Detailed information can be found in cif-format under the given CCDC deposition number.

Table S11: Crystal data and structure refinement for biaryl **9**.

| | |
|---|---|
| Empirical formula | $C_{26}H_{38}B_2NI_2O_4$ |
| Formula weight | 685.95 |
| Temperature | 291(2) K |
| Wavelength | 1.54178 Å |
| Crystal system, space group | Monoclinic, P2(1) |
| Unit cell dimensions | a = 12.1892(2) Å |
| | b = 18.2250(3) Å |
| | c = 14.2107(3) Å |
| | alpha = 90° |
| | beta = 114.6120(10)° |
| | gamma = 90° |
| Volume | 2870.07(9) $Å^3$ |
| Z | 4 |
| Calculated density | 1.587 $Mg/m^3$ |
| Absorption coefficient | 17.429 $mm^{-1}$ |
| F(000) | 1352 |
| Crystal size | 0.370 x 0.231 x 0.124 mm |
| Theta range for data collection | 3.421 to 66.038° |
| Limiting indices | -14<=h<=14, -21<=k<=18, -16<=l<=16 |
| Reflections collected / unique | 32238 / 9066 [R(int) = 0.0478] |
| Completeness to theta = 25.242 | 98.2 % |
| Absorption correction | Numerical |
| Max. and min. transmission | 0.3499 and 0.0304 |
| Refinement method | Full-matrix least-squares on $F^2$ |
| Data / restraints / parameters | 9066 / 51 / 657 |
| Goodness-of-fit on F^2 | 1.032 |
| Final R indices [I>2sigma(I)] | R1 = 0.0421, wR2 = 0.1051 |
| R indices (all data) | R1 = 0.0528, wR2 = 0.1104 |
| Largest diff. peak and hole | 0.762 and -0.471 e.$Å^{-3}$ |

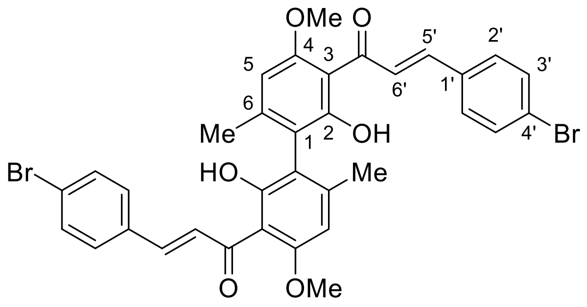


**13**

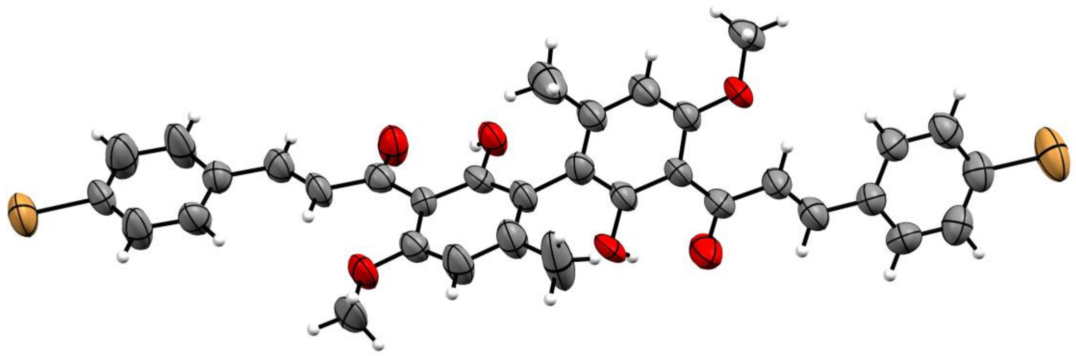

($R_a$)-**13 isomer 1**

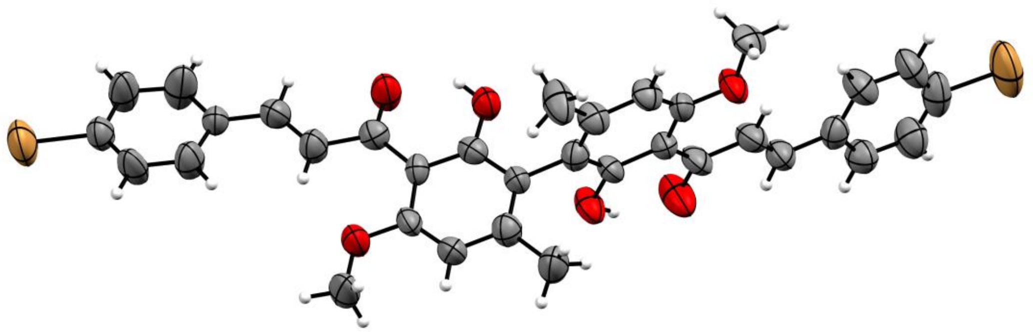

($R_a$)-**13 isomer 2**

CCDC 2342250

Figure S: Bichalcone **9**. Top Lewis structure interpretation Middle and bottom: Crystal structure (two conformers) shown as Oak Ridge Thermal Ellipsoid Plot (ORTEP) of the (*R*)-enantiomer, ellipsoids are shown at 50% probability. Detailed information can be found in cif-format under the given CCDC deposition number.

Table S12: Crystal data and structure refinement for bichalcone ($R_a$)-**13**.

| | |
|---|---|
| Empirical formula | $C_{34}H_{29}O_6Br_2$ |
| Formula weight | 692.38 |
| Temperature | 291(2) K |
| Wavelength | 0.71073 A |
| Crystal system, space group | Orthorhombic, P2(1)2(1)2(1) |
| Unit cell dimensions | a = 9.4449(2) Å |
| | b = 10.6758(3) Å |
| | c = 61.7848(15) Å |
| | alpha = 90° |
| | beta = 90° |
| | gamma = 90° |
| Volume | 6229.9(3) $Å^3$ |
| Z | 8 |
| Calculated density | 1.476 $Mg/m^3$ |
| Absorption coefficient | 2.646 $mm^{-1}$ |
| F(000) | 2800 |
| Crystal size | 0.213 x 0.196 x 0.115 mm |
| Theta range for data collection | 1.936 to 25.426° |
| Limiting indices | -11<=h<=11, -12<=k<=12, -57<=l<=73 |
| Reflections collected / unique | 38636 / 11402 [R(int) = 0.0496] |
| Completeness to theta = 25.242 | 99.9 % |
| Absorption correction | Numerical |
| Max. and min. transmission | 0.7553 and 0.6115 |
| Refinement method | Full-matrix least-squares on $F^2$ |
| Data / restraints / parameters | 11402 / 0 / 770 |
| Goodness-of-fit on F^2 | 1.028 |
| Final R indices [I>2sigma(I)] | R1 = 0.0548, wR2 = 0.1210 |
| R indices (all data) | R1 = 0.1109, wR2 = 0.1335 |
| Largest diff. peak and hole | 0.441 and -0.474 e.$Å^{-3}$ |

## 5 References


(1) Zhu, K.; Song, Z.; Wang, Y.; Zhang, F. Synthesis of 2,2′-dihalobiaryls via Cu-catalyzed halogenation of cyclic diaryliodonium salts. *Org. Lett.* **2020**, *22*, 9356-9359.
(2) Ke, J.; Zu, B.; Guo, Y.; Li, Y.; He, C. Hexafluoroisopropanol-Enabled Copper-Catalyzed Asymmetric Halogenation of Cyclic Diaryliodoniums for the Synthesis of Axially Chiral 2,2′-Dihalobiaryls. *Org. Lett.* **2020**, *23*, 329-333.
(3) Lindstrom, A. B.; Strynar, M. J.; Libelo, E. L. Polyfluorinated compounds: past, present, and future. *Envir. Sci. Technol.* **2011**, *45*, 7954-7961.
(4) Ishiyama, T.; Takagi, J.; Ishida, K.; Miyaura, N.; Anastasi, N. R.; Hartwig, J. F. Mild iridium-catalyzed borylation of arenes. High turnover numbers, room temperature reactions, and isolation of a potential intermediate. *J. Am. Chem. Soc.* **2002**, *124*, 390-391.
(5) Deng, H.; Bengsch, M.; Tchorz, N.; Neumann, C. N. Sterically Controlled Late‐Stage Functionalization of Bulky Phosphines. *Chem. Eur. J.* **2022**, e202202074.
(6) Maleczka, R. E.; Shi, F.; Holmes, D.; Smith, M. R. C−H activation/borylation/oxidation: A one-pot unified route to *meta*-substituted phenols bearing *ortho*-/*para*-directing groups. *J. Am. Chem. Soc.* **2003**, *125*, 7792-7793.
(7) Tlili, A.; Xia, N.; Monnier, F.; Taillefer, M. A very simple copper‐catalyzed synthesis of phenols employing hydroxide salts. *Angewandte Chemie* **2009**, *121*, 8881-8884.
(8) Cheung, C. W.; Buchwald, S. L. Palladium-catalyzed hydroxylation of aryl and heteroaryl halides enabled by the use of a palladacycle precatalyst. *J. Org. Chem.* **2014**, *79*, 5351-5358.
(9) Klischan, M. K. T.; Mazzone, F.; Berning, L.; Greb, J.; Schlamkow, M.; Haase, M.; Frey, W.; Stork, B.; Pfeffer, K.; Pietruszka, J. Modular Approach for the Synthesis and Bioactivity Profiling of 8,8′-Biflavones. *ACS Omega* **2023**, *8*, 41816–41834.
(10) Zhao, K.; Duan, L.; Xu, S.; Jiang, J.; Fu, Y.; Gu, Z. Enhanced reactivity by torsional strain of cyclic diaryliodonium in

Cu-catalyzed enantioselective ring-opening reaction. *Chem* **2018**, *4*, 599-612.
(11) Mislow, K.; Glass, M. A. W.; O'Brien, R. E.; Rutkin, P.; Steinberg, D. H.; Weiss, J.; Djerassi, C. Configuration, Conformation and Rotatory Dispersion of Optically Active Biaryls. *J. Am. Chem. Soc.* **1962**, *84*, 1455-1478.
(12) Greb, J.; Drennhaus, T.; Klischan, M. K. T.; Schroeder, Z. W.; Frey, W.; Pietruszka, J. A Common $C_2$‐Symmetric 2,2′‐Biphenol Building Block and its Application in the Synthesis of (+)‐di‐*epi*‐Gonytolide A. *Chem. Eur. J.* **2023**, *29*, e202300941.
(13) Kashikura, W.; Mori, K.; Akiyama, T. Chiral phosphoric acid catalyzed enantioselective synthesis of *β*-amino-*α*,*α*-difluoro carbonyl compounds. *Org. Lett.* **2011**, *13*, 1860-1863.
(14) Kanoh, S.; Tamura, N.; Motoi, M.; Suda, H. Optical Resolution and Absolute Configuration of Axially Dissymmetric 2,2′-Dihydroxy-6,6′-dimethylbiphenyl. *B. Chem. Soc. Jpn.* **1987**, *60*, 2307-2309.
(15) Liu, T. Z.; Lee, S. D.; Bhatnagar, R. S. Toxicity of palladium. *Toxoco. Lett.* **1979**, *4*, 469-473.
(16) Kielhorn, J.; Melber, C.; Keller, D.; Mangelsdorf, I. Palladium–a review of exposure and effects to human health. *Int. J. Hyg. Environ. Health* **2002**, *205*, 417-432.
(17) Schmid, M.; Zimmermann, S.; Krug, H. F.; Sures, B. Influence of platinum, palladium and rhodium as compared with cadmium, nickel and chromium on cell viability and oxidative stress in human bronchial epithelial cells. *Environ. Int.* **2007**, *33*, 385-390.
(18) Pohorilets, I.; Tracey, M. P.; LeClaire, M. J.; Moore, E. M.; Lu, G.; Liu, P.; Koide, K. Kinetics and inverse temperature dependence of a Tsuji–Trost reaction in aqueous buffer. *ACS Catal.* **2019**, *9*, 11720-11733.
(19) Song, F.; Garner, A. L.; Koide, K. A highly sensitive fluorescent sensor for palladium based on the allylic oxidative insertion mechanism. *J. Am. Chem. Soc.* **2007**, *129*, 12354-12355.
(20) Garner, A. L.; Song, F.; Koide, K. Enhancement of a catalysis-based fluorometric detection method for palladium

through rational fine-tuning of the palladium species. *J. Am. Chem. Soc.* **2009**, *131*, 5163-5171.
